# Electrostatically-directed Pd-catalysis in combination with C–H activation: site-selective coupling of remote chlorides with fluoroarenes and fluoroheteroarenes†

**DOI:** 10.1039/d0sc00105h

**Published:** 2020-02-18

**Authors:** William A. Golding, Robert J. Phipps

**Affiliations:** Department of Chemistry, University of Cambridge Lensfield Road Cambridge CB2 1EW UK rjp71@cam.ac.uk

## Abstract

Systems incorporating catalyst–substrate non-covalent interactions are emerging as a versatile approach to address site-selectivity challenges in remote functionalization reactions. Given the achievements that have been made in this regard using metals such as iridium, manganese and rhodium, it is surprising that non-covalent catalyst direction has not been utilized in reactions incorporating palladium-catalyzed C–H activation steps, despite palladium being arguably the most versatile metal for C–H activation. Herein, we demonstrate that electrostatically directed, site-selective C–Cl oxidative addition is compatible with a subsequent C–H activation step, proceeding *via* a concerted metalation deprotonation-type mechanism. This results in site-selective cross-coupling of dichloroarenes with fluoroarenes and fluoroheteroarenes, with selectivity controlled by catalyst structure. This study demonstrates that Pd-catalyzed C–H activation can be used productively in combination with a non-covalently-directed mode of catalysis, with important implications in both fields.

## Abstract 2

Electrostatically-directed oxidative addition is compatible with a subsequent C–H activation step, enabling site-selective coupling of remote chlorides with fluoroarenes and fluoroheteroarenes.

## Graphical abstract

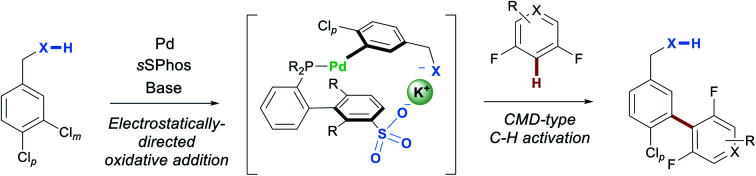

## Introduction

In recent years, non-covalent interactions have been increasingly explored as a powerful tool for modulating positional selectivity in transition-metal catalyzed reactions.^1^ Examples of reactions that have been investigated using this approach include hydroformylation,^2^ alkyne hydrometalation,^3^ cross-coupling^4^ and C–H activation.^5^ The latter is an area of chemistry in which control of positional selectivity is one of the defining challenges and which arguably has much to gain from the application of non-covalent strategies.^6^ To this end, iridium-catalyzed borylation of arenes has received the most attention to date, possibly due to its relatively mild reaction conditions, high functional group tolerance and compatibility with non-polar solvents.^7^ A broad spectrum of non-covalent interactions including hydrogen bonding,^8^ ion-pairing^9^ and electrostatic interactions^10^ have all been employed to direct the reactive iridium catalyst through substrate–ligand interactions.^11^ But it is conspicuous that little progress has been made on applying analogous non-covalent approaches to control site-selectivity in reactions involving palladium-catalyzed C–H activation processes.^12,13^ This is despite the rapid popularization of the use of ‘transient’ directing groups, which combine with the substrate in a reversible but covalent manner, to direct the Pd metal center in the C–H activation step.^14,15^ The inherent reversibility of non-covalent interactions makes them ideal to explore in this context. However, there is relatively little precedent for non-covalent catalyst direction being used in tandem with palladium-catalyzed C–H activation. An interesting recent example from Crimmin and co-workers suggested dispersion interactions can influence regioselectivity in the palladium-catalyzed C–H alumination of toluene.^16^ If compatibility could be more broadly demonstrated it would pave the way for new and innovative design strategies incorporating non-covalent design elements for controlling site-selectivity in Pd-catalyzed C–H activation reactions.

**Fig. 1:**
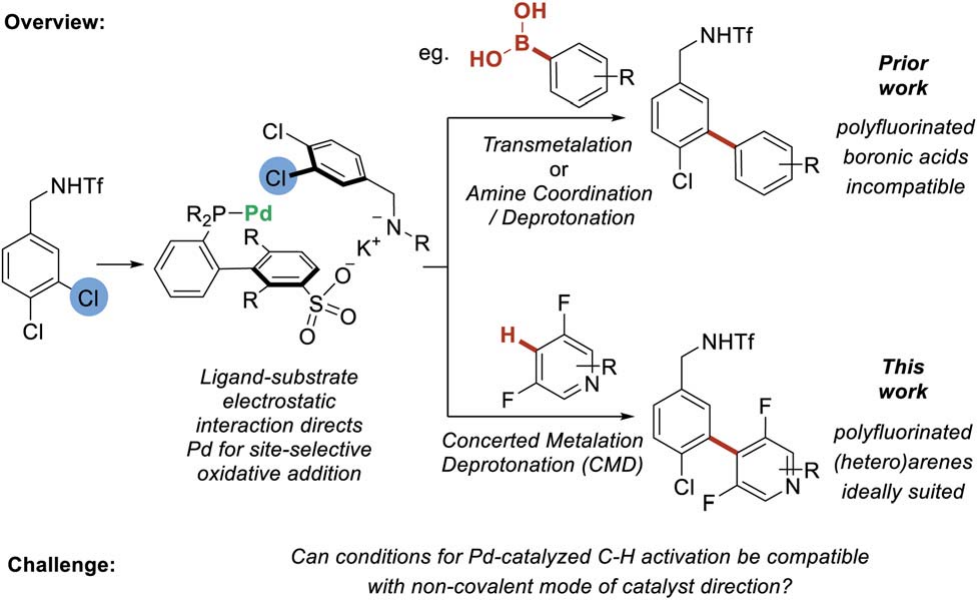
Proposed integration of C–H activation of fluoroarenes with an electrostatically-directed site-selective oxidative addition.

We recently reported that sulfonated phosphine ligands can be used to direct site-selective palladium-catalyzed cross coupling on substrates featuring remote chlorides that would be very challenging to differentiate using existing methods.^4^*s*SPhos is a commercially available, water-soluble phosphine that we repurposed such that the sulfonate group engages in a non-covalent interaction with the substrate.^17^ Experiments provided support for a scenario wherein the potassium cation of the deprotonated substrate interacts with the sulfonate group of the ligand, leading to oxidative addition being directed to the C–Cl bond at the substrate *meta* position (Fig. 1, *upper pathway*). Following this, transmetalation or amine coordination/deprotonation was followed by reductive elimination to typically give a single regioisomer as product. At the outset of this work we questioned whether it might be possible to replace the transmetalation step with a CMD step to enable C–H bond activation to occur on the coupling partner. This would not only increase the efficiency of the C–C bond formation by avoiding prefunctionalization of one reactant, but more importantly would demonstrate proof-of-concept that CMD is compatible with non-covalent catalyst direction, in this case to control site-selectivity in oxidative addition to the C–Cl bond. Proof that this is viable may have broader implications for palladium-catalyzed C–H activation as it could act as a stepping-stone to non-covalent catalysis being applied for control of site-selectivity in the challenging C–H activation step of a catalytic cycle (*vide supra*). To test our proposal, we sought to examine fluoroarenes and fluoroheteroarenes as coupling partners (Fig. 1, *lower pathway*). In addition to being well suited to CMD with Pd-catalysis,^18^ success with this substrate class would also provide complementarity to our previous work. We had attempted to use polyfluorophenyl boronic acids, trifluoroborate salts and MIDA-boronates as coupling partners under our previous conditions but observed no conversion in all cases.^19^ This was also the case when using the *s*SPhos G2 pre-catalyst (see ESI† for full details). Hence, development of a C–H activation variant would be of practical utility, allowing access to these fluorinated biaryl products in a selective manner.

## Results and discussion

When approaching the optimization of the proposed reaction, our primary concern was whether the conditions required for effective C–H activation might interfere with the electrostatic interaction presumed to be responsible for the site-selectivity in the oxidative addition step. We commenced our investigations with triflate-protected dichlorobenzylamine **1a** and pentafluorobenzene (**2a**) (Table 1). Under the conditions that we had previously used for site-selective Suzuki coupling, no product formation was observed (entry 1). Consulting the conditions optimized by Fagnou and co-workers in their seminal work on perfluoroarene C–H activation, we switched the solvent to *i*PrOAc but still observed no product formation (entry 2).^18*a*,18*b*^ Addition of substoichiometric amounts of pivalic acid was found to be beneficial, in accordance with previous observations.^20^ Good yield was obtained with 60 mol% PivOH and, importantly, excellent levels of site-selectivity for coupling of the chloride at the *meta* position was obtained (14 : 1, entries 3 and 4). The yield could be improved by switching from the typical sodium salt of *s*SPhos to the more soluble tetrabutylammonium salt, *s*SPhos(NBu_4_) (entry 5). Finally, an evaluation of Pd sources showed that [(Cinnamyl)PdCl]_2_ gave improved yield (entry 6). When switching the ligand to standard SPhos the site-selectivity dropped to 1.2 : 1, in line with the hypothesis that the sulfonate group on the ligand is crucial for this outcome (entry 7). To probe the effect of having an arene sulfonate moiety present but detached from the ligand structure, we carried out the reaction using 20 mol% SPhos with 20 mol% potassium 2,4-dimethoxybenzenesulfonate as an additive (entry 8). This showed similarly low site-selectivity as observed when using SPhos. The equivalents of pentafluorobenzene could be reduced to three equivalents before significant effect on yield was observed (entries 9 and 10). However, for the purposes of scope exploration, nine equivalents were retained in the remainder of the studies, due to anticipated lower reactivity of some lesser fluorinated arenes.

**Table 1:** Optimization of site-selective coupling between A and B^a^

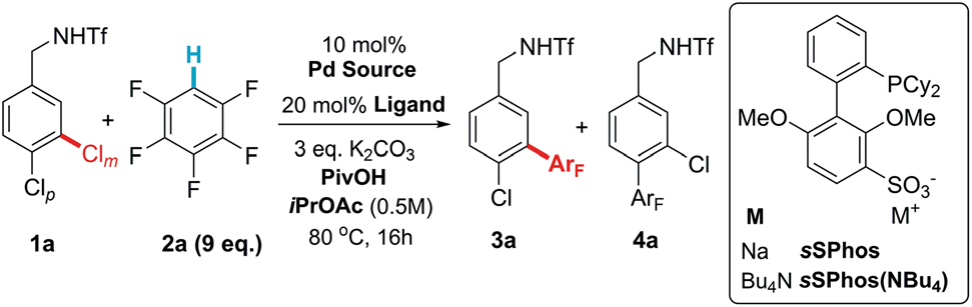					
Entry	Pd Source	Ligand	Eq. PivOH	% Conv.	**3** : **4**
1^b^	Pd(OAc)_2_	*s*SPhos	0	0	N/A
2	Pd(OAc)_2_	*s*SPhos	0	0	N/A
3	Pd(OAc)_2_	*s*SPhos	0.3	48	14 : 1
4	Pd(OAc)_2_	*s*SPhos	0.6	56	14 : 1
5	Pd(OAc)_2_	*s*SPhos(NBu_4_)	0.6	78	16 : 1
6	[(Cin)PdCl]_2_	*s*SPhos(NBu_4_)	0.6	100	15 : 1
7	[(Cin)PdCl]_2_	SPhos	0.6	100	1.2 : 1
8^c^	[(Cin)PdCl]_2_	SPhos	0.6	100	1.2 : 1
9^d^	[(Cin)PdCl]_2_	*s*SPhos(NBu_4_)	0.6	88	16 : 1
10^e^	[(Cin)PdCl]_2_	*s*SPhos(NBu_4_)	0.6	82	13 : 1

a Ratios and conversions determined by ^1^H-NMR analysis with internal standard.

b THF as solvent.

c Potassium 2,4-dimethoxybenzenesulfonate included as additive (20 mol%).

d 6 eq. **2a** used.

e 3 eq. **2a** used.

**Scheme 1:**
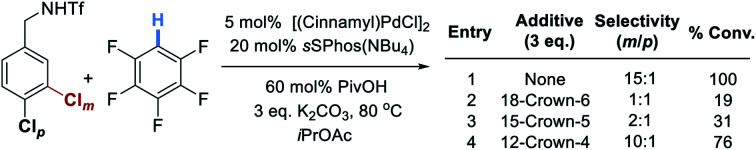
Evaluation of various sizes of crown ether as additives under the optimized conditions.

To provide support for the proposed electrostatic interaction, in which the potassium cation is thought to play a crucial role, we carried out experiments wherein stoichiometric amounts of various crown ethers are added under the optimized reaction conditions (Scheme 1). These showed that addition of 18-Crown-6, which is best able to bind potassium, resulted in no selectivity and poor conversion (entry 2). As the crown ether was made smaller and less able to bind potassium, selectivity and reactivity were restored (entries 3 and 4).

**Scheme 2:**
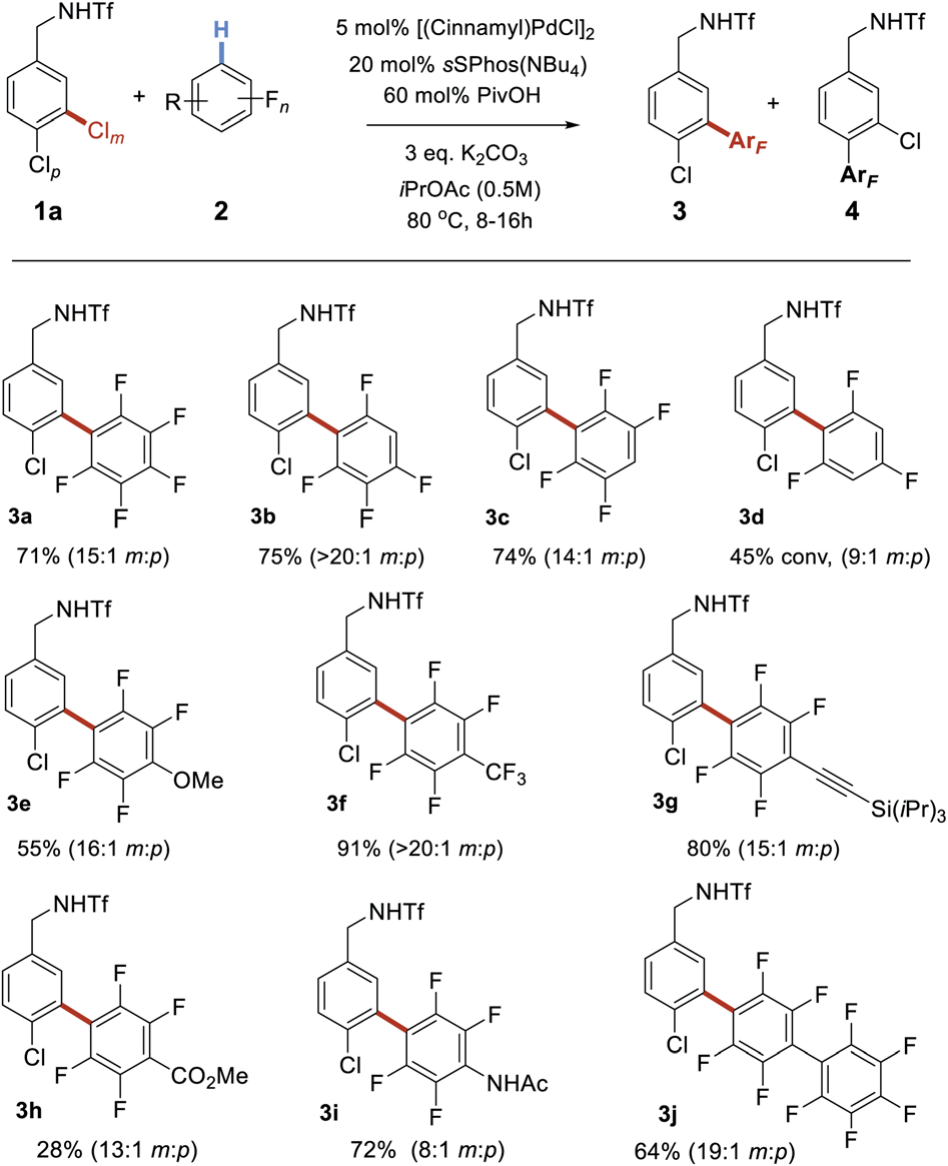
Scope of the perfluoroarene reaction component in coupling with **1a**.

Having optimized conditions for the site-selective coupling of **1a** with pentafluorobenzene, we next evaluated the scope of the perfluoroarene component (Scheme 2). Undesired proteodechlorination of the reaction products was observed in some cases, however this deleterious pathway could be largely avoided by shortening the reaction time (see ESI† for full details). Isomeric tetrafluoroarenes underwent efficient coupling in high yield and with excellent site-selectivity (**3b** and **3c**). 1,3,5-Trifluorobenzene represented the limit of reactivity (**3d**); whilst selectivity was high, conversion was moderate and such a drop-off of reactivity is precedented.^18*a*^ Tetrafluorobenzenes bearing various substituents reacted smoothly and the scope encompassed methoxy (**3e**), trifluoromethyl (**3f**), alkynyl (**3g**), ester (**3h**) and acetamide (**3i**) groups. A further perfluoroarene could be incorporated without issue (**3j**). It is interesting to note that the site-selectivity, whilst typically >10 : 1, does vary to some degree with the fluoroarene partner, despite the selectivity-determining step occurring in the oxidative addition. We attribute this small variation to a solvent effect – as the reactions are relatively concentrated (0.5 M) and nine equivalents of arene are being used, this typically means a ∼1 : 1 ratio by volume of fluoroarene : solvent. It is therefore foreseeable that changes in the fluoroarene component could be manifested in minor fluctuations in oxidative addition regioselectivity.

**Scheme 3:**
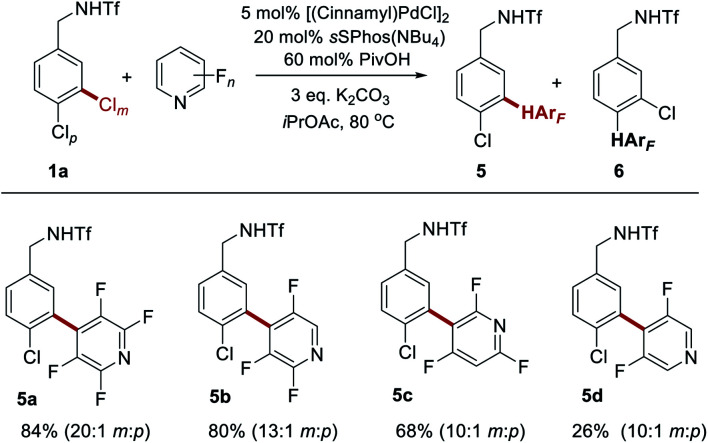
Evaluation of fluorinated pyridines as coupling partners with **1a**.

We next sought to determine whether the reaction would be compatible with fluorinated heteroarenes and so evaluated several variously fluorinated pyridines (Scheme 3). Pleasingly, 2,3,5,6-tetrafluoropyridine was found to give excellent yield and site-selectivity (**5a**). Two different constitutional isomers of trifluorinated pyridine also reacted well, with C–H activation viable at either the C4- (**5b**) or C3- (**5c**) position relative to the pyridine nitrogen, dependant on the nature of the fluorination. Even 3,5-difluoropyridine reacted, albeit with low conversion (**5d**). In this case presumably the inductive withdrawal of the pyridine nitrogen compensates for the low degree of fluorination, still permitting some reactivity to be obtained, and representing the current limit of reactivity according to the present protocol.

**Scheme 4:**
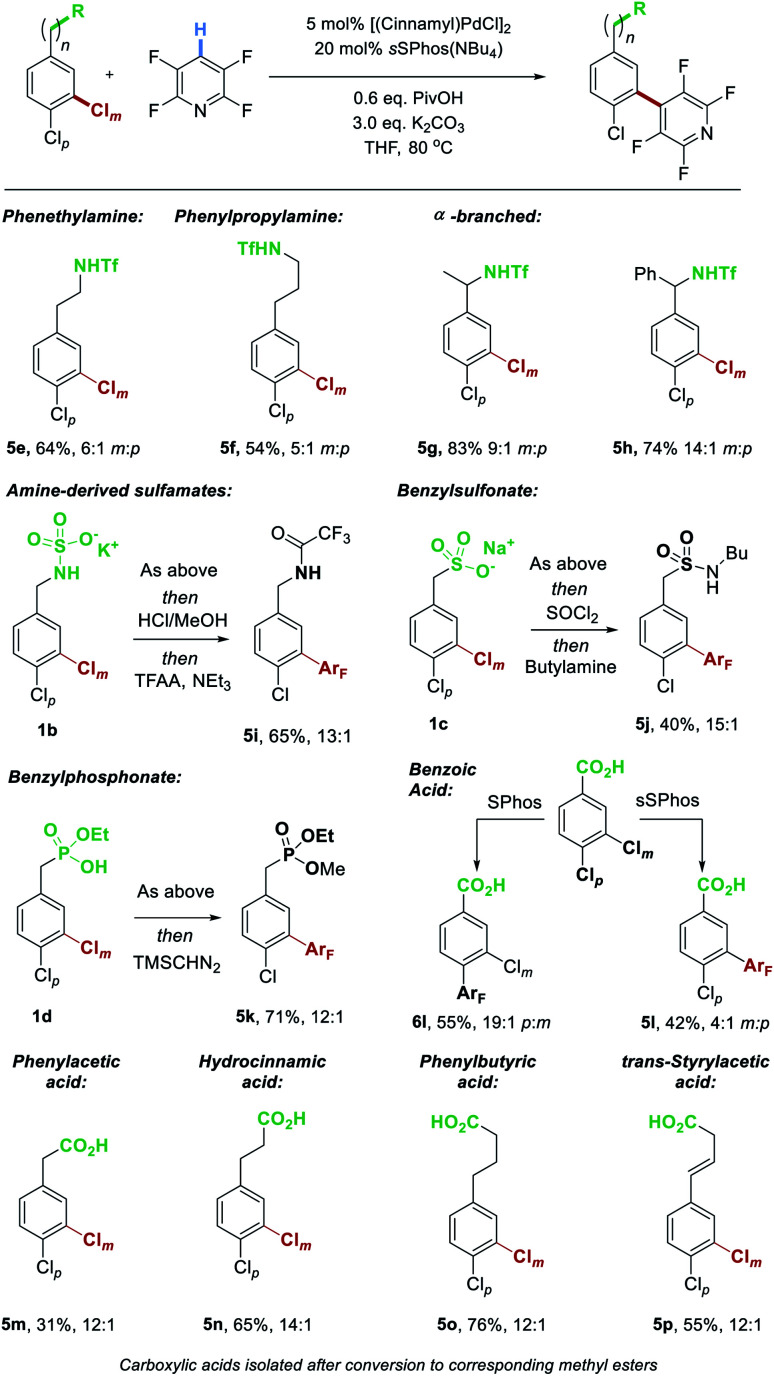
Evaluation of various Brønsted acidic groups able to interact with catalyst resulting in selectivity for reaction at the *meta*-chloride.

We next explored the scope of the aryl chloride component (Scheme 4). Extending the chain length to encompass phenethylamine (**5e**) and phenylpropylamine (**5f**) derivatives was tolerated, with reduced but still synthetically useful levels of site-selectivity. We found that benzylic substitution poses no problem for the catalyst control with both methyl (**5g**) and phenyl (**5h**) groups at that position giving excellent results.

Whilst the triflyl-protected benzylamines can be manipulated post-reaction, obtaining the free amine is challenging. We demonstrate that the parent benzylamine can be readily converted to the corresponding potassium sulfamate salt by treatment with chlorosulfonic acid followed by potassium hydroxide.^11*a*^ This sulfamate salt **1b** undergoes highly site-selective coupling with tetrafluoropyridine (13 : 1). The product was subjected to acid-promoted sulfamate cleavage followed by trifluoroacetylation, to enable isolation as amide **5i**. As found previously with cross-coupling, a sulfonate as well accommodated as an electrostatic directing group in this C–H activation reaction, giving excellent selectivity for coupling at Cl_*m*_ (**5j**, after conversion to the corresponding sulfonamide). Herein, we also demonstrate that a mono-basic benzyl phosphonate also results in excellent site-selectivity, with the coupled product isolated after methylation as **5k**. Perhaps the most commonly encountered Brønsted acidic functional group, the carboxylic acid, functions as an excellent electrostatic directing group, tolerating a variety of chain lengths. Chlorinated phenyl acetic acid (**5m**), hydrocinnamic acid (**5n**) and phenylbutyric acid (**5o**) all resulted in excellent site-selectivity. Furthermore, an alkene could be tolerated in the chain without side reactions and with no erosion of site-selectivity (**5p**). We next sought to evaluate the ability of our ligand to override a substrate's innate selectivity. As anticipated, coupling of 3,4-dichlorobenzoic acid with tetrafluoropyridine using SPhos as ligand gave very high selectivity for the *para*-chloride (**6l**), presumably due to substrate electronics rendering this the most electron deficient of the two C–Cl bonds. In contrast, simply switching the ligand to *s*SPhos rendered a switch in site-selectivity giving a remarkable 4 : 1 ratio of coupling at Cl_*m*_*vs.* Cl_*p*_, and **5l** as major product.

**Scheme 5:**
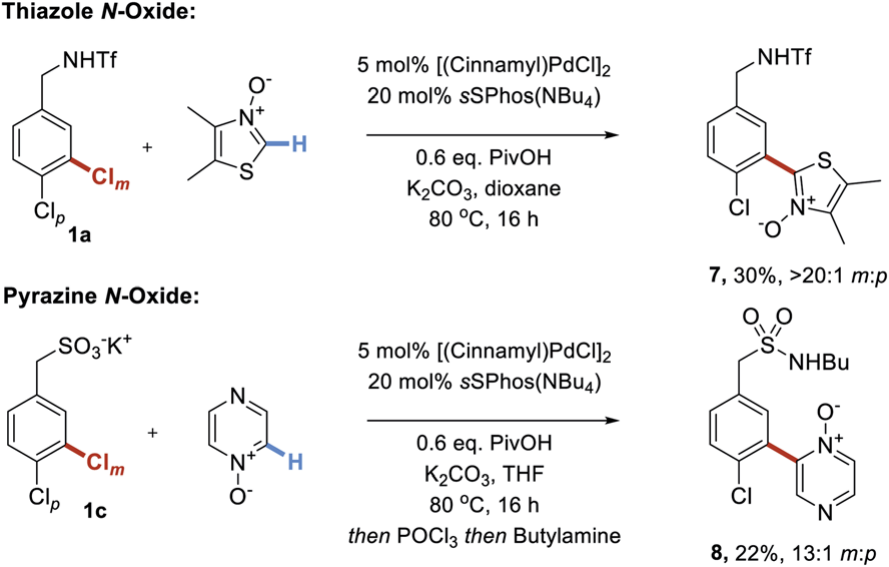
Unoptimized couplings of two examples of heterocyclic *N*-oxides with two different classes of substrate.

Finally, we disclose that thiazole *N*-oxides and pyrazine *N*-oxides are viable C–H activation partners to be used in place of the fluoroarenes (Scheme 5).^21^ These were demonstrated on two different substrate classes of aryl chloride, triflamide **1a** and sulfonate **1c**. Although the conversions were only moderate under the conditions optimized for the perfluoroarenes, importantly site-selectivity was excellent in both cases and these promising results demonstrate the broader potential of this strategy.

## Conclusions

In summary, we have demonstrated that electrostatically-directed palladium catalysis using sulfonylated phosphine ligands is compatible with CMD-type C–H activation in a single catalytic cycle. This results in a highly site-selective fluoroarylation of a number of substrates bearing two undifferentiated C–Cl bonds, with anionic handles for the electrostatic interaction encompassing triflamide, carboxylate, phosphonate, sulfonate and sulfamate. From a practical viewpoint, this work expands the scope of coupling partners able to engage in this site-selective coupling, since fluoroaryl boronic acids were incompatible with the cross-coupling protocol. More importantly though, this is a powerful demonstration that non-covalently-directed catalysis for control of site-selectivity can be used in conjunction with C–H activation. This paves the way for future, related developments in which site-selectivity may be controlled in the C–H activation step itself.

## Conflicts of interest

There are no conflicts to declare.

## Supplementary Material

SC-011-D0SC00105H-s001
