# Supplementary material for: Electrostatically-directed Pd-catalysis in combination with C–H activation: site-selective coupling of remote chlorides with fluoroarenes and fluoroheteroarenes
Source: Chem Sci. 2020 Feb 18;11(11):3022–7. doi: 10.1039/d0sc00105h (PMC8157713; doi:10.1039/d0sc00105h)

Electronic Supplementary Material (ESI) for Chemical Science.
This journal is © The Royal Society of Chemistry 2020

Electrostatically-Directed Pd-Catalysis in Combination with C-H Activation: Site-Selective Coupling of Remote Chlorides with Fluoroarenes and Fluoroheteroarenes

William A. Golding and Robert J. Phipps*

Department of Chemistry, University of Cambridge, Lensfield Road, Cambridge, CB2 1EW, UK. E-mail: rjp71@cam.ac.uk..

Contents

2. General Information 3
3. Optimisation Tables 4
4. Ligand Synthesis 7
5. Synthesis of starting materials 8
6. Fluorinated arene scope 17
7. Fluorinated pyridine scope 23
8. Dichloroarene scope 25
9. N-oxides 34
10. Crown ether experiments 36
11. BF_3K salts and Mida boronate ester 37
12. References 39
13. NMR spectra 40

2. General Information

NMR spectra: ^{1}H NMR spectra were recorded on a 600 MHz Bruker Avance DRX-600 spectrometer, a 500 MHz DCH Cryoprobe spectrometer, a 400 MHz Avance III HD Smart probe spectrometer, and a 400 MHz QNP Cryoprobe spectrometer. Chemical shifts are reported in parts per million (ppm) and the spectra are calibrated to the resonance resulting from incomplete deuteration of the solvent ($CDCl_3$: 7.26 ppm, CD_3OD: 3.31 ppm, $(CD_3)_2CO$: 2.05). ^{13}C NMR spectra were recorded with the same spectrometers with complete proton decoupling. Chemical shifts are reported in ppm with the solvent resonance as the internal standard ($^{13}CDCl_3$: 77.16 ppm, t; $^{13}CD_3OD$: 49.00, sept; $^{13}(CD_3)_2CO$: 29.84, sept, 203.26, s). Data are reported as follows: chemical shift δ/ppm, integration (^{1}H only), multiplicity (s = singlet, d = doublet, t = triplet, q = quartet, quin = quintet, sept = septet, br = broad, m = multiplet or combinations therof; ^{13}C signals are singlets unless stated otherwise), coupling constants *J* in Hz. ^{1}H-COSY, DEPT-135, HMQC, HMBC and NOSEY were used where appropriate to facilitate structural determination of regioisomers. ^{19} F NMR spectra were recorded on a 400 MHz Avance III HD Smart probe spectrometer and were proton decoupled. ^{31}P NMR spectra were recorded on a 600 MHz Bruker Avance DRX-600 spectrometer on a 400 MHz Avance III HD Smart probe spectrometer.

High Resolution Mass Spectrometry (HRMS): Samples were recorded on a Waters Micromass LCT Premier *or* a Waters Xevo G2-S *or* a Waters Vion QTof spectrometer using a positive electrospray ionization (ESI+). The measured values are reported to 4 decimal places and are within ±5 ppm of the calculated value. The calculated values are based on the most abundant isotope

Chromatography: Analytical thin layer chromatography was performed using precoated Merck glass backed silica gel plates (Silicagel 60 F254). Visualisation was by ultraviolet fluorescence (λ = 254 nm) and/or staining potassium permanganate ($KMnO_4$). Flash column chromatography was performed using silica gel 60 (0.040-0.063 µm) from Material Harvest.

Reagents: Unless stated otherwise were used as supplied from commercial sources without further purification. CH_2Cl_2, THF, Et_2O were purified by distillation on site under an inert atmosphere via the following processes: THF and Et_2O were pre-dried over sodium wire then distilled from calcium hydride and lithium aluminium hydride. CH_2Cl_2, *n*-hexane and toluene were distilled from calcium hydride.

3. Optimisation Tables

Table S1. Evaluation of additives

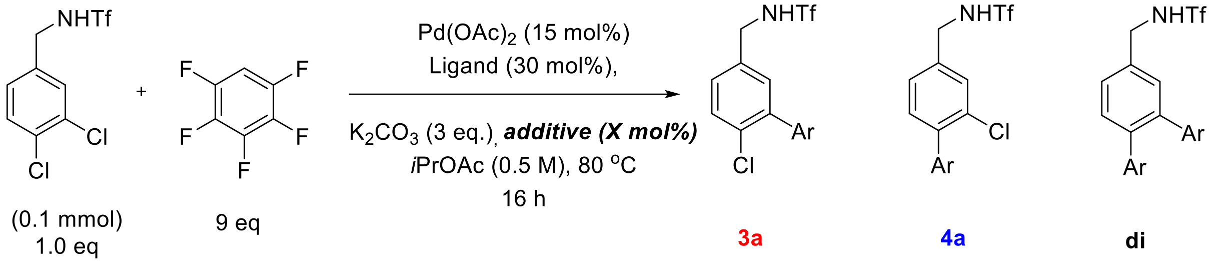

3a **4a** **di**

Entry	Ligand	Additive (**X** mol%)	% 3a	% 4a	% di	3a:4a	% SM	% conv
1	SPhos	**None**	13	10	-	**1.3:1**	76	**23**
2	SPhos	**AdCOOH (30 mol%)**	38	36	2	**1.1:1**	11	**87**
3	SPhos	**AcOH (60 mol%)**	30	25	-	**1.2:1**	45	**55**
4	SPhos	**PivOH (30 mol%)**	42	37	5	**1.1:1**	1	**99**
5	sSPhos	**PivOH (30 mol%)**	74	5	-	**15:1**	4	**95**

Yields determined by ^{1}H-NMR with dimethoxyethane (0.033 mmol) as internal standard

Table S2. Catalyst Loading

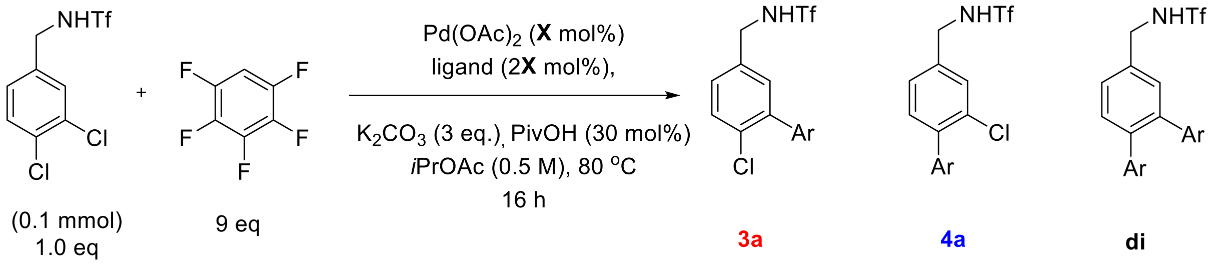

3a **4a** **di**

Entry	Ligand	Loading (**X** mol %)	% 3a	% 4a	% di	3a:4a	% SM	% conv
1	sSPhos	**15**	74	5	-	15:1	4	**95**
2	sSPhos	**10**	45	3	-	14:1	52	**46**
3	sSPhos	**5**	5	-	-	N/A	94	**5**
4	SPhos	**30**	28	31	7	1:1.1	-	**100**
5	SPhos	**15**	41	39	-	1:1	7	**92**
6	SPhos	**10**	43	36	3	1.2:1	9	**90**
7	SPhos	**5**	25	22	-	1.2:1	38	**55**

Yields determined by ^{1}H-NMR with dimethoxyethane (0.033 mmol) as internal standard

Table S3. Evaluation of pivalic acid loading at 10 mol% palladium

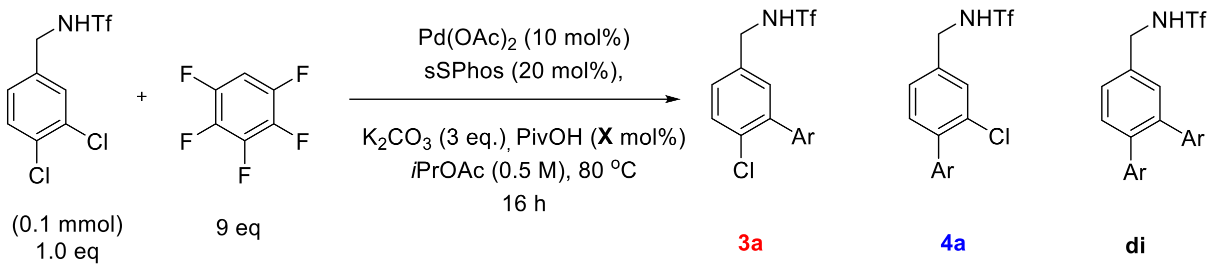

(0.1 mmol) 1.0 eq | 9 eq | **3a** | **4a** | **di**

Entry	Ligand	Loading (**X** mol%)	% 3a	% 4a	% di	3a:4a	% SM	% conv
1	sSPhos	**10**	2	-	-	N/A	80	**3**
2	sSPhos	**20**	18	>1	-	15:1	66	**21**
3	sSPhos	**30**	30	2	-	14:1	55	**35**
4	SPhos	**40**	33	2	-	16:1	56	**38**
5	SPhos	**60**	52	3	-	16:1	34	**62**

Yields determined by ^{1}H-NMR with dimethoxyethane (0.033 mmol) as internal standard

Table S4. Evaluation of palladium source

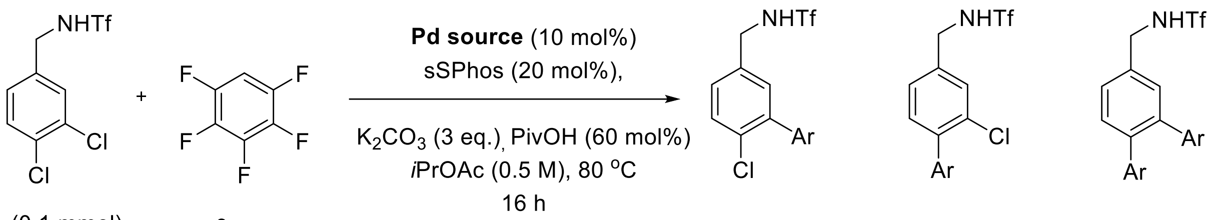

(0.1 mmol) 1.0 eq | 9 eq | **3a** | **4a** | **di**

Entry	Palladium source	% 3a	% 4a	% di	3a:4a	% SM	% conv	Comments
1	$Pd(OAc)_2$	59	3	-	**17:1**	36	**63**	
2	$Pd_2(dba)_3$	50	3	-	**15:1**	42	**56**	
3	$Pd(MeCN)_2Cl_2$	66	4	-	**17:1**	20	**78**	
4	Pd acetylacetonate	-	-	-	**-**	93	**-**	
5	$Pd(TFA)_2$	84	5	-	**17:1**	10	**90**	
6	Palladium(π-cinnamyl) chloride dimer	75	4	-	**17:1**	-	**100**	
7	$Pd(Piv)_2$	76	5	-	**16:1**	13	**86**	
8	$Pd(OAc)_2$	67	4	-	**16:1**	21	**78**	**sSPhos$_{(NBu4)}$ used instead**
9	Palladium(π-cinnamyl) chloride dimer	81	5	1	**15:1**	0	**100**	**sSPhos$_{(NBu4)}$ used instead**

Yields determined by ^{1}H-NMR with dimethoxyethane (0.033 mmol) as internal standard

Table S5. Optimum reaction time

When exploring the scope of the reaction, some less successful couplings resulted in hydrodechlorination of the starting material ($1a_{Dechlorine}$) and product ($3a_{Dechlorine}$) which proved to be difficult to separate from the product. This deleterious pathway could be avoided by reducing the reaction time.

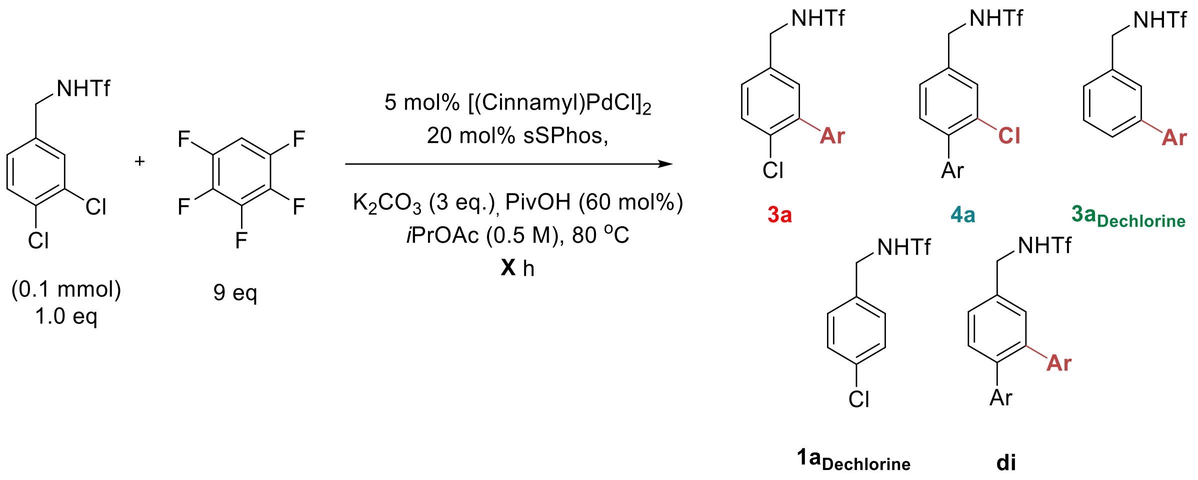

Entry	Time (h)	% 3a	% 4a	% $3a_{Dechlorine}$	% $1a_{Dechlorine}$	di	% SM	% conv
1	2	57	6	0	3	0	34	66
2	3	69	6	0	3	0	22	78
3	4	74	6	0	3	0	16	84
4	5	80	6	0	3	0	12	88
5	6	81	6	0	3	0	9	90
6	7	84	6	0	3	0	7	93
7	22	85	7	2	3	0	3	97

Dimethoxyethane (0.033 mmol) used as internal standard.

4. Ligand Synthesis

sSPhos - sodium 2'-(dicyclohexylphosphaneyl)-2,6-diisopropyl-[1,1'-biphenyl]-4-sulfonate

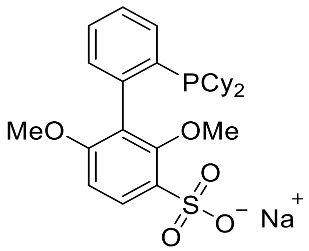

Prepared according to our previous publication.[1] This ligand is also commercially available from Strem (15-1135) and Sigma Aldrich (677280).

sSPhos$_{(NBu4)}$ - tetrabutylammonium 2'-(dicyclohexylphosphaneyl)-2,6-diisopropyl-[1,1'-biphenyl]-4-sulfonate (see Table S5)

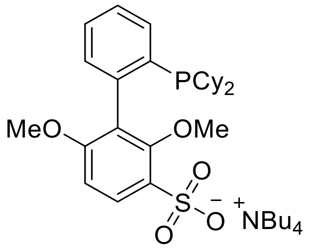

Prepared according in accordance with our previous publication.[1]

5. Synthesis of starting materials

(1a) *N*-(3,4-dichlorobenzyl)-1,1,1-trifluoromethanesulfonamide

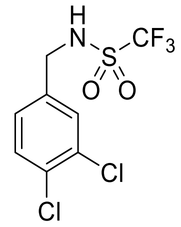

Prepared in accordance with our previous publication.[1]

Triisopropyl((2,3,5,6-tetrafluorophenyl)ethynyl)silane

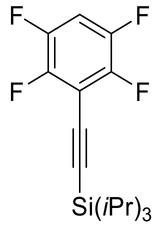

3-Bromo-1,2,4,5-tetrafluorobenzene (0.81 ml, 6.58 mmol), PPh_3 (346.2 mg, 1.32 mmol), CuI (251.3 mg, 1.32 mmol), $Pd(PPh_3)Cl_2$ (463 mg, 0.66 mmol) were dissolved in a mixture of triethylamine (15 ml), and toluene (15 ml). The mixture was heated to 80 °C under argon at which point TIPS-acetylene (2.96 mmol, 13.2 mmol) was added dropwise and allowed to stir at 80 °C for 16 h. Water (30 ml) was added to the cooled mixture and the organic phase was washed with sat. $NH_4Cl_{(aq)}$ (30 ml), 3 M $HCl_{(aq)}$ (30 ml), 10 % $Na_2CO_{3(aq)}$ (30 ml) and dried over $MgSO_4$. The solvents were removed under reduced pressure and the residue was purified *via* column chromatography (hexanes) to give the title compound as a colourless oil (881 mg, 2.67 mmol, 41%).

^{1}H NMR (400 MHz, $CDCl_3$) δ_H 7.02 (tt, *J* = 9.7, 7.3 Hz, 1H), 1.16-1.12 (m, 21H); **^{13}C NMR** (101 MHz, $CDCl_3$) δ_C 147.3 (dddd, *J* = 252.6, 14.6, 4.0, 3.1 Hz), 145.6 (dddd, *J* = 247.6, 13.7, 10.6, 4.3 Hz), 106.4 (t, *J* = 3.9 Hz), 106.1 (t, *J* = 22.8 Hz), 105.5 (tt, *J* = 17.8, 2.7 Hz), 90.50 (t, *J* = 4.1 Hz), 18.48, 11.06; **^{19}F NMR** (376 MHz, $CDCl_3$) δ_F -136.5 - -136.3 (m, 2F), -139.3 - -139.5 (m, 2F).

Data in accordance with that in the literature.[2]

Methyl 2,3,5,6-tetrafluorobenzoate

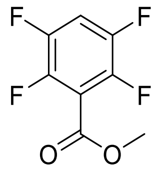

To 2,3,5,6-tetrafluorobenzoic acid (970.5 mg, 5 mmol) in PhMe:MeOH (3:2, 50 mL) under argon, (trimethylsilyl)diazomethane (2M in ether, 5.0 mL, 10 mmol) was added dropwise. The solution was stirred at room temperature for 30 minutes, then AcOH (3.0 mL) added to quench excess (Trimethylsilyl)diazomethane. The solvent was removed under reduced pressure, and the residue was purified *via* column chromatography (CH_2Cl_2) to give the title compound as a colourless oil (609.7 mg, 2.93 mmol, 59%.

^{1}**H NMR** (600 MHz, $CDCl_3$) δ_H 7.01 (tt, *J* = 10.0, 7.2 Hz, 1H), 2.42 (s, 3H); ^{13}**C NMR** (151 MHz, $CDCl_3$) δ_C 166.5, 146.0 (dtd, *J* = 248.6, 12.0, 3.8, Hz), 140.58 (ddd, *J* = 250.3, 15.2, 4.9, 2.2 Hz), 129.6 (tt, *J* = 14.6, 4.0 Hz), 103.1 (t, *J* = 22.8 Hz), 19.8; ^{19}**F NMR** (376 MHz, $CDCl_3$) δ_F -139.2 - -139.3 (m, 2F), -153.2 - -153.3 (m, 2F).

Data in accordance with that in the literature.[3]

N-(2,3,5,6-tetrafluorophenyl)acetamide

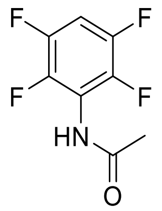

A solution of 2,3,5,6-tetrafluoroaniline (0.66 ml, 6.05 mmol) in ahydrous pyridine (0.54 mmol, 6.66 mmol) was treated with acetic anhydrice (0.63 ml, 6.05 mmol) and heated to reflux for 2 h. After cooling the solution was poured into ice water (10 ml). The resulting precipitate was filtered, dissolved in ethyl acetate, dried over $MgSO_4$, filtered and concentrated. The solid material was washed with heptane (20 ml) and dried to give the title compound as a white crystalline solid (821.7 mg, 4.0 mmol, 66%).

^{1}**H NMR** (400 MHz, $CDCl_3$) δ_H 7.21 (br s, 1H), 7.02 (tt, *J* = 9.7, 7.1 Hz, 1H), 2.26 (s, 3H); ^{13}**C NMR** (101 MHz, $CDCl_3$) δ_C 168.4, 146.0 (dddd, *J* = 247.7 Hz, 13.0, 11.4, 4.1), 142.19 (dm, *J* = 249.7 Hz), 116.8 (t, *J* = 15.5 Hz), 104.0 (t, *J* = 22.7 Hz), 22.9; ^{19}**F NMR** (376 MHz, $CDCl_3$) δ_F -139.1 (s, 2F), -145.47 (q, *J* = 10.2 Hz, 2F).

Data in accordance with that in the literature.[4]

2,2',3,3',4,5,5',6,6'-Nonafluoro-1,1'-biphenyl

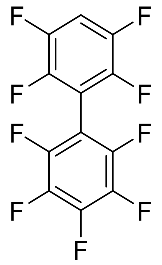

An oven dried, 25 ml two neck round bottom flask fitted with a reflux condenser and a rubber septum was charged with magnesium turnings (364.5 mg, 15 mmol) under argon. THF (15 ml) was added followed by the dropwise addition of bromopentafluorobenzene (1.90 ml, 15 mmol) maintaining a gentle reflux. The mixture was stirred at room temperature for 1 h after which freshly prepared anhydrous $Cu^{I}Br$[5] (4.31 mg, 30 mmol) was added. The brown suspension was stirred for a further 1 h at room temperature, dioxane (7.5 ml) was then added and the resulting grey suspension was stirred for 1 h at room temperature. To the suspension was added a solution of 3-bromo-1,2,4,5-tetrafluorobenzene (0.93 ml, 7.5 mmol) in toluene (7.5 ml) the resulting mixture was stirred at 80 °C for 4 days and then filtered through Celite. The solvents were removed under reduced pressure and the residue was dissolved in toluene (20 ml) and washed with 3 M HCl (20 ml), and brine (20 ml). The organics were dried over $MgSO_4$, filtered and then removed under reduced pressure. The residue was

purified *via* column chromatography (hexanes) to give the title compound as an amorphous white solid (1.37 g, 4.34 mmol, 58%).

^{1}**H NMR** (400 MHz, $CDCl_3$) δ_H 7.27 (tt, *J* = 9.4, 7.4 Hz, 1H); ^{13}**C NMR** (101 MHz, $CDCl_3$) δ_C 145.9 (dddd, *J* = 249.7, 12.7, 10.3, 5.1 Hz), 144.4 (dm, *J* = 252.6 Hz), 144.0 (dt, *J* = 251.1, 4.1 Hz), 142.5 (dtt, *J* = 257.0, 13.3, 5.1 Hz), 137.8 (dm, *J* = 250.9 Hz), 108.1 (t, *J* = 22.5 Hz), 106.97 (t, *J* = 18.4 Hz), 102.6-102.2 (m); ^{19}**F NMR** (376 MHz, $CDCl_3$) δ_F -137.5 - -137.8 (m, 2F), -138.3 - -138.4 (m, 4F), -150.42 (tt, *J* = 20.9, 2.8 Hz, 1F), -160.7 - -160.8 (m, 2F).

Data in accordance with that in the literature.[6]

N-(3,4-dichlorophenethyl)-1,1,1-trifluoromethanesulfonamide

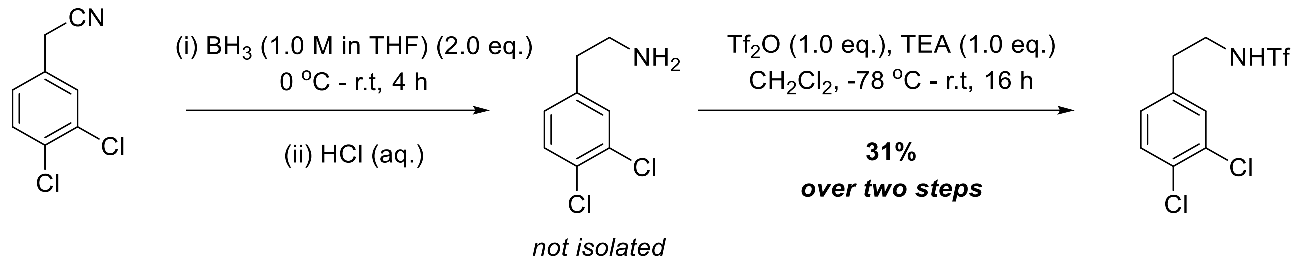

Prepared according to our previous publication in accordance with our previous publication.[1]

N-(3-(3,4-dichlorophenyl)propyl)-1,1,1-trifluoromethanesulfonamide

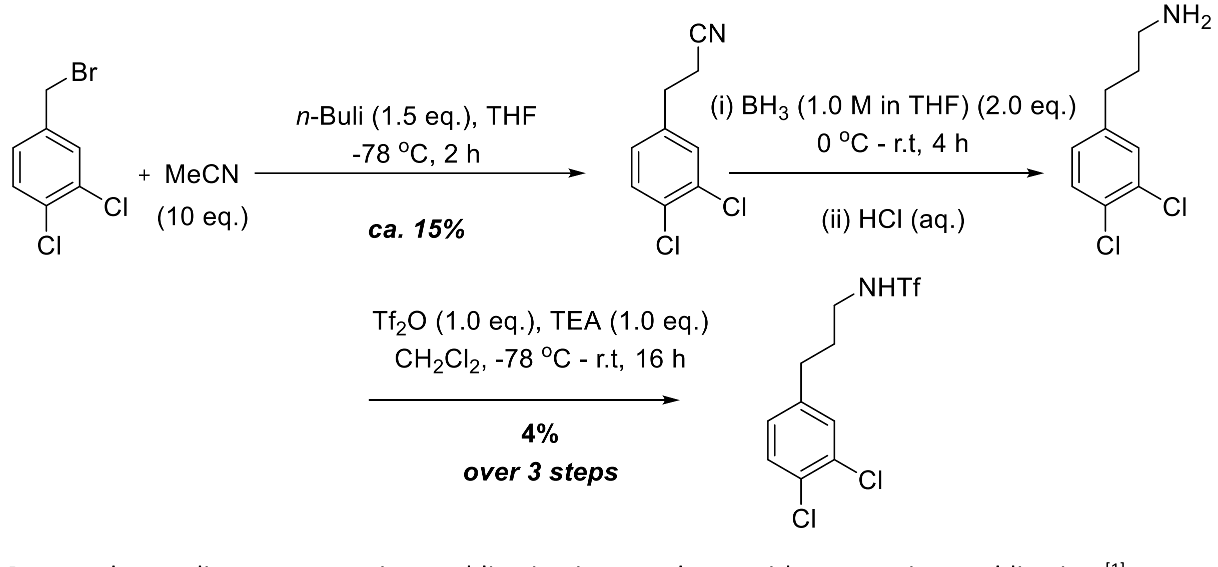

Prepared according to our previous publication in accordance with our previous publication.[1]

N-(1-(3,4-dichlorophenyl)ethyl)-1,1,1-trifluoromethanesulfonamide

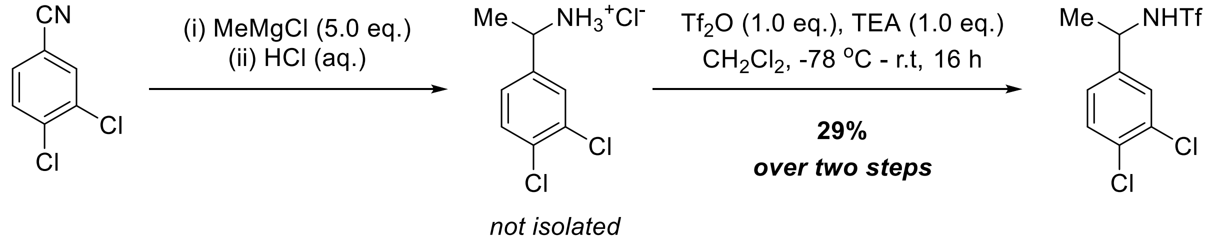

To a solution of 3,4-diclohrobenzonitrile (2.50 g, 14.3 mmol) in Et2O (9.0 mL) was added MeMgBr (3.0 M in Et2O, 25.5 mL, 76.5 mmol). After being stirred at 40 °C for 24 h, the reaction mixture was cooled to 0 °C, quenched with anhydrous MeOH (5.0 mL), diluted with Et2O, filtered through a plug of celite and concentrated invacuo. The crude imine was dissolved in anhydrous MeOH (50 mL) and NaBH4 (1.13 g, 30 mmol) was added in two portions. After being stirred for 3 h, the reaction mixture was quenched with saturated aqueous $NaHCO_3$ and water (50 ml) was added which was extracted with EtOAc (3 x 50 mL), washed with brine, dried with MgSO4 and concentrated under reduced pressure. The crude material was taken up in Et_2O (30 ml) and HCl (2.0 M in Et_2O, 14.3 ml, 28.6 mmol) was added dropwise as 0°C. The resulting precipitate was collected under filtration and washed with portions of ice cold Et_2O (3 x 10 ml) to give the crude ammonium hydrochloride salt. The crude ammonium salt was dissolved in CH_2Cl_2 (200 ml) and triethylamine (2.91 ml, 20.9 mmol) was added and cooled to -78 °C. Trifluoromethanesulfonic anhydride (1.89 ml, 10.9 mmol) was then added dropwise and left to stir at room temperature overnight. HCl (3 M, 50 ml) was added and the organic layer was kept, dried with $MgSO_4$, and concentrated under reduced pressure. The resulting residue was purified *via* column chromatography (eluting with CH_2Cl_2:Hexanes (50:50)) to give the title compound as an yellow oil (1.055 g, 3.29 mmol, 23% *over two steps).*

^{1}H NMR (400 MHz, $CDCl_3$) δ_H 7.48 (d, *J* = 8.3 Hz, 1H), 7.43 (d, *J* = 2.1 Hz, 1H), 7.18 (dd, *J* = 8.3, 2.1 Hz, 1H), 5.54 (br d, *J* = 7.9 Hz, 1H), 4.76 (quin, *J* = 7.3 Hz, 1H), 1.62 (d, *J* = 7.0 Hz, 3H); **^{13}C NMR** (101 MHz, $CDCl_3$) δ_C 141.3, 133.2, 132.5, 131.1, 128.0, 125.3, 119.4 (q, *J* = 320.8 Hz), 54.3, 23.3; **^{19}F NMR** (376 MHz, $CDCl_3$) δ_F -77.5 (s, 3F). **HRMS m/z:** $[M-H]^-$ calculated for $[C_{14}H_9Cl_2F_3NO_2S]^-$: 381.9683, found 381.9679.

N-((3,4-dichlorophenyl)(phenyl)methyl)-1,1,1-trifluoromethanesulfonamide

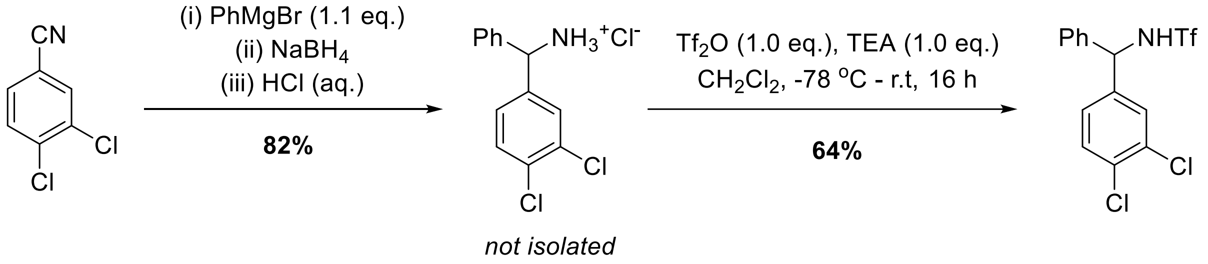

To a solution of bromobenzene (2.13 ml, 20 mmol) in THF (7 ml) was added Magnesium turnings (500 mg, 20 mmol), the resulting mixture was heated to reflux for 30 minutes until Grignard formation. The solution was then cooled to room temperature when 3,4-dichlorobenzonitrile (3.13 g, 18.2 mmol) in THF (8 ml) was added dropwise, the reaction mixture was then heated to reflux for 5 h. MeOH (15 ml) was then added dropwise at 0 °C followed by the portionwise addition of sodium borohydride (1.50 g, *ca.* 40 mmol). The reduction was left stirring at room temperature for 72 h followed by an additional 1 h at 70 °C. The solvents where removed and the reside was poured over HCl (3 M, 30 ml) and the resulting precipitate was collected under filtration and washed with Et_2O to give the hydrochloride salt (3.246 g, 11.2 mmol, 82%). The crude ammonium salt (1.44 g, 5 mmol) was dissolved in CH_2Cl_2 (50 ml) and triethylamine (1.4 ml, 10 mmol) and then cooled to -78 °C. Trifluoromthanesulfonic anhydride (0.89 ml, 5.3 mmol) was then added dropwise and left to stir at room temperature overnight. HCl (3 M, 50 ml) was added and the organic layer was kept, dried with $MgSO_4$, and concentrated under reduced pressure. The resulting residue was purified *via* column chromatography (eluting with EtOAc:Hexanes (20:80)) to give the title compound as pale yellow solid (1.224 g, 3.19 mmol, 64%).

^{1}H NMR (600 MHz, $CDCl_3$) δ_H 7.48 (d, *J* = 8.3 Hz, 1H), 7.44-7.37 (m, 3H), 7.36 (d, *J* = 2.1 Hz, 1H), 7.23-7.20 (m, 2H), 7.14 (dd, *J* = 3.5 Hz, 1H), 5.83 (s, 1H), 5.45 (br s, 1H); **^{13}C NMR** (151 MHz, $CDCl_3$) δ_C 139.8,

138.4, 133.3, 132.7, 130.9, 129.4, 129.1, 129.0, 127.2, 126.4, 119.4 (q, *J* = 321.2 Hz), 61.4; **^{19}F NMR** (376 MHz, $CDCl_3$) δ_F -77.0 (s, 3F). **HRMS m/z:** $[M-H]^-$ calculated for $[C_9H_7Cl_2F_3NO_2S]^-$: 319.9532, found 319.9533.

(1b) Sodium (3,4-dichlorophenyl)methanesulfonate

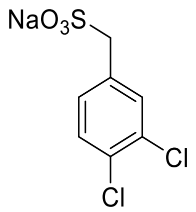

Prepared in accordance with our previous publication.[1]

(1c) Potassium (3,4-dichlorobenzyl)sulfamate

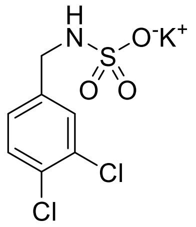

A solution of 3,4-dichlorobenzyl amine (0.80 ml, 6.0 mmol) in CH_2Cl_2 (25 ml) was cooled to 0 °C, under an argon atmosphere. Triethylamine (8.5 ml) was added followed by dropwise addition of chlorosulfonic acid (0.4 ml, 0.6 mmol, 1 eq.) by pipette. The reaction mixture was then allowed to warm to room temperature and stirred for 1 hour. KOH (1.01 g, 18 mmol, 3 eq.) in 50 ml was added and the layers separated, the aqueous layer was kept and washed with CH_2Cl_2 (30 ml) and Et_2O (30 ml). The water was the removed under reduced pressure to give the title compound as a gummy solid in 63% purity. The solid was then taken up in the minimum amount of boiling water and allowed to cool to 0 °C. The resulting precipitate was collected via filtration and the filter cake was washed with ice cold water (30 ml), ice cold ethanol (10 ml), and then ice cold ether (10 ml) to give the title compound as an amorphous white solid (540 mg, 1.89 mmol, 31%).

^{1}H NMR (400 MHz, DMSO) δ_H 7.61 (s, 1H), 7.51 (d, *J* = 8.2 Hz, 1H), 7.31 (dd, *J* = 8.2, 1.7 Hz, 1H), 5.04 (t, *J* = 7.0 Hz, 1H), 3.92 (d, *J* = 7.1 Hz, 2H); ^{13}C NMR (100 MHz, DMSO) δ_C 143.2, 130.9, 130.3, 130.0, 128.9, 128.4, 46.5; **HRMS m/z:** $[M-H]^-$ calculated for $[C_7H_6Cl_2NO_3S]^-$ 253.9451, found 253.9452.

(1d) Ethyl hydrogen (3,4-dichlorobenzyl)phosphonate

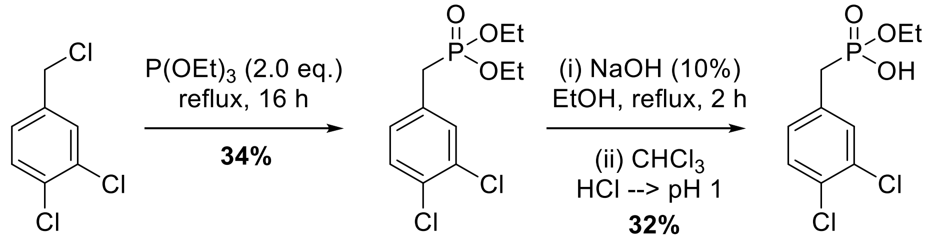

3,4-dichlorobenzyl chloride (2.77 ml, 20 mmol) and triethylphosphite (6.9 ml, 40 mmol) were refluxed under neat conditions for 16 h. The volatiles removed with the aid of a trolley pump (1.7 torr, 100-150 °C) (*some product was collected in the distillate)* and the residue was purified *via* column chromatography (EtOAc:Petroleum ether (70:100 -> 100:0)) to give the diethylphosphonate (2.02 g,

6.82 mmol, 34%). Diethyl (3,4-dichlorobenzyl)phosphonate (2.02 g, 6.82 mmol), EtOH (40 ml) and 10% $NaOH_{(aq)}$ (20 ml) were heated to reflux for two hours. After cooling to room temperature $CHCl_3$ (40 ml) was added. The biphasic system was stirred and the pH was adjusted by adding 3M HCl until pH = 1. The layers were then separated, and the aqueous phase was extracted with EtOAc (2 x 70 ml). The combined organics were then dried over $MgSO_4$, filtered, and concentrated under reduced pressure to give the title compound as a cream solid (574 mg, 2.15 mmol, 32%).

^{1}**H NMR** (400 MHz, $CDCl_3$) δ_H 10.77 (br s, 1H), 7.37-7.34 (m, 2H), 7.09 (d, *J* = 8.8 Hz, 1H), 3.91 (quin, *J* = 7.0 Hz, 2H), 3.00 (d, *J* = 21.7 Hz, 2H), 1.23 (t, *J* = 7.0 Hz, 3H); ^{13}**C NMR** (101 MHz, $CDCl_3$) δ_C 132.4 (d, *J* = 3.5 Hz), 131.8, 131.7 (d, *J* = 6.4 Hz), 131.2 (d, *J* = 4.4 Hz), 130.4 (d, *J* = 3.0 Hz), 129.3 (d, *J* = 6.4 Hz), 62.0 (d, *J* = 7.1 Hz), 32.9 (d, *J* = 141.3 Hz), 16.2 (d, *J* = 6.4 Hz); ^{31}**P NMR** (162 MHz, $CDCl_3$) δ_P 27.3 (s, 1P); **HRMS m/z:** $[M-H]^-$ calculated for $[C_9H_{10}Cl_2O_3P]^-$ 266.9750, found 266.9750.

(2-carboxyethyl)triphenylphosphonium chloride

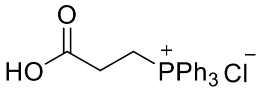

3-Chloropropanoic acid (5.0 g, 46.1 mmol) and triphenylphosphine (12.7 g, 48.5 mmol) were dissolved in acetonitrile (35 ml) and heated to reflux for 5 h. Once cooled to room temperature, the reaction mixture was allowed to stir at room temperature overnight at which point a precipitate formed. The minimum amount of CH_2Cl_2 (*ca. 5.0 ml*) was added until all the precipitate dissolved, Et_2O was then added to induce precipitation which was collected under filtration and washed with ice cold ether (20 ml) to give the title compound as an amorphous white solid (6.0 g, 14.5 mmol, 32%).

^{1}**H NMR** (400 MHz, MeOD) δ_H 7.93-7.88 (m, 3H), 7.86-7.74 (m, 12H), 3.76-3.69 (m, 2H), 2.78-2.71 (m, 2H); ^{13}**C NMR** (101 MHz, MeOD) δ_C 171.8 (d, *J* = 15.4 Hz), 135.2 (d, *J* = 3.0 Hz), 133.5 (d, *J* = 10.1 Hz), 130.2 (d, *J* = 12.7 Hz), 118.1 (d, *J* = 87.0 Hz), 26.3 (d, *J* = 2.7 Hz), 17.5 (d, *J* = 55.5 Hz). ^{31}**P NMR** (162 MHz, MeOD) δ_P 24.30.

Data in accordance with that in the literature.[7]

methyl (E)-4-(3,4-dichlorophenyl)but-3-enoate

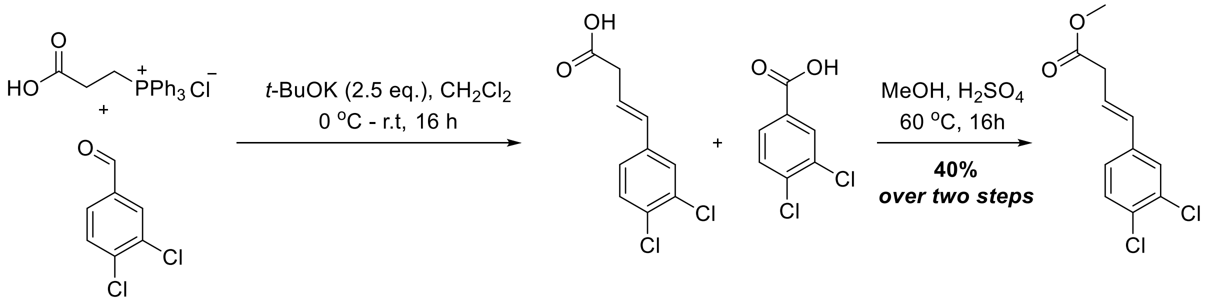

To (2-carboxyethyl)triphenylphosphonium (2.0 g, 4.83 mmol) and 3,4-dichlorobenzaldehyde (*The batch of aldehyde was contaminated with the benzoic acid, a simple acid-base extraction afforded to clean aldehyde)* (704.4 mg, 4.03 mmol) in CH_2Cl_2 (5 ml) at 0 °C was added potassium tert-butoxide (1.13 g, 10.1 mmol) portion wise. The mixture was warmed to room temperature and allowed to stir for overnight. Water (5.0 ml) was then added and washed with CH_2Cl_2 (3 x 10 ml), the aqueous was then acidified to pH 1 by addition of HCl (3 M). The aqueous was then extracted with Et_2O (3 x 10 ml) and the combined organics were dried over $MgSO_4$, and concentrated under reduced pressure to give a mixture of the desired acid and 3,4-dichlorobenzoic acid as impurity. The mixture was taken up in

anhydrous MeOH (5.0 ml) and sulfuric acid (0.05 ml) was added then stirred at 60 °C for 16 h. After cooling to room temperature, EtOAc (20 ml) was added and washed with saturated $NaHCO_3$ (20 ml) and brine (20 ml). The organic layer was then dried over $MgSO_4$, concentrated under reduced pressure and purified *via* column chromatography (eluting with EtOAc:hexanes (5:95)) to give the title compound as a yellow oil.

^{1}H NMR (400 MHz, $CDCl_3$) δ_H 7.43 (d, *J* = 2.0 Hz, 1H), 7.35 (d, *J* = 8.3 Hz, 1H), 7.17 (dd, *J* = 8.3, 2.0 Hz, 1H), 6.38 (d, *J* = 16.0 Hz, 1H), 6.30 (dt, *J* = 15.7, 6.6 Hz, 1H), 3.73 (s, 3H), 3.25 (d, *J* = 6.6 Hz, 2H). **^{13}C NMR** (101 MHz, $CDCl_3$) δ_C 171.5, 136.9, 132.6, 131.2, 131.1, 130.4, 123.0, 125.5, 123.9, 52.0, 38.0.

Data in accordance with that in the literature.[8]

(E)-4-(3,4-dichlorophenyl)but-3-enoic acid

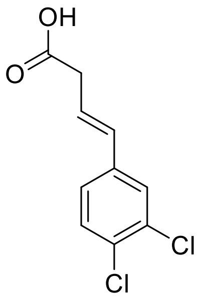

Methyl (E)-4-(3,4-dichlorophenyl)but-3-enoate (381.9 mg, 1.57 mmol) and LiOH (97.2 mg, 4.06 mmol) were dissolved in THF:H_2O:MeOH (4:1:1, 6.0 ml) and heated to 70 °C and monitored by TLC. After 2 hour the solvents were removed under reduced pressure and the residue was taken up in CH_2Cl_2:H_2O (1:1, 20 ml) and the aqueous was acidified to pH1 by addition of HCl (3 M). The layers were separated and the aqueous was extracted with CH_2Cl_2 (2 x 10 ml) and the combined organics were dried over $MgSO_4$ and concentrated under reduced. The residue was purified *via* column chromatography (eluting with MeOH:CH_2Cl_2 (5:95)) to give the title compound as a light yellow solid (254.8 mg, 1.11 mmol, 71%).

^{1}H NMR (400 MHz, $CDCl_3$) δ_H 10.58 (br s, 1H), 7.46 (d, *J* = 1.8 Hz, 1H), 7.39 (d, *J* = 8.3 Hz, 1H), 7.20 (dd, *J* = 8.3, 1.8 Hz, 1H), 6.43 (d, *J* = 15.9 Hz, 1H), 6.30 (dt, *J* = 16.0, 6.8 Hz, 1H), 3.33 (d, *J* = 6.8 Hz, 2H). **^{13}C NMR** (101 MHz, $CDCl_3$) δ_C 177.7, 136.7, 132.7, 131.7, 131.4, 130.5, 128.1, 125.5, 123.0, 37.9.

Data in accordance with that in the literature.[9]

4-(3,4-dichlorophenyl)butanoic acid

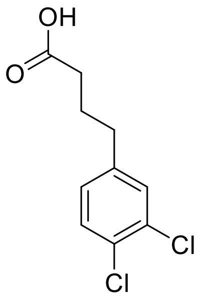

(E)-4-(3,4-dichlorophenyl)but-3-enoic acid (50 mg, 0.22 mmol), palladium on carbon (10% wt, 2.13 mg, 0.02 mmol) and THF (0.66 ml) were added to a 4 mL 15x45 mm crimp top vial containing a stirrer bar. The vial was evacuated and back filled with H_2 5 times and left stirring at room temperature. LCMS suggested full conversion after 1 h. The reaction mixture was then filtered through a plug of Celite

eluting with Et_2O and solvents removed under reduced pressure to give the title compound as a light yellow solid (46.2 mg, 0.199 mmol, 91%).

^{1}H NMR (400 MHz, $CDCl_3$) δ_H 10.47 (br s, 1H), 7.37 (d, *J* = 8.2 Hz, 1H), 7.30 (d, *J* = 1.8 Hz, 1H), 7.04 (dd, *J* = 8.2, 1.8 Hz, 1H), 2.66 (t, *J* = 7.7 Hz, 2H), 2.40 (t, *J* = 7.3 Hz, 2H), 1.98 (quintet, *J* = 7.5 Hz, 2H); **^{13}C NMR** (101 MHz, $CDCl_3$) δ_C 179.6, 141.4, 132.3, 130.4, 130.4, 130.08, 128.0, 34.1, 33.1, 25.8.

Data in accordance with that in the literature.[10]

4,5-Dimethylthiazole 3-oxide

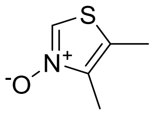

2-UNSUBSTITUTED THIAZOLE N-OXIDES ARE SENSITIVE TO HEAT. AS SUCH, THIS COMPOUND WAS STORED IN A FREEZER UNDER AN ATMOSPHERE OF ARGON AND EVAPORATION UNDER REDUCED PRESSURE WAS PERFORMED WITH ONLY MILD HEATING (28 – 30 ^{O}C)

To 4,5-dimethylthiazole (1.6 ml, 15 mmol) in DCE (30 ml) was added *m*CPBA (77%, 4.04 g, 18 mmol). The reaction was left to stir at room temperature for 16 h. The reaction was cooled to 0 °C and the white solid was filtered off. The filtercake was washed with cold CH_2Cl_2 and the filtrate was collected under reduced pressure at 28 °C. The resulting solid was then purified *via* column chromatography (CH_2Cl_2:MeOH (95:5 -> 90:10)) to give the title compound as an off-white amorphous solid. (1.454 g, 11.3 mmol, 75%).

^{1}H NMR (400 MHz, $CDCl_3$) δ_H 8.05 (s, 1H), 2.37 (d, *J* = 0.7 Hz, 1H), 2.27 (d, *J* = 0.4 Hz, 1H); **^{13}C NMR** (100 MHz, $CDCl_3$) δ_C 141.5, 126.7, 125.6, 13.4, 10.6.

Data in accordance with that in the literature.[11]

Pyrazine 1-oxide

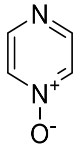

Pyrazine (2.402 g, 30 mmol) and *m*CPBA (77%, 6.901 g, 30 mmol) were stirred in CH_2Cl_2 (150 ml) for 16 h. PPh_3 (3.93 g, 15 mmol) was then added to reduce any unreacted peracid and the mixture was stirred for an additional 3 h. The volatiles were evaporated under reduced pressure and the residue was purified *via* column chromatography (EtOAc:MeOH (80:20)) to give the title compound as a crystalline white solid.

^{1}H NMR (400 MHz, $CDCl_3$) δ_H 8.48 (dd, *J* = 3.5, 1.0 Hz, 2H), 8.11 (dd, *J* = 3.4, 1.4 Hz, 2H); **^{13}C NMR** (100 MHz, $CDCl_3$) δ_C 147.9, 134.2.

Data in accordance with that in the literature.[12]

N-(4-chlorobenzyl)-1,1,1-trifluoromethanesulfonamide

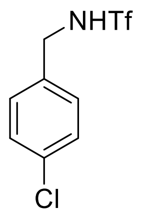

To a cooled (-78 °C) solution of 4-chlorobenzylamine (1.22 ml, 10.0 mmol) and triethylamine (1.52 ml, 11.0 mmol) in CH_2Cl_2 (150 ml) was added dropwise trifluoromethanesulfonic anhydride (1.84 ml, 11.0 mmol). The reaction mixture was warmed to room temperature and stirred for a further 16 h before being quenched with water (100 ml). The organic layer was separated and the aqueous layer was extracted with CH_2Cl_2 (100 ml x 3). The combined organics were dried over $MgSO_4$ and concentrated under reduced pressure. Column chromatography (CH_2Cl_2:Hexanes (50:50)) gave the title compound as a clear oil that solidified on standing to a crystalline yellow solid (509.3 g, 1.87 mmol, 19%).

^{1}H NMR (600 MHz, $CDCl_3$) δ_H 7.39 (d, *J* = 8.5 Hz, 2H), 7.29 (d, *J* = 8.5 Hz, 2H), 5.07 (br s, 1H), 4.44 (d, *J* = 6.0 Hz, 2H). **^{13}C NMR** (101 MHz, $CDCl_3$) δ_C 134.6, 133.8, 129.2, 129.2, 119.6 (q, *J* = 321.0 Hz), 47.4. **^{19}F NMR** (376 MHz, $CDCl_3$) δ_F -77.2 (s, 3F).

Data in accordance with that in the literature.[13]

Potassium 2,4-dimethoxybenzenesulfonate

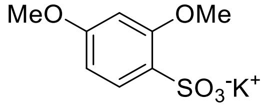

To 1,3-dimethoxybenzene (8.50 g, 61.5 mmol) was added sulfuric acid (5 ml) at 0 °C. This mixture was allowed to warm to room temperature and stir for a further 3 h at which point the mixture set solid. This was then poured carefully over a saturated solution of potassium carbonate (100 ml) and the resulting precipitate was collected under filtration. The solid was then dried under reduced pressure and any remaining water was removed by the sequential addition then removal of ethanol (15 ml) then toluene (15 ml). Dichloromethane (2 x 30 ml) was then added and stirred briefly before being decanted to remove any remaining starting material, the solid was finally dried *in vacuo* to give the title compound as a white amorphous solid (14.75 g, 57.5 mmol, 94%).

^{1}H NMR (600 MHz, D_2O) δ_H 7.63 (q, *J* = 3.1 Hz, 1H), 6.60 (d, *J* = 1.9 Hz, 1H), 6.53 (m, *J* = 2.2 Hz, 1H), 3.82 (s, 1H), 3.78 (s, 1H); **^{13}C NMR** (101 MHz, D_2O) δ_C 163.17 (s, 1C), 157.71 (s, 1C), 129.61 (s, 1C), 122.78 (s, 1C), 104.54 (s, 1C), 99.25 (s, 1C), 55.73 (s, 1C), 55.64 (s, 1C).

Data in accordance with that in the literature.[14]

6. Fluorinated arene scope

^{1}H-COSY, DEPT-135, HMQC, HMBC and NOSEY were used to facilitate structural determination of the major regioisomers. The diagnostic relationships used to confirm the obtained regioisomer are shown below:

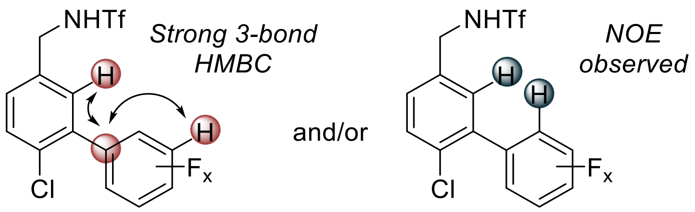

The ratio of *meta*:*para* coupled products in the ^{1}H-NMR of the crude reactions was assigned by integration of the benzylic peaks at ~4.5 ppm which typically possessed a characteristic shift depending on the regioisomer. Due to the very small amounts of para-coupled product obtained due to the high regioselectivity of the reactions it was not feasible to characterise these independently but this has been done for a representative example (**3a**/**4a**).

General procedure for Direct arylation (GP1)

N-(3,4-dichlorobenzyl)-1,1,1-trifluoromethanesulfonamide (61.4 mg, 0.20 mmol, 1.0 eq.), sSPhos$_{(NBu4)}$ (29.2 mg, 0.04 mmol, 20 mol%), [(Cinnamyl)PdCl]$_2$ (5.2 mg, 0.01 mmol, 5 mol%), K_2CO_3 (82.9 mg, 0.6 mmol, 3.0 eq.) and - *if solid* - the fluoroarene (9.0 eq.) were added to a 4 mL 15x45 mm crimp top vial containing a stirrer bar. The vial was evacuated and back filled with argon 3 times, at which point *i*PrOAc (0.2 ml) and - *if liquid* - the fluoroarene (9.0 eq.) were added and the top wrapped with parafilm. The reaction mixture was stirred for 8 *or* 16 h at 80 °C in a heating block and the solvent was removed under a stream of nitrogen and $CHCl_3$ (2.0 ml) and 3M HCl (2.0 ml) were added. The organic layers were separated and the aqueous was extracted with $CHCl_3$ (3 x 2.0 ml). The combined organics were dried over $MgSO_4$, concentrated under reduced pressure and dimethoxyethane (3.0 mg, 0.0333 mmol) in $CDCl_3$ (0.7 ml) was added to assess the *meta*:*para* coupled ratio in the crude ^{1}H NMR. The residue was then purified *via* column chromatography.

(3a) N-((6-chloro-2',3',4',5',6'-pentafluoro-[1,1'-biphenyl]-3-yl)methyl)-1,1,1-trifluoromethanesulfonamide

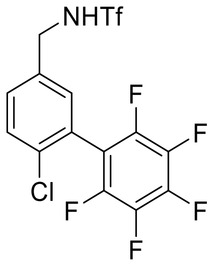

GP1 was followed with an 8 h reaction time and pentafluorobenzene (0.20 ml, 1.8 mmol). Analysis of the crude ^{1}H NMR showed a meta:para ratio of 15:1. The residue was purified *via* flash chromatography (eluting with CH_2Cl_2:Hexanes (50:50) *then* the mixture was purified again eluting with EtOAc:Hexanes (10:90)) to give the title compound (in a 16:1 Cl_m:Cl_p ratio) as an amorphous yellow solid (61.8 mg, 0.141 mmol, 71%).

^{1}**H NMR** (400 MHz, $CDCl_3$) δ_H 7.58 (d, *J* = 8.3 Hz, 1H), 7.42 (dd, *J* = 8.3, 2.2 Hz, 1H), 7.29 (d, *J* = 2.3 Hz, 1H), 5.39 (br s, 1H), 4.48 (d, *J* = 5.5 Hz, 2H); ^{13}**C NMR** (101 MHz, $CDCl_3$) δ_C 144.1 (dm, *J* = 249.9 Hz), 141.4 (dm, *J* = 257.9 Hz), 137.7 (dm, *J* = 254.9 Hz), 134.9, 134.6, 131.3, 130.7, 130.4, 126.6 (d, *J* = 1.5 Hz), 119.7 (q, *J* = 320.9 Hz), 112.7 (td, *J* = 19.0, 4.0 Hz), 47.2; ^{19}**F NMR** (376 MHz, $CDCl_3$) δ_F -77.1 (s, 3F), -139.7 (q, *J* = 10.2 Hz, 2F), -153.2 (t, *J* = 20.5 Hz, 1F), -161.5 - -161.6 (m, 2F). **HRMS m/z:** $[M-H]^-$ calculated for $[C_{14}H_5ClF_8NO_2S]^-$ 437.9616, found 437.9616.

(4a) N-((2-chloro-2',3',4',5',6'-pentafluoro-[1,1'-biphenyl]-4-yl)methyl)-1,1,1-trifluoromethanesulfonamide

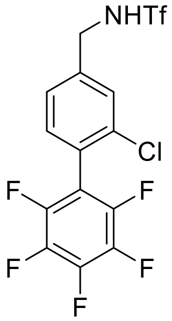

GP1 was followed with an 6 h reaction time, pentafluorobenzene (0.20 ml, 1.8 mmol) and SPhos (20.4 mg, 0.04 mmol) instead of $sSPhos_{(NBu4)}$. Analysis of the crude ^{1}H NMR with reference to DME (0.033 mmol) as an internal standard showed a NMR yield of 47:39:12 (Cl_m:Cl_p:SM). The residue was purified *via* flash chromatography (eluting with CH_2Cl_2:Hexanes (50:50) *then* the mixture was purified again eluting withEtOAc:Hexanes (10:90)) to give the title compound in a 1.3:1.0:0.3 Cl_m:Cl_p:SM as a yellow oil (*isolated mass 49.3 mg, corrected mass of meta+para* 45.4 mg, 0.103 mmol 52%).

By deduction of the isolated meta isomer and starting material the para isomer can be described as follows: ^{1}**H NMR** (400 MHz, $CDCl_3$) δ_H 7.54 (d, *J* = 1.5 Hz, 1H), 7.38 (dd, *J* = 8.1, 1.5 Hz, 1H), 7.35 (d, *J* = 8.1 Hz, 1H), 5.35 (br s, 1H), 4.51 (s, 2H); ^{13}**C NMR** (101 MHz, $CDCl_3$) δ_C 144.1 (dm, *J* = 252.3 Hz), 141.4 (dm, *J* = 256.0 Hz), 139.0 (dm, *J* = 252.9 Hz), 138.7, 135.2, 132.7, 129.2, 126.24 (br s), 126.2, 119.6 (q, *J* = 319.8 Hz), 112.68 (td, *J* = 18.8, 3.9 Hz), 47.2; ^{19}**F NMR** (376 MHz, $CDCl_3$) δ_F -77.1 (s, 3F), -139.6 - -139.8 (m, 2F), -153.3 (t, *J* = 20.5 Hz, 1F), -161.6 - -161.7 (m, 2F). **HRMS m/z:** Not obtained due to this compound being isolated as a mixture with an isomer that has the same mass.

(3b) N-((6-chloro-2',3',4',6'-tetrafluoro-[1,1'-biphenyl]-3-yl)methyl)-1,1,1-trifluoromethanesulfonamide

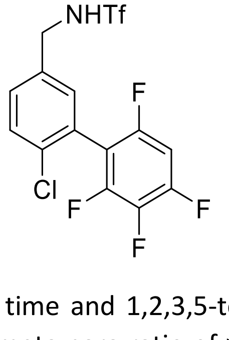

GP1 was followed with an 8 h reaction time and 1,2,3,5-tetrafluorobenzene (0.20 ml, 1.8 mmol). Analysis of the crude ^{1}H NMR showed a meta:para ratio of >20:1. The residue was purified *via* flash chromatography 3 times (eluting with EtOAc:Hexanes (10:90)) to give the title compound (in a >20:1 Cl_m:Cl_p ratio) as a yellow oil (63.1 mg, 0.150 mmol, 75%).

^{1}H NMR (400 MHz, $CDCl_3$) δ_H 7.56 (d, *J* = 8.3 Hz, 1H), 7.39 (dd, *J* = 2.1, 8.3 Hz, 1H), 7.28 (d, *J* = 2.1 Hz, 1H), 6.94-6.86 (m, 1H), 5.37 (br s, 1H), 4.47 (s, 2H); **^{13}C NMR** (101 MHz, $CDCl_3$) δ_C 154.3 (dm, *J* = 247.4 Hz), 151.2 (dm, *J* = 253.2 Hz), 148.7 (dm, *J* = 247.4 Hz), 137.4 (dm, *J* = 250.3 Hz), 135.0 134.3 131.5 130.6 130.0 127.7 119.6 (q, *J* = 320.9 Hz), 113.0-112.8 (m), 101.3-100.8 (ddd, *J* = 28.4, 21.4, 4.0 Hz), 47.2; **^{19}F NMR** (376 MHz, $CDCl_3$) δ_F -77.2 (s, 3F), -115.3 (dd, *J* = 10.8, 2.3 Hz, 1F), -131.1 (ddd, *J* = 21.6, 6.4, 2.3 Hz, 1F), -132.2 (dd, *J* = 21.5, 6.4 Hz, 1F), -164.5 (dt, *J* = 21.8, 11.1 Hz, 1F); **HRMS m/z:** $[M-H]^-$ calculated for $[C_{14}H_6ClF_7NO_2S]^-$ 419.9701, found 419.9703.

(3c) N-((6-chloro-2',3',5',6'-tetrafluoro-[1,1'-biphenyl]-3-yl)methyl)-1,1,1-trifluoromethanesulfonamide

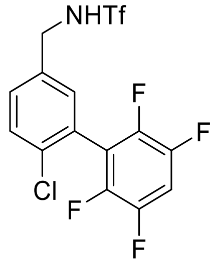

GP1 was followed with an 8 h reaction time and 1,2,4,5-tetrafluorobenzene (0.20 ml, 1.8 mmol). Analysis of the crude ^{1}H NMR showed a meta:para ratio of 14:1. The residue was purified *via* flash chromatography (eluting with CH_2Cl_2:Hexanes (50:50) *then* the mixture was purified again eluting with EtOAc:Hexanes (10:90)) to give the title compound (in a >20:1 Cl_m:Cl_p ratio) as a white amorphous solid (62.0 mg, 0.147 mmol, 74%).

^{1}H NMR (400 MHz, $CDCl_3$) δ_H 7.59 (d, *J* = 8.3 Hz, 1H), 7.42 (dd, *J* = 8.3, 2.1 Hz, 1H), 7.30 (d, *J* = 2.1 Hz, 1H), 7.22-7.13 (m, 1H), 5.18 (br s, 1H), 4.49 (s, 2H); **^{13}C NMR** (101 MHz, $CDCl_3$) δ_C 146.1 (dm, *J* = 238.7 Hz), 143.6 (dm, *J* = 246.2 Hz), 134.8, 134.3, 131.2, 130.7, 130.2, 127.7 (t, *J* = 2.3 Hz), 119.6 (q, *J* = 323.4 Hz), 118.3 (t, *J* = 18.3 Hz), 106.4 (t, *J* = 22.5 Hz), 47.28; **^{19}F NMR** (376 MHz, $CDCl_3$) δ_F -77.1 (s, 3F), -138.6 (q, *J* = 11.5 Hz, 2F), -140.4 (q, *J* = 11.4 Hz, 2F); **HRMS m/z:** $[M-H]^-$ calculated for $[C_{14}H_6ClF_7NO_2S]^-$ 419.9701, found 419.9709.

(3d) N-((6-chloro-2',4',6'-trifluoro-[1,1'-biphenyl]-3-yl)methyl)-1,1,1-trifluoromethanesulfonamide

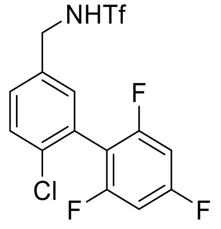

GP1 was followed with an 16 h reaction time and 1,3,5-tetrafluorobenzene (0.19 ml, 1.8 mmol). Analysis of the crude ^{1}H NMR with reference to DME (0.033 mmol) as an internal standard showed a NMR yield of 28:3:12:10 (Cl_m:Cl_p:SM:$SM_{H\text{-}Dechlorinated}$).The residue was purified *via* flash chromatography (CH_2Cl_2:Hexanes (50:50) *then* (EtOAc:Hexanes (10:90)) to give the title compound in a mixture with Cl_m:Cl_p:SM:$SM_{H\text{-}Dechlorinated}$ (5:1:3:3) as a colourless oil (*isolated mass* 8.2 mg, *calculated mass of meta + para* 4.8 mg, 0.0118 mmol, 6%).

By deduction of the starting material, and the hydro-dechlorinated starting material the meta isomer can be described as followed: **^{1}H NMR** (400 MHz, $CDCl_3$) δ_H 7.55 (d, *J* = 8.3 Hz, 1H), 7.37 (dd, *J* = 8.2,

2.2 Hz, 1H), 7.28 (d, J = 2.2 Hz, 1H), 6.80-6.78 (m, 2H), 5.18 (br s, 1H), 4.47 (s, 2H); **^{13}C NMR** (126 MHz, $CDCl_3$) δ_C 162.9 (dt, J = 251.1, 15.3 Hz), 160.2 (ddd, J = 250.4, 15.1, 9.6 Hz), 135.1, 134.0, 131.6, 130.5, 129.3, 128.7, 119.6 (q, J = 321.0 Hz), 111.8 (td, J = 20.6, 4.6 Hz), 100.7-100.2 (m), 47.36 **^{19}F NMR** (376 MHz, $CDCl_3$) δ_F -77.1 (s, 3F), -106.8 (t, J = 6.5 Hz, 1F), -108.7 (d, J = 6.7 Hz, 2F).**HRMS m/z:** [M-H]$^-$ calculated for $[C_{14}H_7ClF_6NO_2S]^-$ 401.9796, found 401.9794.

(3e) N-((6-chloro-2',6'-difluoro-4'-methoxy-[1,1'-biphenyl]-3-yl)methyl)-1,1,1-trifluoromethanesulfonamide

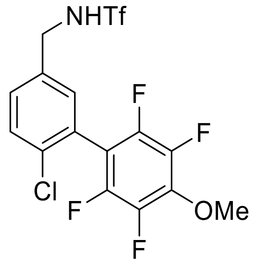

GP1 was followed with an 8 h reaction time and 1,2,4,5-tetrafluoro-3-methoxy benzene (0.20 ml, 1.8 mmol). Analysis of the crude ^{1}H NMR showed a meta:para ratio of 16:1. The residue was purified *via* flash chromatography (eluting with EtOAc:Hexanes (10:90) *then* the mixture was purified again eluting with (CH_2Cl_2:Hexanes (50:50)) to give the title compound (in a 16:1 Cl_m:Cl_p ratio) as a colourless oil (50.0 mg, 0.111 mmol, 55%).

^{1}H NMR (400 MHz, $CDCl_3$) δ_H 7.56 (d, J = 8.3 Hz, 1H), 7.39 (dd, J = 8.3, 2.0 Hz, 1H), 7.29 (d, J = 2.0 Hz, 1H), 5.23 (br s, 1H), 4.47 (s, 2H), 4.15 (t, J = 1.4 Hz, 3H); **^{13}C NMR** (101 MHz, $CDCl_3$) δ_C 144.2 (dm, J = 245.8 Hz), 140.9 (dm, J = 246.2 Hz), 138.8-138.6 (m), 135.1, 134.3, 131.6, 130.6, 130.0, 127.5 (t, J = 2.1 Hz), 119.6 (q, J = 320.9 Hz), 110.8 (t, J = 18.9 Hz), 62.2 (t, J = 3.8 Hz), 47.3; **^{19}F NMR** (376 MHz, $CDCl_3$) δ_F -77.1 (s, 3F), -141.6 - -141.8 (m, 2F), -157.8 - -157.9 (m, 2F); **HRMS m/z:** [M-H]$^-$ calculated for $[C_{15}H_8ClF_7NO_3S]^-$ 449.9807, found 449.9822.

(3f) N-((6-chloro-2',3',5',6'-tetrafluoro-4'-(trifluoromethyl)-[1,1'-biphenyl]-3-yl)methyl)-1,1,1-trifluoromethanesulfonamide

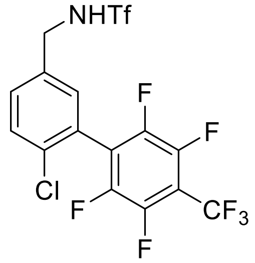

GP1 was followed with an 16 h reaction time and 2,3,5,6-tetrafluorobenzotrifluoride (0.25 ml, 1.8 mmol). Analysis of the crude ^{1}H NMR showed a meta:para ratio of >20:1. The residue was purified *via* flash chromatography (CH_2Cl_2:Hexanes (50:50)) *then* (EtOAc:Hexanes (10:90)) to give the title compound (in a >20:1 Cl_m:Cl_p ratio) as a colourless oil (89.5 mg, 0.183 mmol, 92%).

^{1}H NMR (400 MHz, $CDCl_3$) δ_H 7.61 (d, J = 8.3 Hz, 1H), 7.46 (dd, J = 8.3, 2.0 Hz, 1H), 7.30 (d, J = 2.0 Hz, 1H), 5.33 (br s, 1H), 4.49 (s, 3H); **^{13}C NMR** (101 MHz, $CDCl_3$) δ_C 144.2 (dm, J = 251.0 Hz) (*HSQC data suggests there are two carbons peaks overlapping*), 134.7, 134.4, 130.9, 130.8 (*HSQC data suggests there are two carbons peaks overlapping*), 126.3 (t, J = 2.0 Hz), 121.7 (t, J = 18.2 Hz), 120.7 (q, J = 276.6 Hz), 119.7 (q, J = 319.9 Hz), 110.1 (dt, J = 34.3, 12.6 Hz), 47.1.**^{19}F NMR** (376 MHz, $CDCl_3$) δ_F -56.4 (t, J =

21.8 Hz, 3F), -77.2 (s, 3F), -137.7 - -137.8 (m, 2F), -140.0 – 140.2 (m, 2F); **HRMS m/z:** $[M-H]^-$ calculated for $[C_{15}H_5ClF_{10}NO_2]^-$ 487.9575, found 487.9591.

(3g) N-((6-chloro-2',3',5',6'-tetrafluoro-4'-((triisopropylsilyl)ethynyl)-[1,1'-biphenyl]-3-yl)methyl)-1,1,1-trifluoromethanesulfonamide

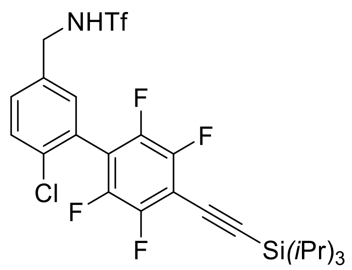

GP1 was followed with an 16 h reaction time and triisopropyl((2,3,5,6-tetrafluorophenyl)ethynyl)silane (330.1 mg, 1.8 mmol). Analysis of the crude ^{1}H NMR showed a meta:para ratio of 15:1. The residue was purified *via* flash chromatography (eluting with CH_2Cl_2:Hexanes (50:50) *then* the mixture was purified again eluting with (EtOAc:Hexanes (10:90)) to give the title compound (in a 15:1 Cl_m:Cl_p ratio) as an amorphous white solid (95.7 mg, 0.159 mmol, 80%).

^{1}H NMR (400 MHz, $CDCl_3$) δ_H 7.58 (d, *J* = 8.3 Hz, 1H), 7.42 (dd, *J* = 8.3, 2.0 Hz, 1H), 7.27 (d, *J* = 2.0 Hz, 1H), 5.35 (br s, 1H), 4.48 (s, 2H), 1.19-1.13 (m, 21H); **^{13}C NMR** (101 MHz, $CDCl_3$) δ_C 147.3 (dt, *J* = 253.5, 3.4 Hz), 143.6 (dm, *J* = 249.1 Hz), 134.8, 134.4, 131.2, 130.7, 130.3, 127.3 (t, *J* = 2.2 Hz), 119.6 (q, *J* = 321.0 Hz), 117.7 (t, *J* = 18.7 Hz), 107.2 (t, *J* = 3.8 Hz), 105.4-105.0 (m), 90.4 (t, *J* = 4.0 Hz), 47.2, 18.5, 11.2; **^{19}F NMR** (376 MHz, $CDCl_3$) δ_F -77.1 (s, 3F), -136.3 (q, *J* = 11.7 Hz, 2F), -140.8 (q, *J* = 11.5 Hz, 2F). **HRMS m/z:** $[M-H]^-$ calculated for $[C_{23}H_{26}ClF_7NO_2SSi]^-$ 600.1030, 600.1008

(3h) Methyl 2'-chloro-2,3,5,6-tetrafluoro-5'-(((trifluoromethyl)sulfonamido)methyl)-[1,1'-biphenyl]-4-carboxylate

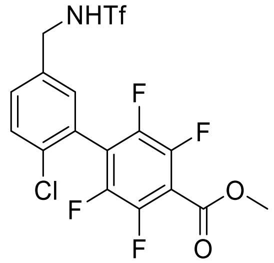

GP1 was followed with an 16 h reaction time and methyl 2,3,5,6-tetrafluorobenzoate (208.0 mg, 1.8 mmol). Analysis of the crude ^{1}H NMR showed a meta:para ratio of 13:1. The residue was purified *via* flash chromatography (eluting with EtOAc:Hexanes (10:90) *then* the mixture was purified again eluting with CH_2Cl_2:Hexanes (50:50)) *then* (EtOAc:Hexanes (10:90)) to give the title compound (in a >20:1 Cl_m:Cl_p ratio) as an amorphous white solid (26.5 mg, 0.058 mmol, 28%).

^{1}H NMR (400 MHz, $CDCl_3$) δ_H 7.60 (d, *J* = 8.3 Hz, 1H), 7.45 (dd, *J* = 8.3, 2.0 Hz, 1H), 7.29 (d, *J* = 2.0 Hz, 1H), 5.36 (br s, 1H), 4.49 (s, 2H), 4.02 (s, 3H); **^{13}C NMR** (101 MHz, $CDCl_3$) δ_C 160.1, 144.3 (dm, *J* = 261.7 Hz), 143.8 (m, *J* = 254.6 Hz), 134.6, 134.5, 130.9, 130.7, 130.6, 126.8 (t, *J* = 2.2 Hz), 120.6 (t, *J* = 18.6 Hz), 119.6 (q, *J* = 321.0 Hz), 112.8 (t, *J* = 15.7 Hz), 53.5, 47.2; **^{19}F NMR** (376 MHz, $CDCl_3$) δ_F -77.1 (s, 3F), -138.9 - -138.7 (m, 2F), -139.0 – 139.1 (m, 2F). **HRMS m/z:** $[M-H]^-$ calculated for $[C_{16}H_8ClF_7NO_4S]^-$ 477.9751, found 477.9763.

(3i) N-(2'-chloro-2,3,5,6-tetrafluoro-5'-(((trifluoromethyl)sulfonamido)methyl)-[1,1'-biphenyl]-4-yl)acetamide

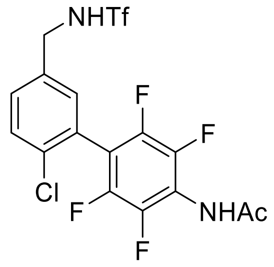

GP1 was followed with an 16 h reaction time and N-(2,3,5,6-tetrafluorophenyl)acetamide (206.0 mg, 1.8 mmol). Analysis of the crude ^{1}H NMR showed a meta:para ratio of 8:1. The residue was purified *via* flash chromatography (eluting with EtOAc:Hexanes (40:60) *then* the mixture was purified again eluting with EtOAc:Hexanes (30:70)) to give the title compound (in a 8:1 Cl_m:Cl_p ratio) as an amorphous white solid (69.3 mg, 0.145 mmol, 72%).

^{1}H NMR (400 MHz, MeOD) δ_H 7.63 (d, *J* = 8.4 Hz, 1H), 7.51 (dd, *J* = 8.4, 2.0 Hz, 1H), 7.42 (d, *J* = 2.0 Hz, 1H), 4.44 (s, 2H), 2.22 (s, 3H); **^{13}C NMR** (101 MHz, MeOD) δ_C 170.5, 143.8 (dm, *J* = 246.9 Hz), 142.4 (dt, *J* = 247.9, 3.9 Hz), 136.7, 133.4, 131.1, 130.2, 129.8, 126.5 (t, *J* = 2.1 Hz), 112.0 (q, *J* = 320.8 Hz), 117.0 (t, *J* = 15.0 Hz), 115.7 (t, *J* = 18.9 Hz), 45.9, 21.0; **^{19}F NMR** (376 MHz, MeOD) δ_F -79.4 (s, 3F), -143.7 (q, *J* = 10.8 Hz, 2F), -147.9 (q, *J* = 11.0 Hz, 2F); **HRMS m/z:** $[M-H]^-$ calculated for $[C_{16}H_9ClF_7N_2O_3S]^-$ 476.9916, found 476.9913.

(3j) N-((6-chloro-2',2'',3',3'',4'',5',5'',6',6''-nonafluoro-[1,1':4',1''-terphenyl]-3-yl)methyl)-1,1,1-trifluoromethanesulfonamide

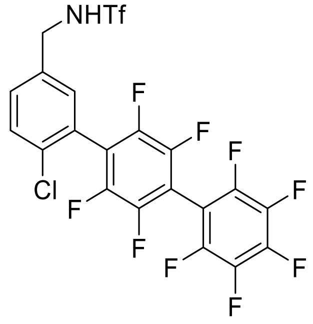

GP1 was followed with an 8 h reaction time and methyl 2,2',3,3',4,5,5',6,6'-nonafluoro-1,1'-biphenyl (315.9.0 mg, 1.8 mmol). Analysis of the crude ^{1}H NMR showed a meta:para ratio of 19:1. The residue was purified *via* flash chromatography (eluting with EtOAc:Hexanes (10:90) *then* the mixture was purified again eluting with CH_2Cl_2:Hexanes (50:50)) to give the title compound (in a >20:1 Cl_m:Cl_p ratio) as an amorphous white solid (75.8 mg, 0.129 mmol, 65%).

^{1}H NMR (400 MHz, $CDCl_3$) δ_H 7.62 (d, *J* = 8.3 Hz, 1H), 7.45 (dd, *J* = 8.3, 2.1 Hz, 1H), 7.38 (d, *J* = 2.1 Hz, 1H), 5.37 (br s, 1H), 4.51 (s, 2H); **^{13}C NMR** (101 MHz, $CDCl_3$) δ_C 145.80, 144.6 (dm, *J* = 253.1 Hz), 144.0 (d, *J* = 255.5 Hz, 2C), 142.6 (dm, *J* = 258.4 Hz), 137.9 (dm, *J* = 251.8 Hz), 134.7, 134.6, 131.1, 130.8, 130.5, 127.0 (t, *J* = 2.2 Hz), 119.7 (q, *J* = 321.1 Hz), 119.8 (t, *J* = 18.5 Hz), 106.6 (t, *J* = 18.9 Hz), 102.4 (t, *J* = 19.2 Hz), 47.2; **^{19}F NMR** (376 MHz, $CDCl_3$) δ_F -77.0 (s, 3F), -137.1 (m, *J* = 5.5 Hz, 1F), -137.1 (dm, *J* = 91.3 Hz, 2F), -137.8 - -138.1 (m, 2F), -138.8 - -138.9 (m, 2F), -149.9 (tt, *J* = 20.9, 2.5Hz, 1F), -160.3 - -160.5 (m, 2F); **HRMS m/z:** $[M-H]^-$ calculated for $[C_{20}H_5ClF_{12}NO_2S]^-$ 585.9543, found 585.9570.

7. Fluorinated pyridine scope

(5a) N-(4-chloro-3-(perfluoropyridin-4-yl)benzyl)-1,1,1-trifluoromethanesulfonamide

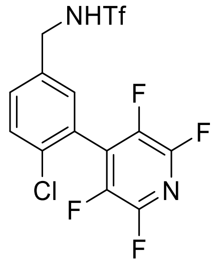

GP1 was followed with an 8 h reaction time and 2,3,5,6-tetrafluoropyridine (0.18 ml, 1.8 mmol). Analysis of the crude ^{1}H NMR showed a meta:para ratio of >20:1. The residue was purified *via* flash chromatography (eluting with CH_2Cl_2:Hexanes (70:30) *then* the mixture was purified again eluting with EtOAc:Hexanes (15:85)) to give the title compound (in a >20:1 Cl_m:Cl_p ratio) as an amorphous white solid (71.0 mg, 0.168 mmol, 84%).

^{1}H NMR (400 MHz, $CDCl_3$) δ_H 7.62 (d, *J* = 8.3 Hz, 1H), 7.48 (dd, *J* = 8.3, 1.8 Hz, 1H), 7.32 (d, *J* = 1.8 Hz, 1H), 5.45 (br s, 1H), 4.50 (s, 2H); **^{13}C NMR** (101 MHz, $CDCl_3$) δ_C 143.6 (dm, *J* = 246.6 Hz), 139.4 (dm, *J* = 259.7 Hz), 134.9, 134.0, 131.1, 131.0, 130.6 (t, *J* = 15.3 Hz), 130.3, 126.0 (br s), 119.56 (q, *J* = 320.9 Hz), 47.1; **^{19}F NMR** (376 MHz, $CDCl_3$) δ_F -77.1 (s, 3F), -89.6 - -89.8 (m, 2F), -140.8 - -141.0 (m, 2F); **HRMS m/z:** $[M-H]^-$ calculated for $[C_{13}H_5ClF_7N_2O_2S]^-$ 420.9654, found 420.9656.

(5b) N-(4-chloro-3-(2,3,5-trifluoropyridin-4-yl)benzyl)-1,1,1-trifluoromethanesulfonamide

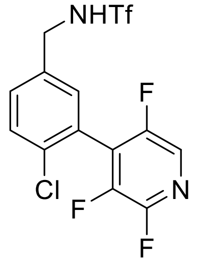

GP1 was followed with an 16 h reaction time and 2,3,5-trifluoropyridine (0.17 ml, 1.8 mmol). Analysis of the crude ^{1}H NMR showed a meta:para ratio of 13:1. The residue was purified *via* flash chromatography (eluting with CH_2Cl_2:Hexanes (70:30) *then* the mixture was purified again eluting with EtOAc:Hexanes (15:85)) to give the title compound (in a 13:1 Cl_m:Cl_p ratio) as an amorphous cream coloured solid (64.5 mg, 0.160 mmol, 80%).

^{1}H NMR (400 MHz, $CDCl_3$) δ_H 7.98 (s, 1H), 7.60 (d, *J* = 8.3 Hz, 1H), 7.46 (dd, *J* = 8.3, 1.7 Hz, 1H), 7.31 (d, *J* = 1.7 Hz, 1H), 5.52 (br s, 1H), 4.50 (s, 2H); **^{13}C NMR** (101 MHz, $CDCl_3$) δ_C 153.9 (dd, *J* = 258.0 Hz), 148.4 (ddd, *J* = 237.7, 15.6, 2.2 Hz), 142.3 (ddd, *J* = 266.2, 31.2, 3.1 Hz), 134.7, 134.1, 130.83, 130.75, 130.5, 128.5 (ddd, *J* = 28.1, 14.1, 6.5 Hz), 127.1 (ddd, *J* = 20.1, 14.3, 3.6 Hz), 126.4 (d, *J* = 2.5 Hz), 119.6 (q, *J* = 320.9 Hz), 47.1; **^{19}F NMR** (376 MHz, $CDCl_3$) δ_F -77.1 (s, 3F), -88.5 (t, *J* = 27.1 Hz, 1F), -129.1 (d, *J* = 28.8 Hz, 1F), -136.3 (d, *J* = 25.6 Hz, 1F); **HRMS m/z:** $[M-H]^-$ calculated for $[C_{13}H_6ClF_6N_2O_2S]^-$ 402.9748, found 402.9751.

(5c) N-(4-chloro-3-(2,4,6-trifluoropyridin-3-yl)benzyl)-1,1,1-trifluoromethanesulfonamide

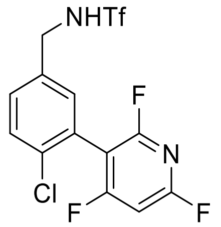

GP1 was followed with an 16 h reaction time and 2,4,6-trifluoropyridine (0.17 ml, 1.8 mmol). Analysis of the crude ^{1}H NMR showed a meta:para ratio of 10:1. The residue was purified *via* flash chromatography (eluting with EtOAc:Hexanes (15:85)) to give the title compound (in a 11:1 Cl_m:Cl_p ratio) as an amorphous white coloured solid (54.6 mg, 0.135 mmol, 63%).

^{1}H NMR (400 MHz, $CDCl_3$) δ_H 7.57 (d, *J* = 8.3 Hz, 1H), 7.41 (dd, *J* = 8.3, 2.1 Hz, 1H), 7.29 (d, *J* = 2.1 Hz, 1H), 6.75-6.72 (m, 1H), 5.34 (br s, 1H), 4.49 (s, 2H); **^{13}C NMR** (101 MHz, $CDCl_3$) δ_C 170.1 (ddd, *J* = 265.1, 12.9, 7.8 Hz), 162.0 (ddd, *J* = 246.2, 18.3, 16.6 Hz), 159.3 (ddd, *J* = 245.2, 18.8, 10.9 Hz), 134.9, 134.5, 131.3, 130.6, 130.2, 126.7 (d, *J* = 3.4 Hz), 119.59 (q, *J* = 320.9 Hz), 107.4 (ddd, *J* = 33.7, 19.4, 7.1 Hz), 95.6 (ddd, *J* = 39.2, 24.8, 6.6 Hz), 47.2; **^{19}F NMR** (376 MHz, $CDCl_3$) δ_H -63.7 (dd, *J* = 20.6, 12.1 Hz, 1F), -65.7 (dd, *J* = 18.2, 13.1 Hz, 1F), -77.0 (s, 3F), -92.2 (dd, *J* = 20.5, 18.2 Hz, 1F); **HRMS m/z:** $[M-H]^-$ calculated for $[C_{13}H_6ClF_6N_2O_2S]^-$ 402.9748, found 402.9750.

(5d) N-(4-chloro-3-(3,5-difluoropyridin-4-yl)benzyl)-1,1,1-trifluoromethanesulfonamide

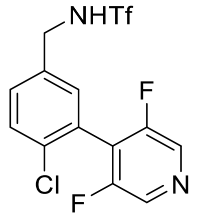

GP1 was followed with an 16 h reaction time and 3,5-difluoropyridine (0.16 ml, 1.8 mmol) in THF at 100 °C. Analysis of the crude ^{1}H NMR showed a meta:para ratio of 12:1. The residue was purified *via* flash chromatography (eluting with EtOAc:Hexanes (15:85)) to give the title compound (in a 12:1 Cl_m:Cl_p ratio) as an amorphous white coloured solid (28.2 mg, 0.073 mmol, 37%).

^{1}H NMR (400 MHz, $CDCl_3$) δ_H 8.38 (s, 2H), 7.59 (d, *J* = 8.3 Hz, 1H), 7.44 (dd, *J* = 8.3, 2.0 Hz, 1H), 7.30 (d, *J* = 2.0 Hz, 1H), 6.34 (br s, 1H), 4.49 (s, 2H); **^{13}C NMR** (101 MHz, $CDCl_3$) δ_C 156.2 (dd, *J* = 262.5, 2.0 Hz), 134.7, 134.4-134.1 (m), 134.2, 130.71, 130.5 (*HSQC data suggests this is 2 carbon peaks overlapping*), 126.8, 123.1 (t, *J* = 17.1 Hz), 119.7 (q, *J* = 321.0 Hz), 47.1; **^{19}F NMR** (376 MHz, $CDCl_3$) δ_F -77.1 (s, 3F), -126.0 (s, 2F); **HRMS m/z:** $[M-H]^-$ calculated for $[C_{13}H_7ClF_5N_2O_2S]^-$ 384.9842, found 384.9843.

8. Dichloroarene scope

General procedure for Direct arylation for different dichloroarenes (GP2)

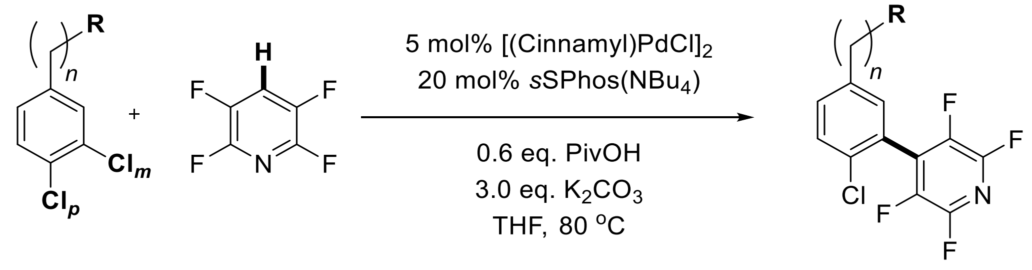

The 3,4-dichloroarene bearing a directing group (0.20 mmol, 1.0 eq.), sSPhos$_{(NBu4)}$ (29.2 mg, 0.04 mmol, 20 mol%), [(Cinnamyl)PdCl]$_2$ (5.2 mg, 0.01 mmol, 5 mol%), K_2CO_3 (82.9 mg, 0.6 mmol, 3.0 eq.) were added to a 4 mL 15x45 mm crimp top vial containing a stirrer bar. The vial was evacuated and back filled with argon 3 times, at which point THF (0.2 ml) and 2,3,5,6-tetrafluoropyridine (0.18 ml, 1.8 mmol, 9.0 eq.) were added and the top wrapped with parafilm. The reaction mixture was stirred for 16 h at 80 °C in a heating block, the solvent was then removed under reduced pressure.

(5e) N-(4-chloro-3-(perfluoropyridin-4-yl)phenethyl)-1,1,1-trifluoromethanesulfonamide

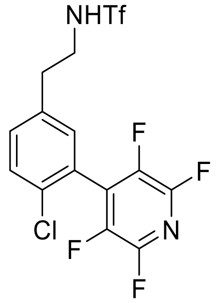

GP2 was followed with N-(3,4-dichlorophenethyl)-1,1,1-trifluoromethanesulfonamide (64.2 mg, 0.20 mmol). Analysis of the crude ^{1}H NMR showed a meta:para ratio of 6:1. The residue was purified *via* flash chromatography (EtOAc:Hexanes (15:85)) *then* (CH_2Cl_2:Hexanes (50:50)) to give the title compound (in a 6:1 Cl_m:Cl_p ratio) as a colourless oil that solidified on standing (55.2 mg, 0.127 mmol, 64%).

^{1}H NMR (400 MHz, $CDCl_3$) δ_H 7.57 (d, *J* = 8.3 Hz, 1H), 7.35 (dd, *J* = 8.3, 1.9 Hz, 1H), 7.18 (d, *J* = 1.9 Hz, 1H), 4.90 (br s, 1H), 3.59 (q, *J* = 5.4 Hz, 2H), 2.97 (t, *J* = 7.0 Hz, 2H); **^{13}C NMR** (101 MHz, $CDCl_3$) δ_C 143.7 (dm, *J* = 245.4 Hz), 139.2 (q, *J* = 259.4 Hz), 136.2, 132.6, 132.2, 131.4, 131.1-130.7 (m), 130.9, 125.8 (br s), 119.5 (q, *J* = 320.2 Hz), 45.1, 36.1; **^{19}F NMR** (376 MHz, $CDCl_3$) δ_F -77.4 (s, 3F), -90.0 (td, *J* = 28.6, 8.0 Hz, 2F), -140.9 (td, *J* = 23.8, 8.2 Hz, 2F); **HRMS m/z:** $[M-H]^-$ calculated for $[C_{14}H_7ClF_7N_2O_2S]^-$ 434.9810, found 434.9826.

(5f) N-(3-(4-chloro-3-(perfluoropyridin-4-yl)phenyl)propyl)-1,1,1-trifluoromethanesulfonamide

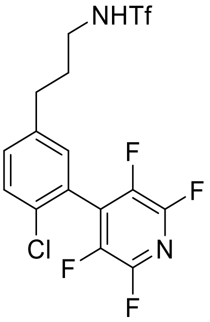

GP2 was followed with N-(3-(3,4-dichlorophenyl)propyl)-1,1,1-trifluoromethanesulfonamide (67.0 mg, 0.20 mmol). Analysis of the crude ^{1}H NMR showed a meta:para ratio of 5:1. The residue was purified *via* flash chromatography (EtOAc:Hexanes (15:85)) *then* (CH_2Cl_2:Hexanes (50:50)) to give the title compound (in a 5:1 Cl_m:Cl_p ratio) as a colourless oil (48.2 mg, 0.107 mmol, 54%).

^{1}H NMR (400 MHz, $CDCl_3$) δ_H 7.52 (d, *J* = 8.3 Hz, 1H), 7.31 (dd, *J* = 8.3, 1.9 Hz, 1H), 7.15 (d, *J* = 1.9 Hz, 1H), 4.96 (br s, 1H), 3.36 (t, *J* = 7.0 Hz, 2H), 2.75 (t, *J* = 7.8 Hz, 2H), 1.97 (app quintet, *J* = 7.2 Hz, 2H); **^{13}C NMR** (101 MHz, $CDCl_3$) δ_C 143.55 (dm, *J* = 246.1 Hz), 139.2 (t, *J* = 260.2 Hz), 139.7, 131.7, 131.5, 131.4-131.2 (m), 130.8, 130.4, 125.4, 119.56 (q, *J* = 321.0 Hz), 43.7, 31.6 (*HMBC and HSQC suggest two carbons under this peak*). **^{19}F NMR** (376 MHz, $CDCl_3$) δ_F -77.2 (s, 3F), -90.2 (td, *J* = 29.0, 14.3 Hz, 2F), -141.02 (td, *J* = 28.6, 13.0 Hz, 2F); **HRMS m/z:** $[M-H]^-$ calculated for $[C_{15}H_9ClF_7N_2O_2S]^-$ 448.9967, found 448.9986.

(5g) N-(1-(4-chloro-3-(perfluoropyridin-4-yl)phenyl)ethyl)-1,1,1-trifluoromethanesulfonamide

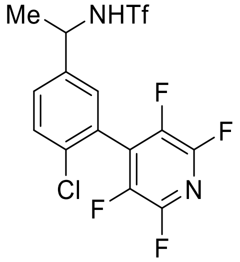

GP2 was followed with N-(1-(3,4-dichlorophenyl)ethyl)-1,1,1-trifluoromethanesulfonamide (64.0 mg, 0.20 mmol). Analysis of the crude ^{1}H NMR showed a meta:para ratio of 9:1. The residue was purified *via* flash chromatography (EtOAc:Hexanes (5:95)) to give the title compound (in a 9:1 Cl_m:Cl_p ratio) as a colourless oil (72.8 mg, 0.167 mmol, 83%).

^{1}H NMR (400 MHz, $CDCl_3$) δ_H 7.61 (d, *J* = 8.4 Hz, 1H), 7.46 (dd, *J* = 8.4, 2.1 Hz, 1H), 7.30 (d, *J* = 2.1 Hz, 1H), 5.53 (br s, 1H), 4.84 (q, *J* = 6.8 Hz, 1H), 1.66 (d, *J* = 7.0 Hz, 3H); **^{13}C NMR** (101 MHz, $CDCl_3$) δ_C 143.9 (ddd, *J* = 246.4, 13.2, 2.6 Hz), 140.6, 140.56 (dm, *J* = 260.4 Hz), 133.6, 130.9, 130.8 (tt, *J* = 16.9, 3.1 Hz), 129.4, 128.7, 125.9, 119.4 (q, *J* = 320.8 Hz), 54.2, 23.1; **^{19}F NMR** (376 MHz, $CDCl_3$) δ_F -77.41 (s, 3F), -89.8 - -90.0 (m, 2F), -140.7 - -141.1 (m, 2F); **HRMS m/z:** $[M-H]^-$ calculated for $[C_{14}H_7ClF_7N_2O_2S]^-$ 434.9810, found 434.9814.

(5h) N-((4-chloro-3-(perfluoropyridin-4-yl)phenyl)(phenyl)methyl)-1,1,1-trifluoromethanesulfonamide

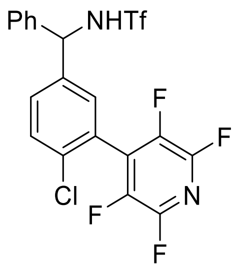

GP2 was followed with N-((4-chloro-3-(perfluoropyridin-4-yl)phenyl)(phenyl)methyl)-1,1,1-trifluoromethanesulfonamide (76.6 mg, 0.20 mmol). Analysis of the crude ^{1}H NMR showed a meta:para ratio of 14:1. The residue was purified *via* flash chromatography (EtOAc:Hexanes (5:95)) *then* (CH_2Cl_2:hexanes (50:50)) to give the title compound (in a 14:1 Cl_m:Cl_p ratio) as a colourless oil (74.2 mg, 0.149 mmol, 74%).

^{1}H NMR (400 MHz, $CDCl_3$) δ_H 7.61 (d, *J* = 8.4 Hz, 1H), 7.45-7.37 (m, 4H), 7.25-7.21 (m, 2H), 5.92 (d, *J* = 8.8 Hz, 1H), 5.62 (d, *J* = 8.8 Hz, 1H); **^{13}C NMR** (101 MHz, $CDCl_3$) δ_C 143.6 (dm, *J* = 250.9 Hz), 139.2 (q, *J* = 260.8 Hz), 139.2, 138.2, 133.7, 130.8, 130.8-130.4 (m), 130.5, 129.8, 129.5, 129.1, 127.2, 125.8, 119.4 (q, *J* = 320.9 Hz), 61.5; **^{19}F NMR** (376 MHz, $CDCl_3$) δ_F -77.06 (s, 3F), -89.6 - -89.9 (m, 2F), -140.7 – 140.9 (m, 2F); **HRMS m/z:** $[M-H]^-$ calculated for $[C_{19}H_9ClF_7N_2O_2S]^-$ 496.9967, found 496.9970.

(5i) N-butyl-1-(4-chloro-3-(perfluoropyridin-4-yl)phenyl)methanesulfonamide

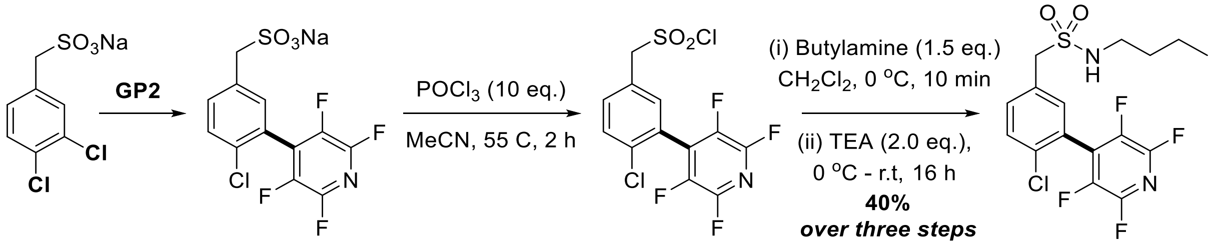

GP2 was followed with sodium (3,4-dichlorophenyl)methanesulfonate (53.0 mg, 0.20 mmol). The vial was then sealed, evacuated, and back filled with argon 3 times. MeCN (1.0 ml) was added to the residue followed by the dropwise addition of $POCl_3$ (0.19 ml, 2.0 mmol, 10 eq.) (C*aution! The remaining K_2CO_3 present in the reaction mixture results in a considerable release of gas*). The resulting mixture was stirred at 55 °C for 2 hours. The volatiles were then removed under a stream of nitrogen and H_2O and CH_2Cl_2 (2 ml) were added. The layers were separated and the aqueous layer was extracted with CH_2Cl_2 (3 x 2 ml). The combined organics were dried over $MgSO_4$, filtered and concentrated *in vacuo*. The sulfonyl chloride was then redissolved in CH_2Cl_2 (2.0 ml) and cooled to 0 °C under argon at which point butylamine (0.03 ml, 0.3 mmol, 1.5 eq.) was added dropwise. After 10 minutes, triethylamine (0.05 ml, 0.4 mmol, 2.0 eq.) was added dropwise and left to stir at room temperature for 16 h. The solvent was removed under reduced pressure, and analysis of the crude ^{1}H NMR showed a meta:para ratio of 14:1. The residue was purified *via* column chromatography (eluting with EtOAc:Hexanes (15:85) *then* the mixture was purified again eluting with CH_2Cl_2) to give the title compound (in a >20:1 Cl_m:Cl_p ratio) as a white amorphous solid (10.0 mg, 0.0243 mmol, 12%) and a separate set of fractions containing the title compound (in a 9:1 Cl_m:Cl_p ratio) as a white amorphous solid (22.6 mg, 0.0551 mmol, 28%) resulting in an overall yield of (in a 15:1 Cl_m:Cl_p ratio) (32.6 mg, 0.0794 mmol, 40%).

^{1}H NMR (400 MHz, $CDCl_3$) δ_H 7.62 (d, *J* = 8.3 Hz, 1H), 7.54 (dd, *J* = 8.3, 2.0 Hz, 1H), 7.39 (d, *J* = 2.0 Hz, 1H), 4.26 (s, 2H), 4.14 (t, *J* = 6.0 Hz, 1H), 3.01 (q, *J* = 6.8 Hz, 2H), 1.47 (quin, *J* = 7.1 Hz, 2H), 1.31 (sext, *J* = 7.4 Hz, 2H), 0.89 (t, *J* = 7.3 Hz, 3H); **^{13}C NMR** (101 MHz, $CDCl_3$) δ_C 143.5 (dm, *J* = 243.8 Hz), 139.4 (dm, *J* =

260.7 Hz), 134.2, 133.9, 133.1, 130.7, 130.6, 129.1, 125.7, 57.9, 43.6, 32.4, 19.6, 13.5; **^{19}F NMR** (376 MHz, $CDCl_3$) δ_F -89.6 - -89.8 (m, 2F), -140.8 - -140.9 (m, 2F); **HRMS m/z:** $[M\text{-}H]^-$ calculated for $[C_{16}H_{14}ClF_4N_2O_2S]^-$ 409.0406, found 409.0404.

(5j) N-(4-chloro-3-(perfluoropyridin-4-yl)benzyl)-2,2,2-trifluoroacetamide

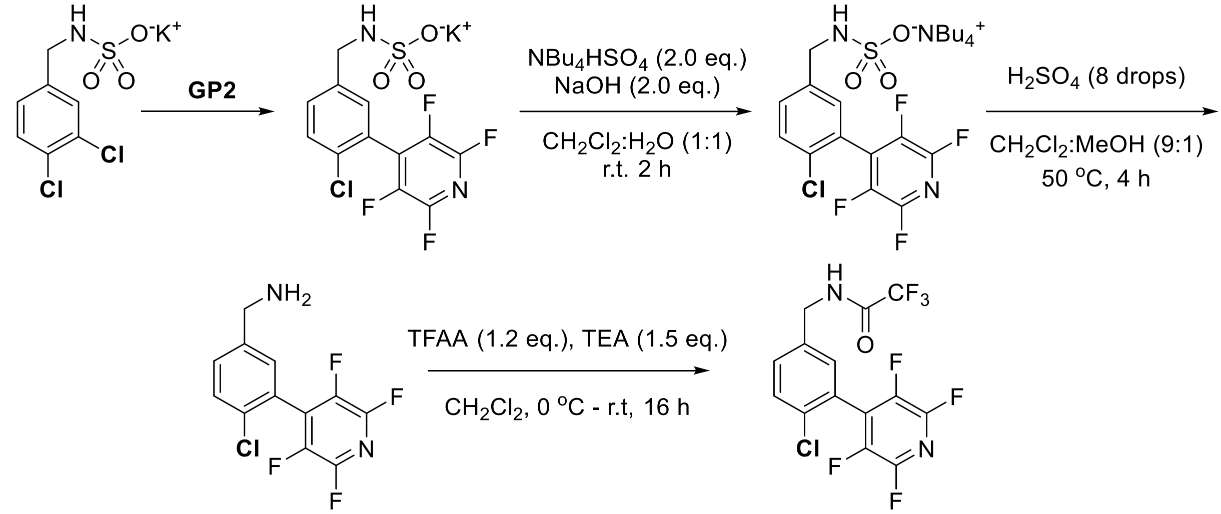

GP2 was followed with potassium (3,4-dichlorobenzyl)sulfamate (58.4.0 mg, 0.20 mmol). Tetrabutylammonium hydrogen sulfate (135.8 mg, 0.4 mmol, 2.0 eq.) and NaOH (16 mg, 0.4 mmol, 2.0 eq.) were added and the stirred rapidly in a biphasic system of CH_2Cl_2 and H_2O (1:1, 2.0 ml) for 2 hours. The layers were then separated and the aqueous was extracted with CH_2Cl_2 (5 x 2 ml), the combined organics were dried over $MgSO_4$, filtered and concentrated *in vacuo.* The vessel was then sealed, evacuated, and back filled with argon 3 times at which point a mixture of CH_2Cl_2 (0.9 ml) and MeOH (0.1 ml) were added. H_2SO_4 (conc. 8 drops) was then added dropwise and allowed to stir at room temperature until LCMS showed full deprotection (*caution! Allowing this deprotection step to proceed for longer than necessary results in low mass balance*). Triethylamine (0.5 ml) was then added and the mixture concentrated to dryness under reduced pressure. The crude amine was dissolved in dry CH_2Cl_2 (1.0 ml) and cooled to 0 °C. Triethylamine (0.06 ml, 0.4 mmol) and trifluoroacetic anhydride (0.06 ml, 0.4 mmol) were added and the resulting mixture was allowed to warm up to room temperature and stirred for 16 hours. The solvent was evaporated and analysis of the crude ^{1}H NMR showed a meta:para ratio of 14:1. The residue was purified *via* column chromatography (eluting with EtOAc:Hexanes (20:80)) *then* the mixture was purified again eluting with (EtOAc:Hexanes (20:80)) to give the title compound (in a 13:1 Cl_m:Cl_p ratio) as a white amorphous solid (40.2 mg, 0.129 mmol, 65%).

^{1}H NMR (400 MHz, $CDCl_3$) δ_H 7.59 (d, *J* = 8.3 Hz, 1H), 7.44 (dd, *J* = 8.3, 1.9 Hz, 1H), 7.28 (d, *J* = 1.9 Hz, 1H), 6.82 (br s, 1H), 4.58 (d, *J* = 6.1 Hz, 2H); **^{13}C NMR** (101 MHz, $CDCl_3$) δ_C 157.4 (q, *J* = 37.6 Hz), 143.6 (dm, *J* = 245.9 Hz), 139.4 (dm, *J* = 262.4 Hz), 135.6, 133.6, 131.3, 130.9, 130.7 (t, *J* = 3.1 Hz), 130.5, 125.9, 115.7 (q, *J* = 287.8 Hz), 42.8; **^{19}F NMR** (376 MHz, $CDCl_3$) δ_F -75.7 (s, 3F), -89.7 – -89.9 (m, 2F), -140.8 - -141.0 (m, 2F). **HRMS m/z:** $[M\text{-}H]^-$ $[C_{14}H_5ClF_7N_2O]^-$ 384.9984, found 384.9982.

(5k) Ethyl methyl (4-chloro-3-(perfluoropyridin-4-yl)benzyl)phosphonate

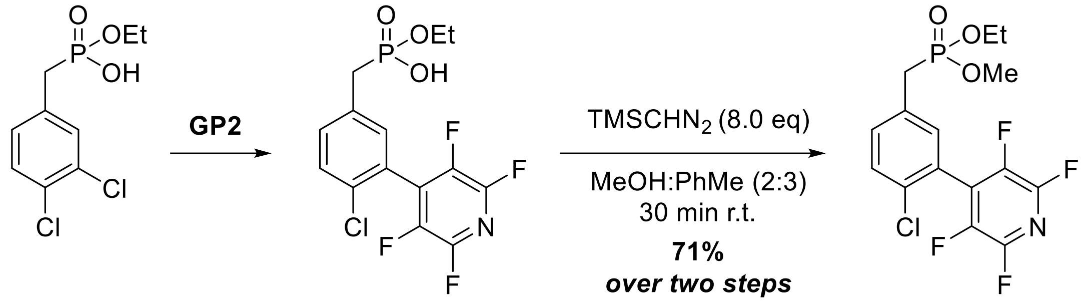

GP2 was followed with ethyl hydrogen (3,4-dichlorobenzyl)phosphonate (53.4 mg, 0.20 mmol). The vial was then sealed, evacuated, and back filled with argon 3 times. The residue was then redissolved in PhMe:MeOH (3:2, 2.5 mL) and (Trimethylsilyl)diazomethane (2M in ether, 1.0 mL, 8 equiv.) added. The solution was stirred at room temperature for 30 minutes, then AcOH (0.1 mL) added to quench excess (Trimethylsilyl)diazomethane. The solvent was removed under reduced pressure, and analysis of the crude ^{1}H NMR showed a meta:para ratio of 12:1. The residue was purified *via* column chromatography (eluting with EtOAc:Hexanes:triethylamine (70:29:1)) to give the title compound (in a 12:1 Cl_m:Cl_p ratio) as a colourless oil (56.2 mg, 0.142 mmol, 71%).

^{1}H NMR (400 MHz, $CDCl_3$) δ_H 7.52 (d, J = 8.3 Hz, 1H), 7.42 (dt, J = 8.3, 2.2 Hz, 1H), 7.27 (t, J = 2.2 Hz, 1H), 4.08-4.00 (m, 2H), 3.67 (d, J = 11.0 Hz, 3H), 3.16 (d, J = 21.8 Hz, 2H), 1.24 (t, J = 7.1 Hz, 3H); **^{13}C NMR** (101 MHz, $CDCl_3$) δ_C 143.5 (dm, J = 249.1 Hz), 139.4 (dm, J = 260.1 Hz), 133.1 (d, J = 6.5 Hz), 132.2 (d, J = 5.4 Hz) (*HMBC suggests an overlapping carbon peak here*), 132.1, 131.3 (d, J = 9.1 Hz), 131.3-130.8 (m), 130.3 (d, J = 3.0 Hz), 125.3 (br s), 62.7 (d, J = 6.8 Hz), 52.8 (d, J = 6.9 Hz), 32.6 (d, J = 139.2 Hz), 16.3 (d, J = 5.7 Hz); **^{19}F NMR** (376 MHz, $CDCl_3$) δ_F -90.1 - -90.3 (m, 2F), -140.8 - -141.1 (m, J = 10.8 Hz, 1F); **^{31}P NMR** (162 MHz, $CDCl_3$) δ_P 25.9. **HRMS m/z:** $[M+H]^+$ calculated for $[C_{15}H_{14}ClF_4NO_3P]^+$ 398.0330, found 398.0333

(5l) Methyl 4-chloro-3-(perfluoropyridin-4-yl)benzoate

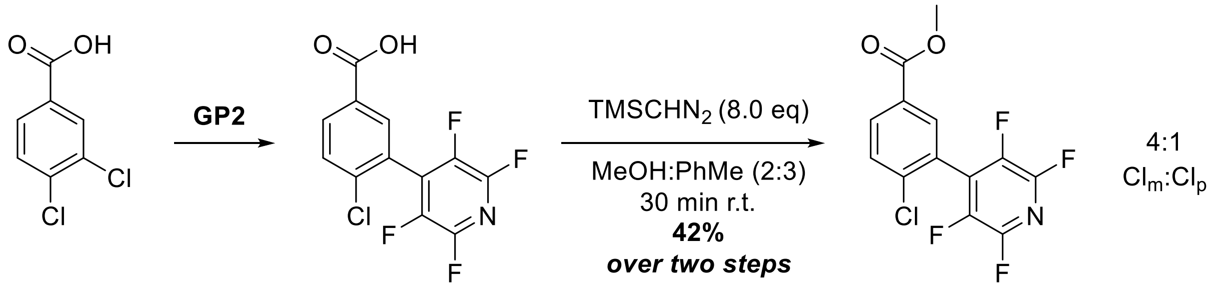

GP2 was followed with 3,4-dichlorobenzoic acid (38.2 mg, 0.20 mmol). The vial was then sealed, evacuated, and back filled with argon 3 times. The residue was then redissolved in PhMe:MeOH (3:2, 2.5 mL) and (Trimethylsilyl)diazomethane (2M in ether, 1.0 mL, 8 equiv.) added. The solution was stirred at room temperature for 30 minutes, then AcOH (0.1 mL) added to quench excess (Trimethylsilyl)diazomethane. The solvent was removed under reduced pressure, and analysis of the crude ^{1}H NMR showed a meta:para ratio of 4:1. The residue purified *via* column chromatography (eluting with CH_2Cl_2:hexanes (30:70)) to give the title compound (in a 4:1 Cl_m:Cl_p ratio) as an amorphous white solid (27.1 mg, 0.0848 mmol, 42%).

By deduction of the isolated para isomer (**6i**) the meta isomer can be described as followed: **^{1}H NMR** (400 MHz, $CDCl_3$) δ_H 8.16 (dd, J = 8.4, 1.9 Hz, 1H), 8.03 (d, J = 1.9 Hz, 1H), 7.67 (d, J = 8.4 Hz, 1H), 3.95 (s, 3H); **^{13}C NMR** (101 MHz, $CDCl_3$) δ_C 165.2, 143.6 (dm, J = 246.2 Hz), 139.2 (dm, J = 261.0 Hz), 138.4, 132.7, 132.3, 130.6, 130.5, 129.5, 125.5, 52.6; **^{19}F NMR** (376 MHz, $CDCl_3$) δ_F -89.6 - -89.8 (m, 2F), -140.7

- -140.9 (m, 2F); **HRMS m/z:** Not obtained due to this compound being isolated as a mixture with an isomer that has the same mass.

(6l) Methyl 2-(3-chloro-4-(perfluoropyridin-4-yl)phenyl)acetate

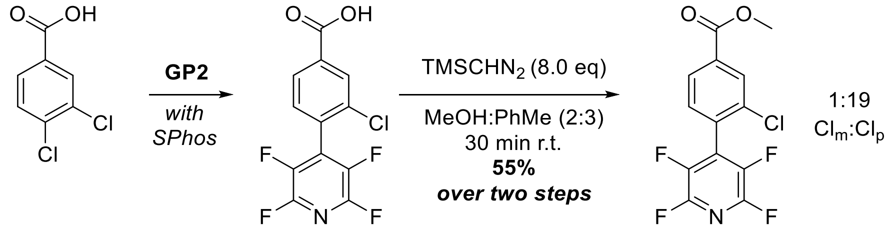

GP2 was followed with 2-(3,4-dichlorophenyl)acetic acid (41.0 mg, 0.20 mmol) and SPhos instead of $sSPhos_{(NBu4)}$. The vial was then sealed, evacuated, and back filled with argon 3 times. The residue was then redissolved in PhMe:MeOH (3:2, 2.5 mL) and (Trimethylsilyl)diazomethane (2M in ether, 1.0 mL, 8 equiv.) added. The solution was stirred at room temperature for 30 minutes, then AcOH (0.1 mL) added to quench excess (Trimethylsilyl)diazomethane. The solvent was removed under reduced pressure, and analysis of the crude ^{1}H NMR showed a meta:para ratio of 1:19. The residue was then purified *via* column chromatography (eluting with CH_2Cl_2:hexanes (30:70)) to give the title compound (in a 1:>20 Cl_m:Cl_p ratio) as amorphous white solid (35.0 mg, 0.110 mmol, 55%).

^{1}H NMR (400 MHz, $CDCl_3$) δ_H 8.25 (d, *J* = 1.5 Hz, 1H), 8.09 (dd, *J* = 8.0, 1.5 Hz, 1H), 7.44 (d, *J* = 8.0 Hz, 1H), 3.98 (s, 3H); **^{13}C NMR** (101 MHz, $CDCl_3$) δ_C 165.0, 143.6 (dm, *J* = 246.2 Hz), 139.2 (dm, *J* = 260.4 Hz), 134.0, 133.7, 131.3, 131.2, 130.48 (tt, *J* = 16.5, 3.3 Hz), 129.4 (br s), 128.1, 52.8; **^{19}F NMR** (376 MHz, $CDCl_3$) δ_F -89.5 - -89.7 (m, 2F), -140.6 - -140.8 (m, 2F). **HRMS m/z:** $[M+H]^+$ $[C_{13}H_7ClF_4NO_2]^+$ 320.0096, found 320.0084.

(5m) Methyl 2-(4-chloro-3-(perfluoropyridin-4-yl)phenyl)acetate

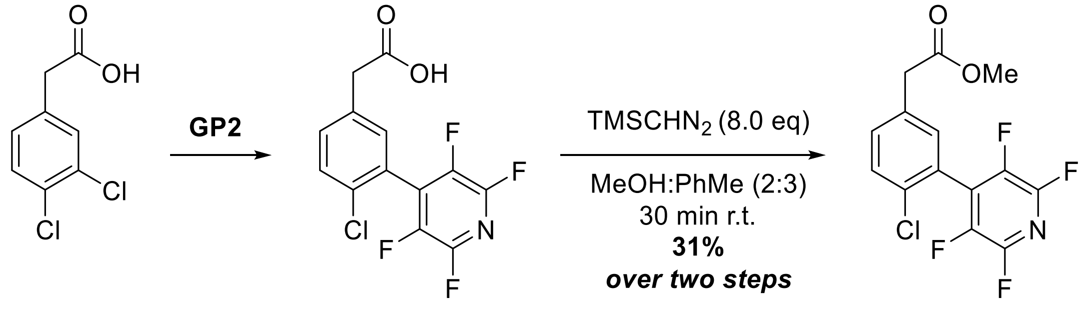

GP2 was followed with 2-(3,4-dichlorophenyl)acetic acid (41.0 mg, 0.20 mmol). The vial was then sealed, evacuated, and back filled with argon 3 times. The residue was then redissolved in PhMe:MeOH (3:2, 2.5 mL) and (Trimethylsilyl)diazomethane (2M in ether, 1.0 mL, 8 equiv.) added. The solution was stirred at room temperature for 30 minutes, then AcOH (0.1 mL) added to quench excess (Trimethylsilyl)diazomethane. The solvent was removed under reduced pressure, and analysis of the crude ^{1}H NMR showed a meta:para ratio of 12:1. The residue purified *via* column chromatography (eluting with CH_2Cl_2:hexanes (40:60)) to give the title compound (in a 12:1 Cl_m:Cl_p ratio) as an amorphous white solid (20.9 mg, 0.0628 mmol, 31%).

^{1}H NMR (400 MHz, $CDCl_3$) δ_H 7.54 (d, *J* = 8.3 Hz, 1H), 7.42 (dd, *J* = 8.3, 1.8 Hz, 1H), 7.27 (d, *J* = 1.8 Hz, 1H), 3.72 (s, 3H), 3.67 (s, 2H); **^{13}C NMR** (101 MHz, $CDCl_3$) δ_C 170.9, 143.5 (dm, *J* = 236.4 Hz), 139.4 (dm, *J* = 260.3 Hz), 133.4, 132.8, 132.4, 131.9, 131.3-131.0 (m), 130.3, 125.3 (br s), 52.3, 40.1; **^{19}F NMR**

(376 MHz, $CDCl_3$) δ_F -90.0 - -90.3 (m, 2F), -140.7 - -140.9 (m, 2F); **HRMS m/z:** $[M+H]^+$ calculated for $[C_{14}H_9ClF_4NO_2]^+$ 334.0252, found 334.0245.

As a control reaction, the above was carried out using SPhos as ligand:

(6m) Methyl 2-(3-chloro-4-(perfluoropyridin-4-yl)phenyl)acetate

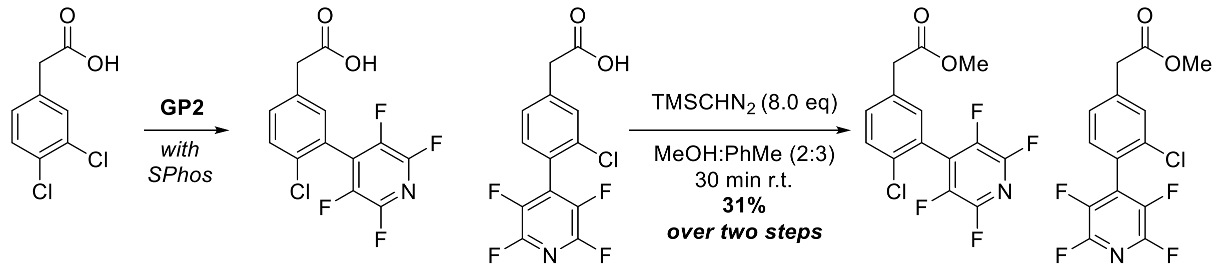

GP2 was followed with 2-(3,4-dichlorophenyl)acetic acid (41.0 mg, 0.20 mmol) and SPhos instead of $sSPhos_{(NBu4)}$. The vial was then sealed, evacuated, and back filled with argon 3 times. The residue was then redissolved in PhMe:MeOH (3:2, 2.5 mL) and (Trimethylsilyl)diazomethane (2M in ether, 1.0 mL, 8 equiv.) added. The solution was stirred at room temperature for 30 minutes, then AcOH (0.1 mL) added to quench excess (Trimethylsilyl)diazomethane. The solvent was removed under reduced pressure, and analysis of the crude ^{1}H NMR showed a meta:para ratio of 1:1. The residue was then purified *via* column chromatography (eluting with EtOAc:Hexanes (15:85) *then* the mixture was purified again eluting with CH_2Cl_2:hexanes (40:60)) to give the title compound (in a 1:2 Cl_m:Cl_p ratio) as an amorphous white solid (20.7 mg, 0.0622 mmol, 31%).

By deduction of the isolated meta product the para isomer can be described as followed: **^{1}H NMR** (400 MHz, $CDCl_3$) δ_H 7.53 (d, J = 1.8 Hz, 1H), 7.36 (dd, J = 7.9, 1.8 Hz, 1H), 7.30 (d, J = 7.9 Hz, 1H), 3.75 (s, 3H), 3.70 (s, 2H). **^{13}C NMR** (101 MHz, $CDCl_3$) δ_C 170.7, 143.5 (dm, J = 247.5 Hz), 139.6 (dm, J = 259.7 Hz), 138.2, 133.6, 131.3-130.9 (m), 131.1, 131.0, 128.2, 124.0 (br s), 52.4, 40.5. **^{19}F NMR** (376 MHz, $CDCl_3$) δ_F -90.1 - -90.3 (m, 2F), -140.8 - -140.9 (m, 2F). **HRMS m/z:** Not obtained due to this compound being isolated as a mixture with an isomer that has the same mass.

(5n) Methyl 2-(4-chloro-3-(perfluoropyridin-4-yl)phenyl)acetate

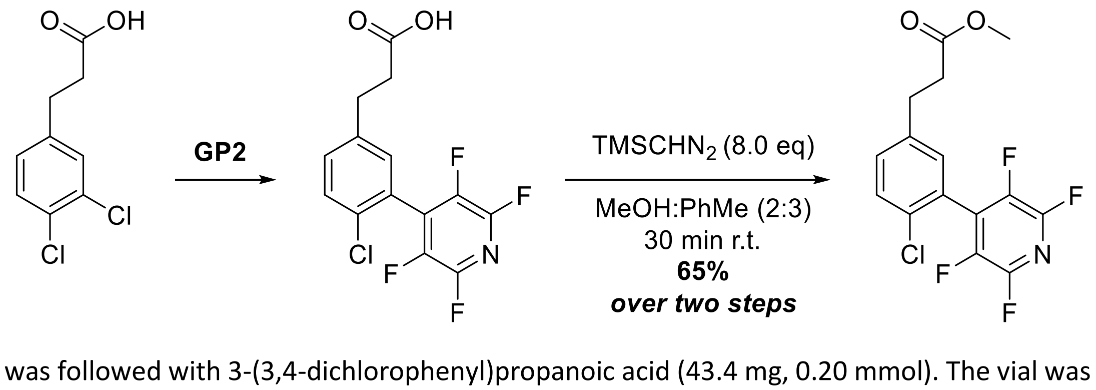

GP2 was followed with 3-(3,4-dichlorophenyl)propanoic acid (43.4 mg, 0.20 mmol). The vial was then sealed, evacuated, and back filled with argon 3 times. The residue was then redissolved in PhMe:MeOH (3:2, 2.5 mL) and (Trimethylsilyl)diazomethane (2M in ether, 1.0 mL, 8 equiv.) added. The solution was stirred at room temperature for 30 minutes, then AcOH (0.1 mL) added to quench excess (Trimethylsilyl)diazomethane. The solvent was removed under reduced pressure, and analysis of the crude ^{1}H NMR showed a meta:para ratio of 15:1. The residue purified *via* column chromatography (eluting with CH_2Cl_2:hexanes (40:60)) to give the title compound (in a 11:1 Cl_m:Cl_p ratio) as a colourless oil (47.1 mg, 0.133 mmol, 66%).

^{1}H NMR (400 MHz, $CDCl_3$) δ 7.49 (d, *J* = 8.3 Hz, 1H), 7.33 (dd, *J* = 8.3, 1.9 Hz, 1H), 7.18 (d, *J* = 1.9 Hz, 1H), 3.66 (s, 3H), 2.99 (t, *J* = 7.5 Hz, 2H), 2.66 (t, *J* = 7.5 Hz, 2H); **^{13}C NMR** (101 MHz, $CDCl_3$) δ 172.7, 143.5 (ddd, *J* = 246.1, 13.0, 2.9 Hz), 140.0, 139.6 (dm, *J* = 259.2 Hz) 131.8, 131.4 (tt, *J* = 16.8, 3.1 Hz), 131.3, 131.0, 130.2, 125.1, 51.8, 35.1, 30.0; **^{19}F NMR** (376 MHz, $CDCl_3$) δ -90.2 - -90.4 (m 2F), -140.9 – 141.1 (m, 2F); **HRMS m/z:** [M] calculated for [$C_{15}H_{10}ClF_4NO_2$] 347.0336, found 347.0343.

(5o) methyl 4-(4-chloro-3-(perfluoropyridin-4-yl)phenyl)butanoate

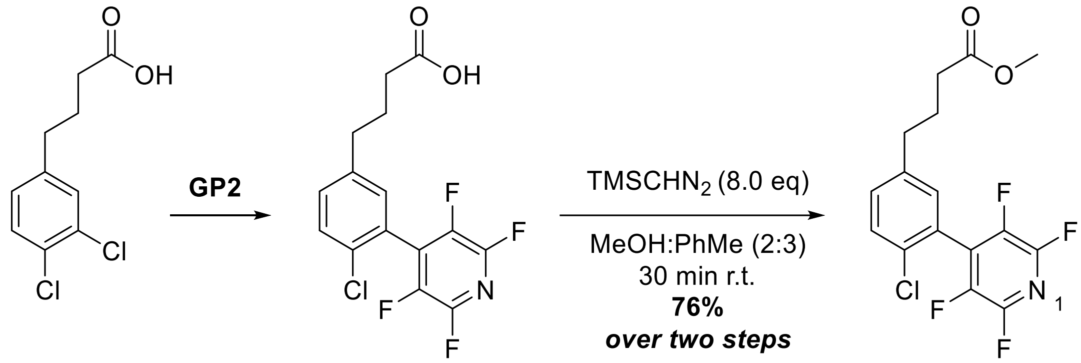

GP2 was followed with 4-(3,4-dichlorophenyl)butanoic acid (43.4 mg, 0.20 mmol). The vial was then sealed, evacuated, and back filled with argon 3 times. The residue was then redissolved in PhMe:MeOH (3:2, 2.5 mL) and (Trimethylsilyl)diazomethane (2M in ether, 1.0 mL, 8 equiv.) added. The solution was stirred at room temperature for 30 minutes, then AcOH (0.1 mL) added to quench excess (Trimethylsilyl)diazomethane. The solvent was removed under reduced pressure, and the residue was purified *via* column chromatography (eluting with CH_2Cl_2:hexanes (40:60)) to give the title compound (in a 8:1 Cl_m:Cl_p ratio) as a colourless oil (55.2 mg, 0.153 mmol, 76%).

^{1}H NMR (400 MHz, $CDCl_3$) δ_H 7.49 (d, *J* = 8.3 Hz, 1H), 7.31 (dd, *J* = 8.3, 1.9 Hz, 1H), 7.15 (d, *J* = 1.9 Hz, 1H), 3.67 (s, 3H), 2.70 (t, *J* = 7.7 Hz, 2H), 2.35 (t, *J* = 7.3 Hz, 2H), 1.97 (q, *J* = 6.0 Hz, 2H); **^{13}C NMR** (101 MHz, $CDCl_3$) δ_C 173.5, 143.5 (ddd, *J* = 245.9, 12.6, 2.8 Hz), 140.9, 139.3 (d m, *J* = 259.8 Hz), 131.9, 131.5 (tt, *J* = 17.0, 3.1 Hz), 130.97, 130.96, 130.1, 125.0, 51.6, 34.3, 33.1, 26.1; **^{19}F NMR** (376 MHz, $CDCl_3$) δ_F -90.3 - -90.5 (m, 2F), -140.9 - -141.1 (m, 2F); **HRMS m/z:** [M] calculated for [$C_{16}H_{12}ClF_4NO_2$] 361.0493, found 361.0492.

(5p) methyl (E)-4-(4-chloro-3-(perfluoropyridin-4-yl)phenyl)but-3-enoate

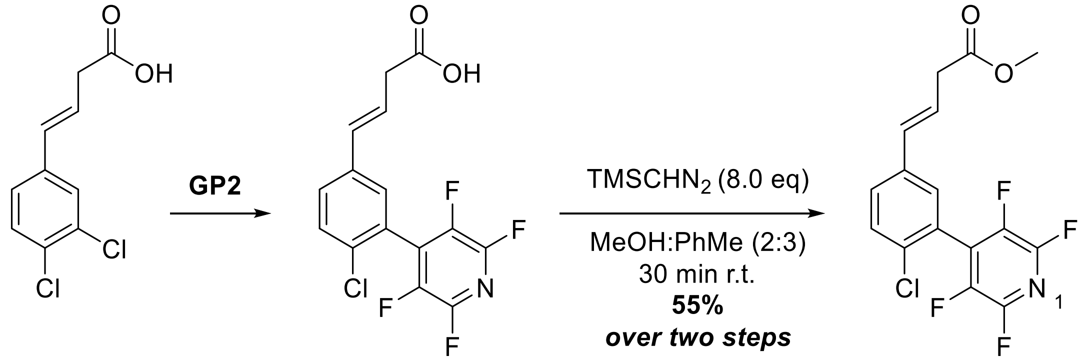

GP2 was followed with (E)-4-(4-chloro-3-(perfluoropyridin-4-yl)phenyl)but-3-enoic acid (45.8 mg, 0.20 mmol). The vial was then sealed, evacuated, and back filled with argon 3 times. The residue was then redissolved in PhMe:MeOH (3:2, 2.5 mL) and (Trimethylsilyl)diazomethane (2M in ether, 1.0 mL, 8 equiv.) added. The solution was stirred at room temperature for 30 minutes, then AcOH (0.1 mL) added to quench excess (Trimethylsilyl)diazomethane. The solvent was removed under reduced

pressure, and the residue was purified *via* column chromatography (eluting with CH_2Cl_2:hexanes (40:60)) to give the title compound (in a 12:1 Cl_m:Cl_p ratio) as a colourless oil (39.6 mg, 0.110 mmol, 55%).

^{1}H NMR (400 MHz, $CDCl_3$) δ_H 7.51 (d, *J* = 8.4 Hz, 1H), 7.48 (dd, *J* = 8.4, 1.8 Hz, 1H), 7.30 (d, *J* = 1.8 Hz, 1H), 6.48 (d, *J* = 15.8 Hz, 1H), 6.35 (dt, *J* = 16.0, 6.9 Hz, 1H), 3.72 (s, 3H), 3.28 (q, *J* = 2.7 Hz, 2H). **^{13}C NMR** (101 MHz, $CDCl_3$) δ_C 171.5, 143.5 (ddd, *J* = 245.9, 12.2, 2.3 Hz), 139.4 (dm, *J* = 261.2 Hz), 136.2, 132.2, 131.4-131.0 (m), 131.2, 130.3, 129.3, 128.7, 125.4, 124.3, 52.1, 38.0. **^{19}F NMR** (376 MHz, $CDCl_3$) δ_F -90.1 - -90.3 (m, 2F), -140.9 - -141.0 (m, 2F); **HRMS m/z:** [M] calculated for [$C_{16}H_9ClF_4NO_2$] 358.0258, found 358.0257.

9. N-oxides

(7) 2-(2-chloro-5-(((Trifluoromethyl)sulfonamido)methyl)phenyl)-4,5-dimethylthiazole 3-oxide

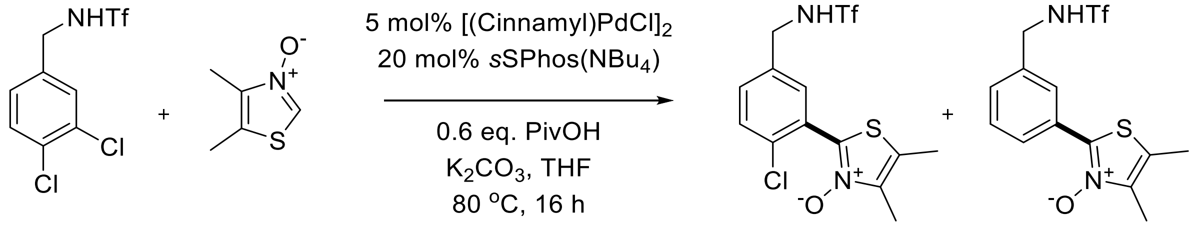

N-(3,4-dichlorobenzyl)-1,1,1-trifluoromethanesulfonamide (61.4 mg, 0.20 mmol, 1.0 eq.), sSPhos$_{(NBu4)}$ (29.2 mg, 0.04 mmol, 20 mol%), [(Cinnamyl)PdCl]$_2$ (5.2 mg, 0.01 mmol, 5 mol%), K_2CO_3 (82.9 mg, 0.6 mmol, 3.0 eq.) and 4,5-dimethylthiazole 3-oxide (32.3 mg, 0.25 mmol, 1.25 eq.) were added to a 4 mL 15x45 mm crimp top vial containing a stirrer bar. The vial was evacuated and back filled with argon 3 times, at which point THF (0.5 ml) was added and the top wrapped with parafilm. The reaction mixture was stirred for 4 h at 80 °C in a heating block and the solvent was removed under a stream of nitrogen. The residue was then purified *via* column chromatography (EtOAc) *then* (eluting with Acetone:Hexanes (30:70)) to give a set of fractions of the title compound as a colourless oil (13.1 mg, 0.0328 mmol, 16%) and an additional set of fractions that contained the title product with the hydro-dechlorinated product in a 7:1 ratio (*isolated mass* 12.1 mg, *corrected mass* 10.7 mg, 0.0268 mmol, 14%). Overall yield (23.8 mg, 0.596 mmol, 30%).

^{1}H NMR (400 MHz, $CDCl_3$) δ_H 9.54 (br s, 1H), 7.69 (d, *J* = 2.1 Hz, 1H), 7.37 (d, *J* = 8.4 Hz, 1H), 7.26 (dd, *J* = 8.4, 2.1 Hz, 1H), 4.14 (s, 2H), 2.46 (d, *J* = 0.7 Hz, 3H), 2.36 (d, *J* = 0.7 Hz, 3H); **^{13}C NMR** (101 MHz, $CDCl_3$) δ_C 141.5, 139.0, 135.8, 132.5, 130.5, 130.2, 129.8, 125.9, 125.3, 119.2 (q, *J* = 322.9 Hz), 46.0, 13.0, 11.0; **^{19}F NMR** (376 MHz, $CDCl_3$) δ_F -77.4 (s, 3F); **HRMS m/z:** $[M-H]^-$ $[C_{13}H_{11}ClF_3N_2O_3S_2]^-$ 398.9857, found 398.9861.

(8) 2-(5-((N-butylsulfamoyl)methyl)-2-chlorophenyl)pyrazine 1-oxide

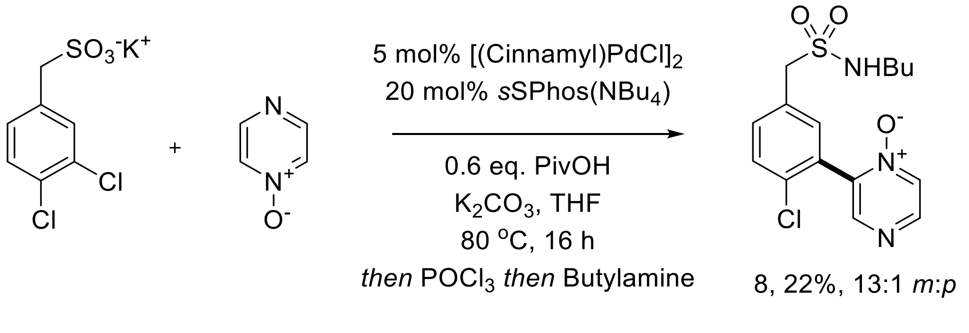

8, 22%, 13:1 *m*:*p*

Potassium (3,4-dichlorophenyl)methanesulfonate (53.0 mg, 0.20 mmol), sSPhos$_{(NBu4)}$ (29.2 mg, 0.04 mmol, 20 mol%), [(Cinnamyl)PdCl]$_2$ (5.2 mg, 0.01 mmol, 5 mol%), K_2CO_3 (82.9 mg, 0.6 mmol, 3.0 eq.) and pyrazine 1-oxide (38.4 mg, 0.4 mmol, 2.0 eq.) were added to a 4 mL 15x45 mm crimp top vial containing a stirrer bar. The vial was evacuated and back filled with argon 3 times, at which point THF (0.5 ml) was added and the top wrapped with parafilm. The reaction mixture was stirred for 16 h at 80 °C in a heating block and the solvent was removed and the vial sealed, evacuated and then back filled with argon 3 times. MeCN (1.0 ml) was added to the residue followed by the dropwise addition of $POCl_3$ (0.19 ml, 2.0 mmol, 10 eq.) (C*aution! The remaining K_2CO_3 present in the reaction mixture results in a considerable release of gas*). The resulting mixture was stirred at 55 °C for 2 hours. The

volatiles were then removed under a stream of nitrogen and H_2O and CH_2Cl_2 (2 ml) were added. The layers were separated and the aqueous layer was extracted with CH_2Cl_2 (3 x 2 ml). The combined organics were dried over $MgSO_4$, filtered and concentrated *in vacuo*. The sulfonyl chloride was then redissolved in CH_2Cl_2 (2.0 ml) and cooled to 0 ^oC under argon at which point butylamine (0.03 ml, 0.3 mmol, 1.5 eq.) was added dropwise. After 10 minutes, triethylamine (0.05 ml, 0.4 mmol, 2.0 eq.) was added dropwise and left to stir at room temperature for 16 h. The solvent was removed under reduced pressure and the residue was purified *via* column chromatography 4 times (eluting with EtOAc *then* Acetone:hexanes (30:70) *then* CH_2Cl_2:MeOH (95:5) *then* CH_2Cl_2:EtOAc (50:50) to give the title compound (in a 13:1 Cl_m:Cl_p ratio) as a colourless oil (15.8 mg, 0.0445, 22%).

^{1}H NMR (400 MHz, $CDCl_3$) δ_H 8.56 (s, 1H), 8.49 (d, *J* = 4.1 Hz, 1H), 8.22 (dd, *J* = 4.1, 0.6 Hz, 1H), 7.57 (d, *J* = 9.0 Hz, 1H), 7.51-7.49 (m, 2H), 4.38 (t, *J* = 6.0 Hz, 2H), 4.25 (s, 2H), 3.05 (q, *J* = 6.1 Hz, 2H), 1.50 (quin, *J* = 7.3 Hz, 2H), 1.33 (sext, *J* = 7.2 Hz, 2H), 0.90 (t, *J* = 7.3 Hz, 3H); **^{13}C NMR** (101 MHz, $CDCl_3$) δ_C 149.2, 147.1, 142.8, 135.0, 134.2, 133.7, 133.5, 130.4, 128.9, 128.9, 57.6, 43.5, 32.4, 19.7, 13.6 **HRMS m/z:** $[M-H]^-$ $[C_{15}H_{19}ClN_3O_3S]^-$ 356.0836, fond 356.0822.

10. Crown ether experiments

Crown ether experiments

General procedure **1** was followed with addition of the respective crown ether which was added before the vial was backfilled with argon. Reactions were assessed by analysing the crude ^{1}H NMR with reference to dimethoxymethane as an internal standard.

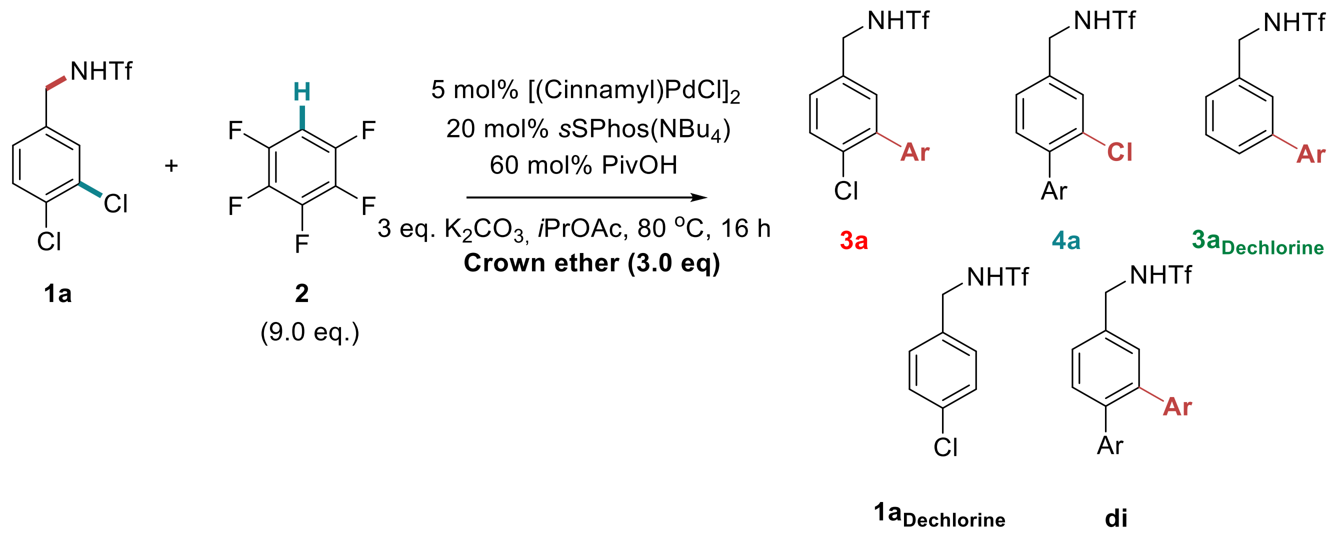

Entry	Crown ether	% 3a	% 4a	% $3a_{Dechlorine}$	% $1a_{Dechlorine}$	di	% SM	% conv	comments
1	12-4	45	4	1	13	-	20	**76**	30 min reaction time
2	15-5	11	6	-	8	-	66	**31**	
3	18-6	6	6	-	6	-	75	**19**	

Yields determined by ^{1}H-NMR with dimethoxyethane (0.033 mmol) as internal standard

11. BF_3K salts and Mida boronate ester

In our previous publication we observed no conversion to product when trying to perform a Suzuki reaction with 1,3,5-trifluorobenzene boronic acid.[1] Here we have also evaluated related BF_3K salts and MIDA protected boronate esters under slow release conditions as developed by Burke[13] and Molander[15] to see whether these can be effective.

Burke conditions for MIDA boronates

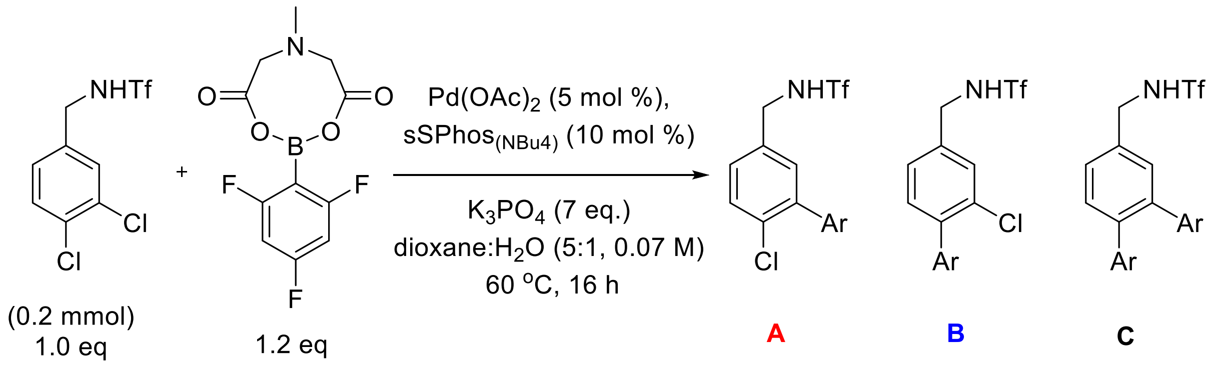

Entry	% A	% B	% C	A:B	% SM	% conv
1	0	0	0	N/A	93	**0**

Yields determined by ^{1}H-NMR with dimethoxyethane (0.033 mmol) as internal standard

Optimised Molander conditions for BF_3K salts

Pentafluorophenyltrifluoroborate performed particularly sluggish (38% isolated yield) under Molander's published conditions for aryl- and heteroaryltrifluoroborates.[14] More recently, Parmon published more efficient optimised conditions for pentafluorophenyltrifluoroborate which were used instead.[16]

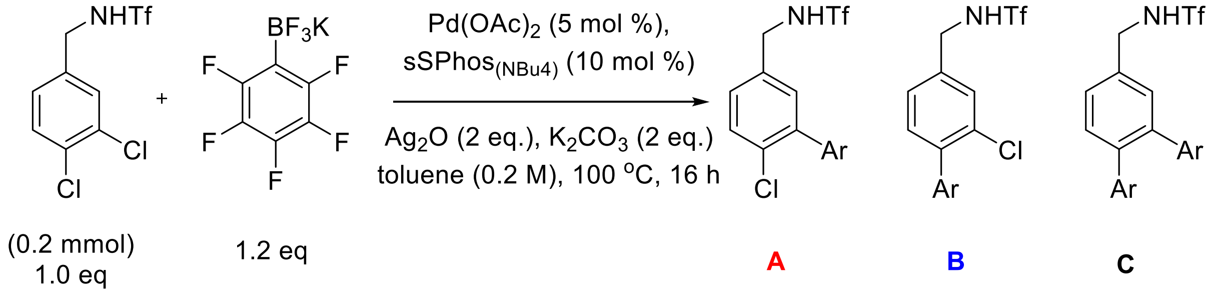

Entry	% A	% B	% C	A:B	% SM	% conv
1	0	0	0	N/A	93	**0**

Yields determined by ^{1}H-NMR with dimethoxyethane (0.033 mmol) as internal standard

"Suzuki conditions"

Even though the published conditions showed no conversion we tested both boron species under our system for cross coupling, however we could not observe product formation wither with sSPhos/Pd(OAc)$_2$ or sSPhosPdG2 (Aldrich 763314).

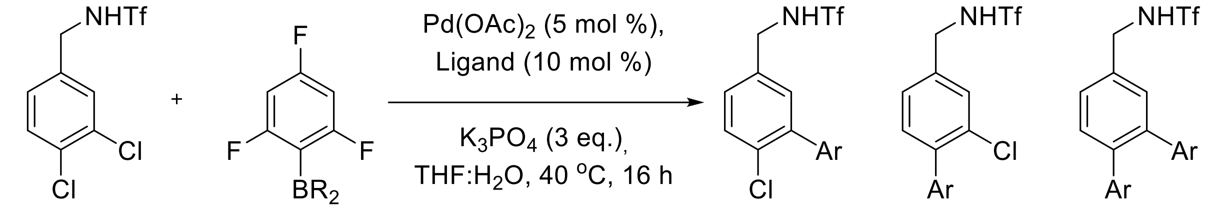

(0.2 mmol)
1.0 eq

A B C

Entry	Boronic acid	Ligand	% A	% B	% C	A:B	% SM	% conv
1	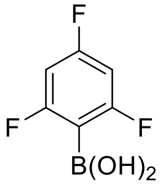	**sSPhos**	0	0	0	N/A	100	**0**
2		**sSPhos Pd G2**	0	0	0	N/A	100	**0**
3	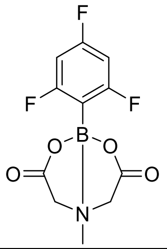	**sSPhos**	0	0	0	N/A	100	**0**
4		**sSPhos Pd G2**	0	0	0	N/A	100	**0**
5	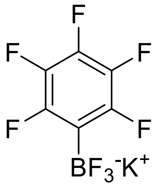	**sSPhos**	0	0	0	N/A	100	**0**
6		**sSPhos Pd G2**	0	0	0	N/A	96	**0**

Yields determined by ^{1}H-NMR with dimethoxyethane (0.033 mmol) as internal standard

12. References

[1] W. A. Golding, R. Pearce-Higgins, R. J. Phipps, *J. Am. Chem. Soc.* **2018**, 140, 13570-13574

[2] D. Türp, M. Wagner, V. Enkelmann, K. Müllen. *Angew. Chem. Int. Ed,* **2011**, 50, 4962-4965

[3] K. Kikushima, M. Grellier, M. Ohashi, S. Ogoshi, *Angew. Chem. Int. Ed.* **2017**, 56, 16191-16196

[4] M. C. Van Zandt, M. L. Jones, D. E. Gunn, L. S. Geraci, J. H. Jones, D. R. Sawicki, J. Sredy, J. L. Jacot, A. T. DiCioccio, T. Petrova, A. Mitschler, A. D. Podjarny, *J. Med. Chem.* **2005**, 48, 3141-3152.

[5] A. B. Theis, C. A. Townsend, *Synth. Commun,* **2006**, 157-166

[6] Y. Sakamoto, T. Suzuki, A. Miura, H. Fujikawa, Y. Taga, *J. Am. Chem. Soc.* **2000,** 122, 1832-1833

[7] K. S. Narayanan, K. D. Berlin, *J. Org. Chem.* **1980**, *45*, 2240

[8] H. M. L. Davies, D. G. Stafford, T. Hansen, *Org. Lett,* **1999**, 1, 233-236

[9] A. Stößel, M. Schlenck, S. Hinz, P. Küppers, J. Heer, M. Gütschow, C. E. Müller, *J. Med. Chem,* **2013,** 56, 4580-4596

[10] J. Vercouillie, M. Abarbri, J-L. Parrain, A. Duchene, J. Thibonnet, *Synth. Comm,* **2004**, 34, 3751-3762

[10] M. G. N. Russel, R. W. Carling, J. R. Atack, F. A. Bromidge, S. M. Cook, P. Hunt, C. Isted, M. Lucas, R. M. McKernan, A. Mitchinson, K. W. Moore, R. Narquizian, A. J. Macaulay, D. Thomas, S-A. Thompson, K. A. Wafford, J. L. Casto, *J. Med. Chem*. **2005**, 48, 1367-1383

[11] J. P. Leclerc, K. Fagnou, *Angew. Chem. Int. Ed.* **2006**, 7781-7786

[12] A. Greenfield, C. Grosanu, *Tett. Lett.* **2008,** 49, 6300-6303

[13] D. M. Knapp, E. P. Gillis, M. D. Burke, *J. Am. Chem. Soc,* **2009**, 131, 6961-6963

[14] S-C. Tso, M. Lou, C-Y. Wu, W-J. Gui, J. L. Chuang, L. K. Morlock, N. S. Williams, R. M. Wynn, X. Qi, D. T. Chuang, *J. Med. Chem,* **2017**, 60, 1142-1150

[15] G. A. Molander, B. Biolatto, *J. Org. Chem,* **2003**, 68, 4302-4314

[16] A. Y. Shabalin, A. Y. Adonin, V. V. Bardin, V. N. Parmon, *Tetrahedron*, **2014**, 70, 3720-3725

13. NMR spectra

Synthesis of ligands

^{1}H NMR (600 MHZ, MeOD) sodium 2'-(dicyclohexylphosphaneyl)-2,6-diisopropyl-[1,1'-biphenyl]-4-sulfonate **(sSPhos)**

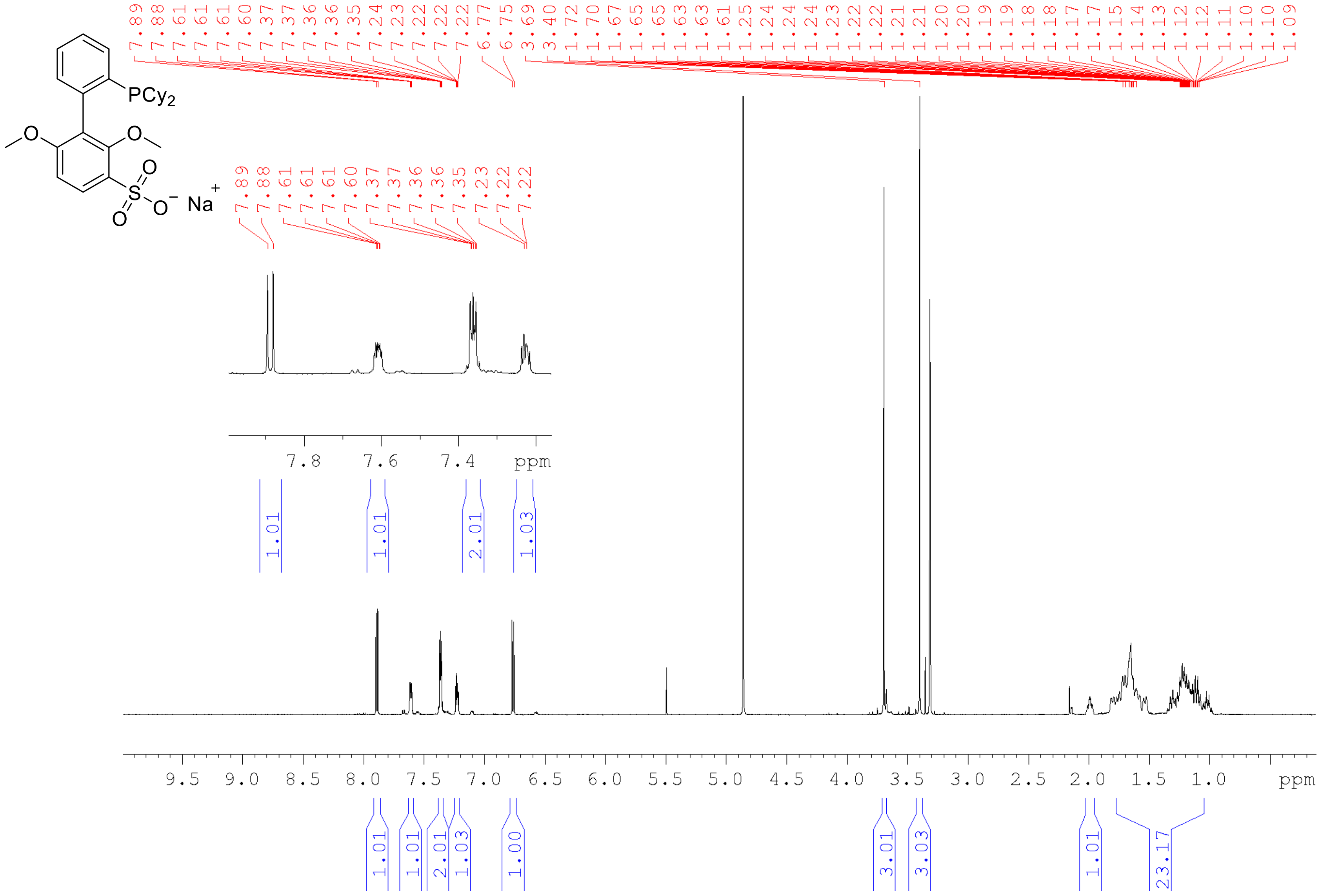

^{13}C NMR (101 MHZ, MeOD) sodium 2'-(dicyclohexylphosphaneyl)-2,6-diisopropyl-[1,1'-biphenyl]-4-sulfonate **(sSPhos)**

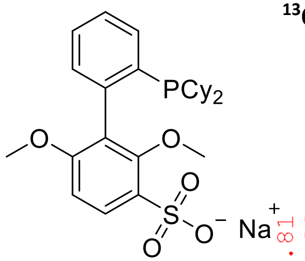

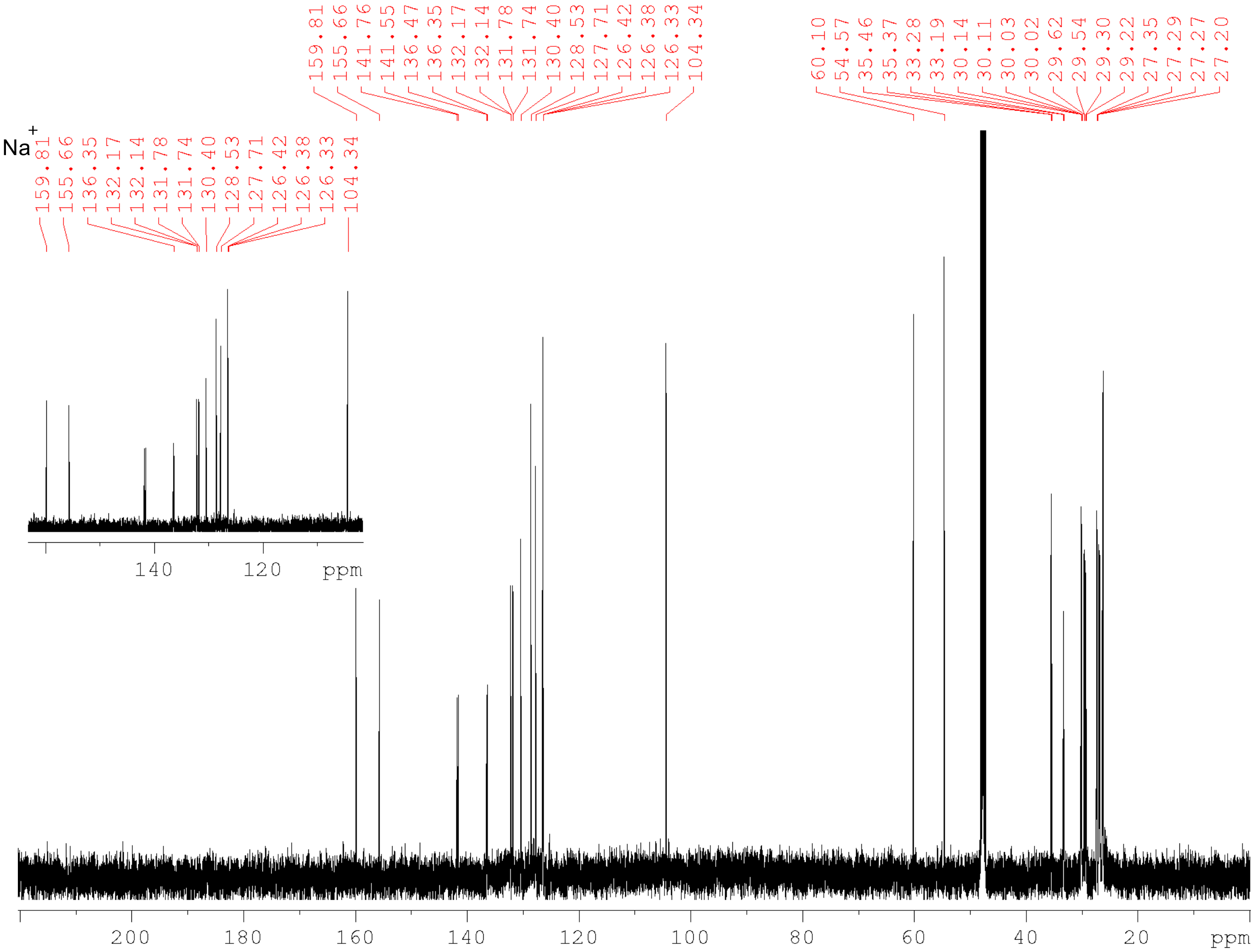

^{31}P NMR (162 MHZ, MeOD) sodium 2'-(dicyclohexylphosphaneyl)-2,6-diisopropyl-[1,1'-biphenyl]-4-sulfonate **(sSPhos)**

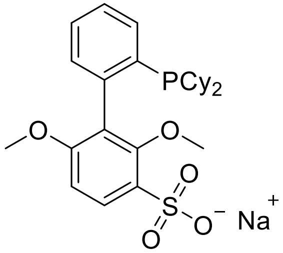

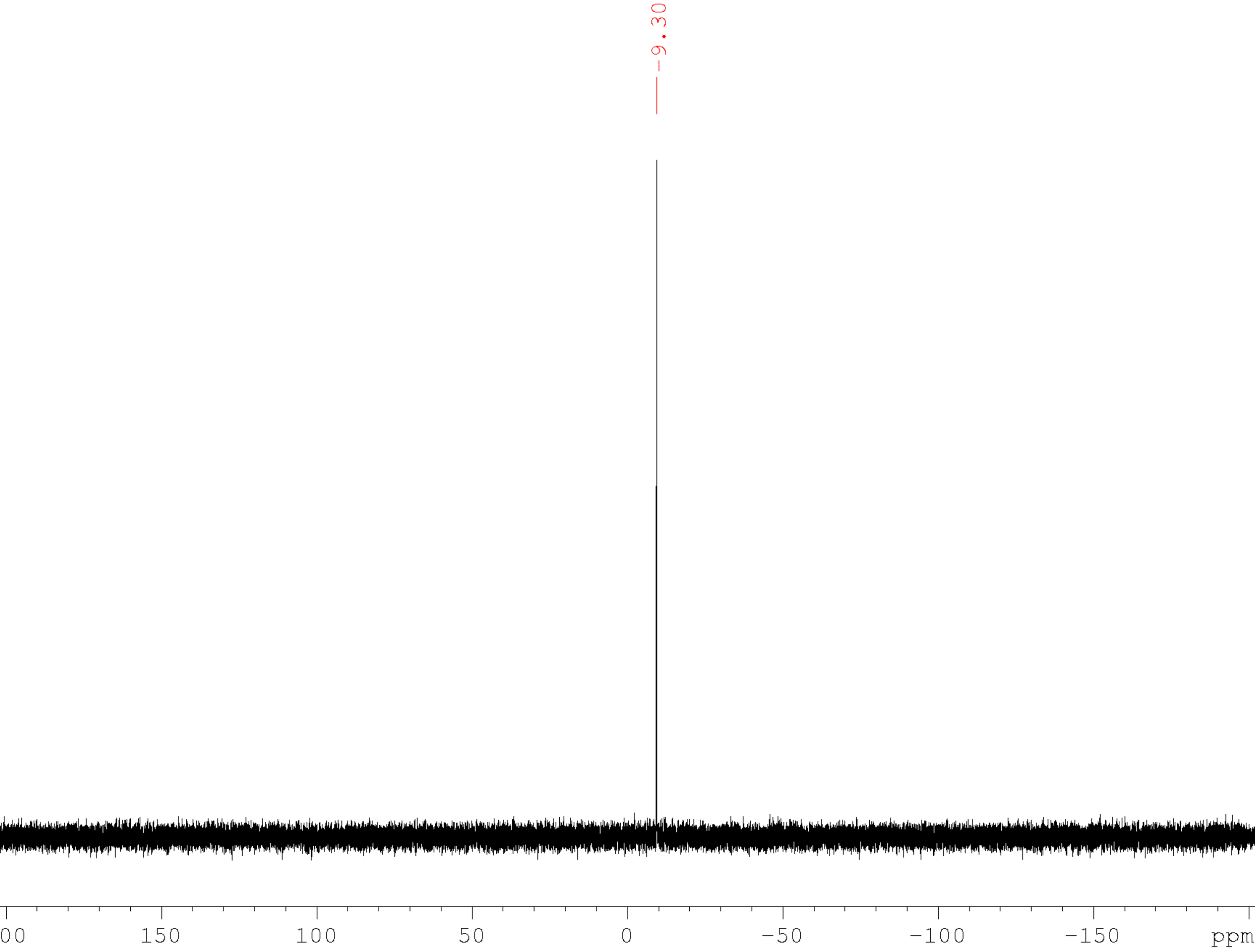

^{1}H NMR (400 MHZ, $CDCl_3$) tetrabutylammonium 2'-(dicyclohexylphosphaneyl)-2,6-diisopropyl-[1,1'-biphenyl]-4-sulfonate **(sSPhos$_{NBu4}$)**

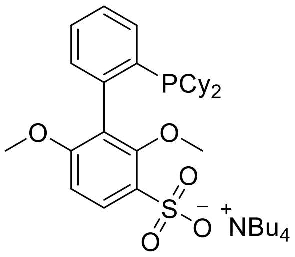

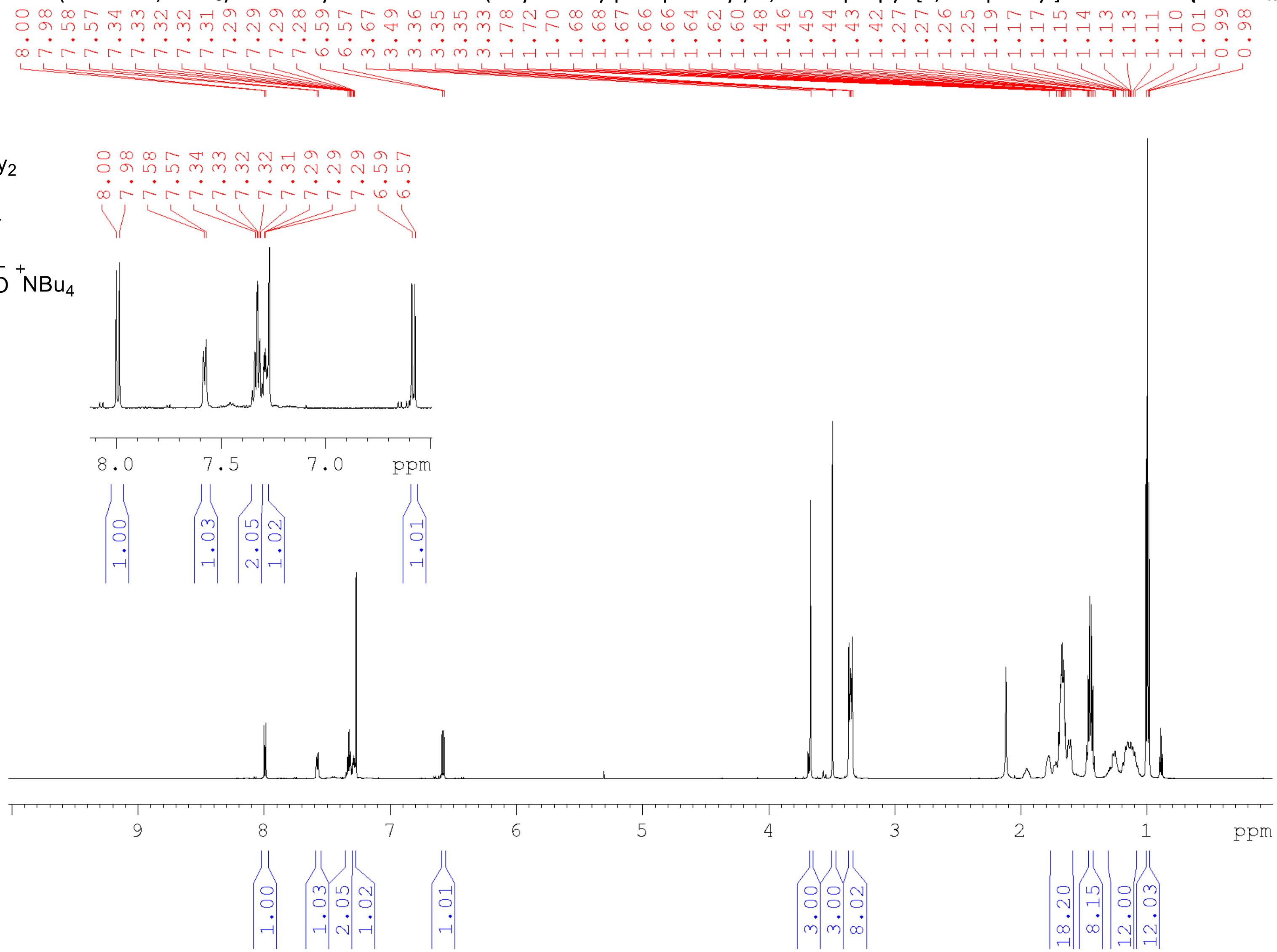

^{13}C NMR (151 MHZ, $CDCl_3$, *phosphorous decoupled*) tetrabutylammonium 2'-(dicyclohexylphosphaneyl)-2,6-diisopropyl-[1,1'-biphenyl]-4-sulfonate **(sSPhos$_{NBu4}$)**

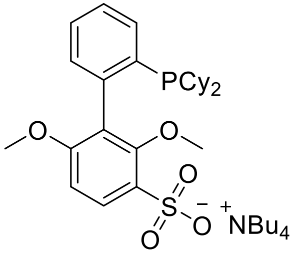

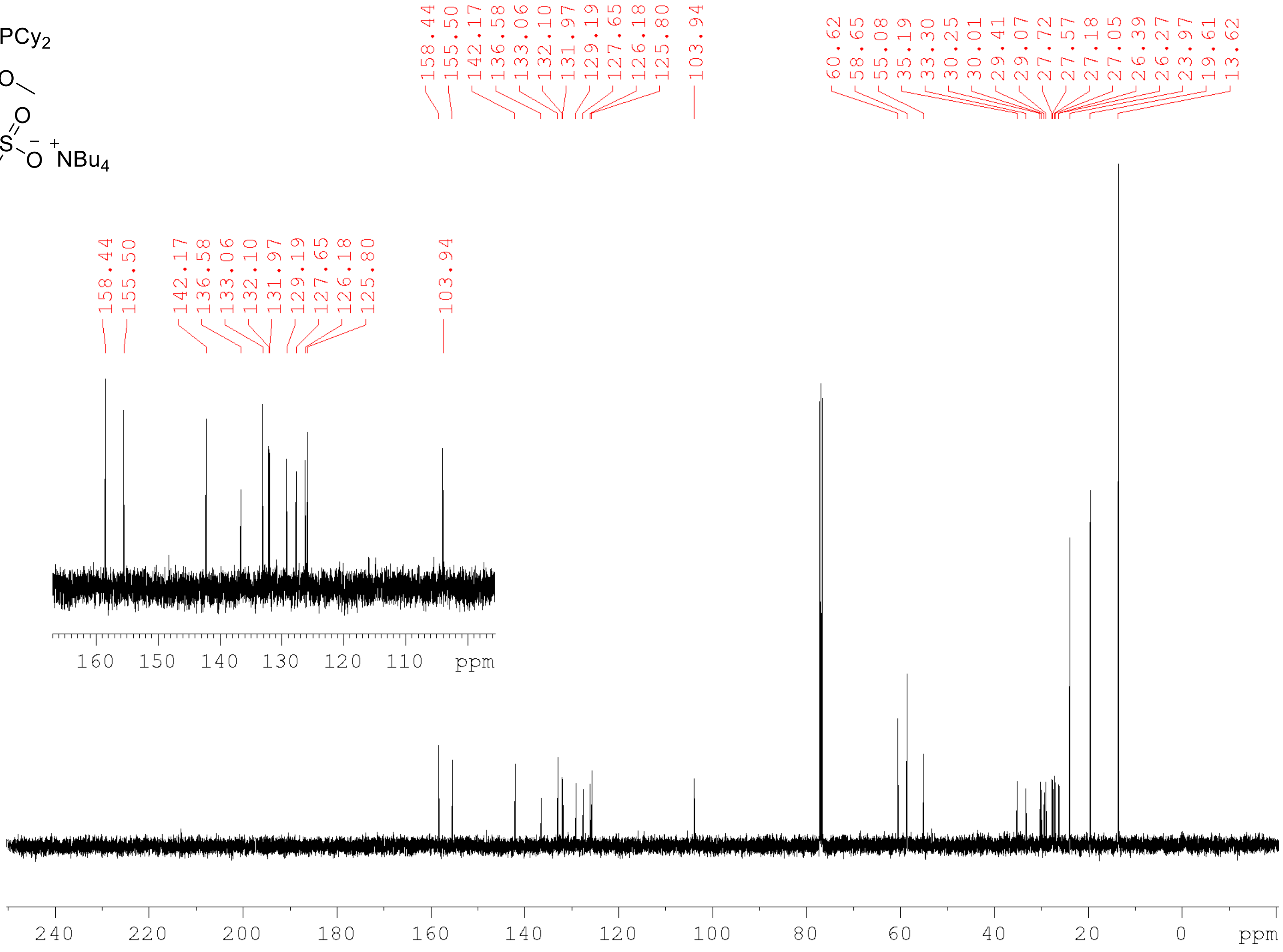

^{31}P NMR (162 MHZ, MeOD) tetrabutylammonium 2'-(dicyclohexylphosphaneyl)-2,6-diisopropyl-[1,1'-biphenyl]-4-sulfonate **(sSPhos$_{NBu4}$)**

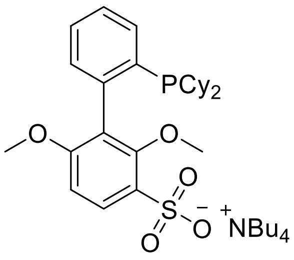

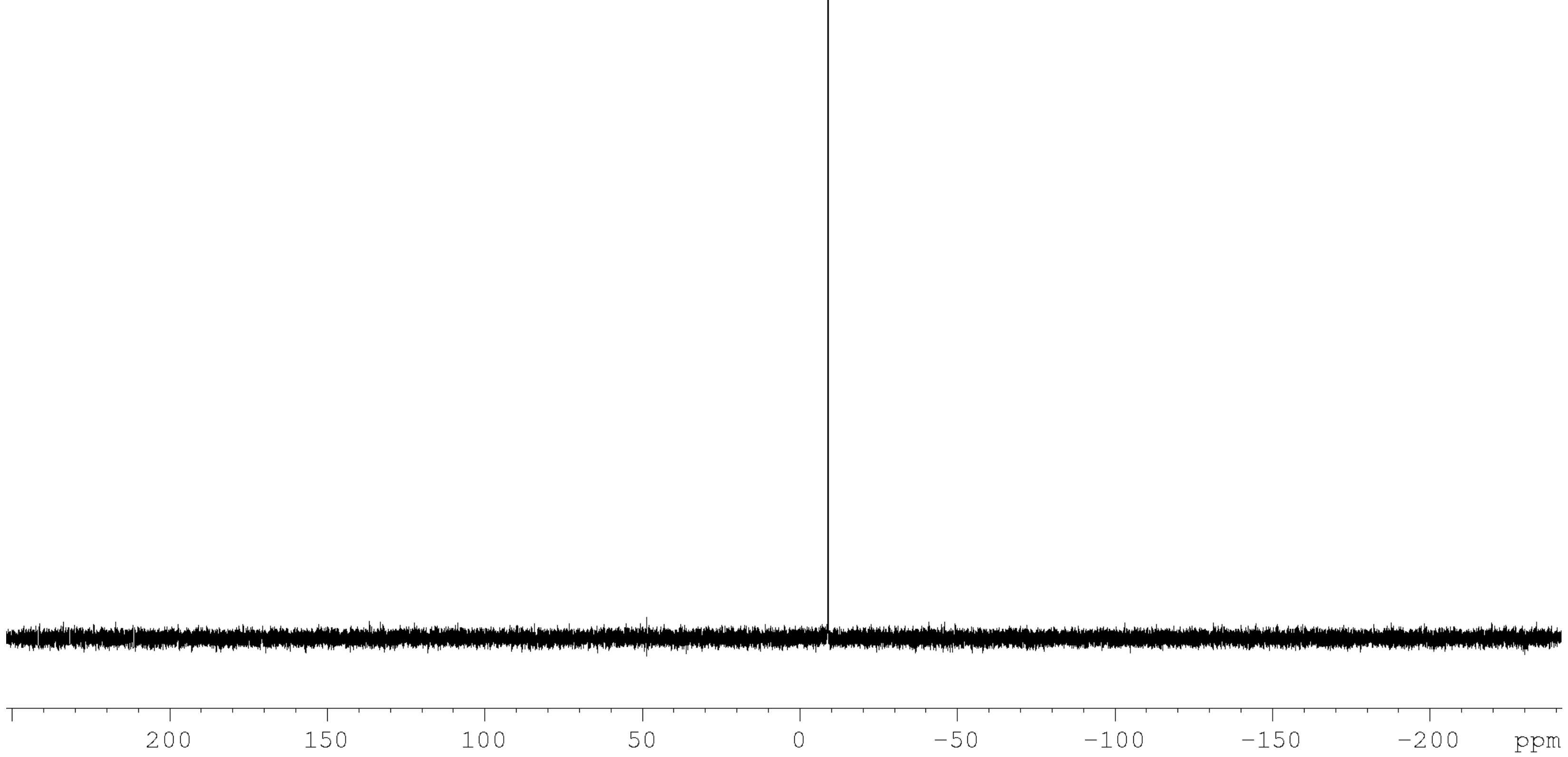

Synthesis of starting material

^{1}H NMR (600 MHZ, $CDCl_3$) **(1a)** *N*-(3,4-dichlorobenzyl)-1,1,1-trifluoromethanesulfonamide

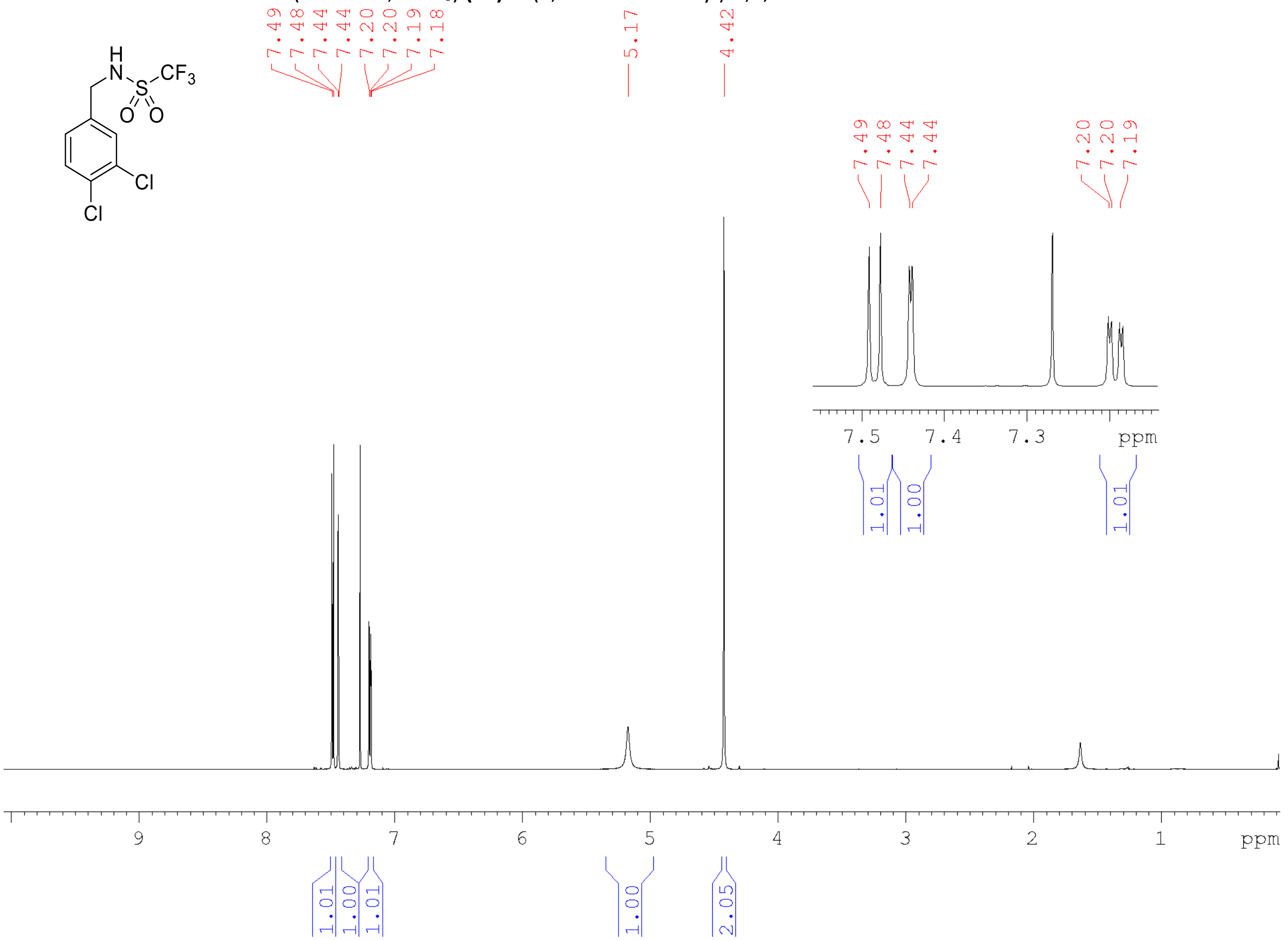

^{13}C NMR (151 MHz, $CDCl_3$) **(1a)** *N*-(3,4-dichlorobenzyl)-1,1,1-trifluoromethanesulfonamide

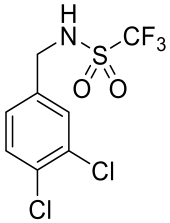

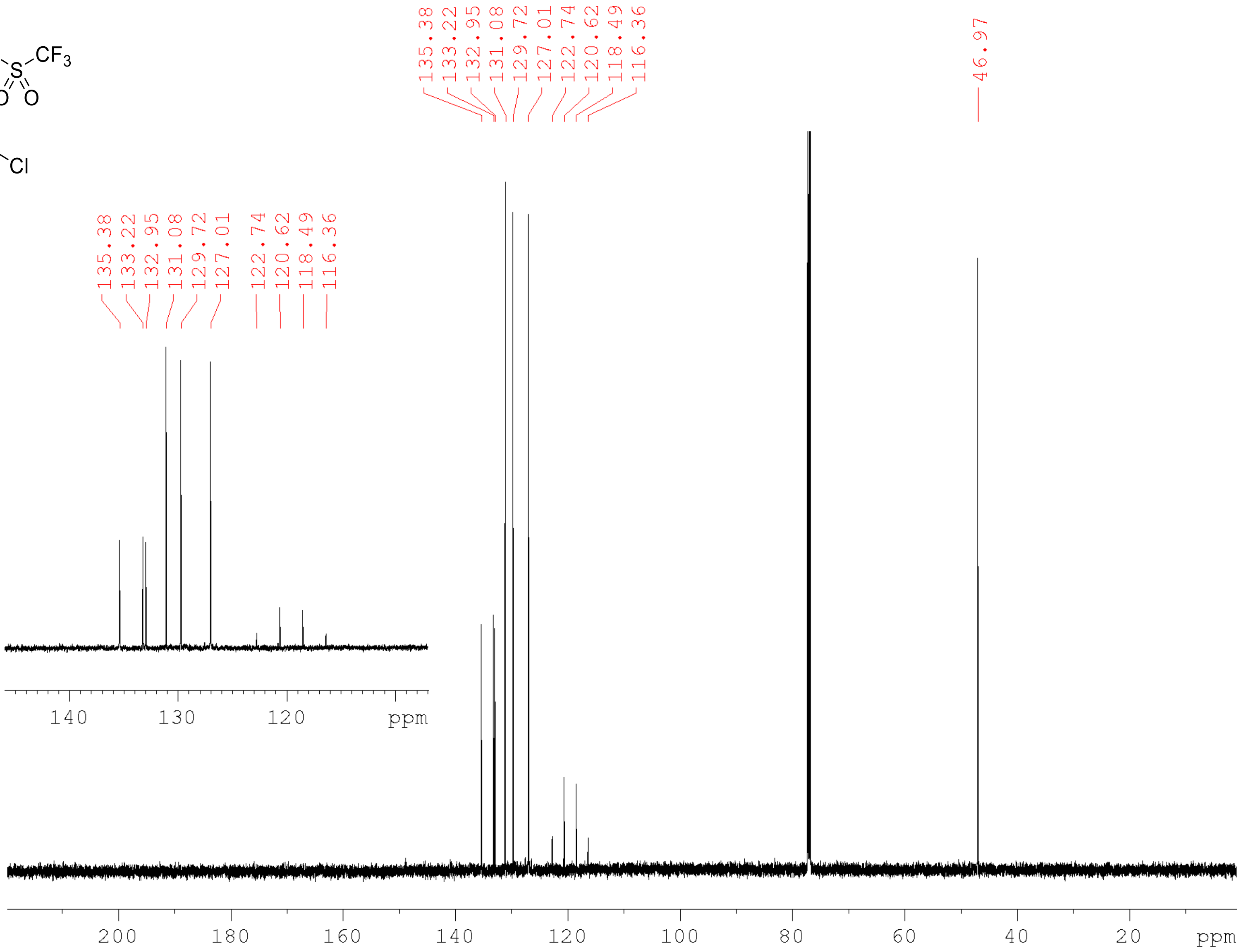

^{19}**F NMR** (376 MHZ, $CDCl_3$) **(1a)** *N*-(3,4-dichlorobenzyl)-1,1,1-trifluoromethanesulfonamide

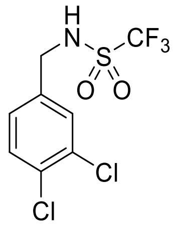

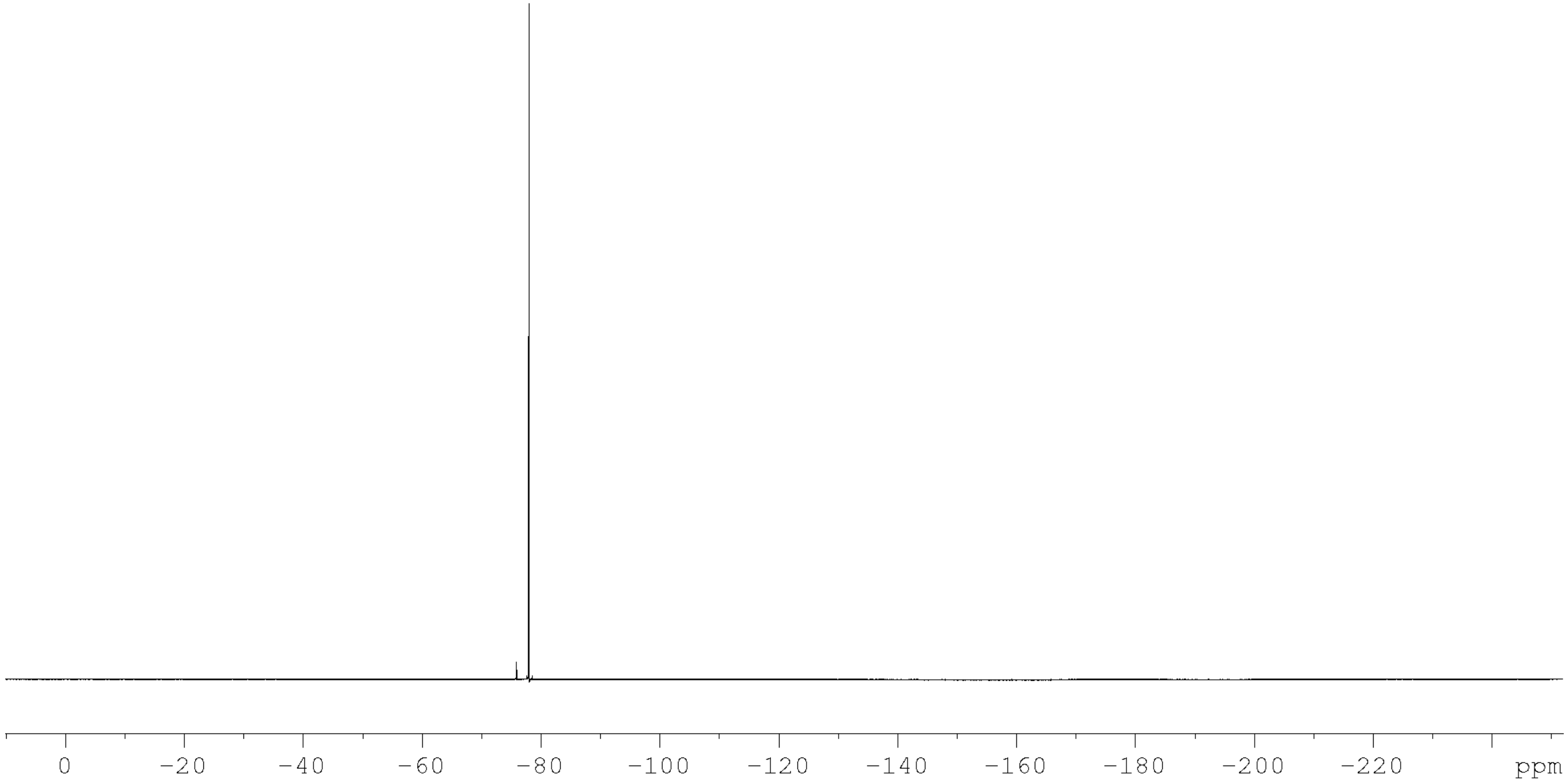

^{1}H NMR (400 MHZ, $CDCl_3$) triisopropyl((2,3,5,6-tetrafluorophenyl)ethynyl)silane

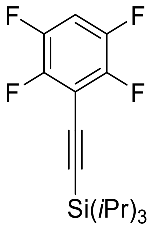

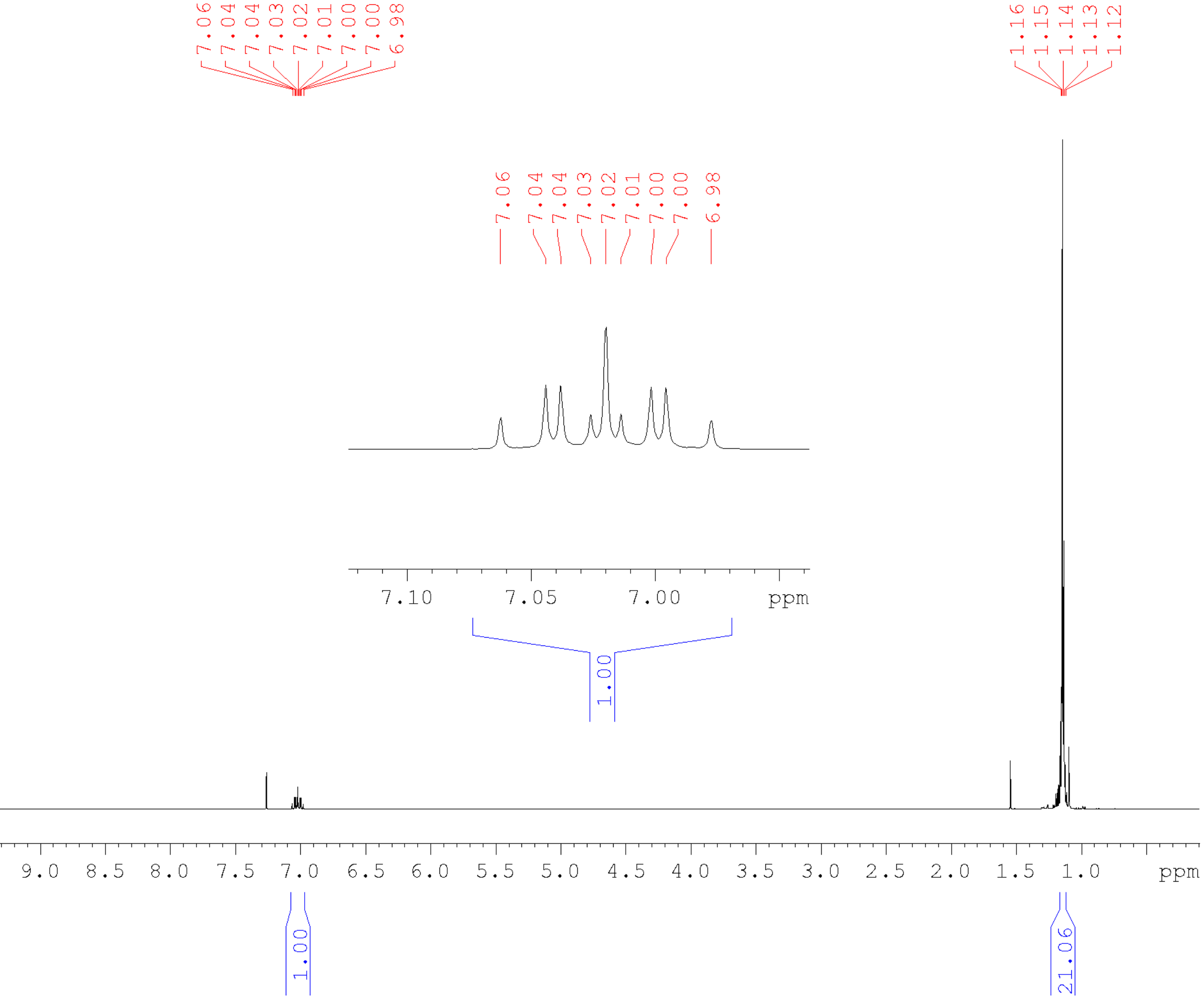

^{13}**C NMR** (101 MHZ, $CDCl_3$) triisopropyl((2,3,5,6-tetrafluorophenyl)ethynyl)silane

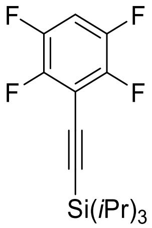

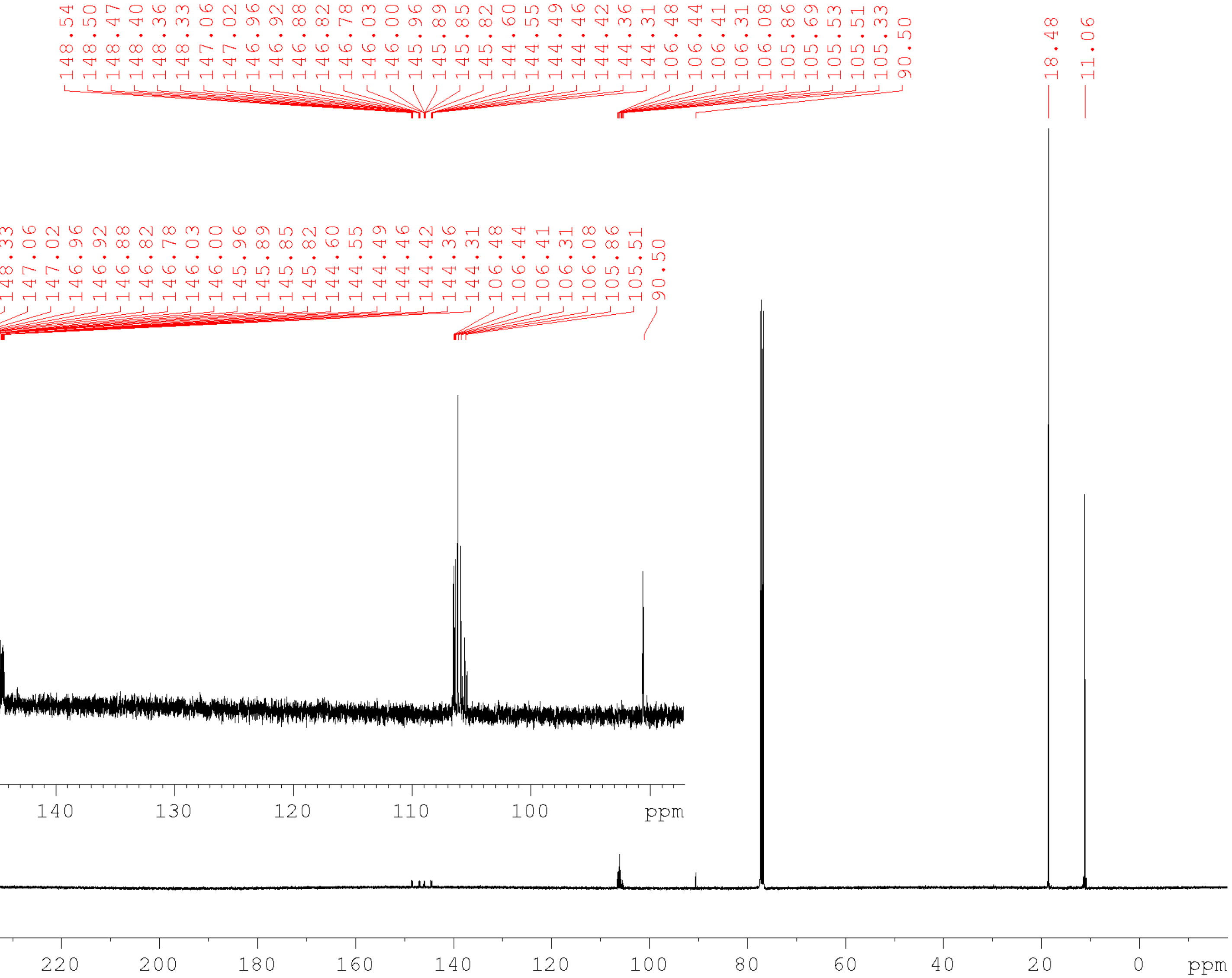

^{19}F NMR (376 MHZ, $CDCl_3$) triisopropyl((2,3,5,6-tetrafluorophenyl)ethynyl)silane

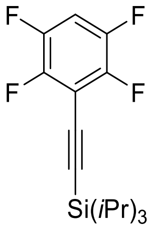

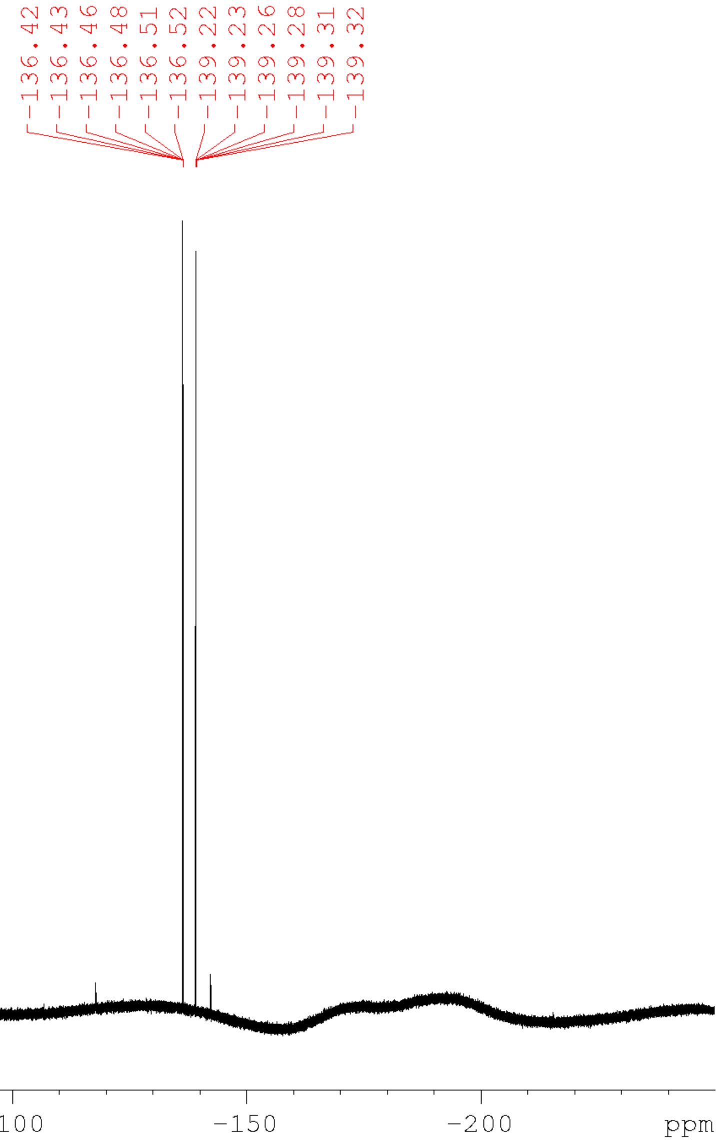

¹H NMR (600 MHZ, $CDCl_3$) methyl 2,3,5,6-tetrafluorobenzoate

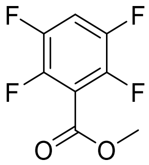

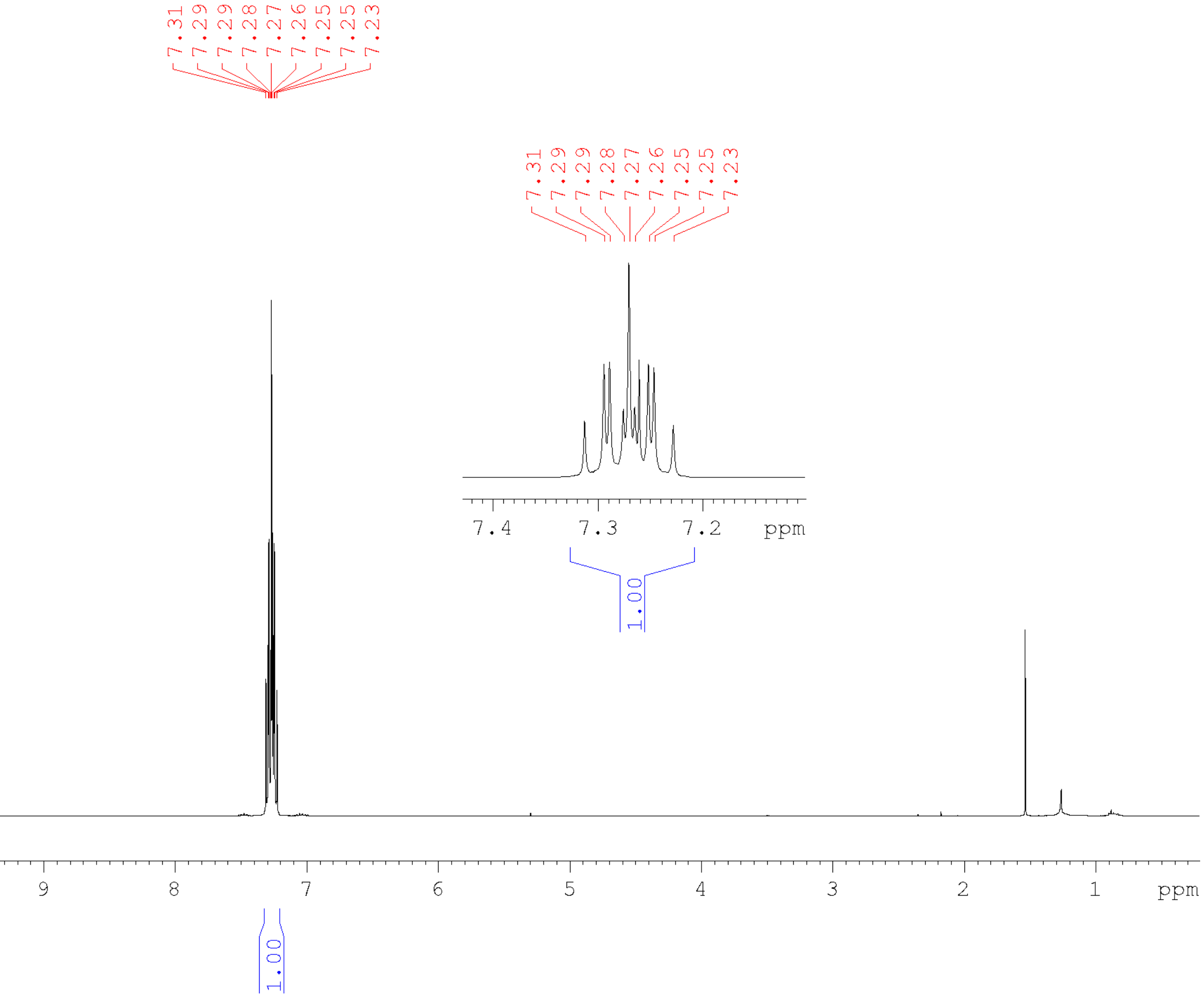

^{13}C NMR (151 MHZ, $CDCl_3$) methyl 2,3,5,6-tetrafluorobenzoate

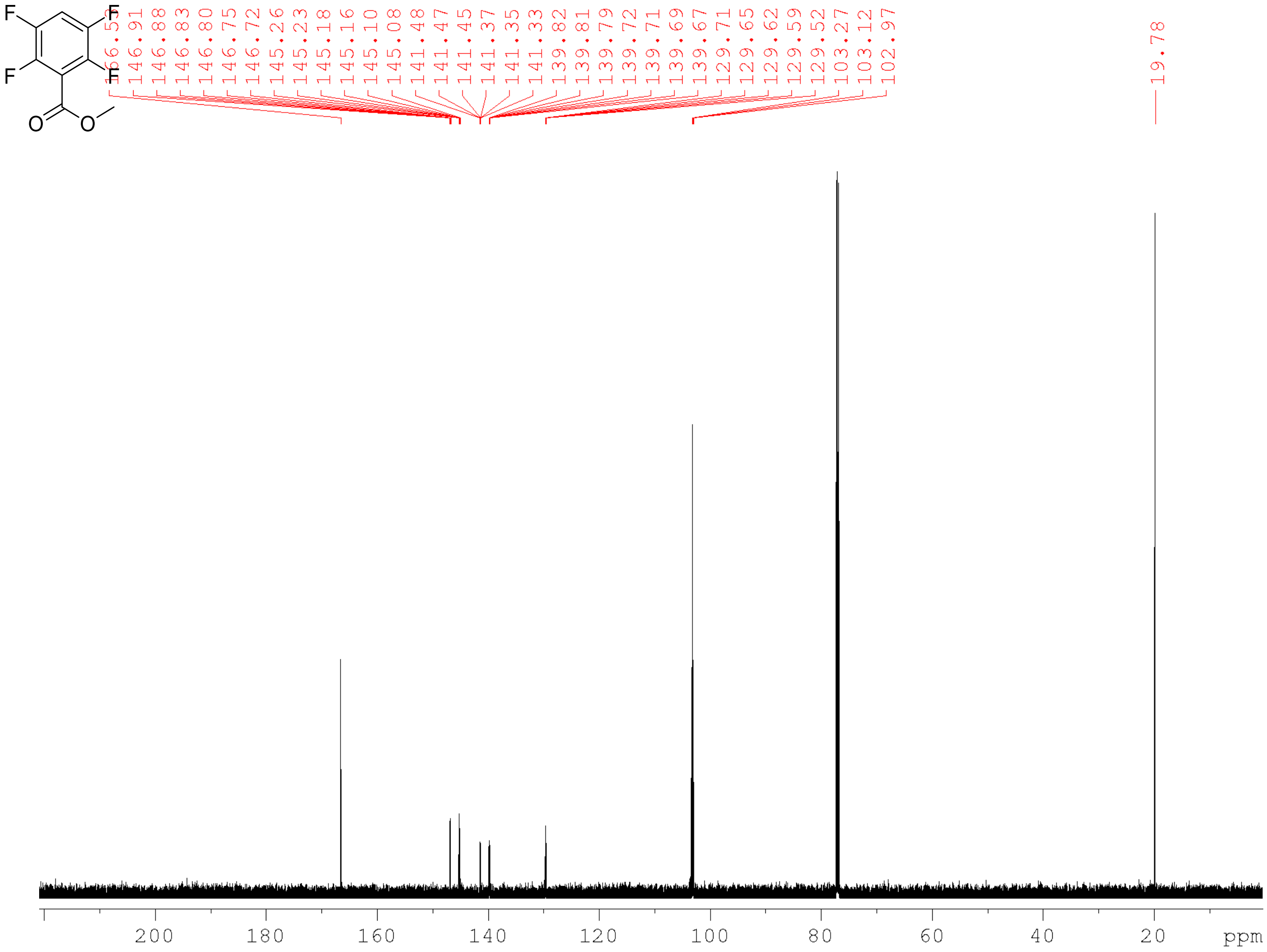

^{19}F NMR (376 MHZ, $CDCl_3$) methyl 2,3,5,6-tetrafluorobenzoate

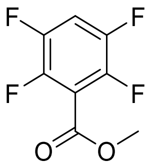

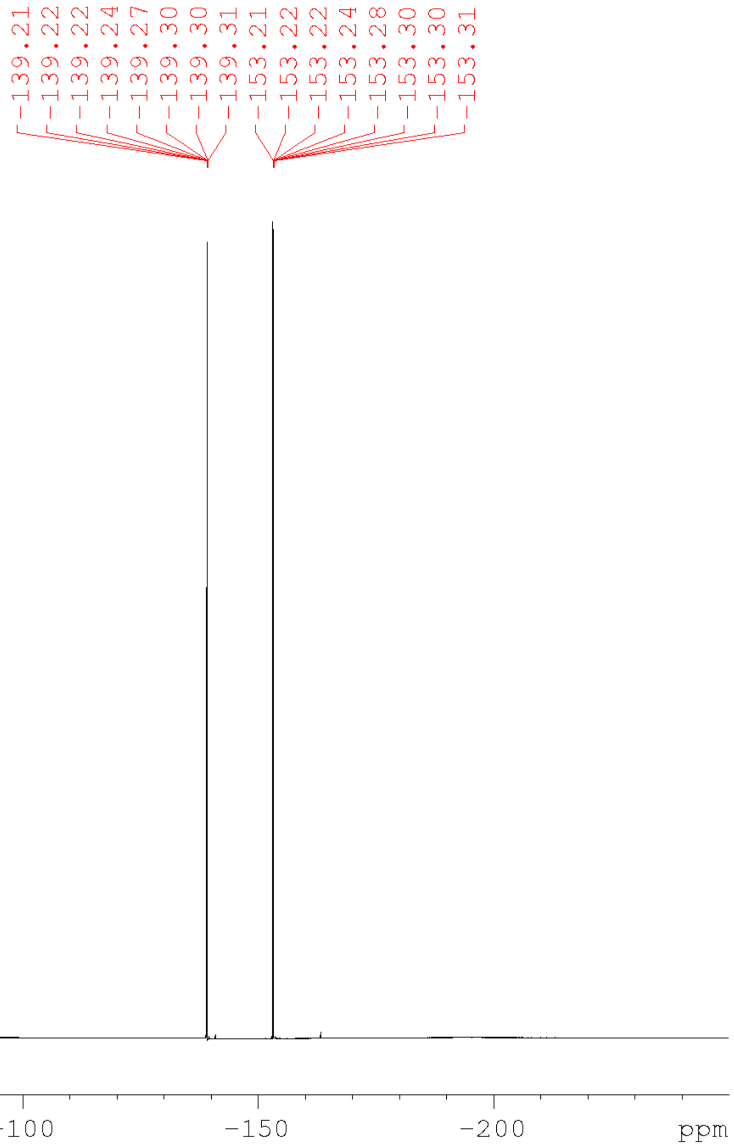

^{1}H NMR (600 MHZ, $CDCl_3$) N-(2,3,5,6-tetrafluorophenyl)acetamide

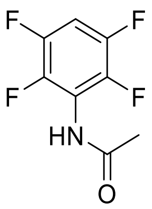

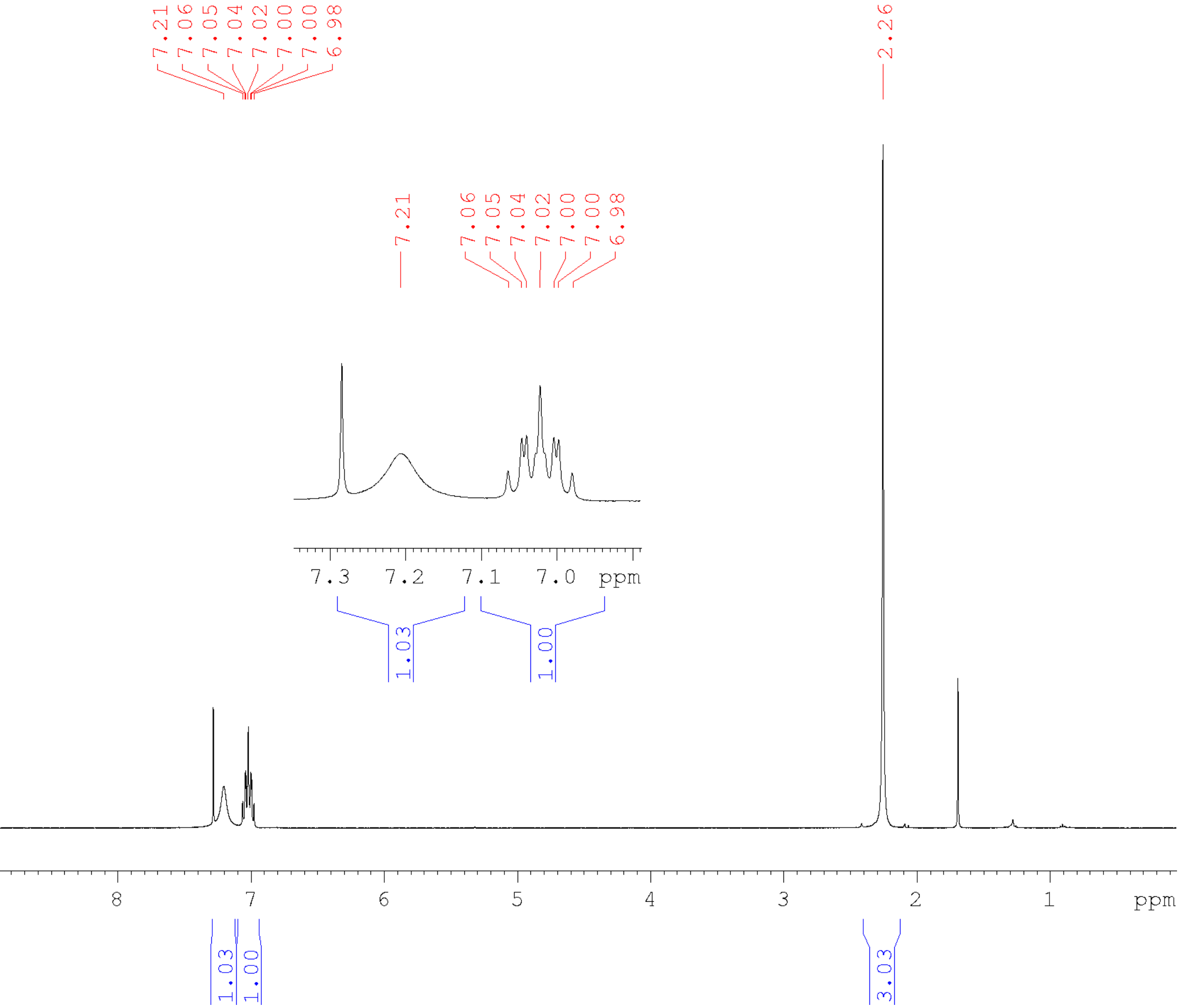

^{13}C NMR (101 MHZ, $CDCl_3$) N-(2,3,5,6-tetrafluorophenyl)acetamide

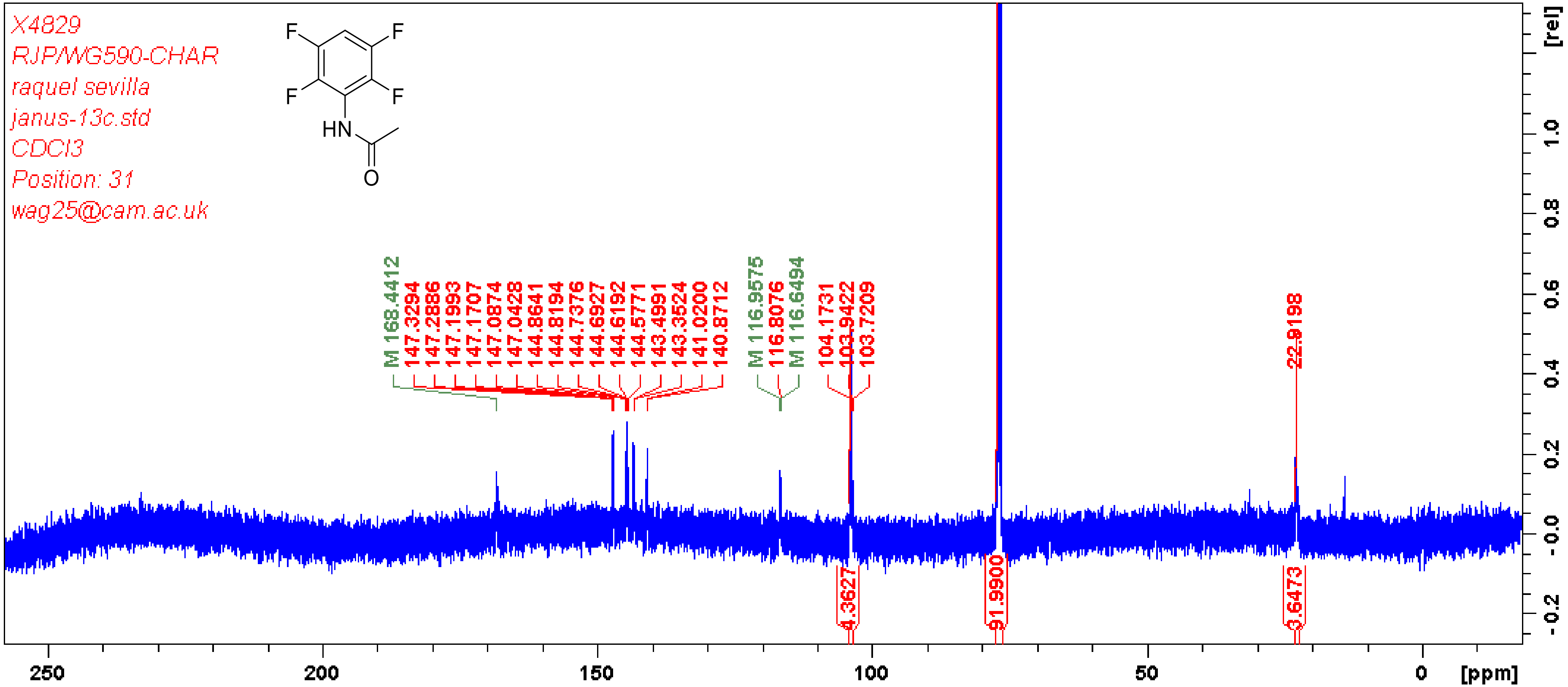

^{19}F NMR (376 MHZ, $CDCl_3$) N-(2,3,5,6-tetrafluorophenyl)acetamide

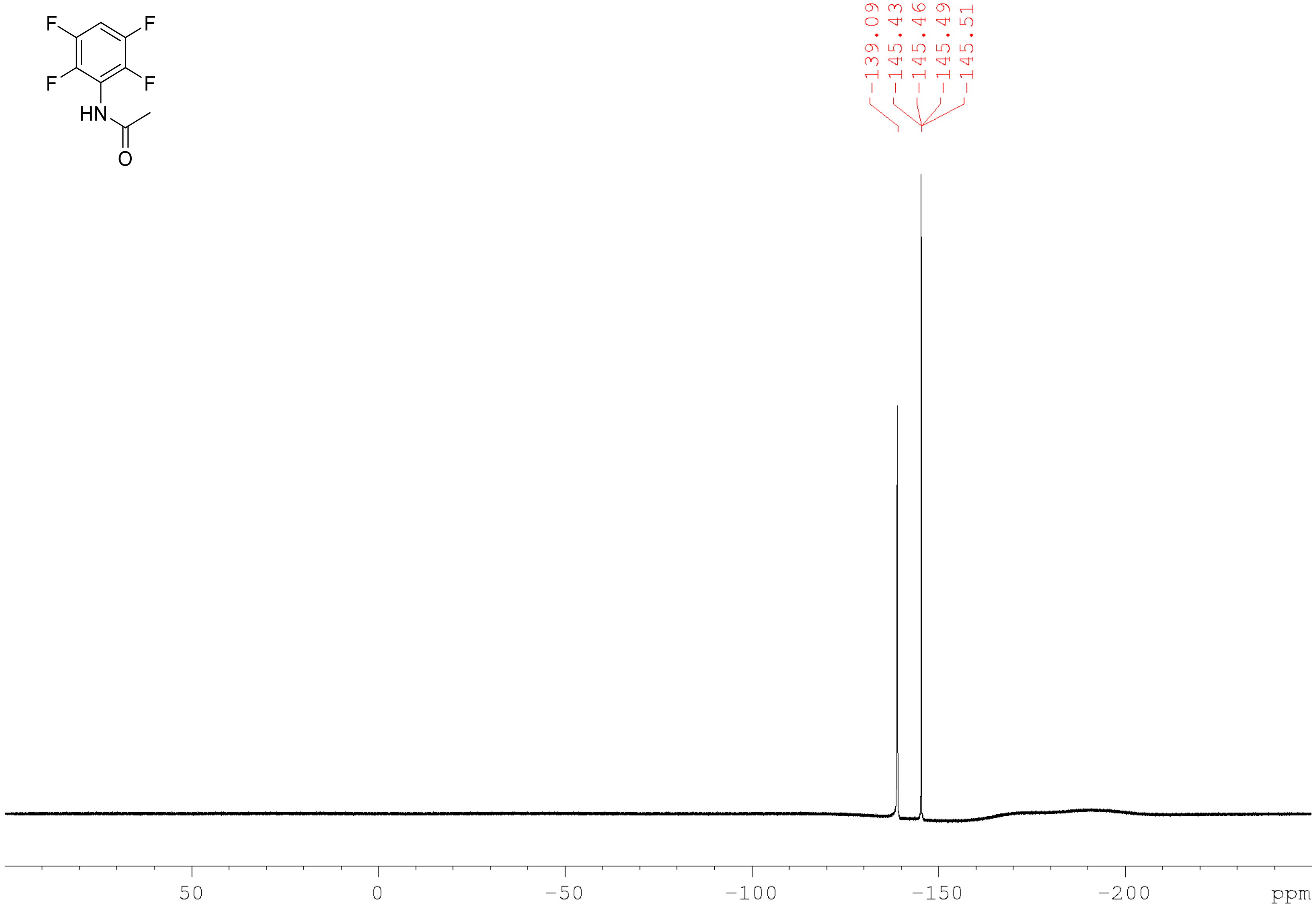

^{1}H NMR (400 MHZ, $CDCl_3$) 2,2',3,3',4,5,5',6,6'-nonafluoro-1,1'-biphenyl

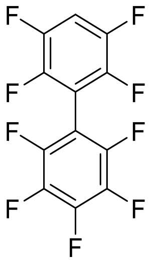

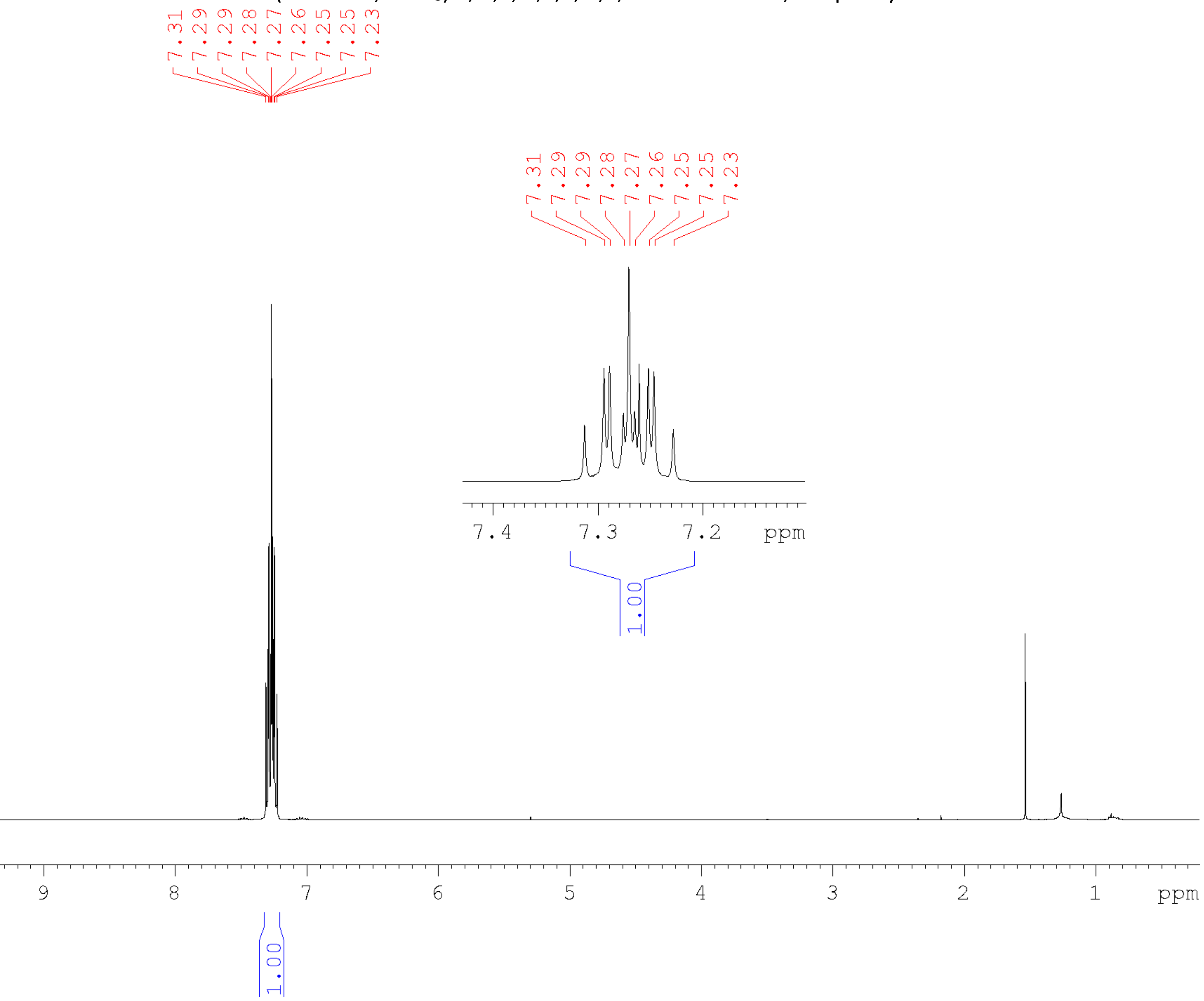

^{13}C NMR (400 MHZ, $CDCl_3$) 2,2',3,3',4,5,5',6,6'-nonafluoro-1,1'-biphenyl

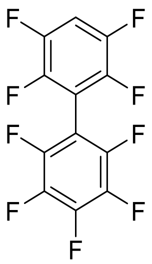

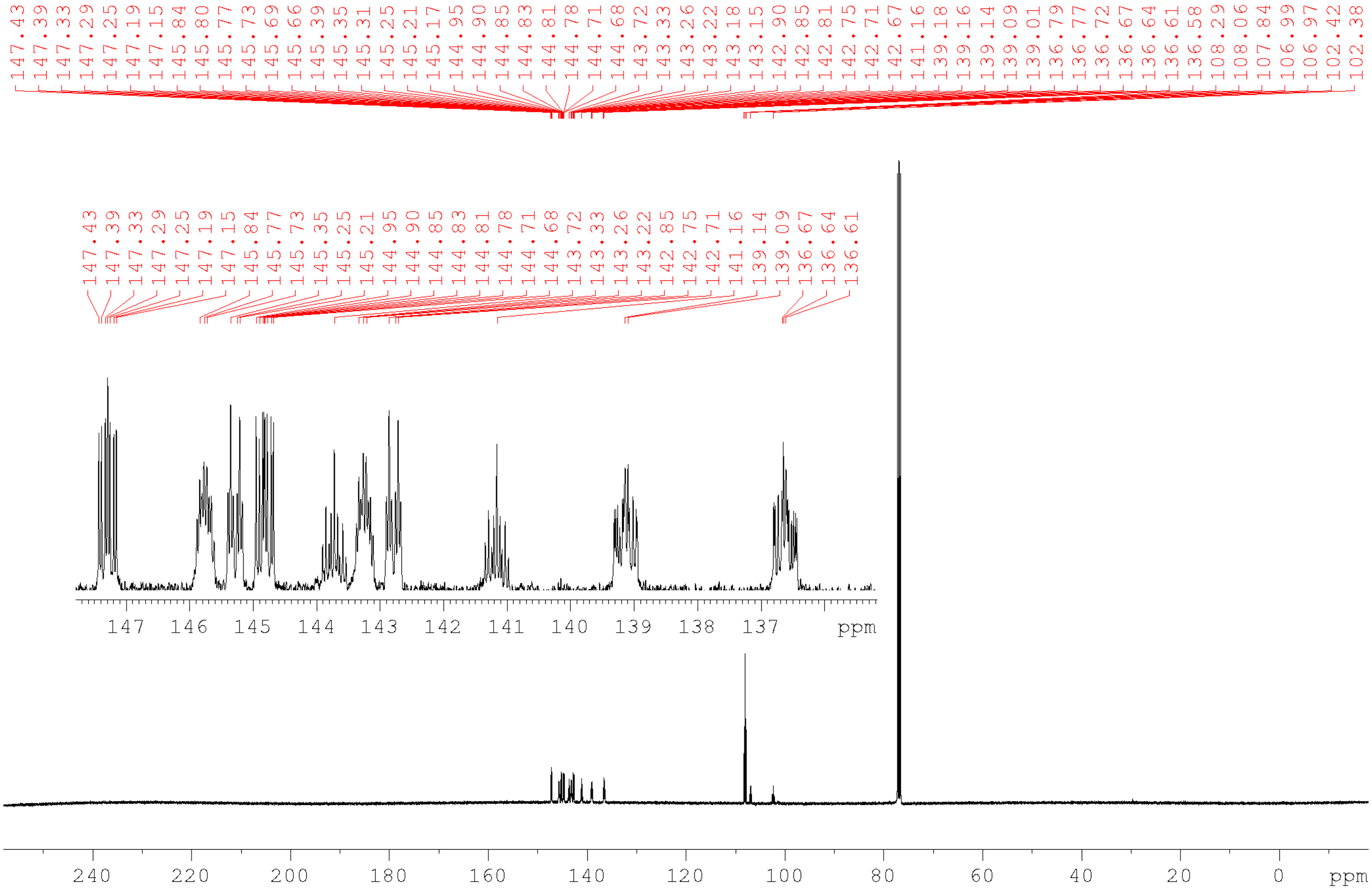

^{19}F NMR (376 MHZ, $CDCl_3$) 2,2',3,3',4,5,5',6,6'-nonafluoro-1,1'-biphenyl

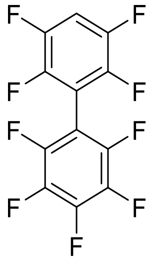

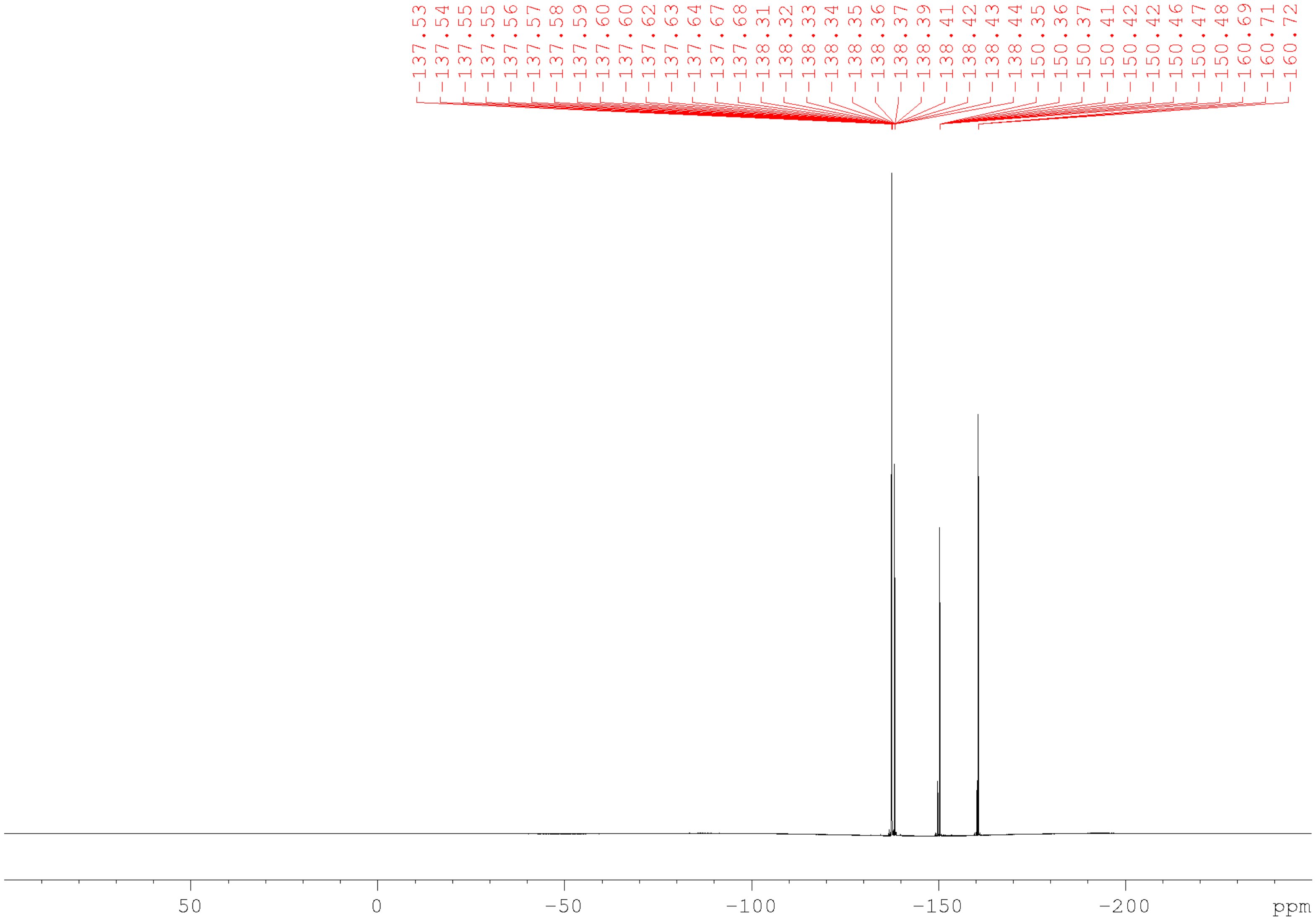

^{1}H NMR (400 MHZ, $CDCl_3$) N-(3,4-dichlorophenethyl)-1,1,1-trifluoromethanesulfonamide

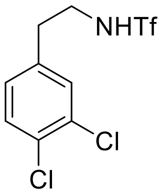

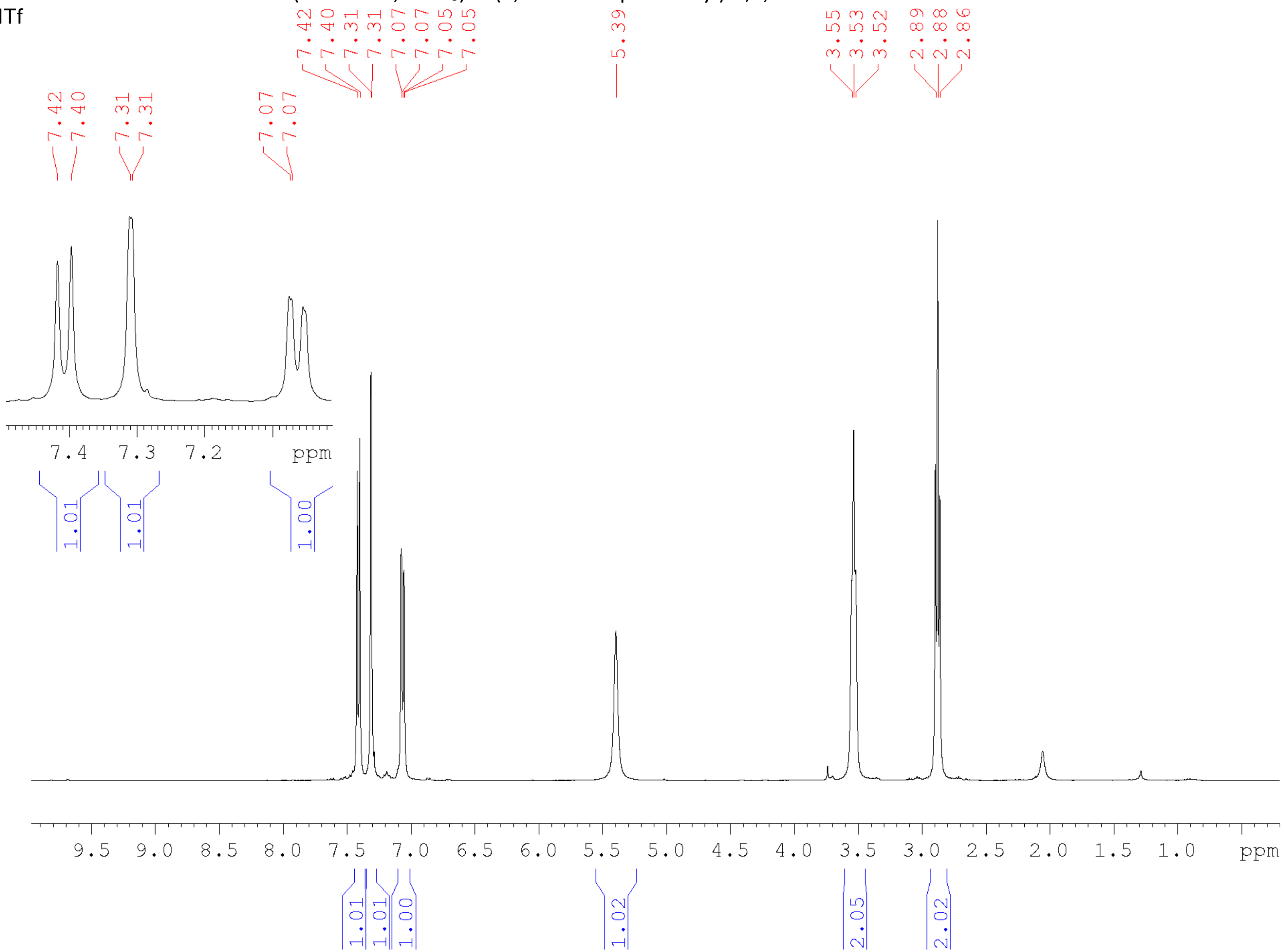

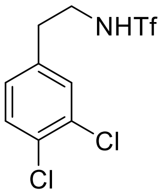

^{13}C NMR (101 MHZ, $CDCl_3$) N-(3,4-dichlorophenethyl)-1,1,1-trifluoromethanesulfonamide

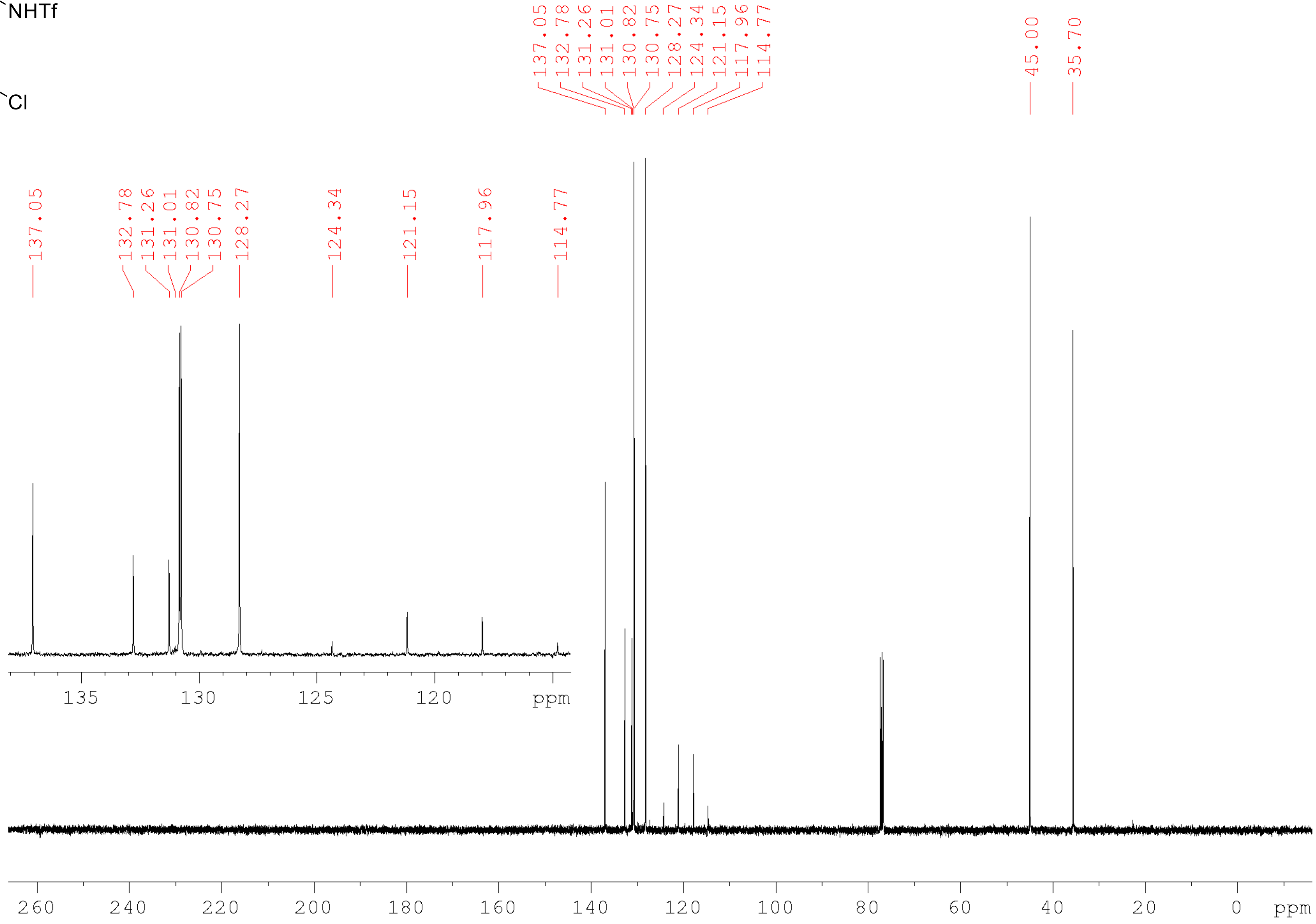

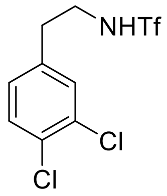

^{19}F NMR (376 MHZ, $CDCl_3$) N-(3,4-dichlorophenethyl)-1,1,1-trifluoromethanesulfonamide (WG219)

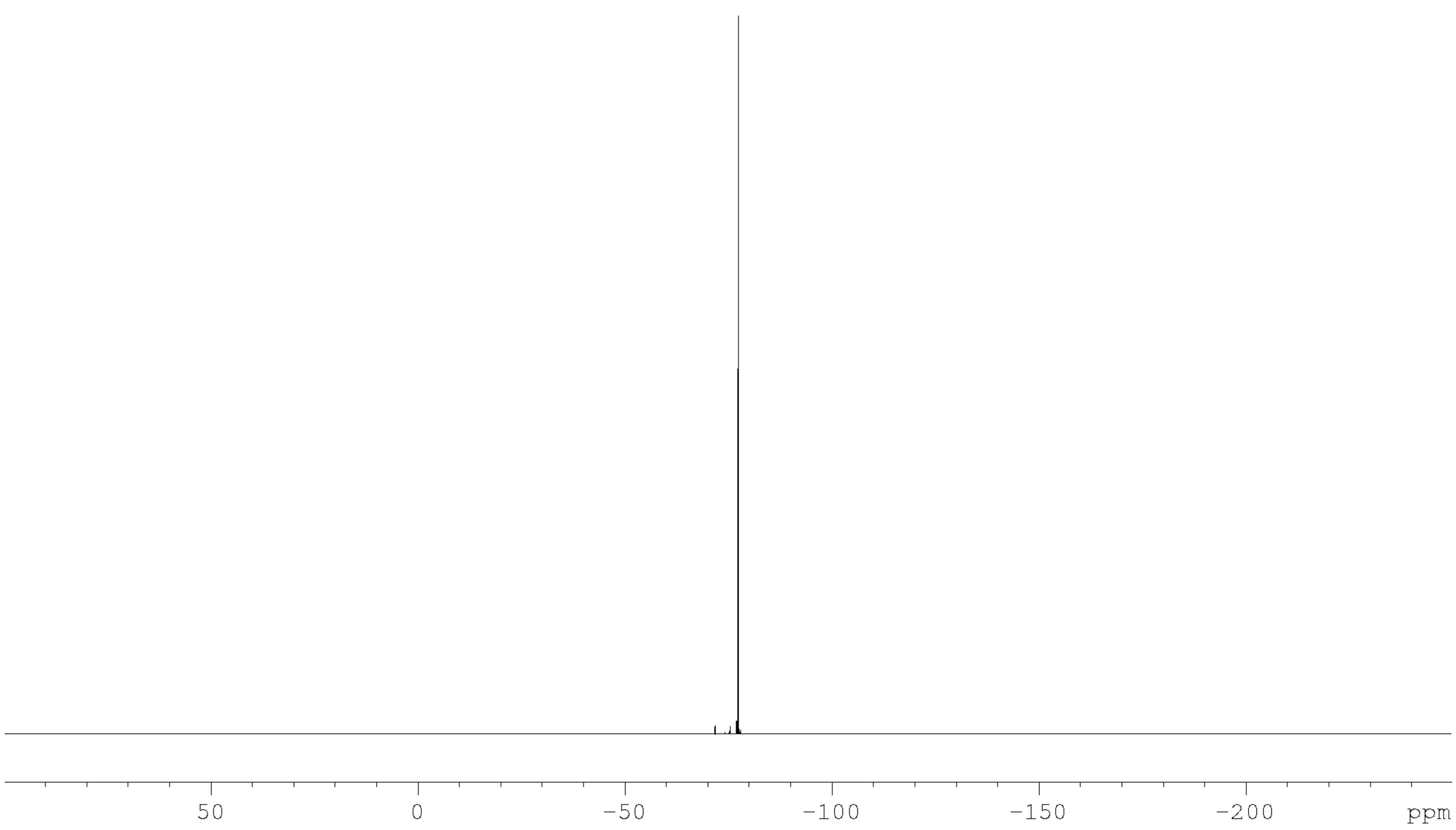

^{1}H NMR (400 MHZ, $CDCl_3$) N-(3-(3,4-dichlorophenyl)propyl)-1,1,1-trifluoromethanesulfonamide

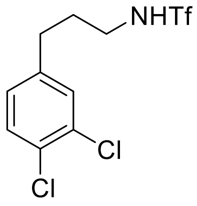

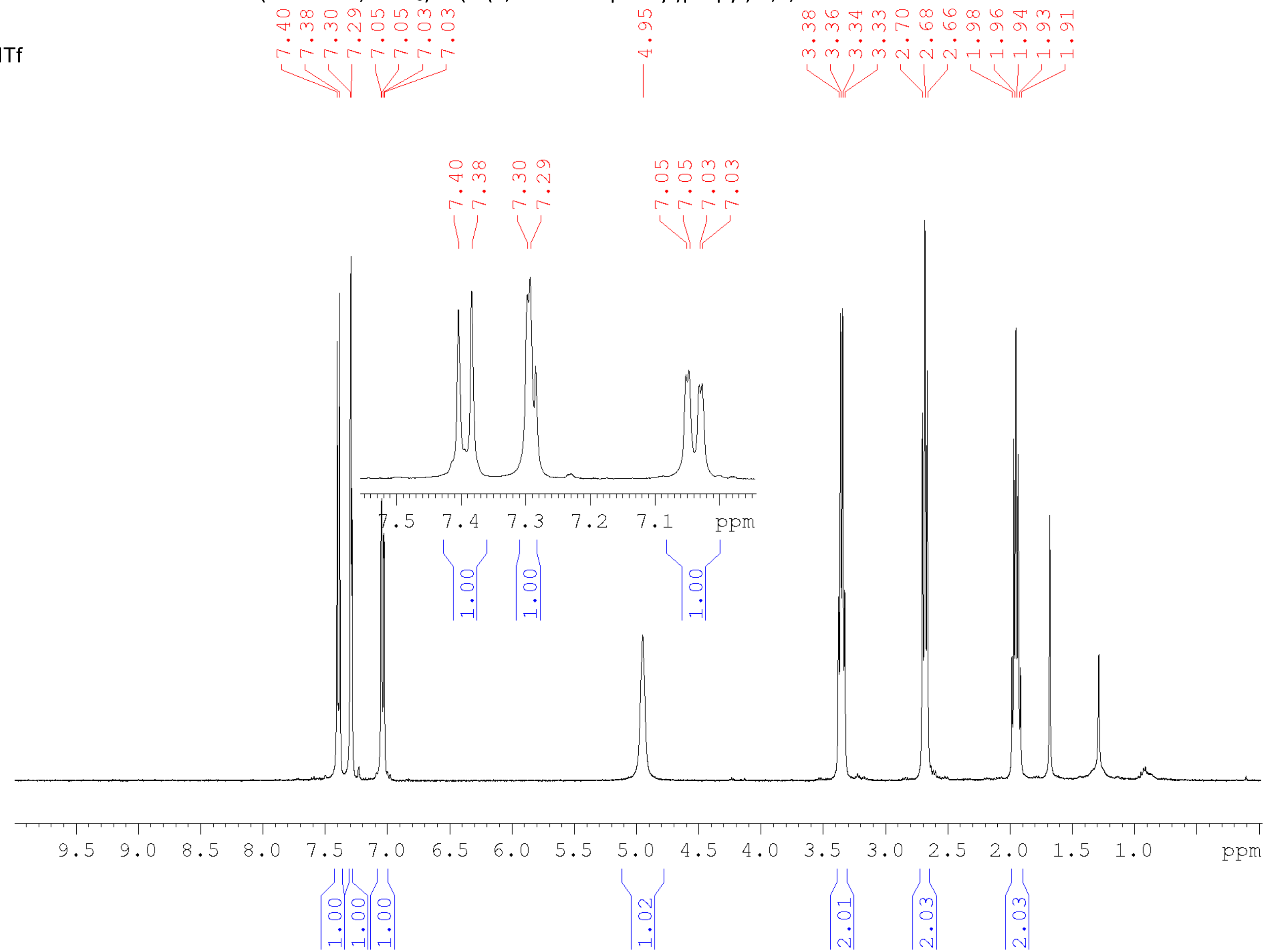

^{13}C NMR (151 MHZ, $CDCl_3$) N-(3-(3,4-dichlorophenyl)propyl)-1,1,1-trifluoromethanesulfonamide

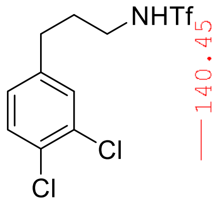

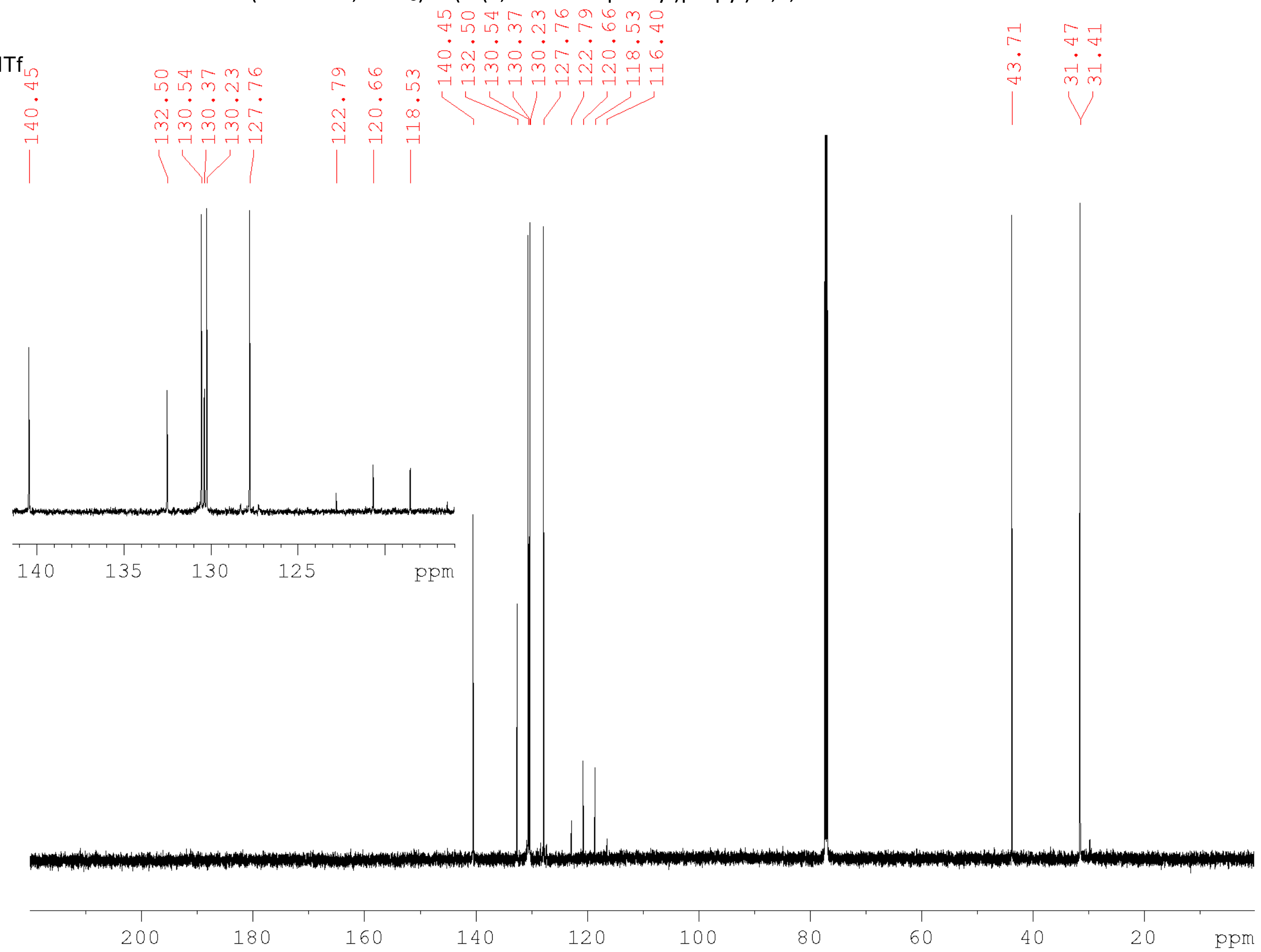

^{19}F NMR (376 MHZ, $CDCl_3$) N-(3-(3,4-dichlorophenyl)propyl)-1,1,1-trifluoromethanesulfonamide

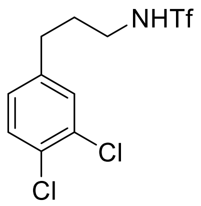

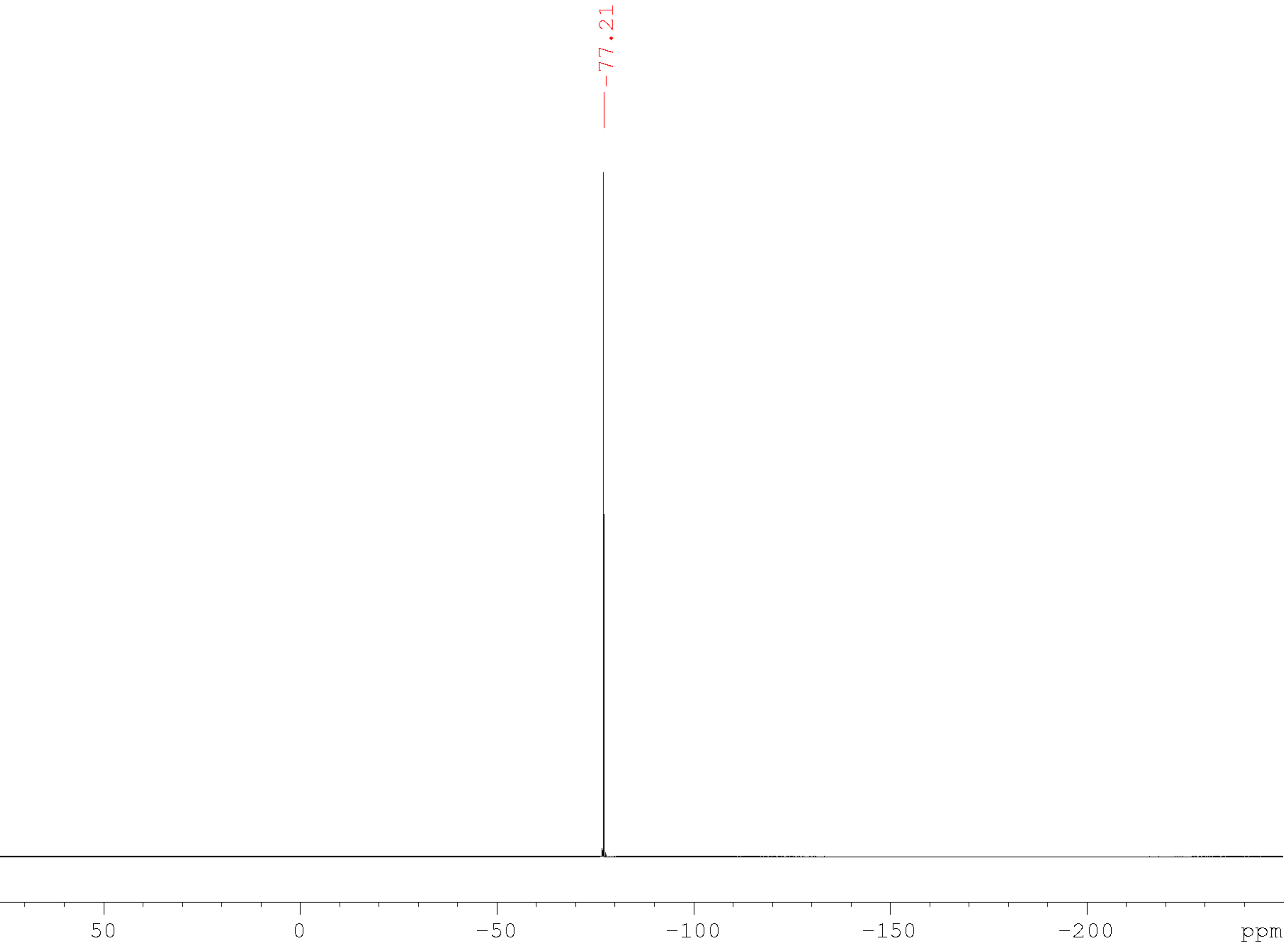

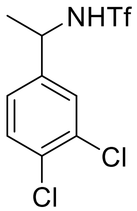

^{1}**H NMR** (400 MHZ, $CDCl_3$) N-(1-(3,4-dichlorophenyl)ethyl)-1,1,1-trifluoromethanesulfonamide

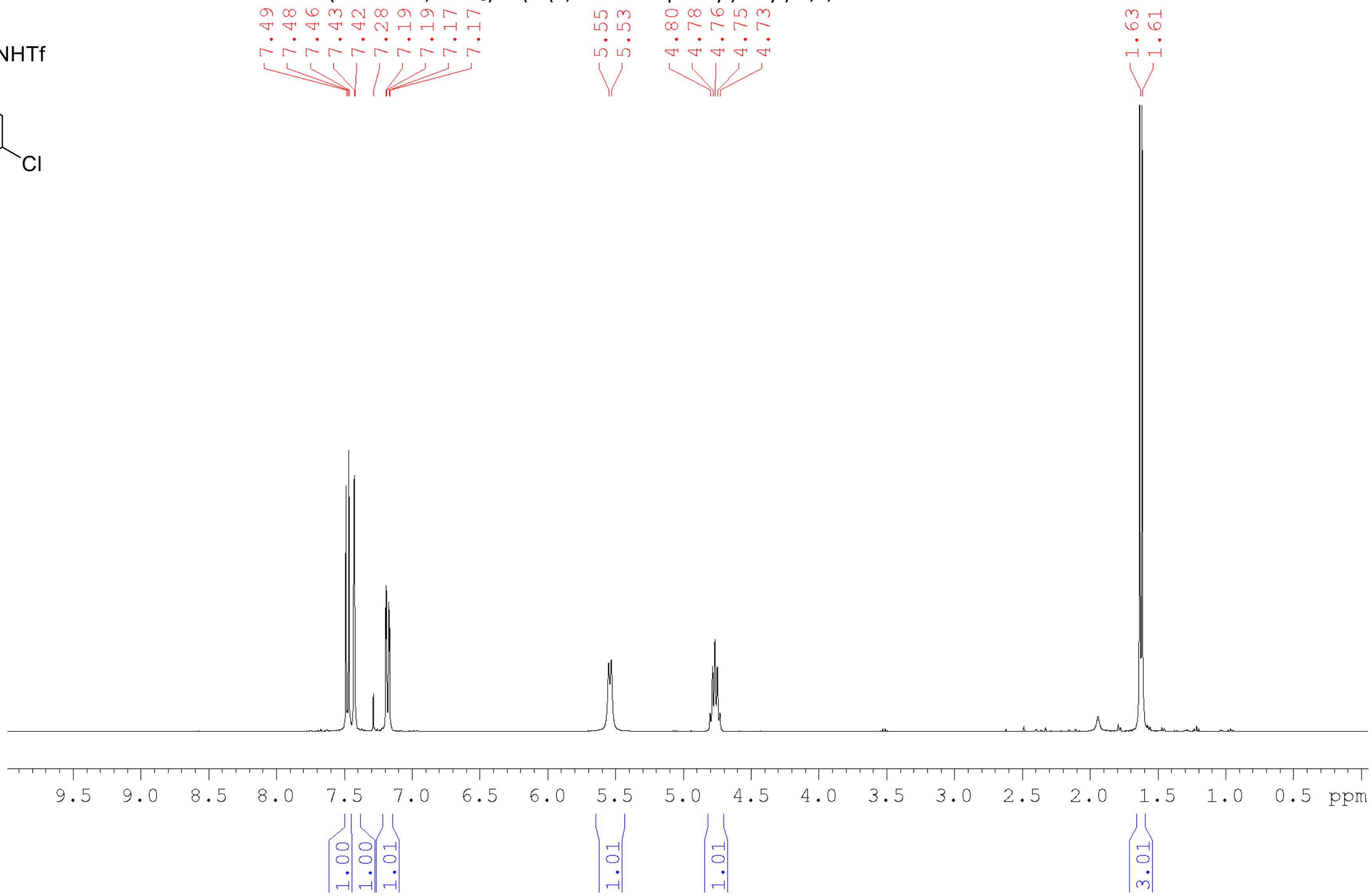

^{13}C NMR (101 MHZ, $CDCl_3$) N-(1-(3,4-dichlorophenyl)ethyl)-1,1,1-trifluoromethanesulfonamide

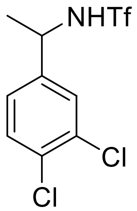

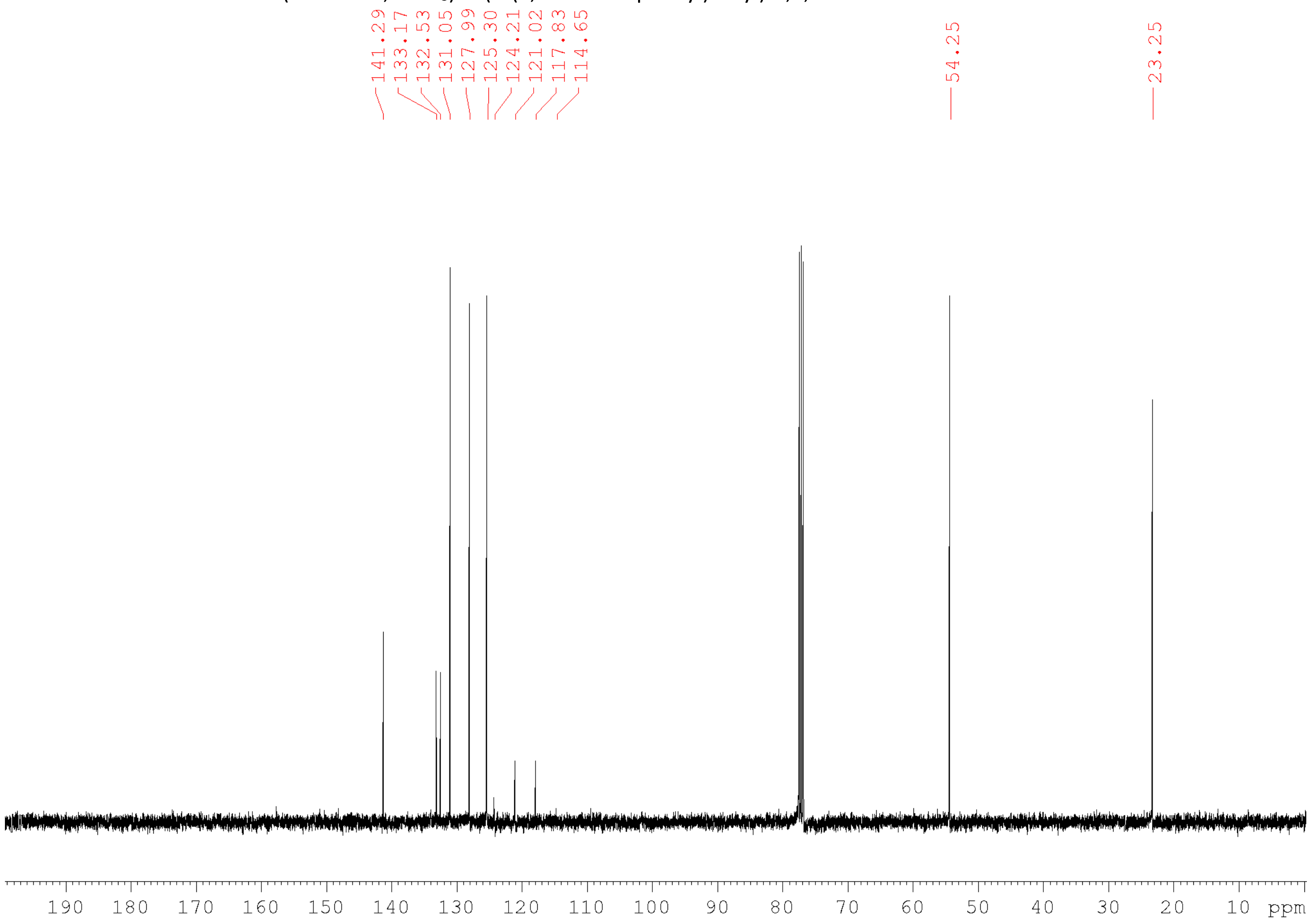

^{19}F NMR (376 MHZ, CDCl3) N-(1-(3,4-dichlorophenyl)ethyl)-1,1,1-trifluoromethanesulfonamide

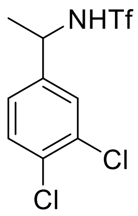

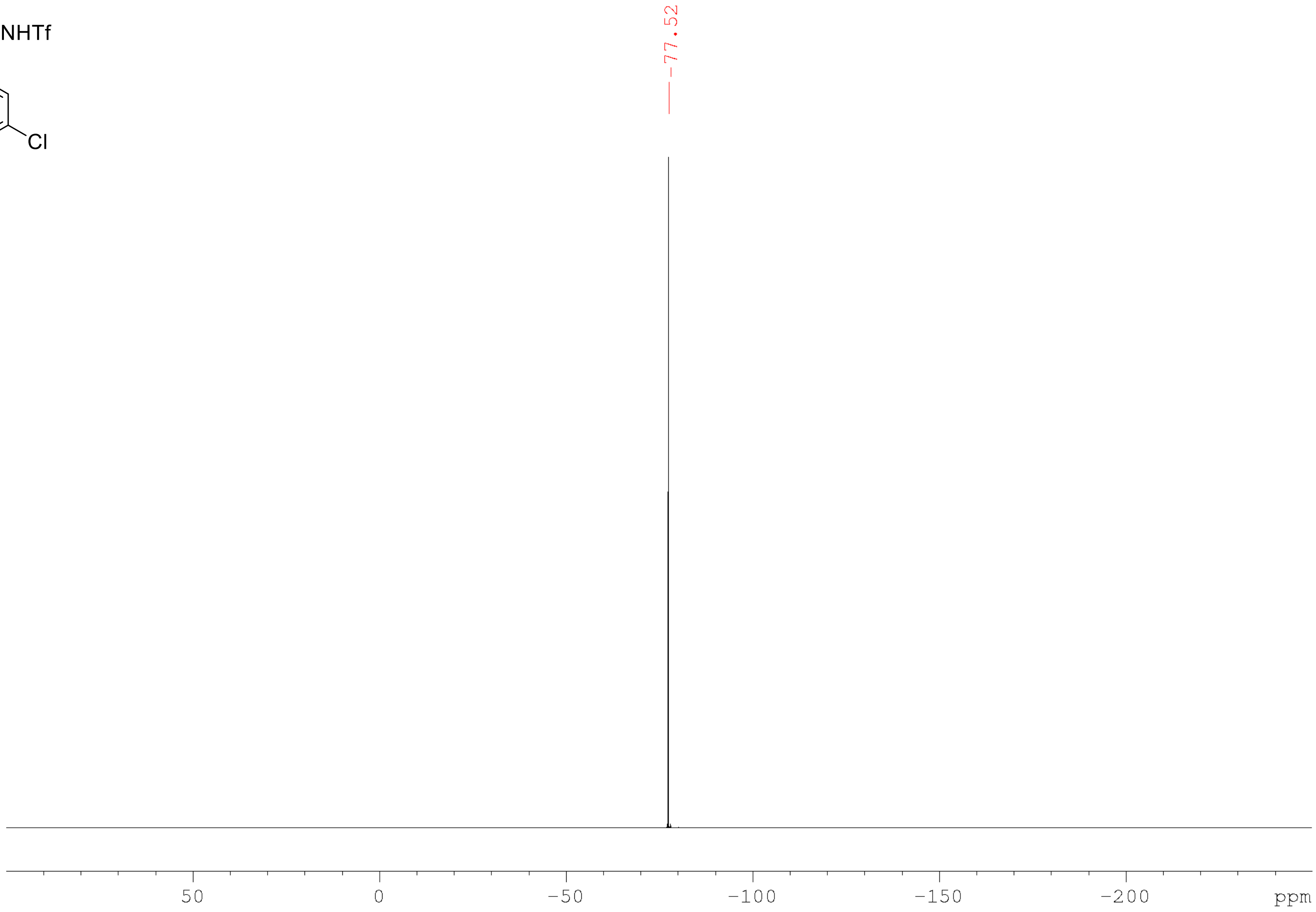

^{1}H NMR (600 MHZ, $CDCl_3$) N-((3,4-dichlorophenyl)(phenyl)methyl)-1,1,1-trifluoromethanesulfonamide

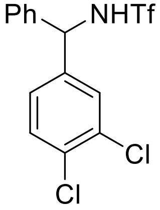

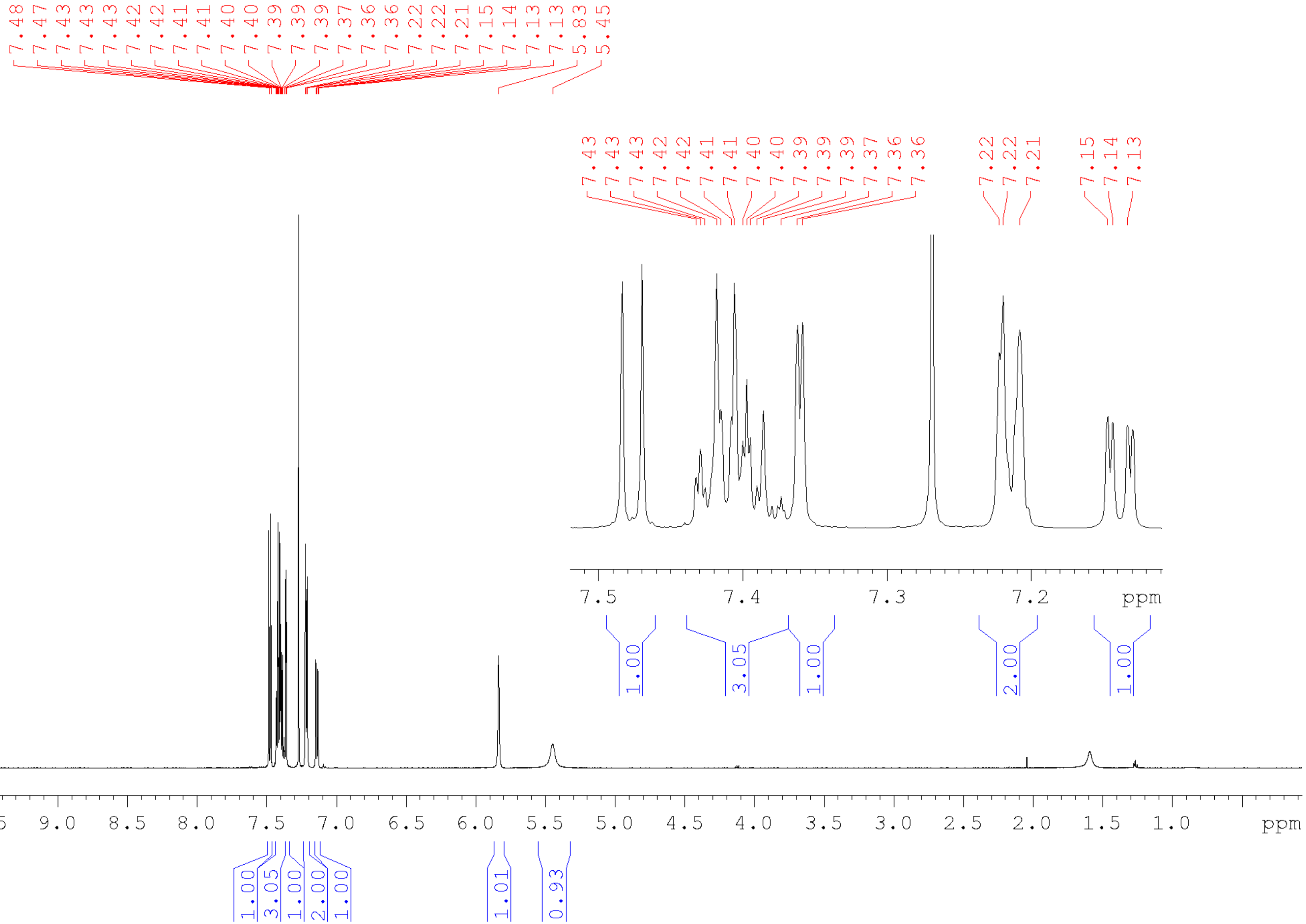

^{13}C NMR (101 MHZ, $CDCl_3$) N-((3,4-dichlorophenyl)(phenyl)methyl)-1,1,1-trifluoromethanesulfonamide

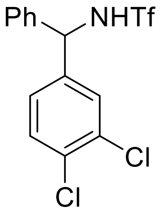

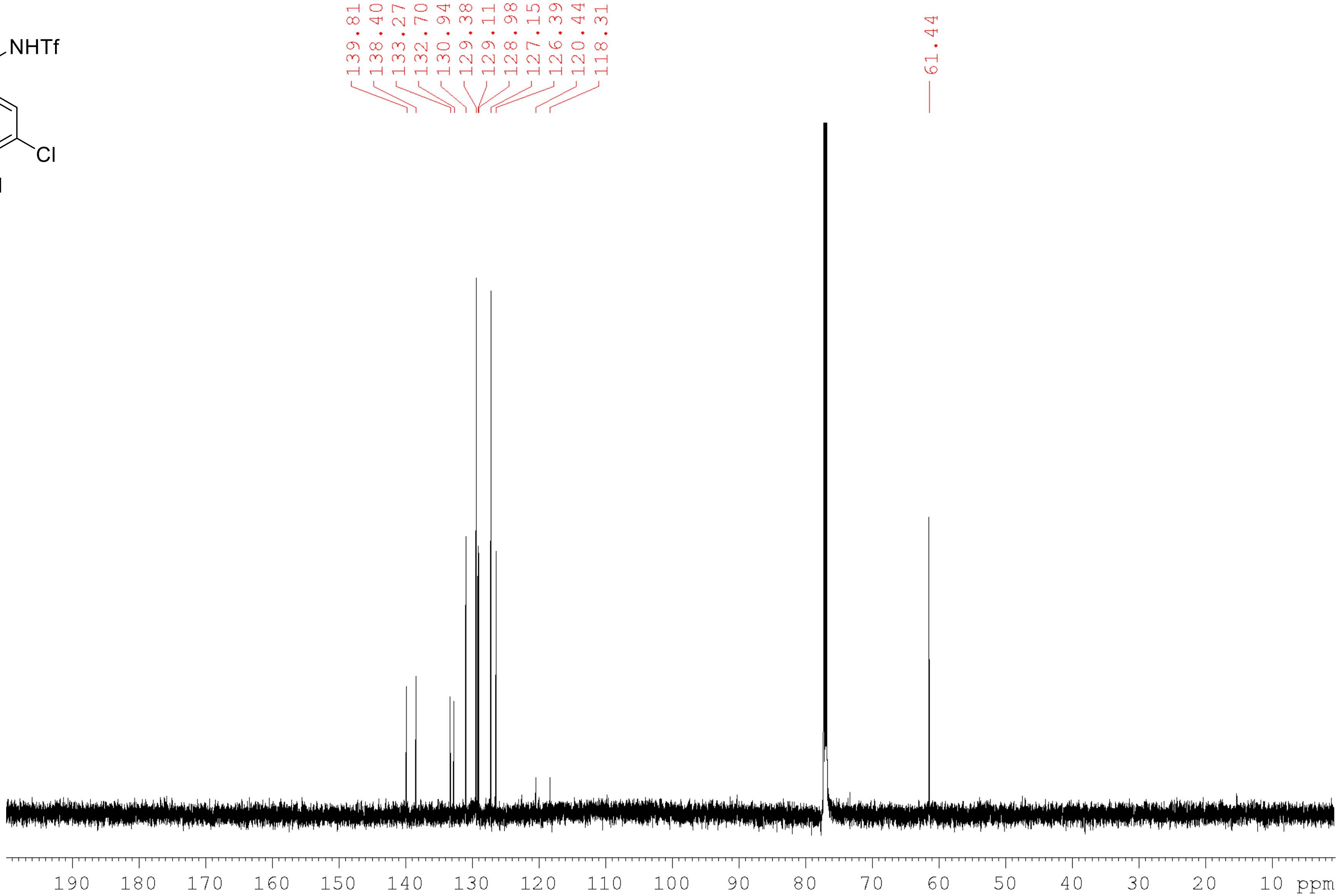

^{19}F NMR (376 MHZ, $CDCl_3$) N-((3,4-dichlorophenyl)(phenyl)methyl)-1,1,1-trifluoromethanesulfonamide

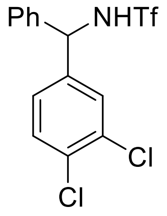

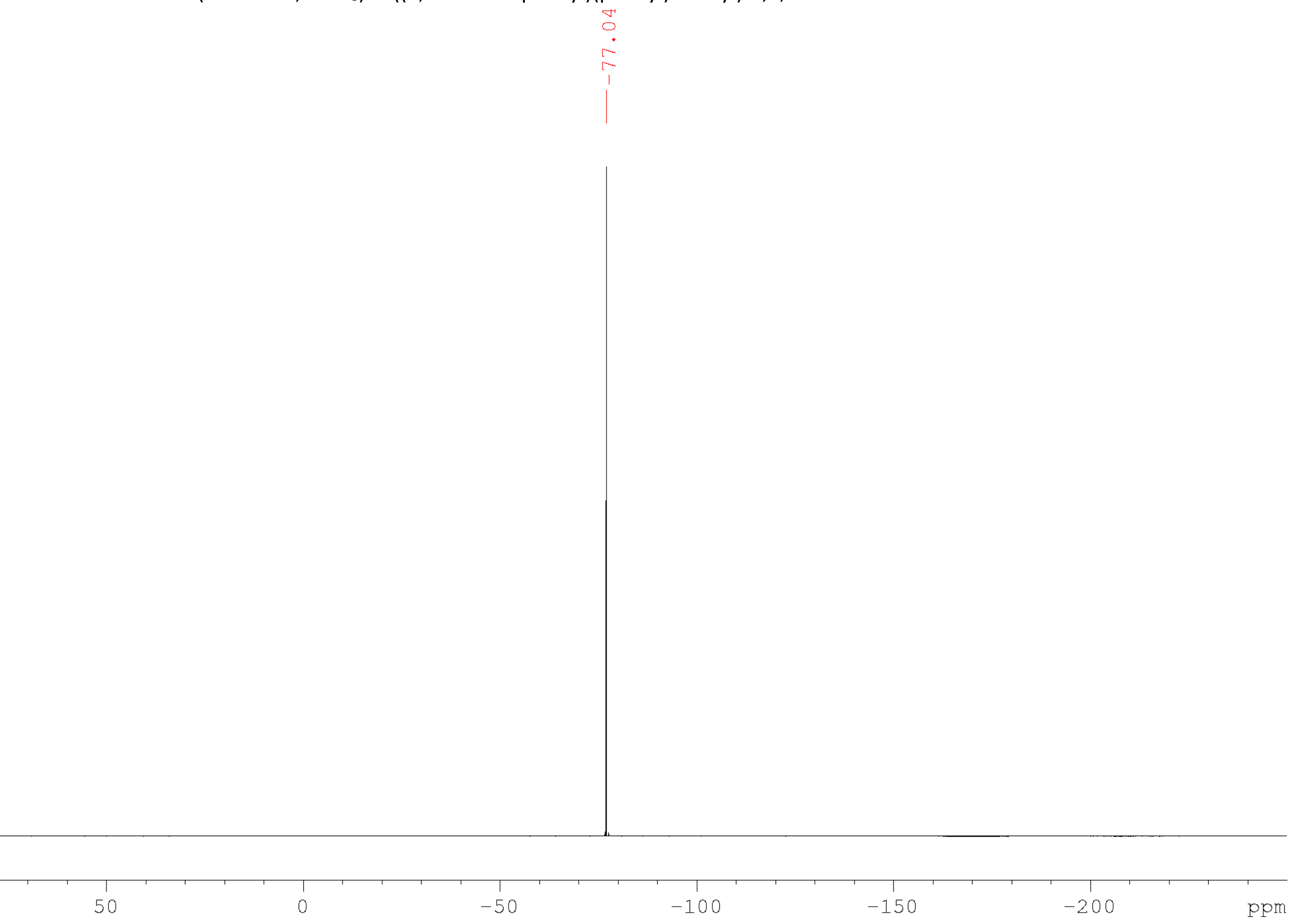

^{1}H NMR (600 MHz, MeOD) **(1b)** sodium (3,4-dichlorophenyl)methanesulfonate

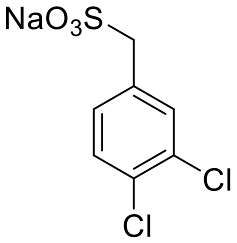

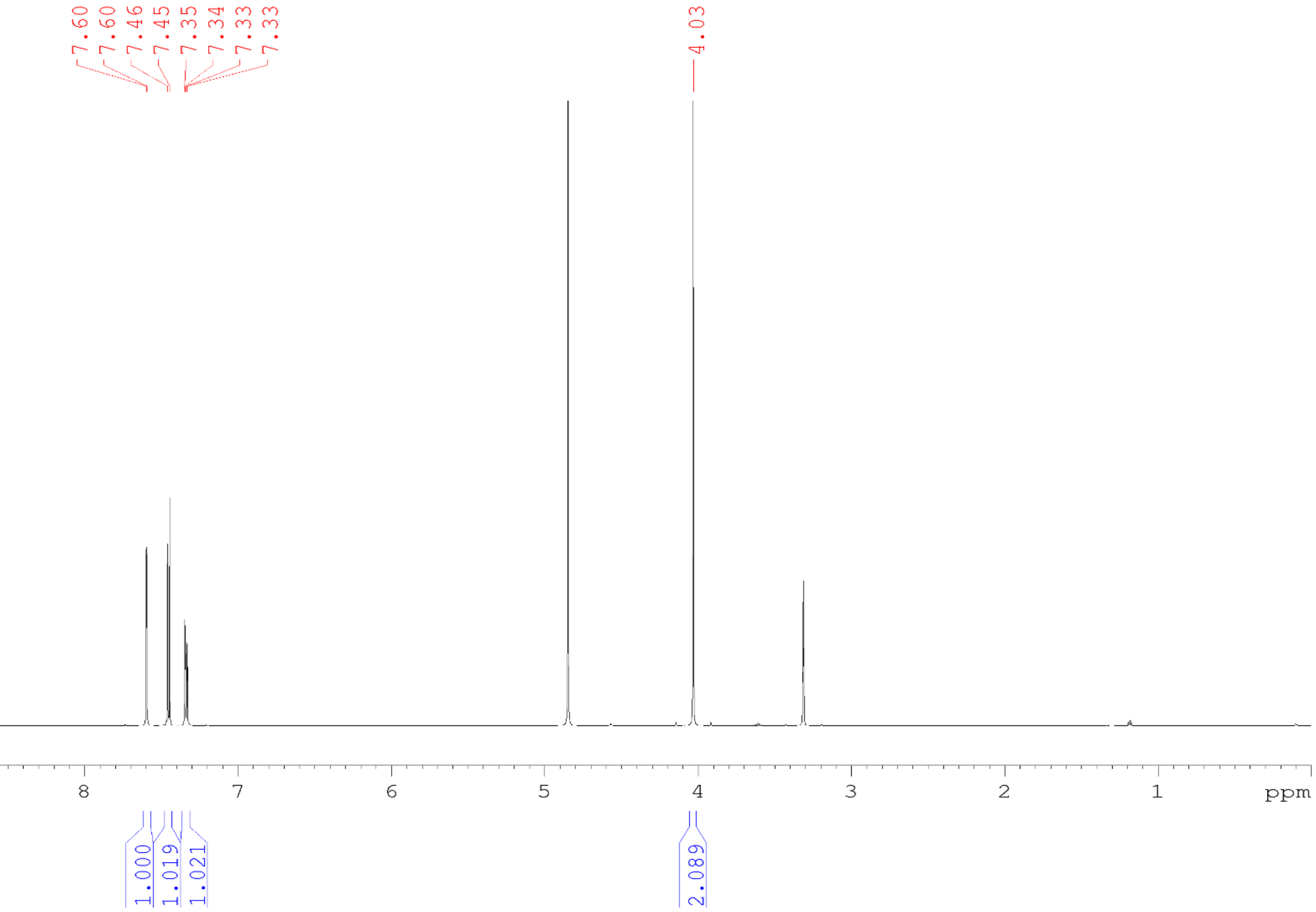

^{13}C NMR (151 MHz, MeOD) **(1b)** sodium (3,4-dichlorophenyl)methanesulfonate

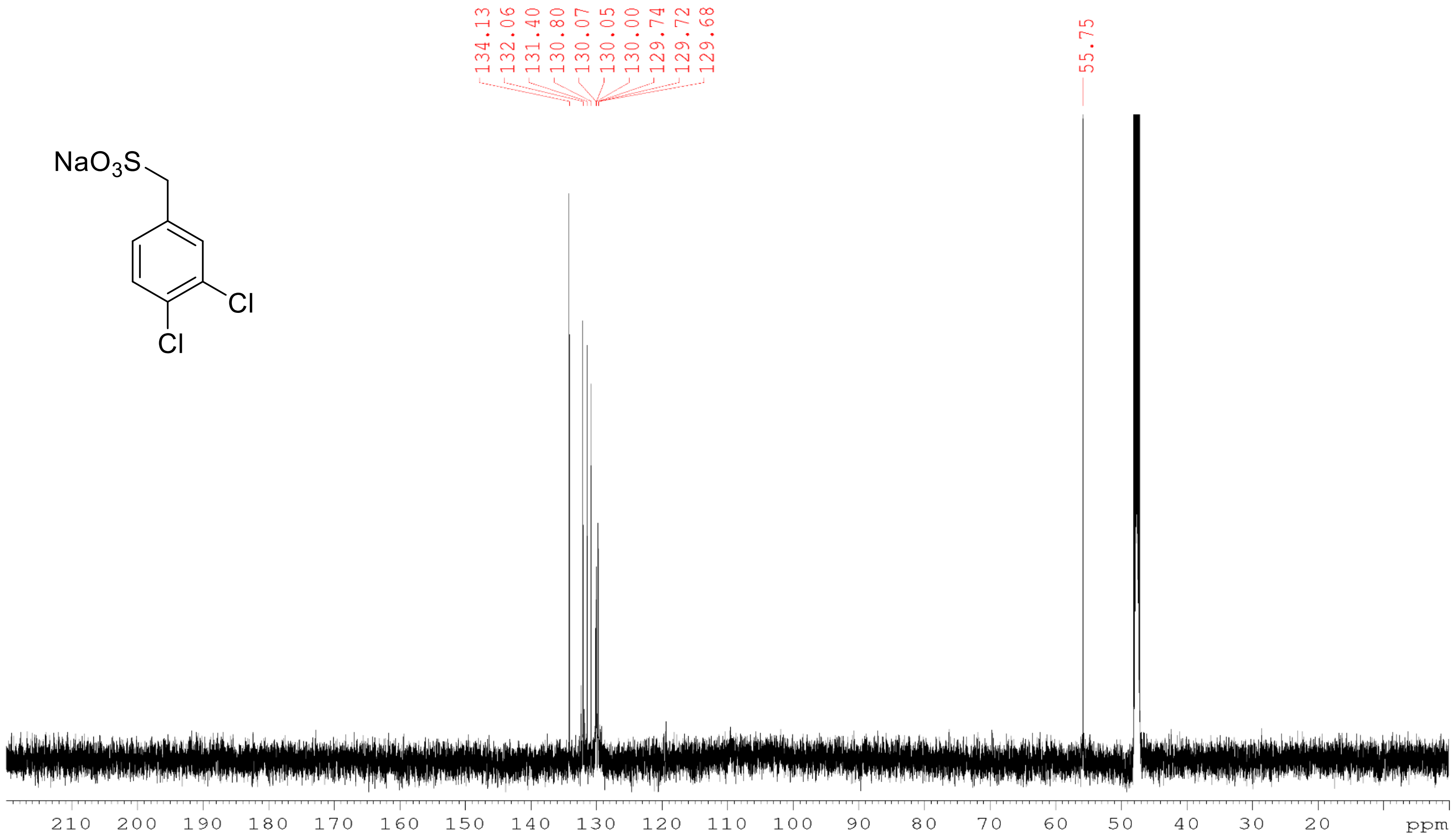

^{1}H NMR (400 MHz, DMSO) **(1c)** potassium (3,4-dichlorobenzyl)sulfamate

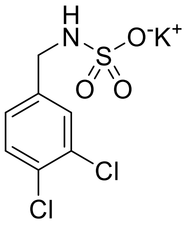

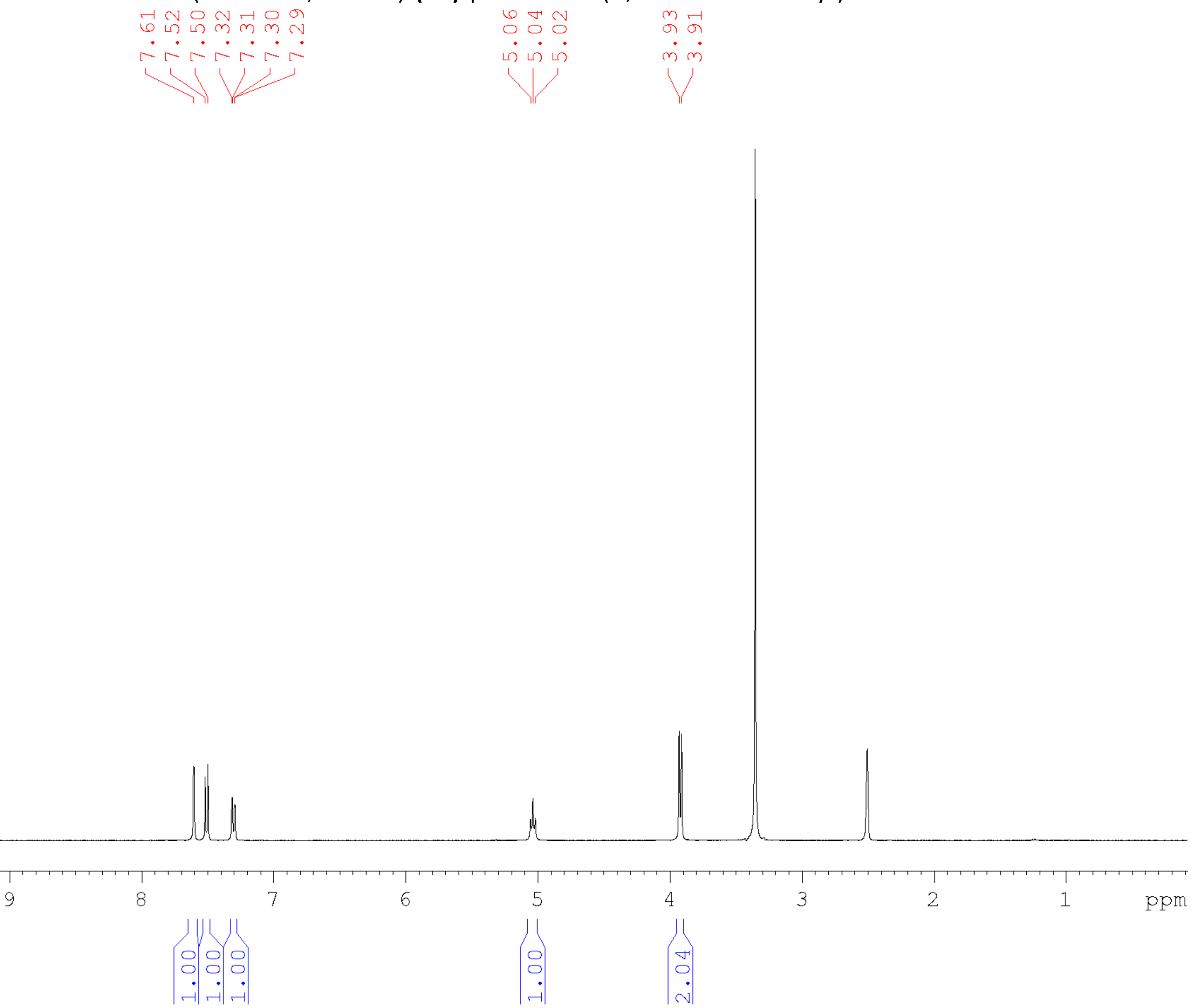

^{13}**C NMR** (100 MHz, DMSO) **(1c)** potassium (3,4-dichlorobenzyl)sulfamate

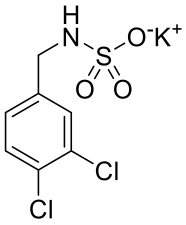

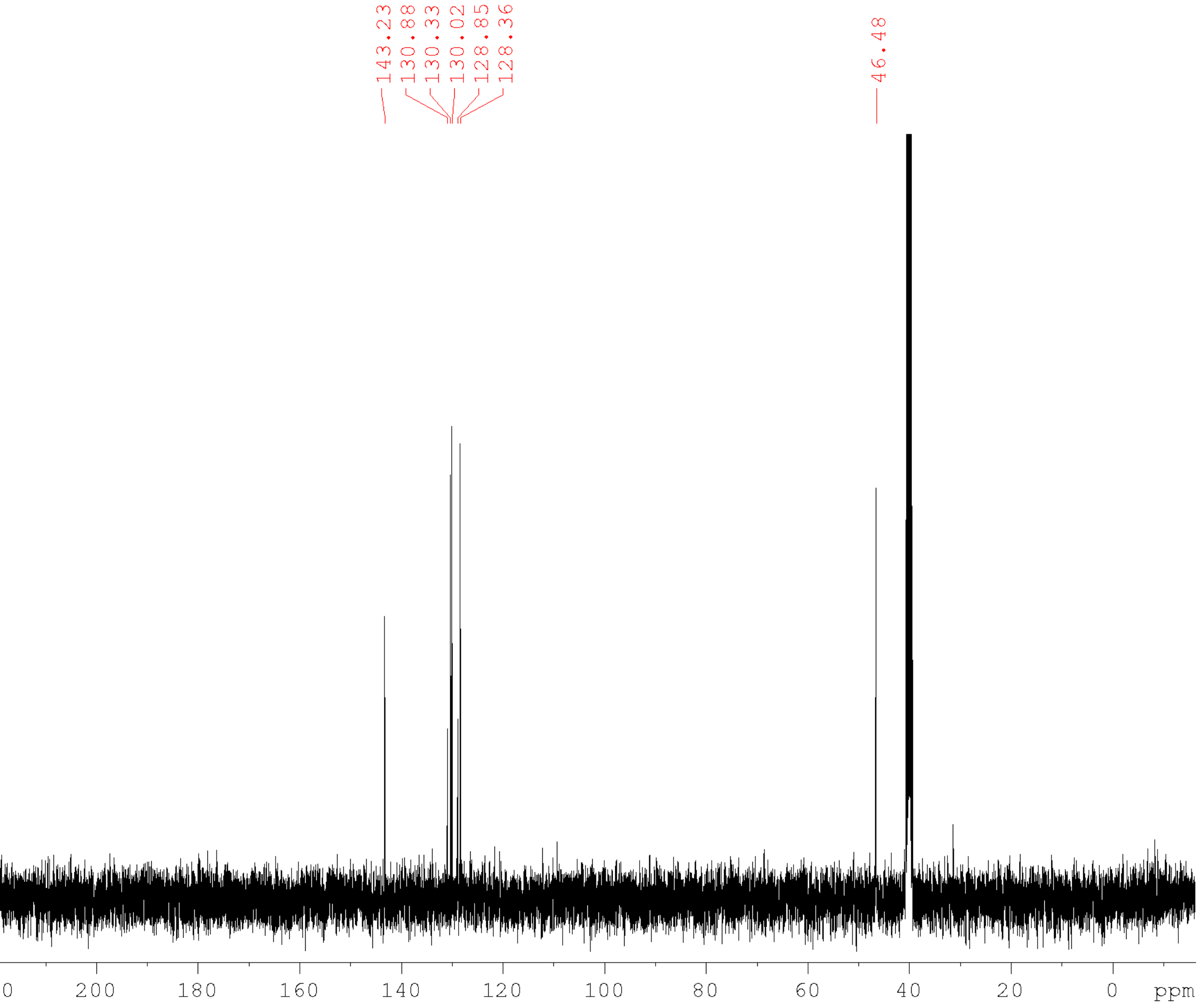

^{1}H NMR (400 MHz, $CDCl_3$) **(1d)** ethyl hydrogen (3,4-dichlorobenzyl)phosphonate

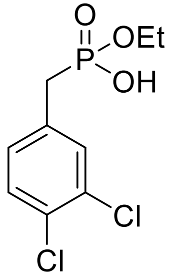

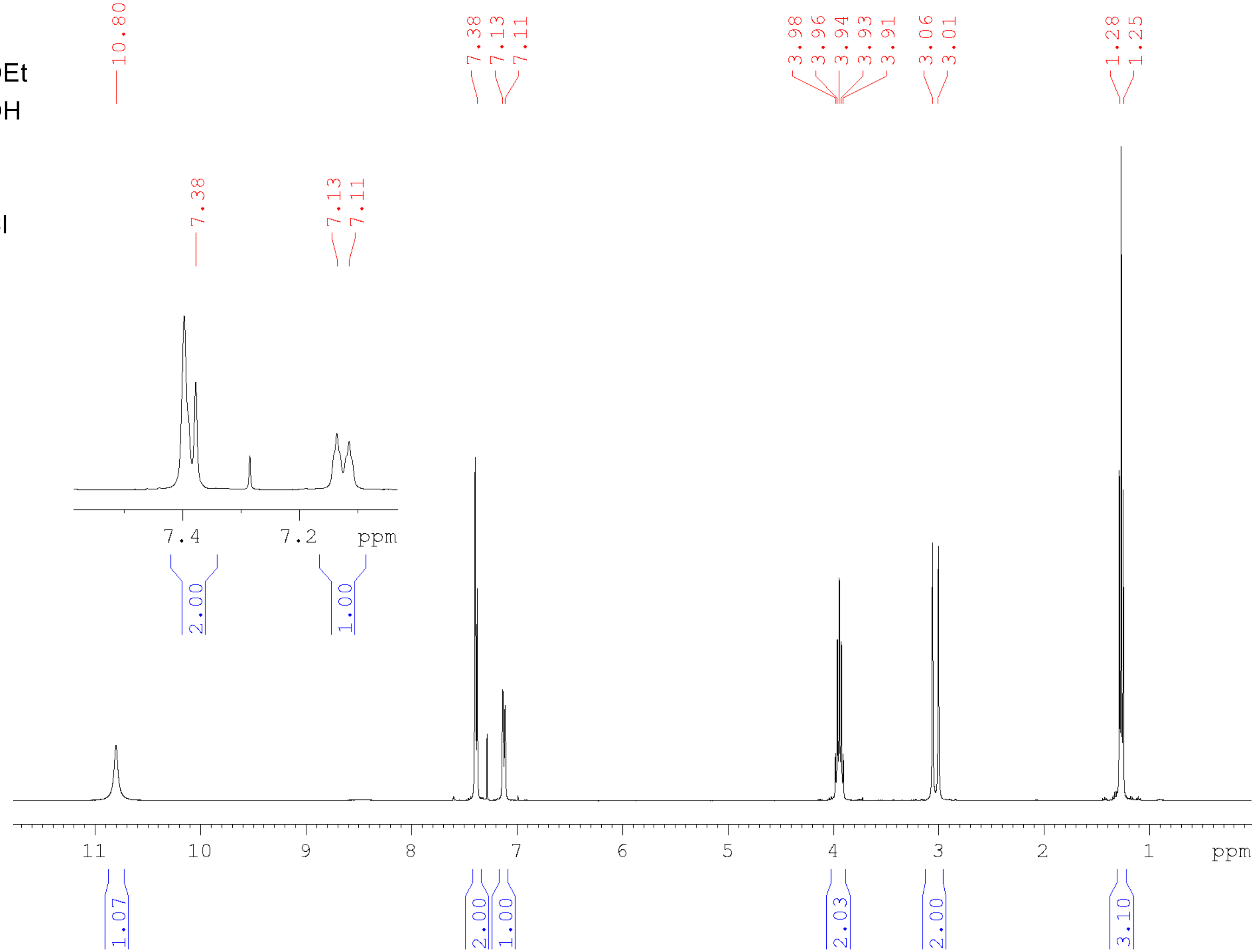

^{13}C NMR (101 MHz, $CDCl_3$) **(1d)** ethyl hydrogen (3,4-dichlorobenzyl)phosphonate

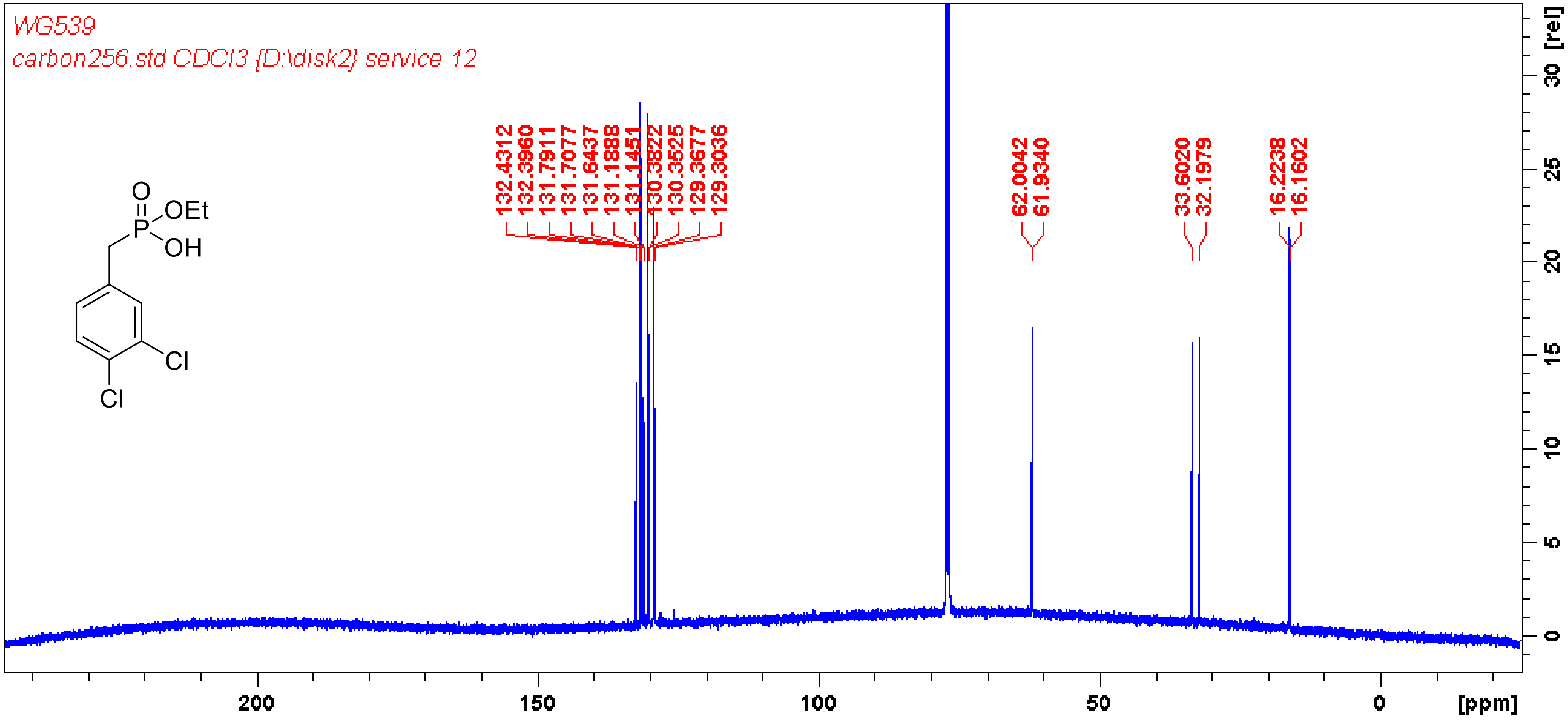

^{31}P NMR (162 MHz, $CDCl_3$) **(1d)** ethyl hydrogen (3,4-dichlorobenzyl)phosphonat

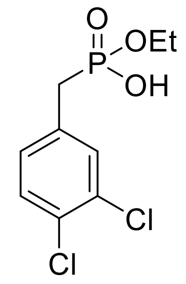

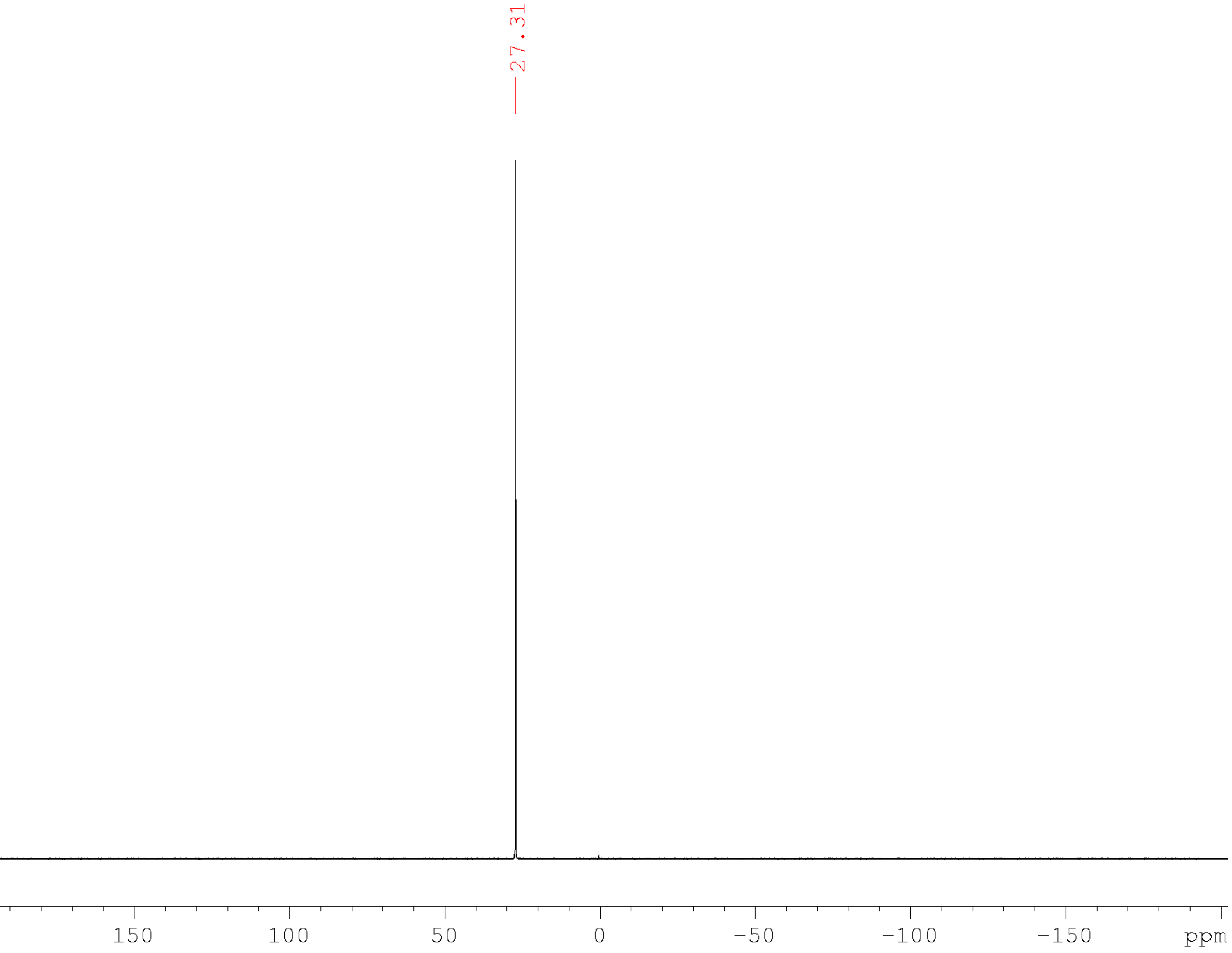

^{1}H NMR (400 MHz, $CDCl_3$) (2-carboxyethyl)triphenylphosphonium chloride

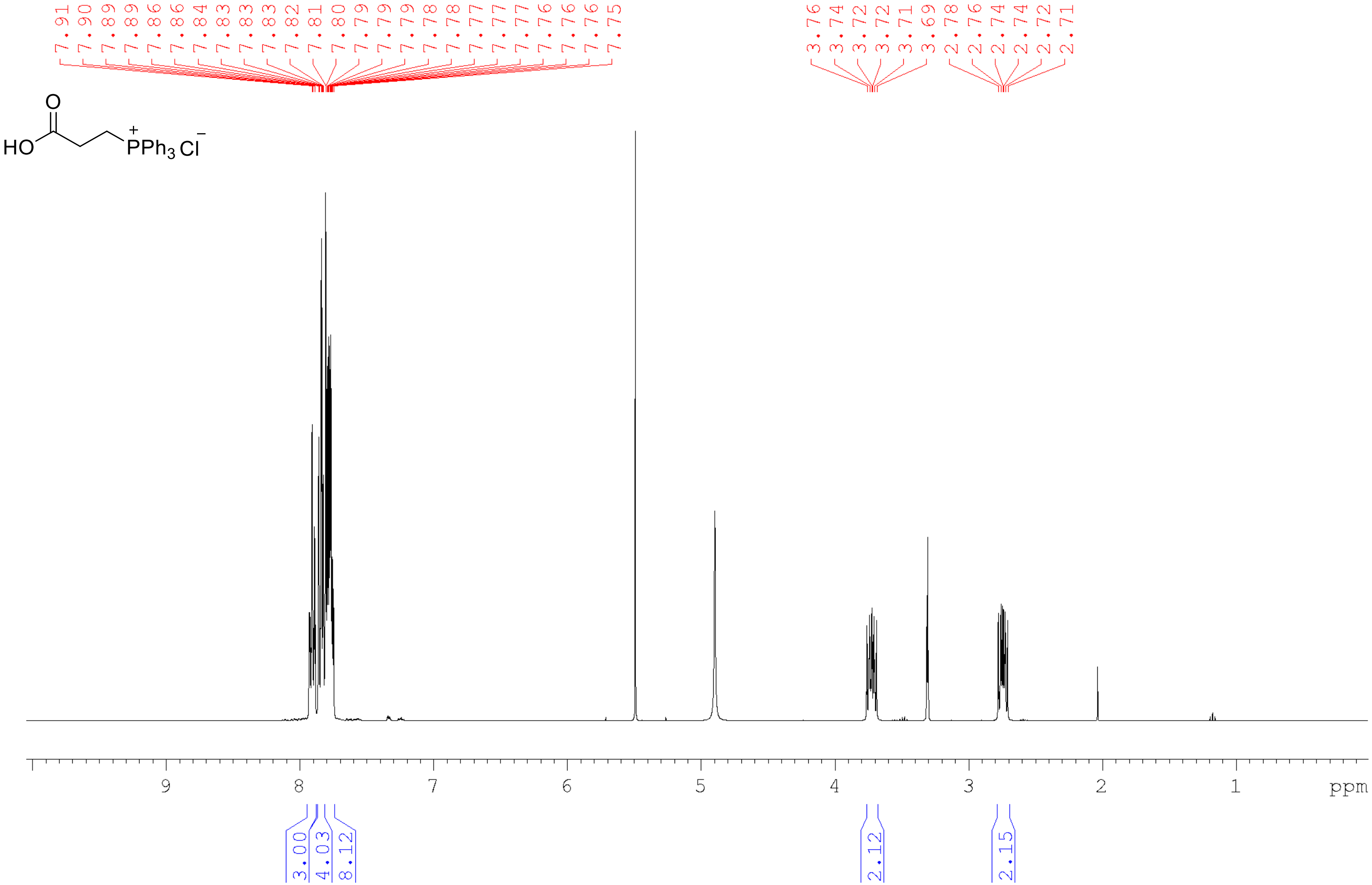

^{13}C NMR (101 MHz, $CDCl_3$) (2-carboxyethyl)triphenylphosphonium chloride

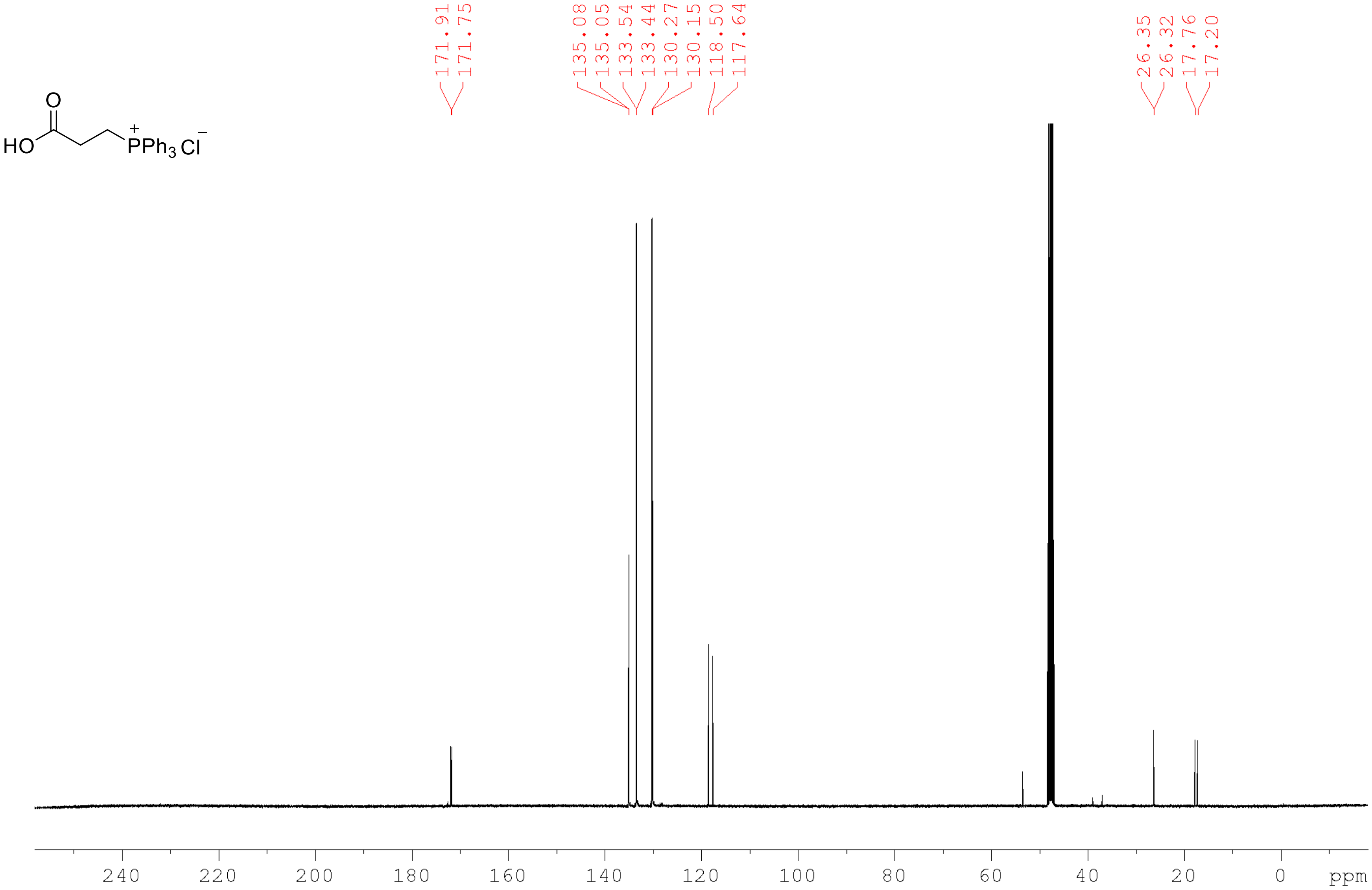

^{31}P NMR (162 MHz, $CDCl_3$) (2-carboxyethyl)triphenylphosphonium chloride

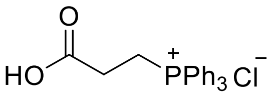

24.30

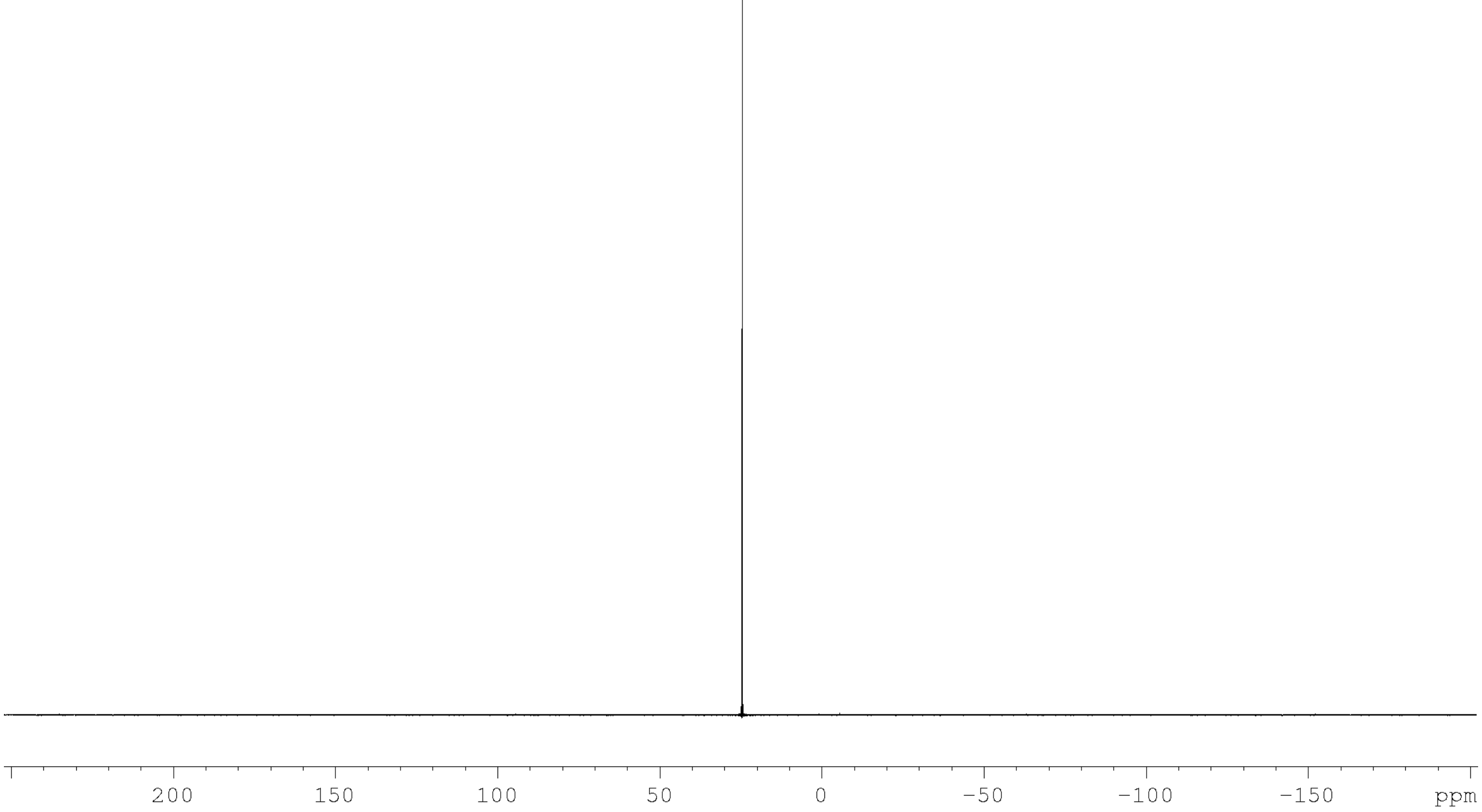

^{1}H NMR (400 MHz, $CDCl_3$) methyl (E)-4-(3,4-dichlorophenyl)but-3-enoate

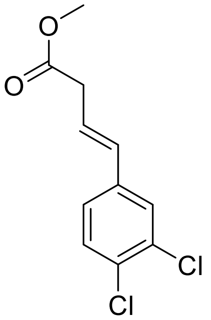

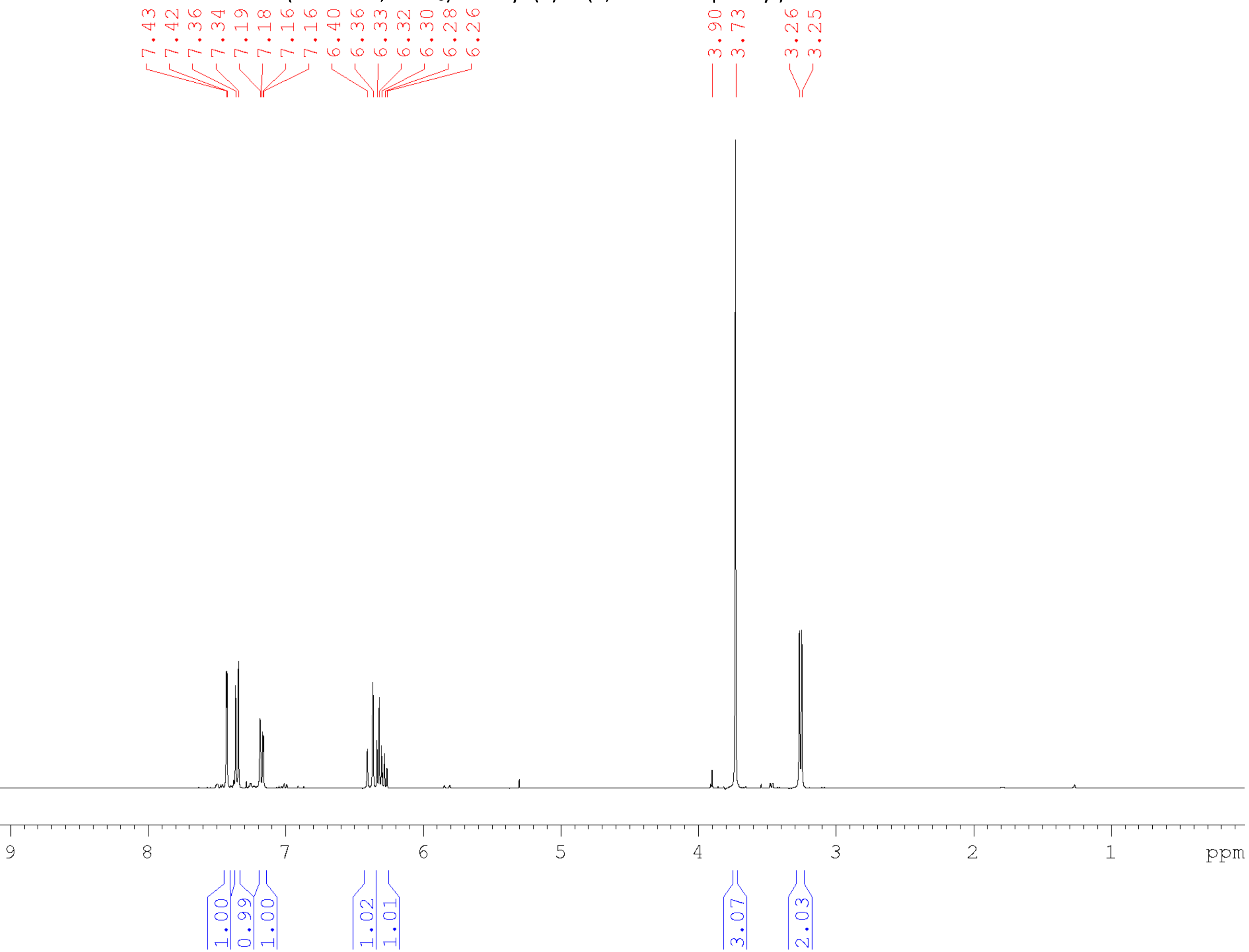

^{13}C NMR (101 MHz, $CDCl_3$) methyl (E)-4-(3,4-dichlorophenyl)but-3-enoate

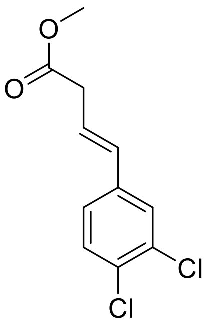

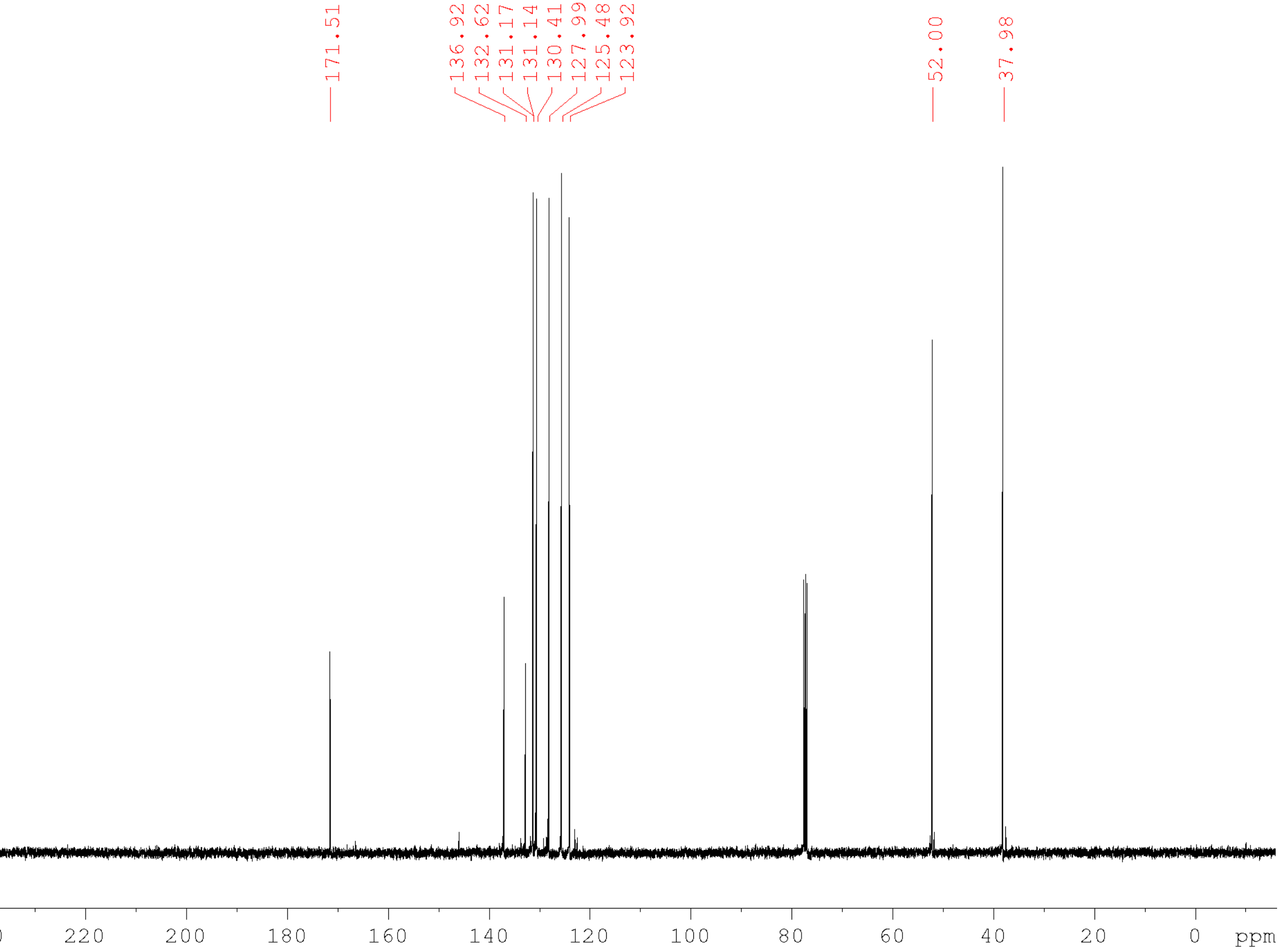

^{1}H NMR (400 MHz, $CDCl_3$) (E)-4-(3,4-dichlorophenyl)but-3-enoic acid

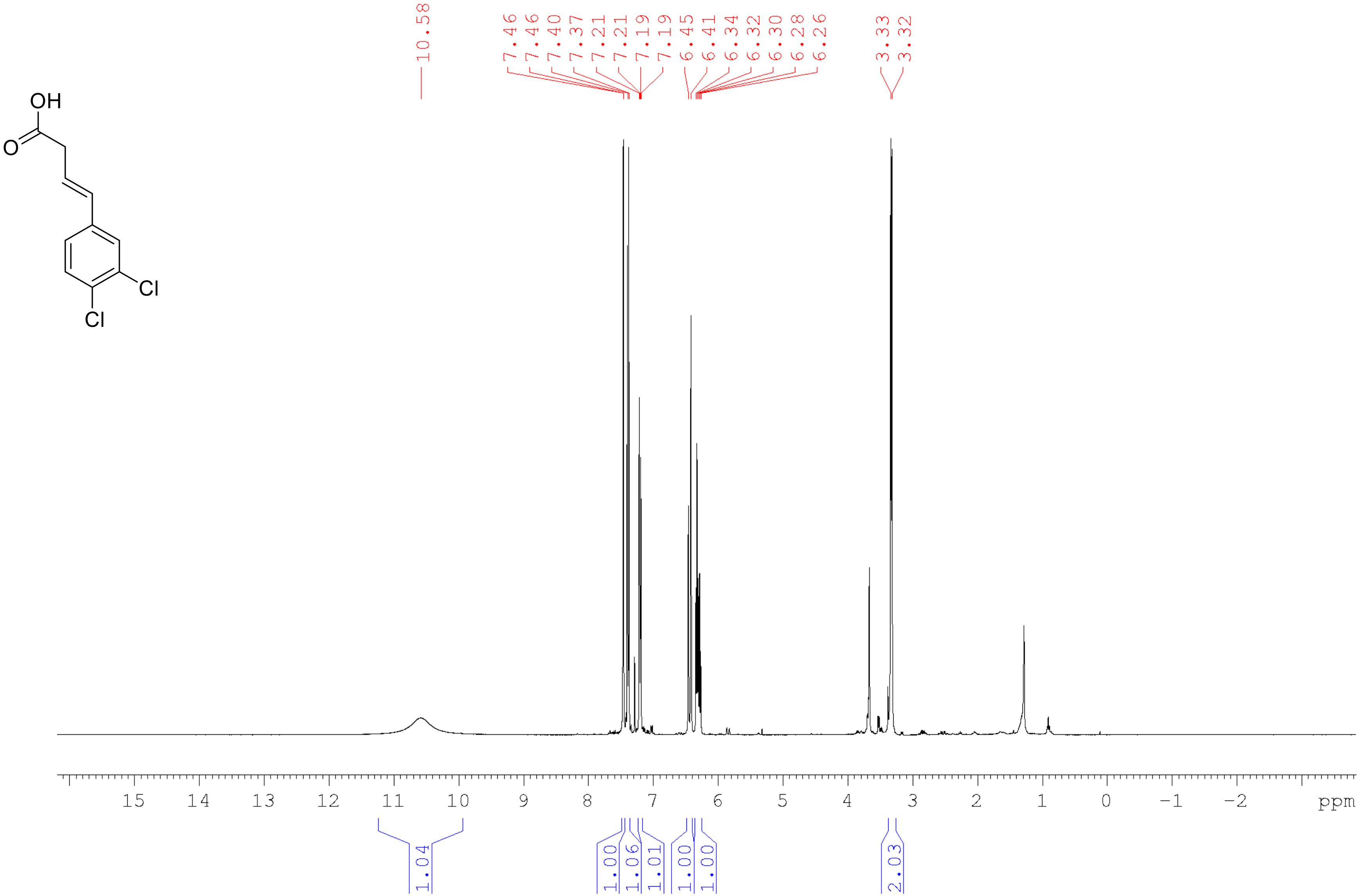

^{13}C NMR (101 MHz, $CDCl_3$) (E)-4-(3,4-dichlorophenyl)but-3-enoic acid

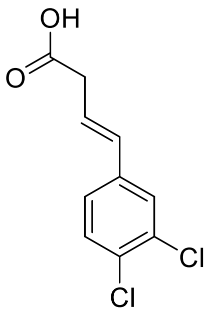

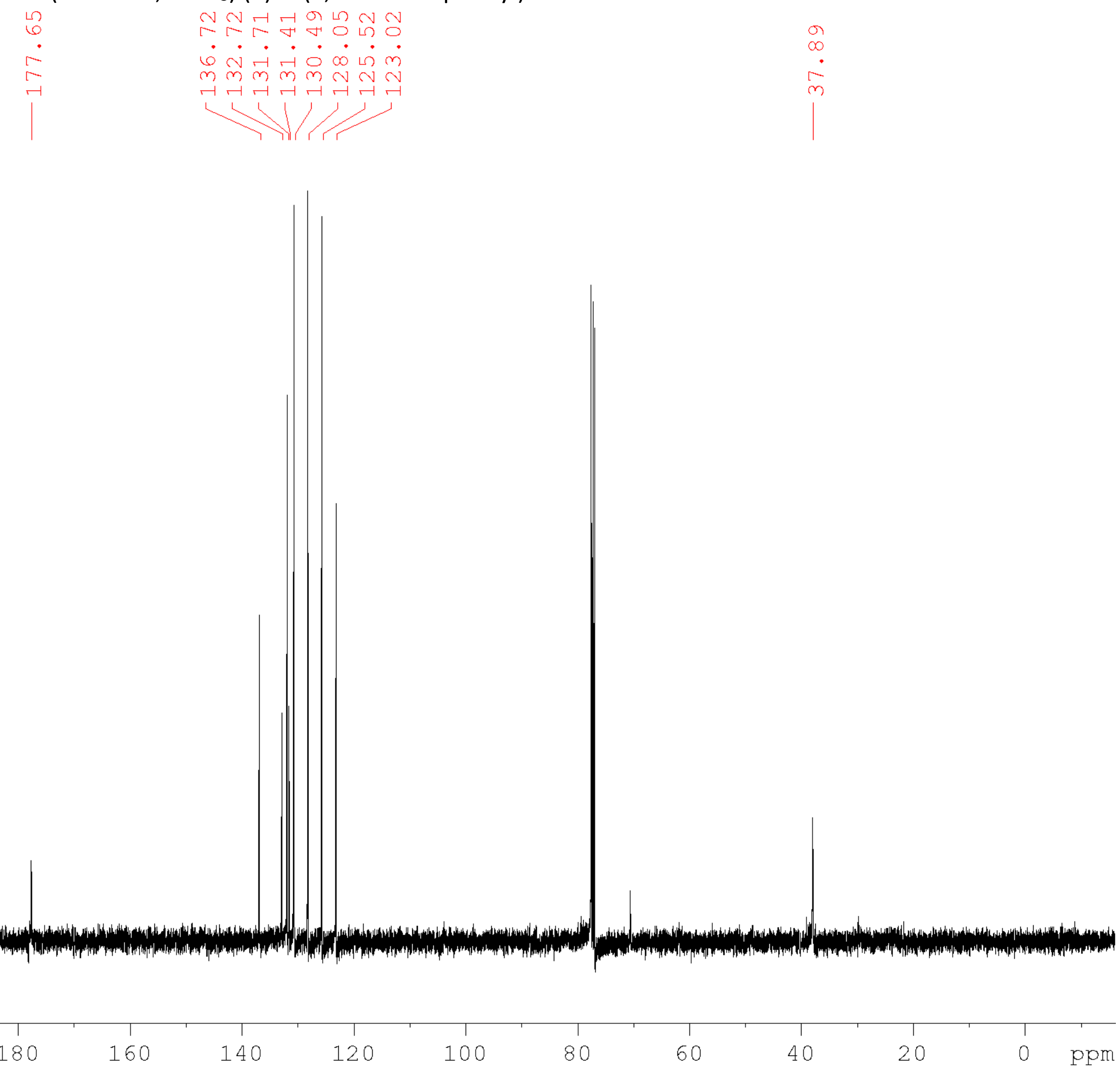

^{1}H NMR (400 MHz, $CDCl_3$) 4-(3,4-dichlorophenyl)butanoic acid

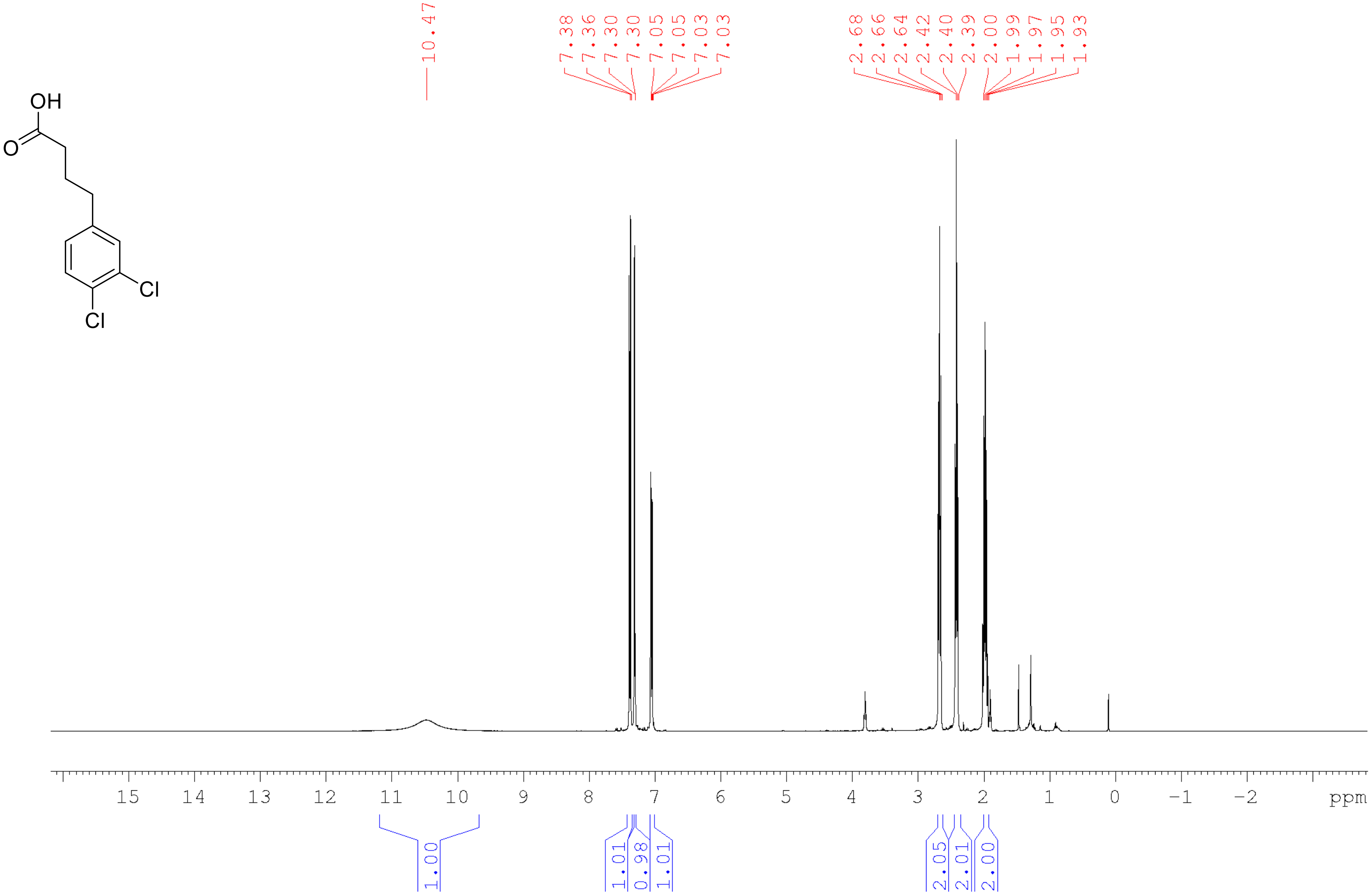

^{13}C NMR (101 MHz, $CDCl_3$) 4-(3,4-dichlorophenyl)butanoic acid

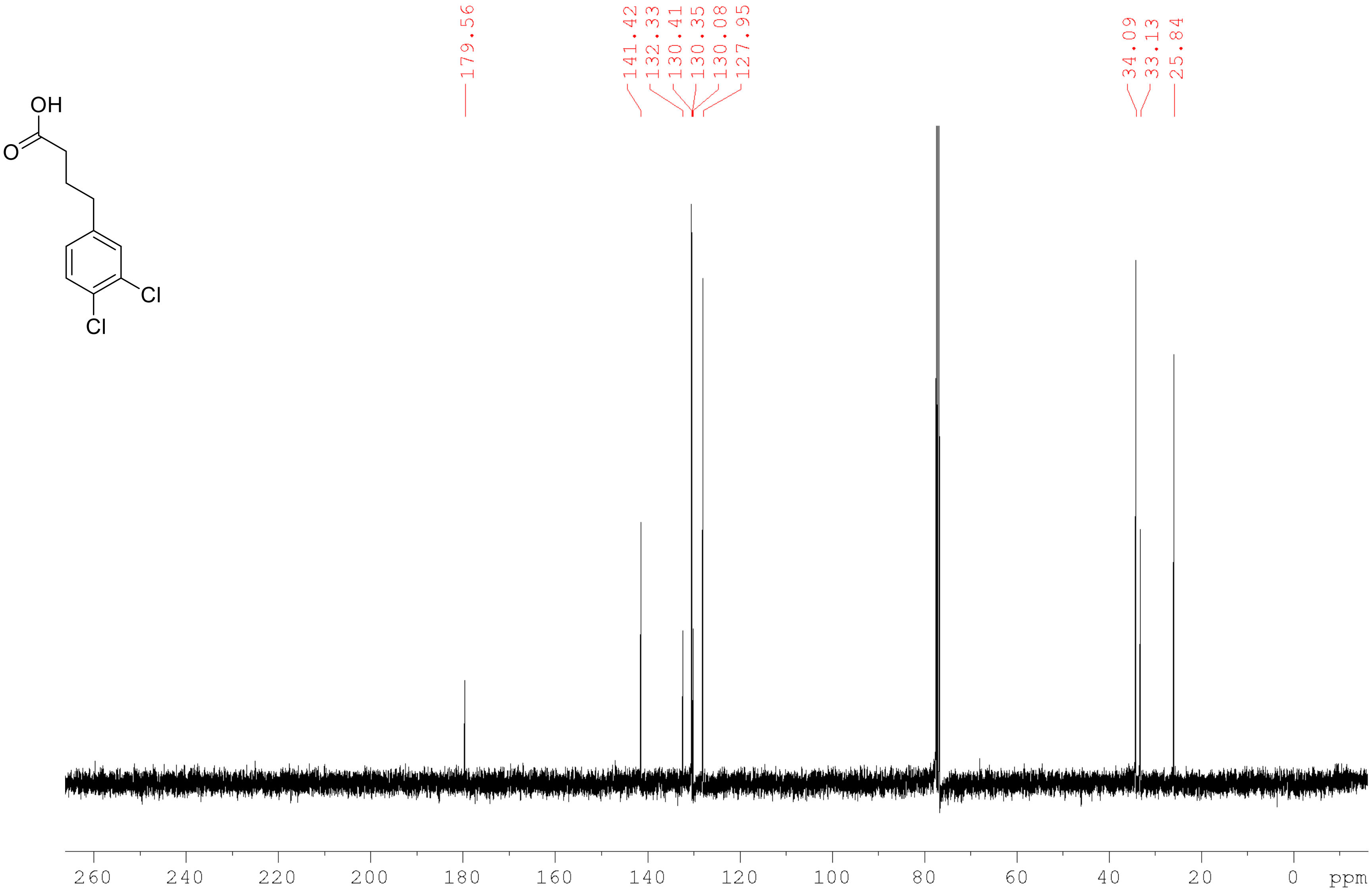

^{1}H NMR (400 MHz, $CDCl_3$) 4,5-dimethylthiazole 3-oxide

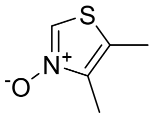

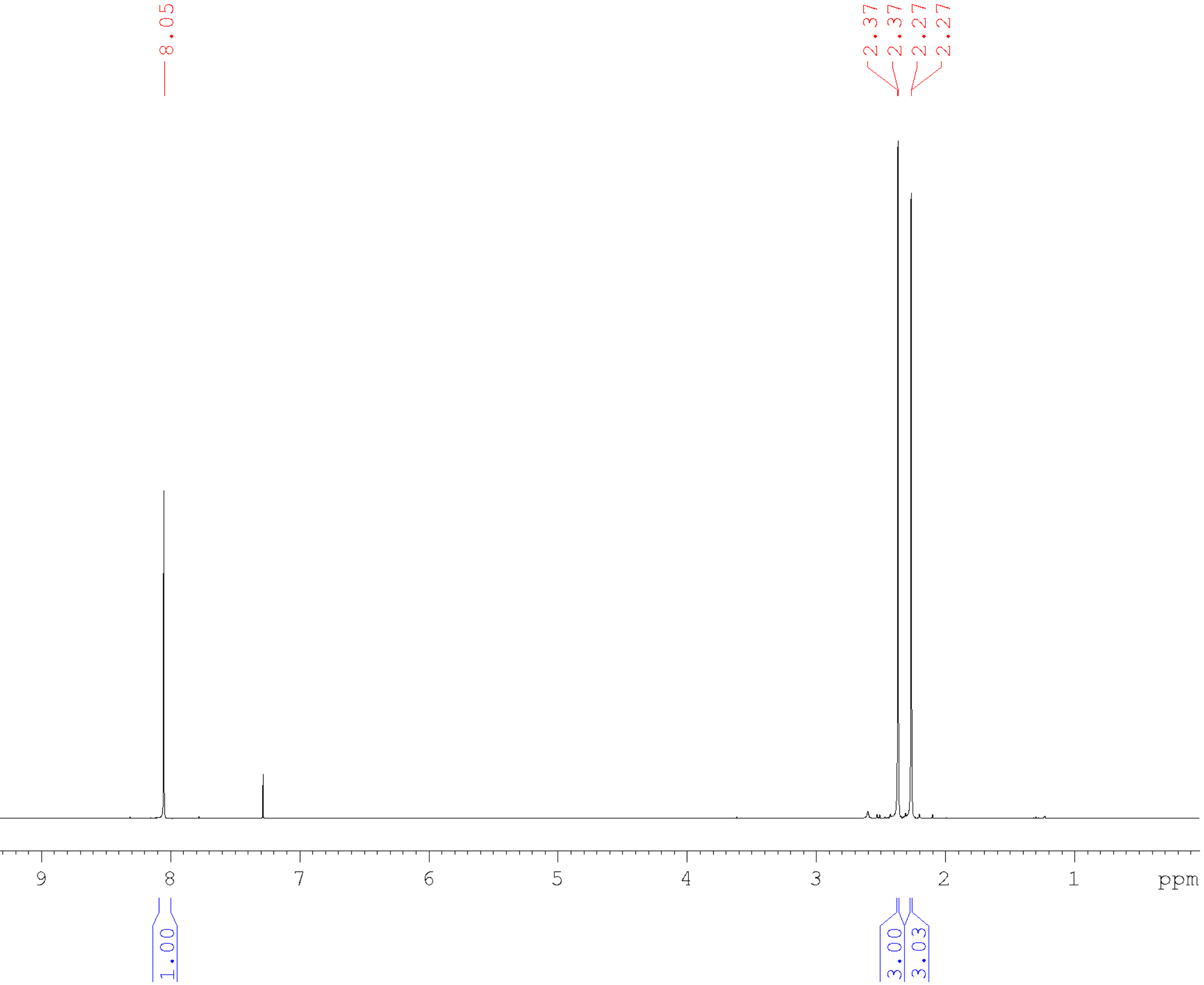

^{13}C NMR (101 MHz, $CDCl_3$) 4,5-dimethylthiazole 3-oxide

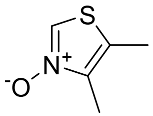

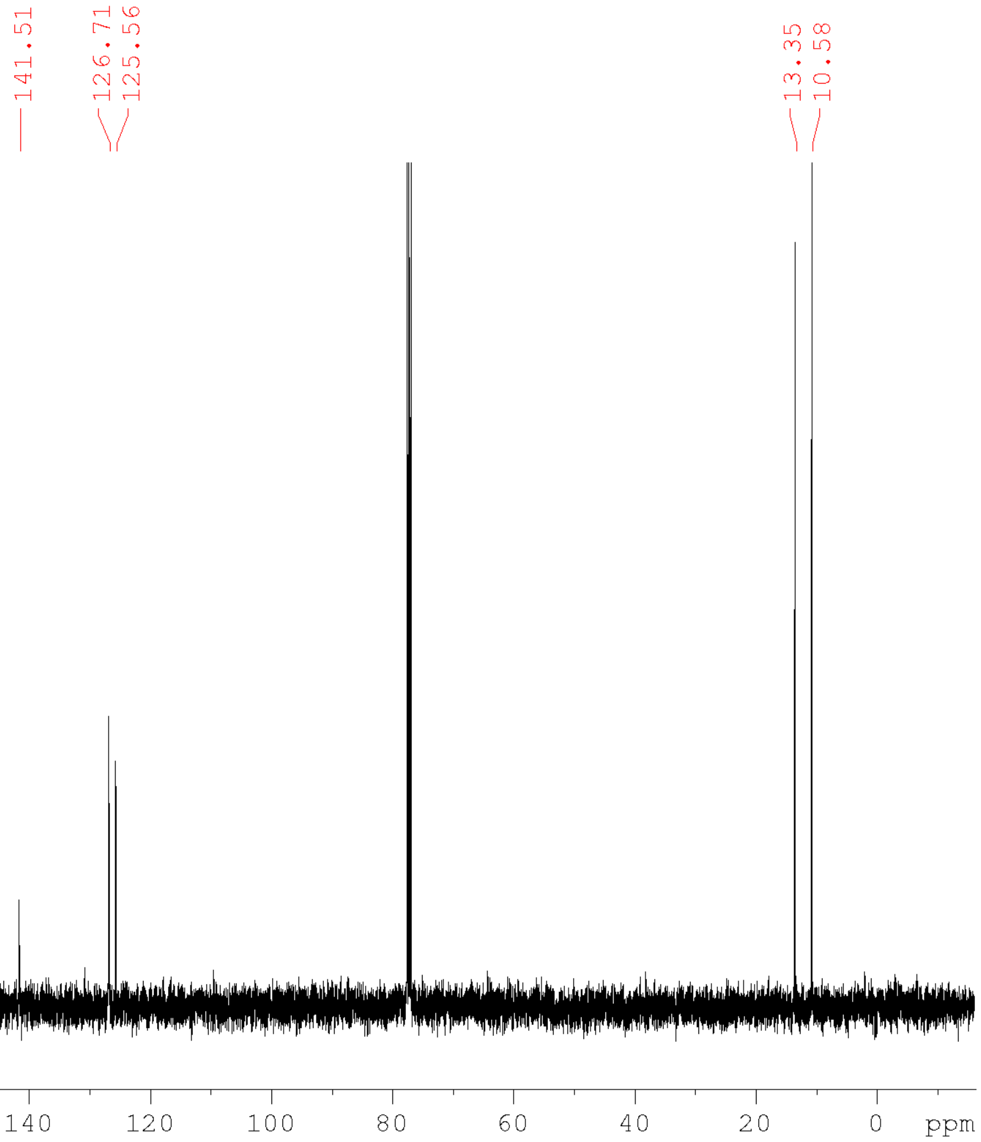

^{1}**H NMR** (400 MHz, $CDCl_3$) pyrazine 1-oxide

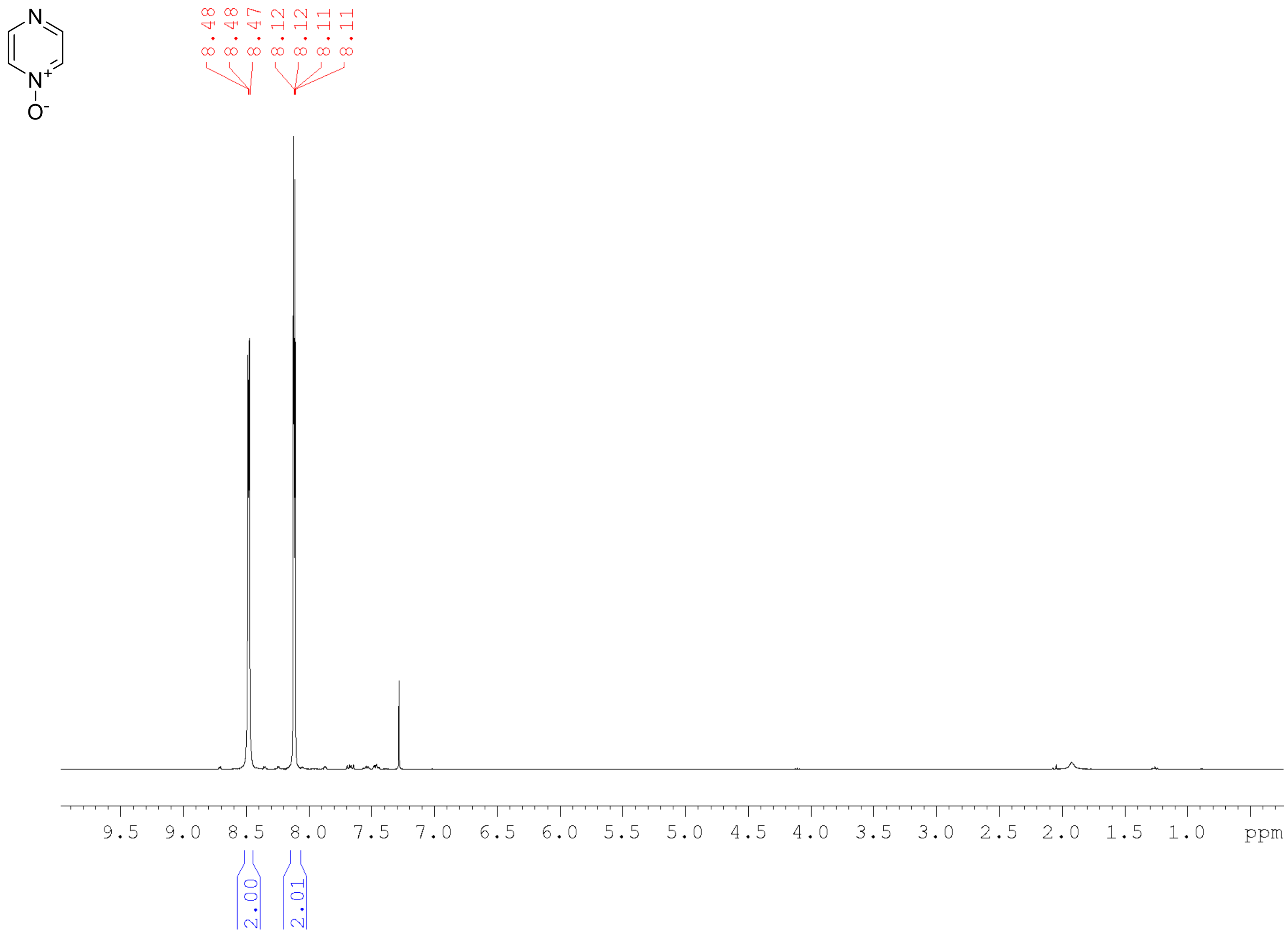

^{13}C NMR (100 MHz, $CDCl_3$) pyrazine 1-oxide

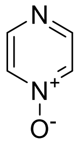

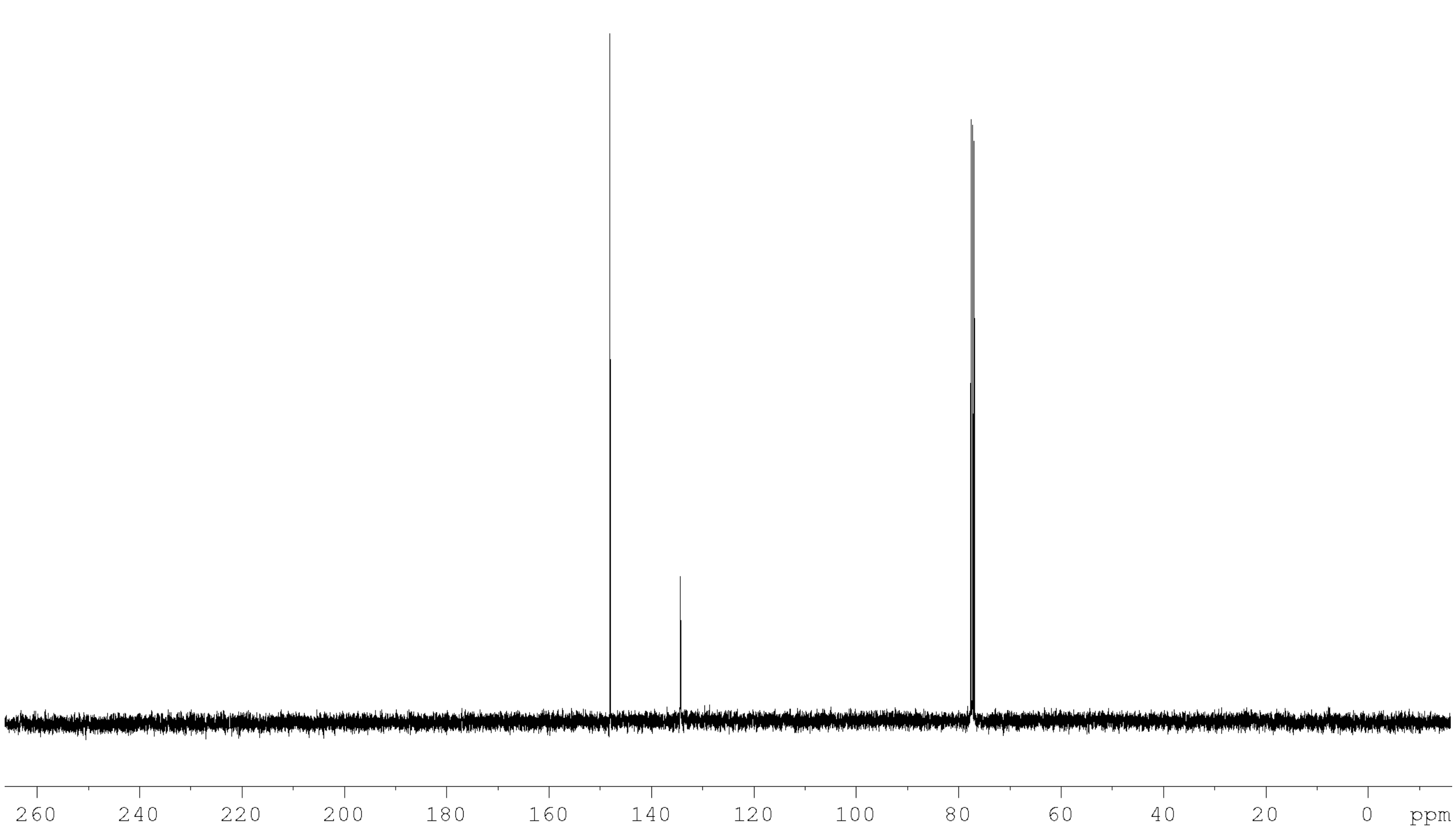

^{1}H NMR (400 MHz, $CDCl_3$) N-(4-chlorobenzyl)-1,1,1-trifluoromethanesulfonamide

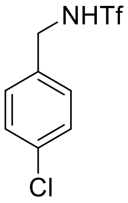

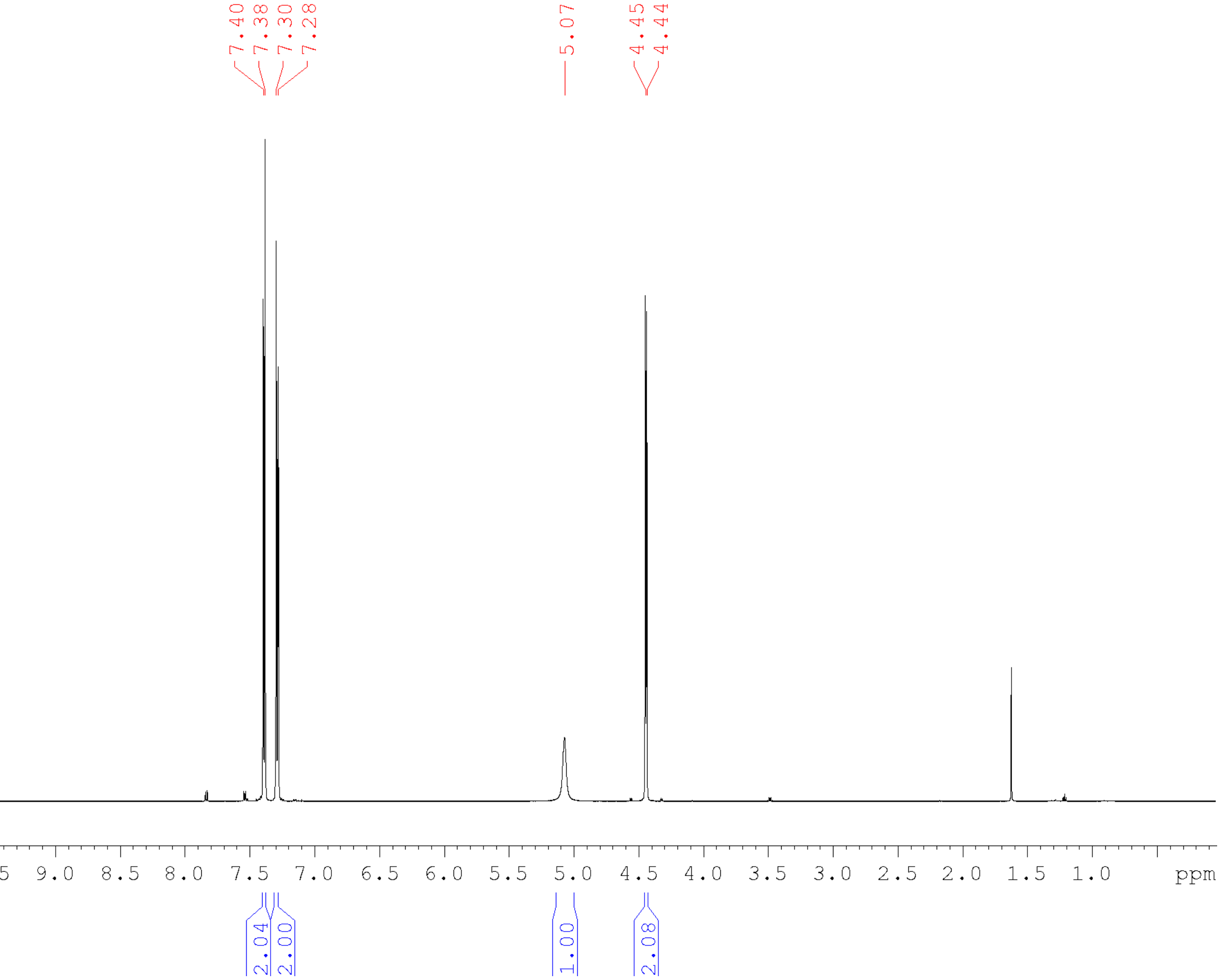

^{13}C NMR (101 MHz, $CDCl_3$) N-(4-chlorobenzyl)-1,1,1-trifluoromethanesulfonamide

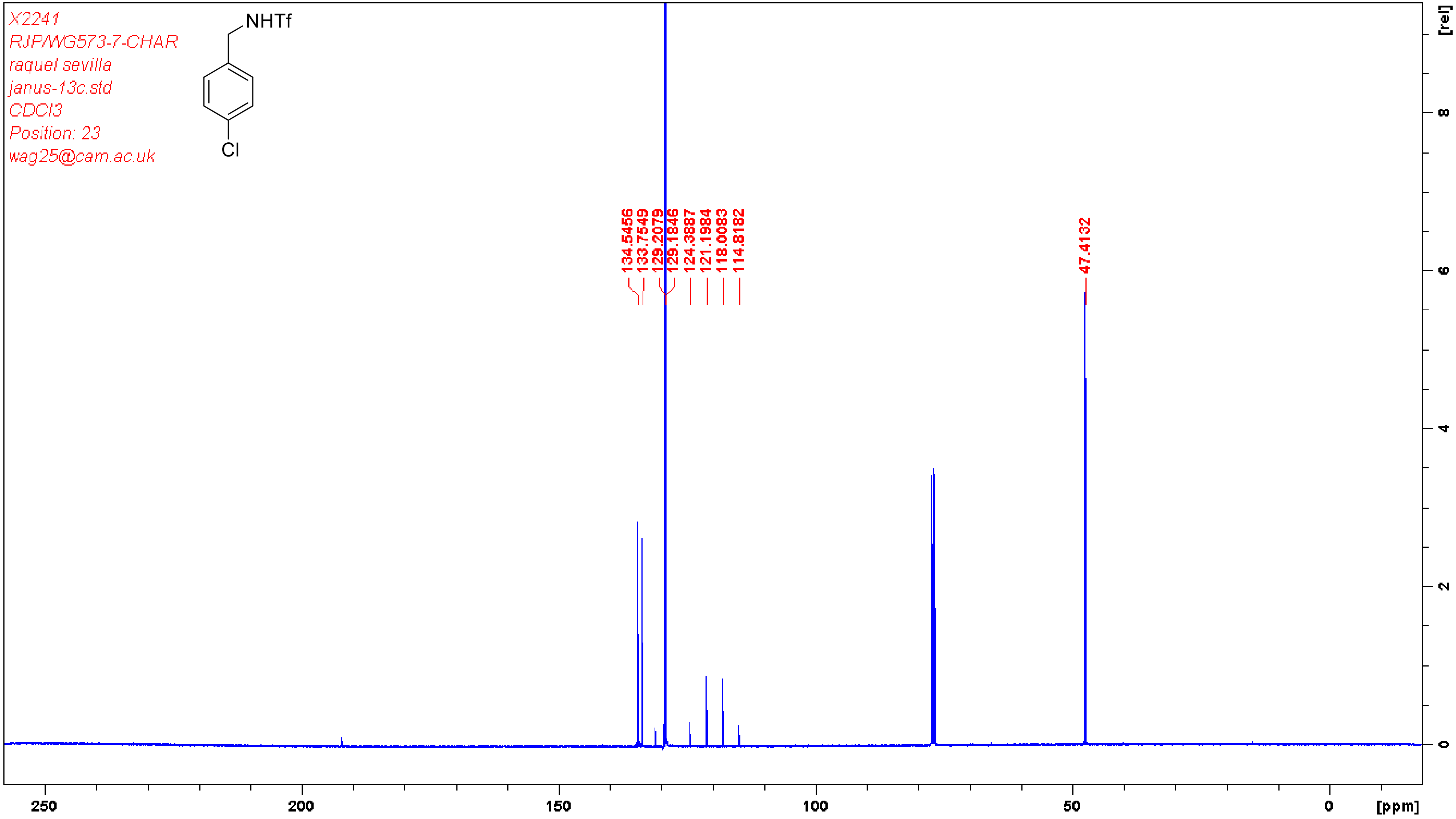

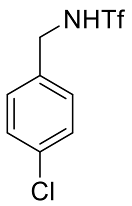

^{19}F NMR (376 MHz, $CDCl_3$) N-(4-chlorobenzyl)-1,1,1-trifluoromethanesulfonamide

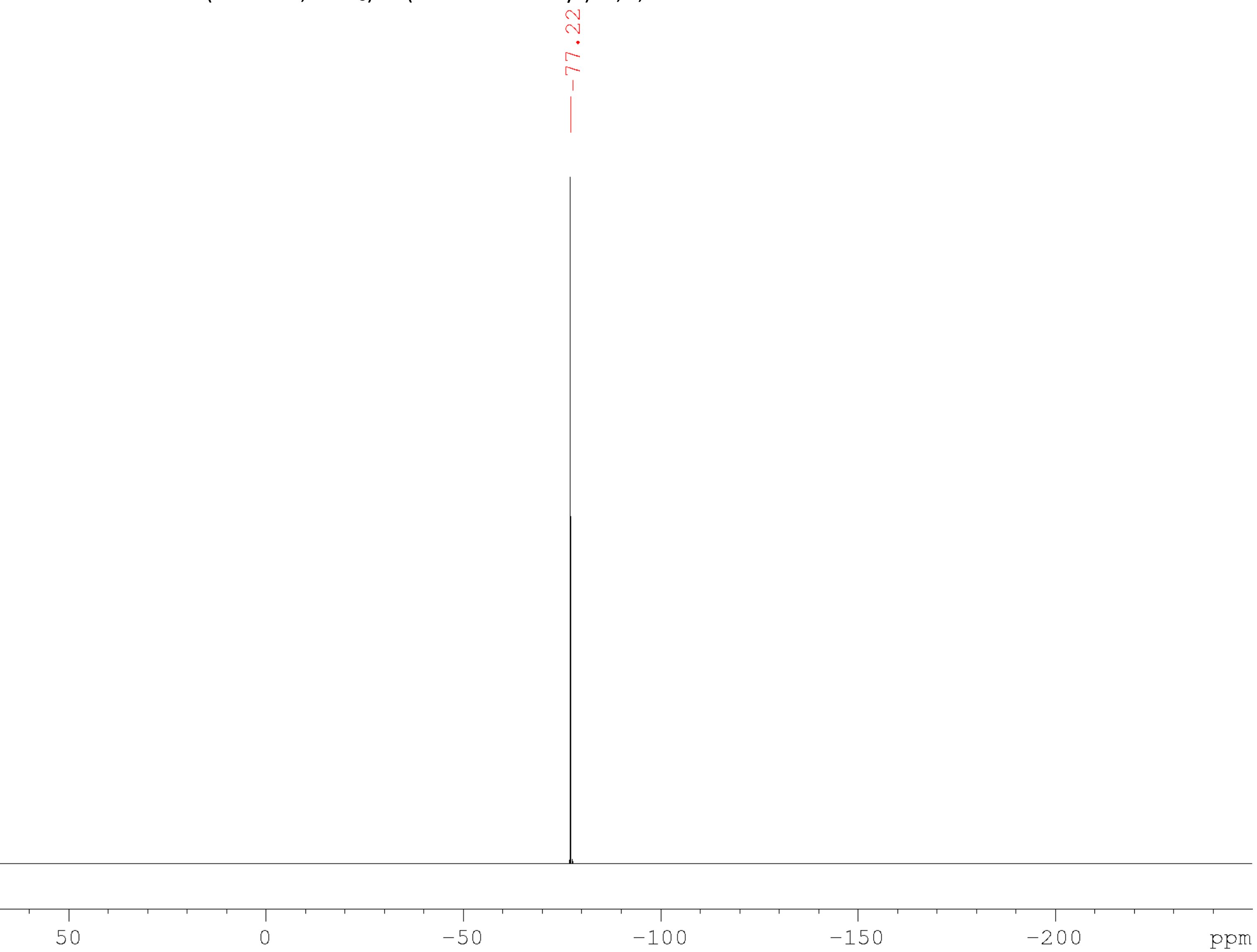

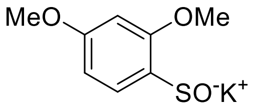

^{1}H NMR (600 MHz, D_2O) Potassium 2,4-dimethoxybenzenesulfonate

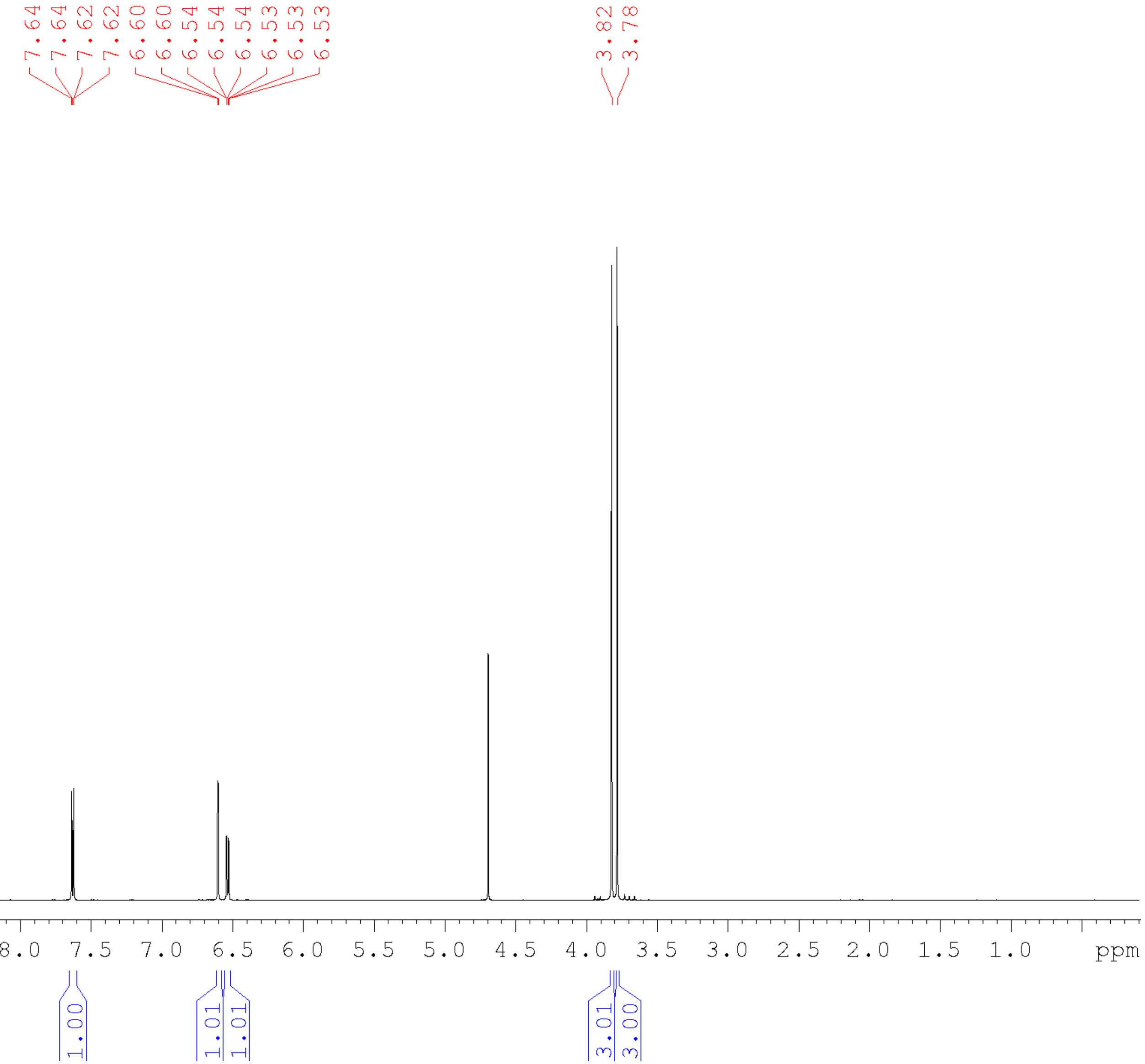

^{13}C NMR (101 MHz, D_2O) Potassium 2,4-dimethoxybenzenesulfonate

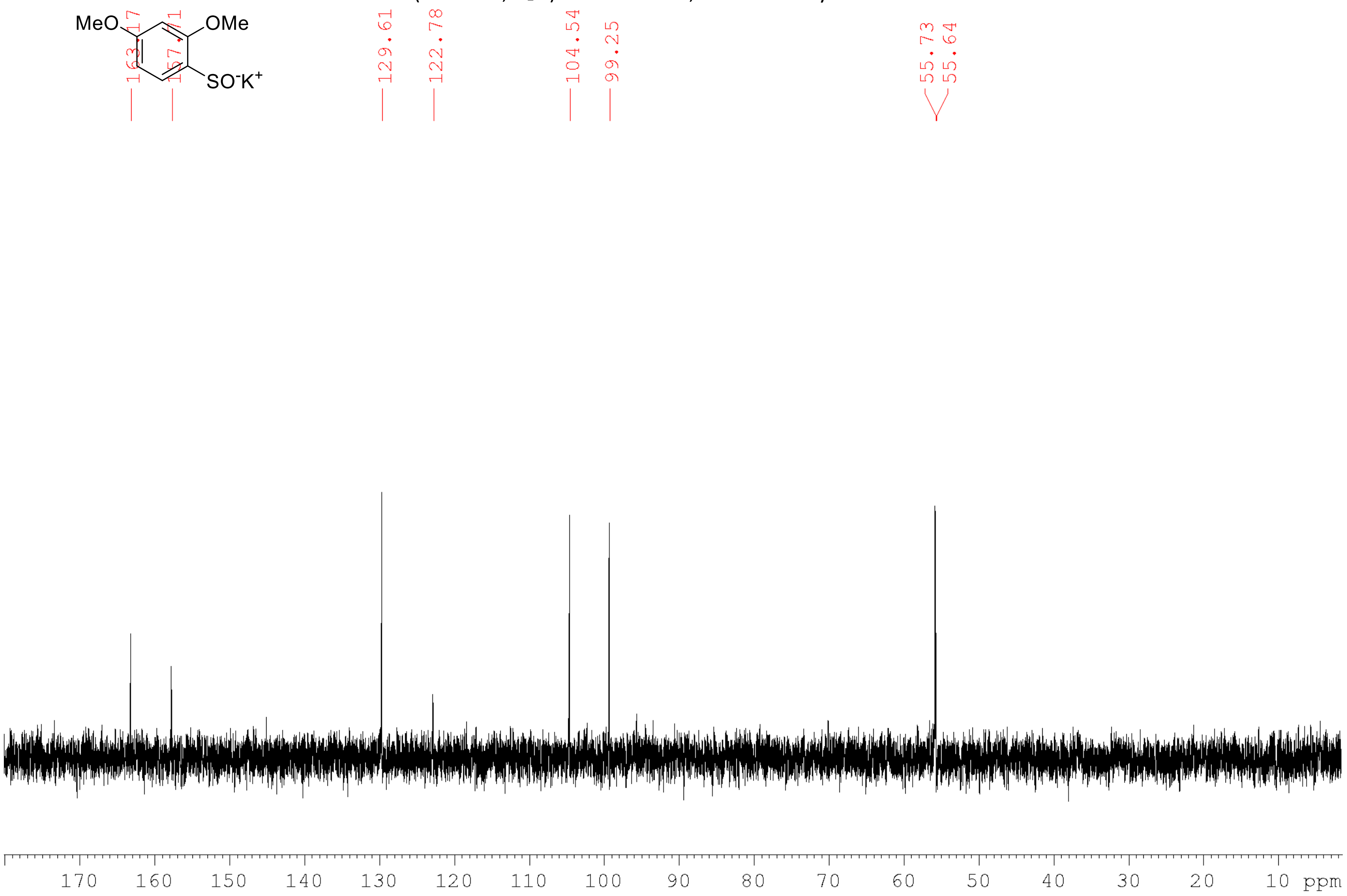

Fluorinated Arene scope

¹H NMR (400 MHZ, $CDCl_3$) **(3a)** N-((6-chloro-2',3',4',5',6'-pentafluoro-[1,1'-biphenyl]-3-yl)methyl)-1,1,1-trifluoromethanesulfonamide

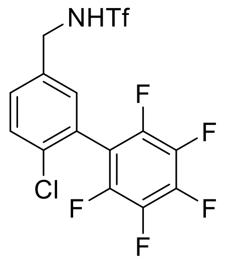

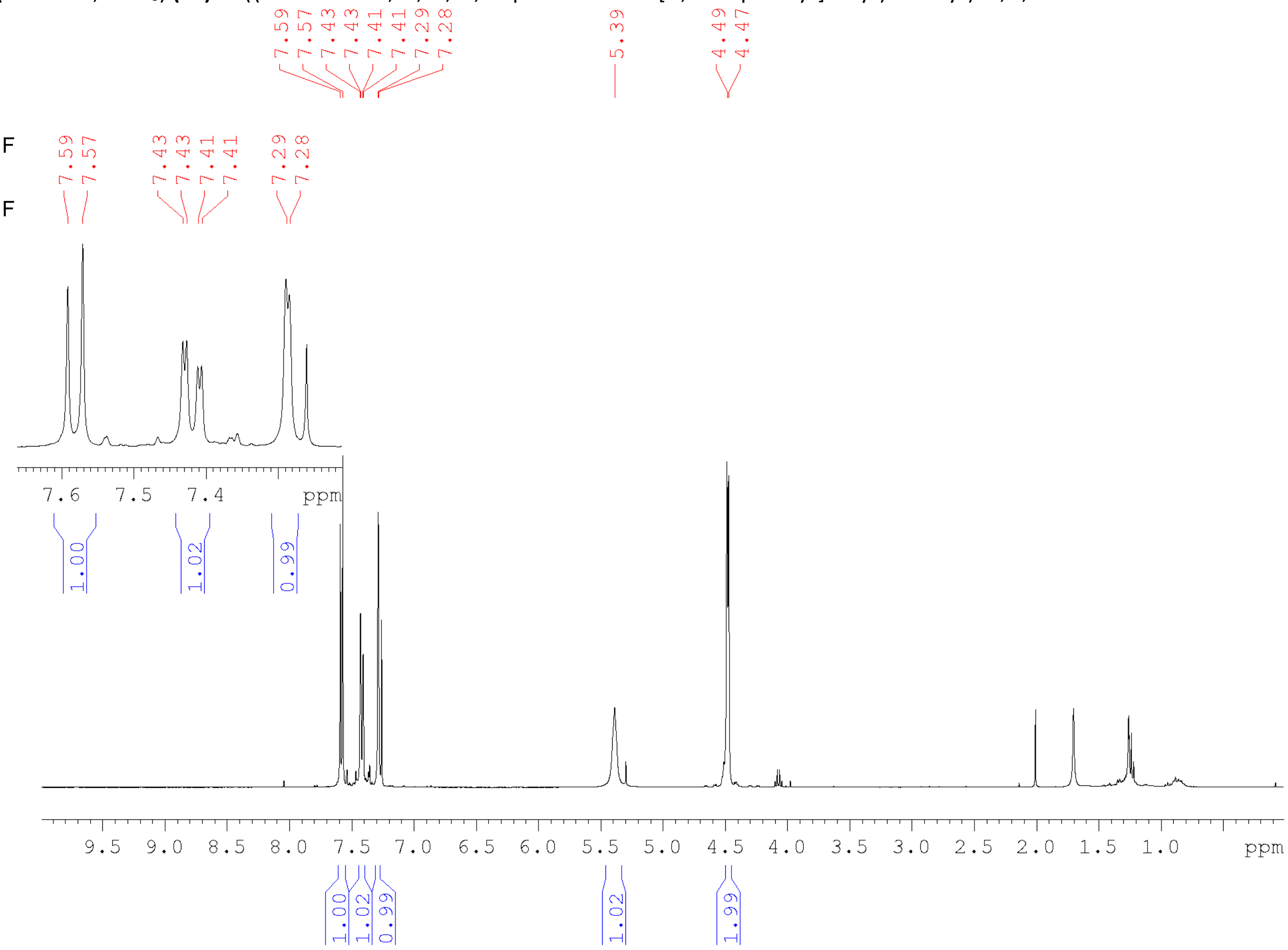

^{13}C NMR (101 MHZ, $CDCl_3$) **(3a)** N-((6-chloro-2',3',4',5',6'-pentafluoro-[1,1'-biphenyl]-3-yl)methyl)-1,1,1-trifluoromethanesulfonamide

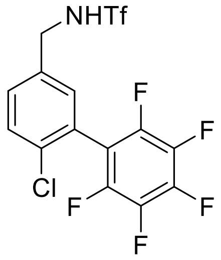

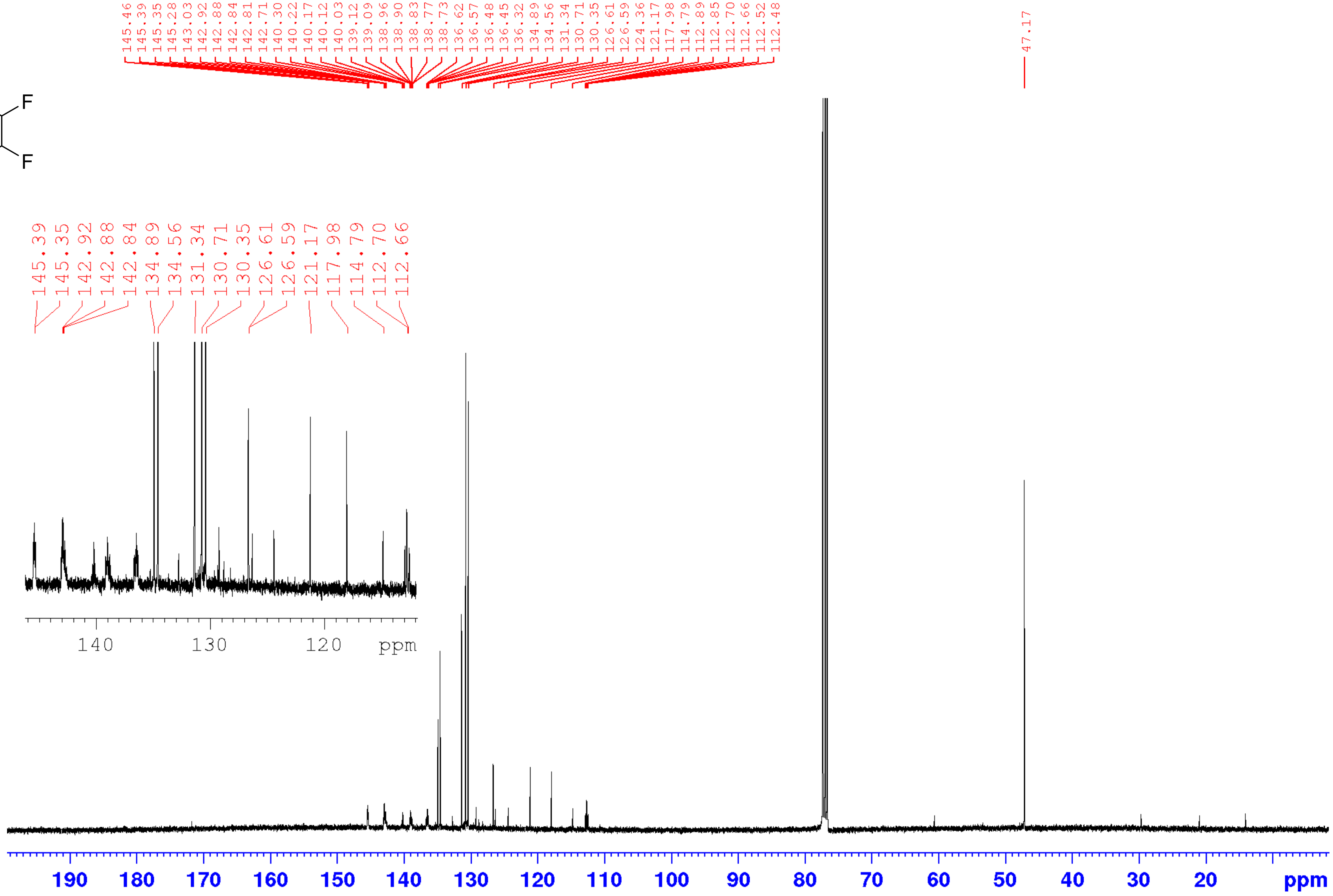

^{19}F NMR (376 MHZ, $CDCl_3$) **(3a)** N-((6-chloro-2',3',4',5',6'-pentafluoro-[1,1'-biphenyl]-3-yl)methyl)-1,1,1-trifluoromethanesulfonamide

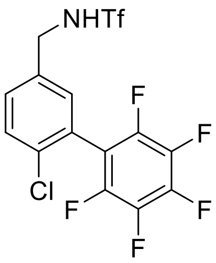

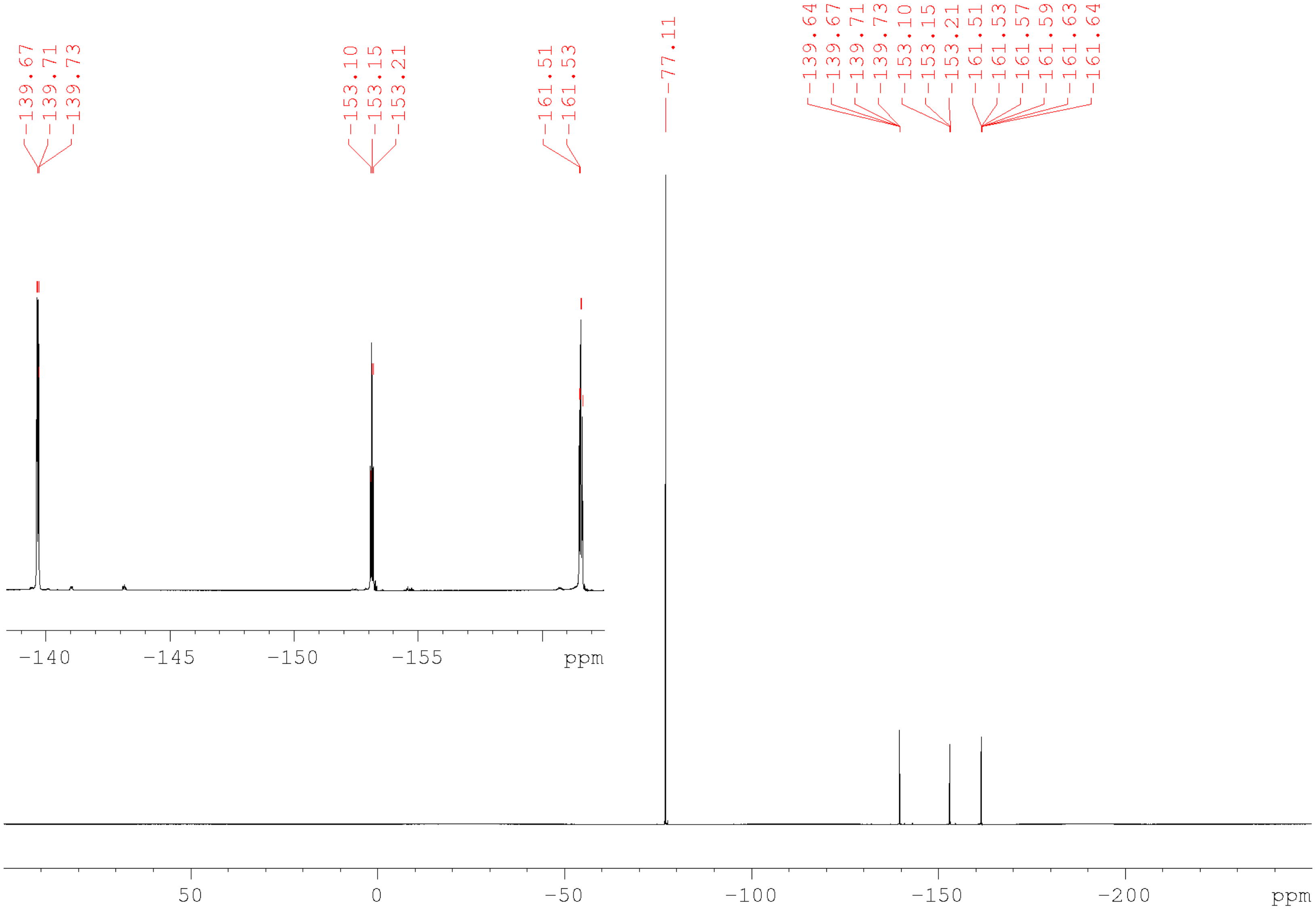

[1]**H NMR** (400 MHZ, $CDCl_3$) **(4a)** N-((2-chloro-2',3',4',5',6'-pentafluoro-[1,1'-biphenyl]-4-yl)methyl)-1,1,1-trifluoromethanesulfonamide

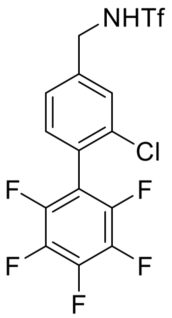

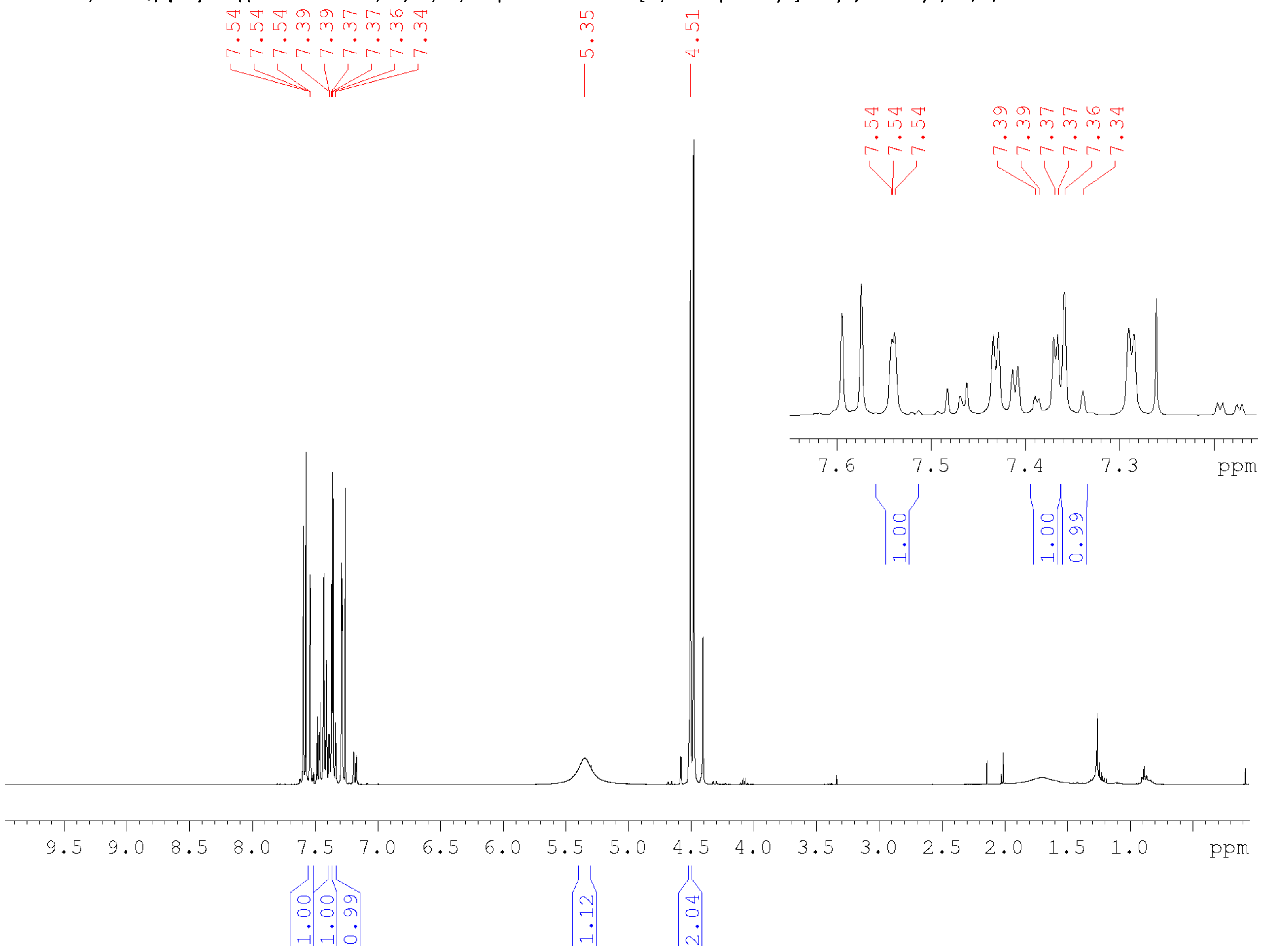

^{1}H NMR (400 MHZ, $CDCl_3$) **(4a)** N-((2-chloro-2',3',4',5',6'-pentafluoro-[1,1'-biphenyl]-4-yl)methyl)-1,1,1-trifluoromethanesulfonamide

(*further analysis – overlaid with meta product (**3a**) + starting material (**1a**)*)

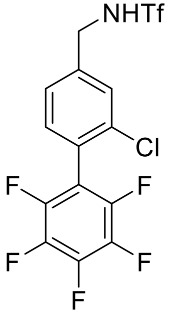

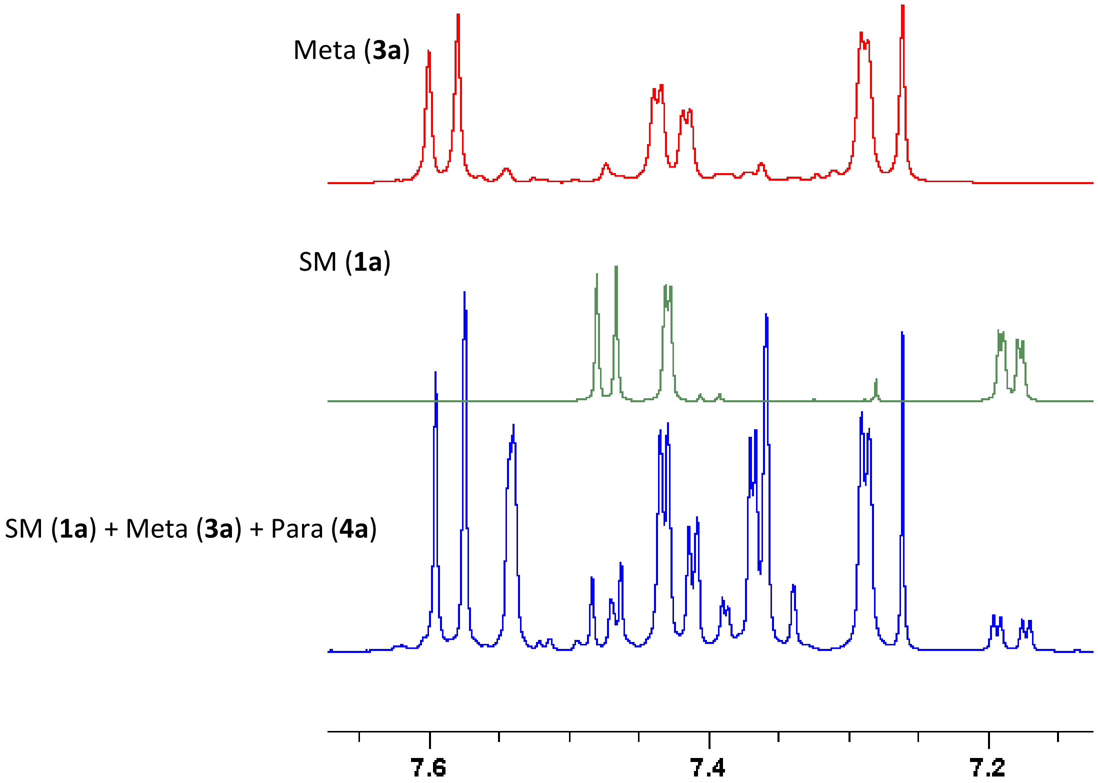

^{13}C NMR (101 MHZ, $CDCl_3$) **(4a)** N-((2-chloro-2',3',4',5',6'-pentafluoro-[1,1'-biphenyl]-4-yl)methyl)-1,1,1-trifluoromethanesulfonamide

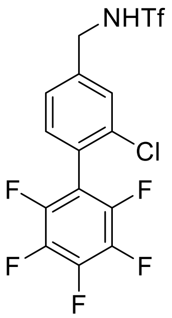

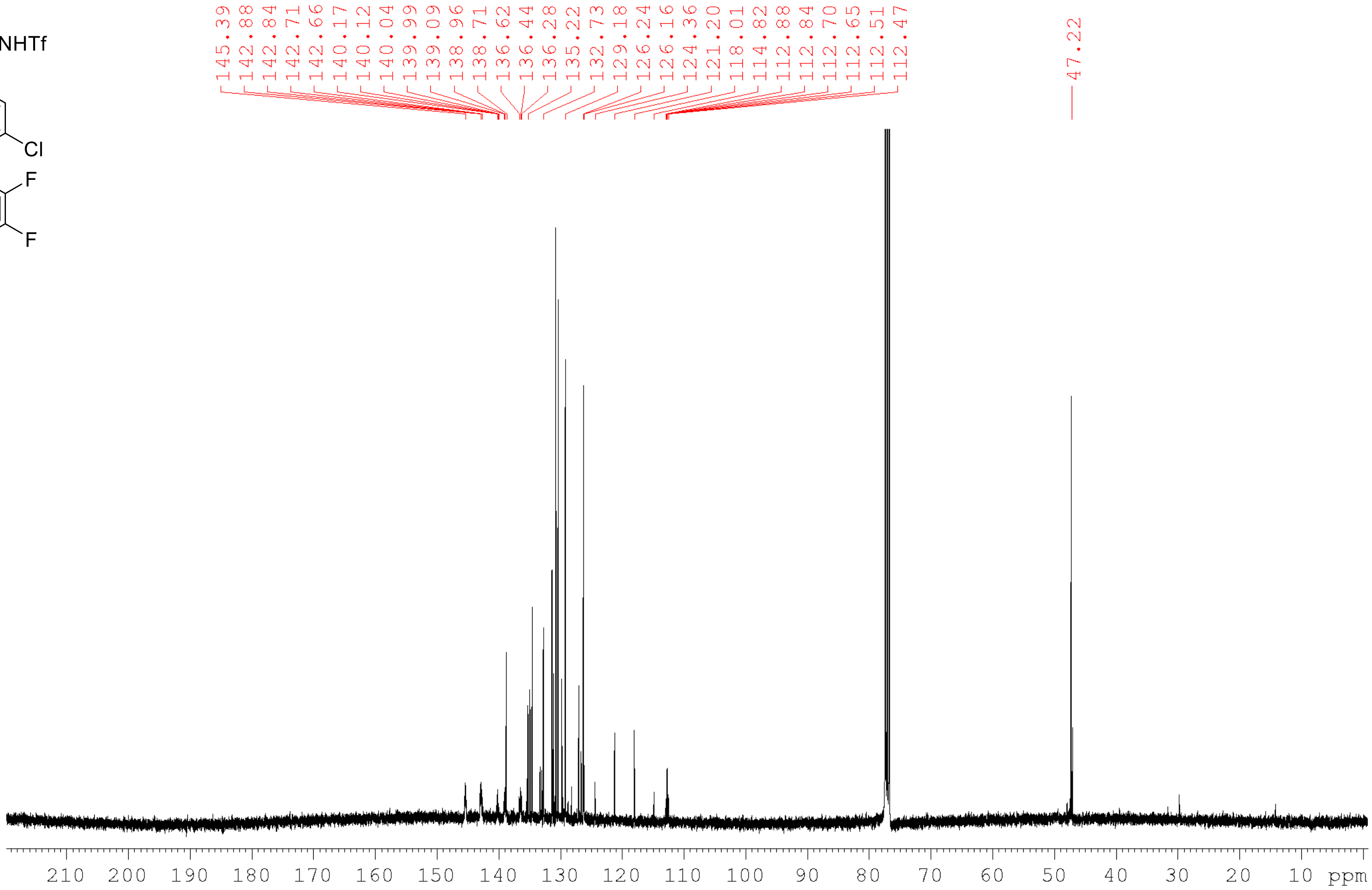

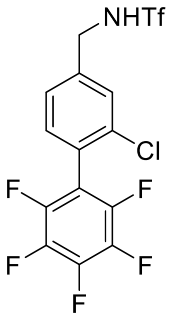

[13]**C NMR** (101 MHZ, $CDCl_3$) **(4a)** N-((2-chloro-2',3',4',5',6'-pentafluoro-[1,1'-biphenyl]-4-yl)methyl)-1,1,1-trifluoromethanesulfonamide

(*further analysis – overlaid with meta product (**3a**) + starting material (**1a**)*)

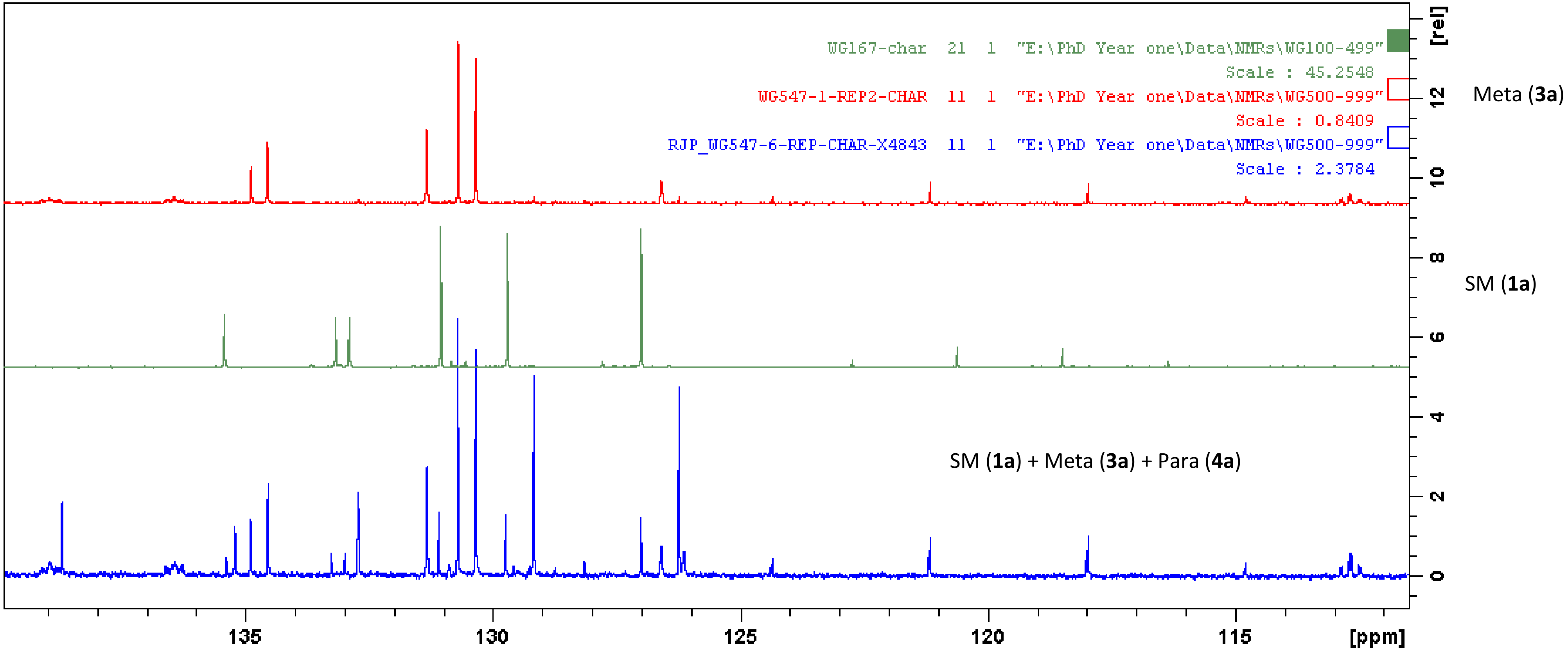

^{19}F NMR (376 MHZ, $CDCl_3$) **(4a)** N-((2-chloro-2',3',4',5',6'-pentafluoro-[1,1'-biphenyl]-4-yl)methyl)-1,1,1-trifluoromethanesulfonamide

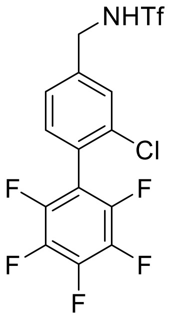

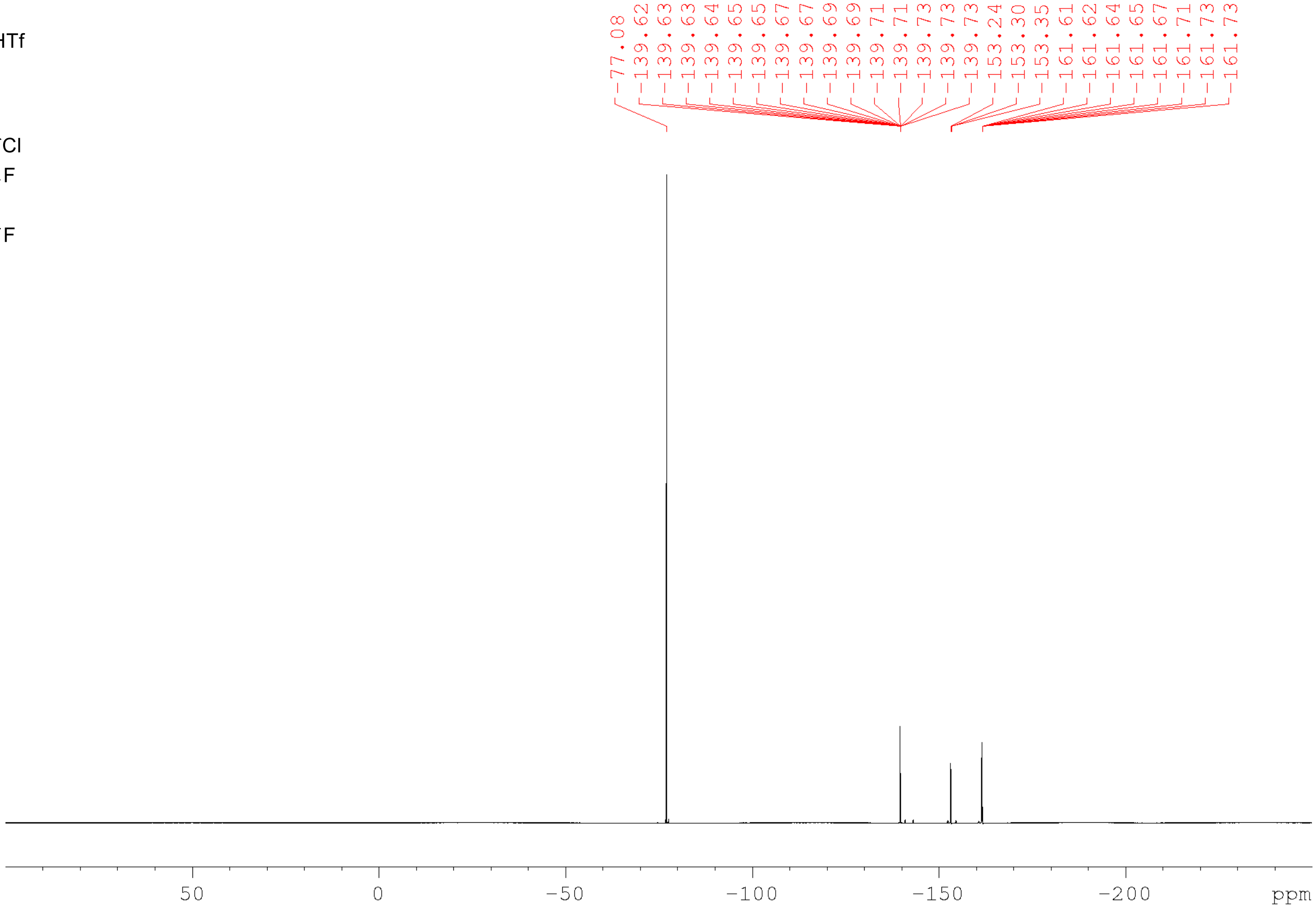

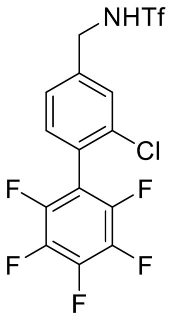

[19]**F NMR** (376 MHZ, $CDCl_3$) **(4a)** N-((2-chloro-2',3',4',5',6'-pentafluoro-[1,1'-biphenyl]-4-yl)methyl)-1,1,1-trifluoromethanesulfonamide

*(further analysis – overlaid with meta product (**3a**) + starting material (**1a**))*

Meta (**3a**)

SM (**1a**)

SM (**1a**) + Meta (**3a**) + Para (**4a**)

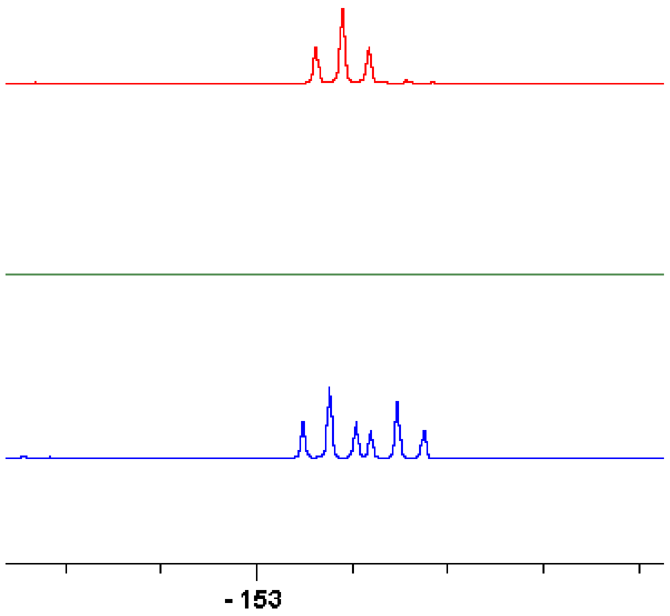

^{1}H NMR (400 MHZ, $CDCl_3$) **(3b)** N-((6-chloro-2',3',4',6'-tetrafluoro-[1,1'-biphenyl]-3-yl)methyl)-1,1,1-trifluoromethanesulfonamide

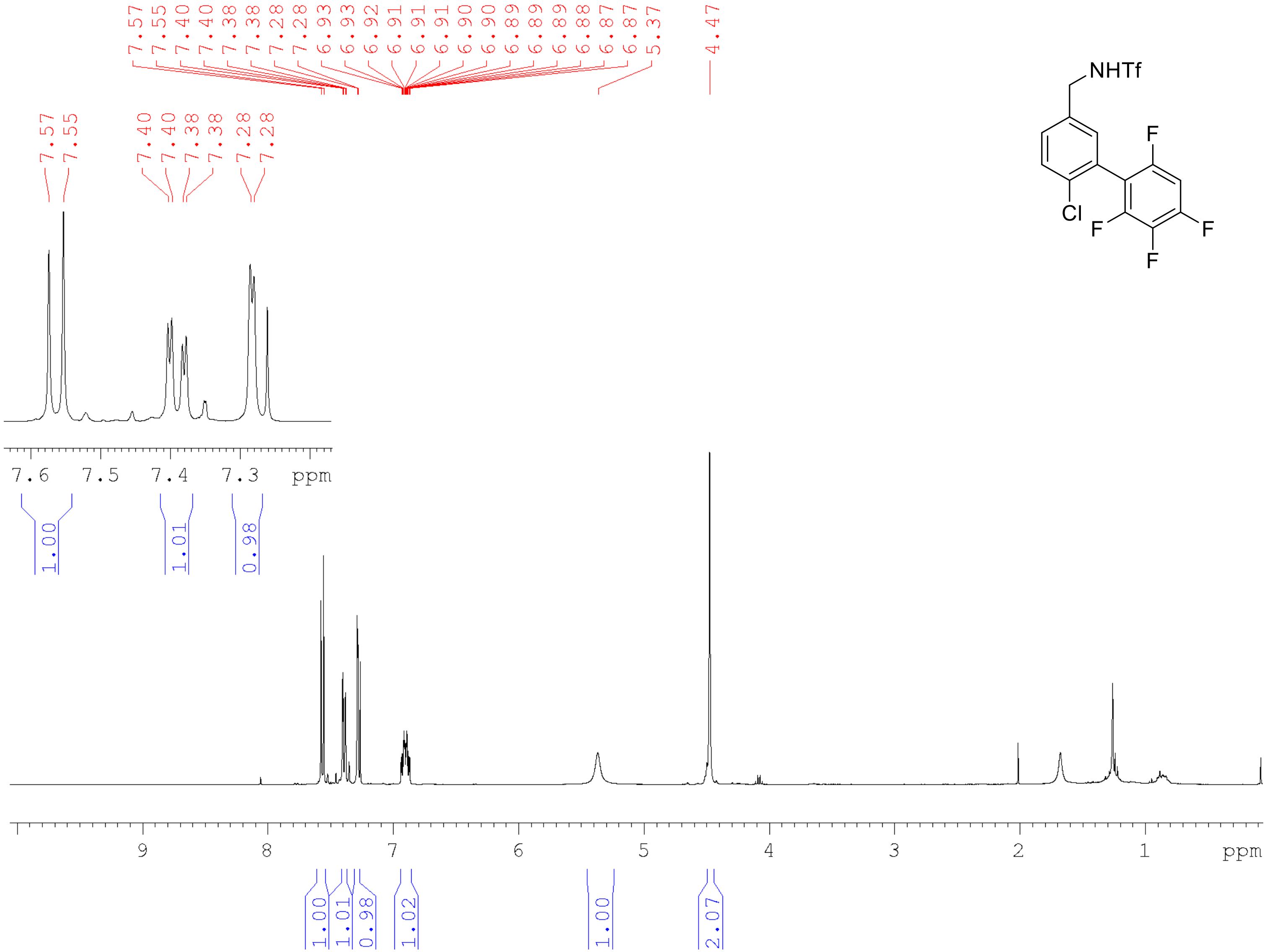

^{13}C NMR (101 MHZ, $CDCl_3$) **(3b)** N-((6-chloro-2',3',4',6'-tetrafluoro-[1,1'-biphenyl]-3-yl)methyl)-1,1,1-trifluoromethanesulfonamide

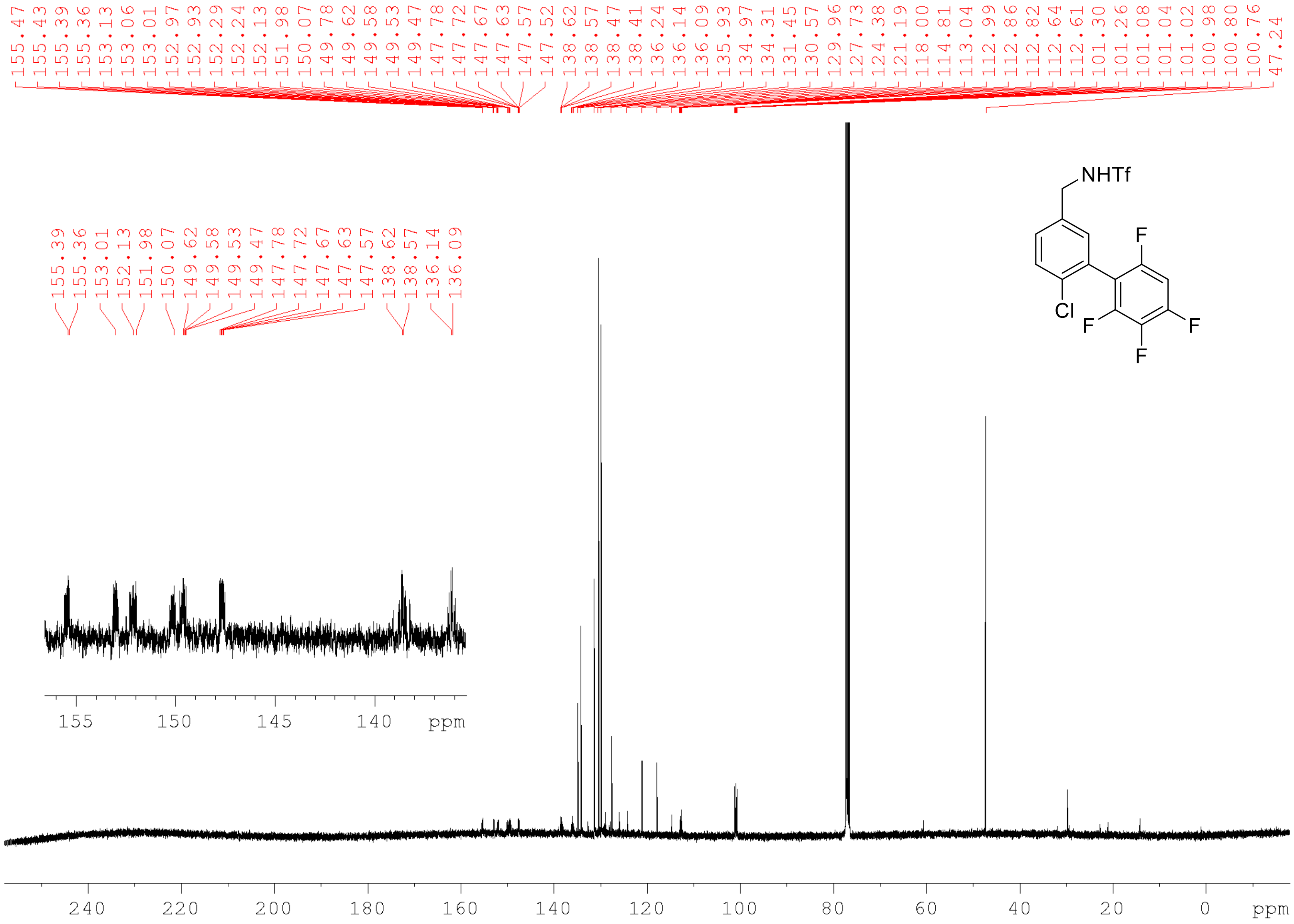

^{19}F NMR (376 MHZ, $CDCl_3$) **(3b)** N-((6-chloro-2',3',4',6'-tetrafluoro-[1,1'-biphenyl]-3-yl)methyl)-1,1,1-trifluoromethanesulfonamide

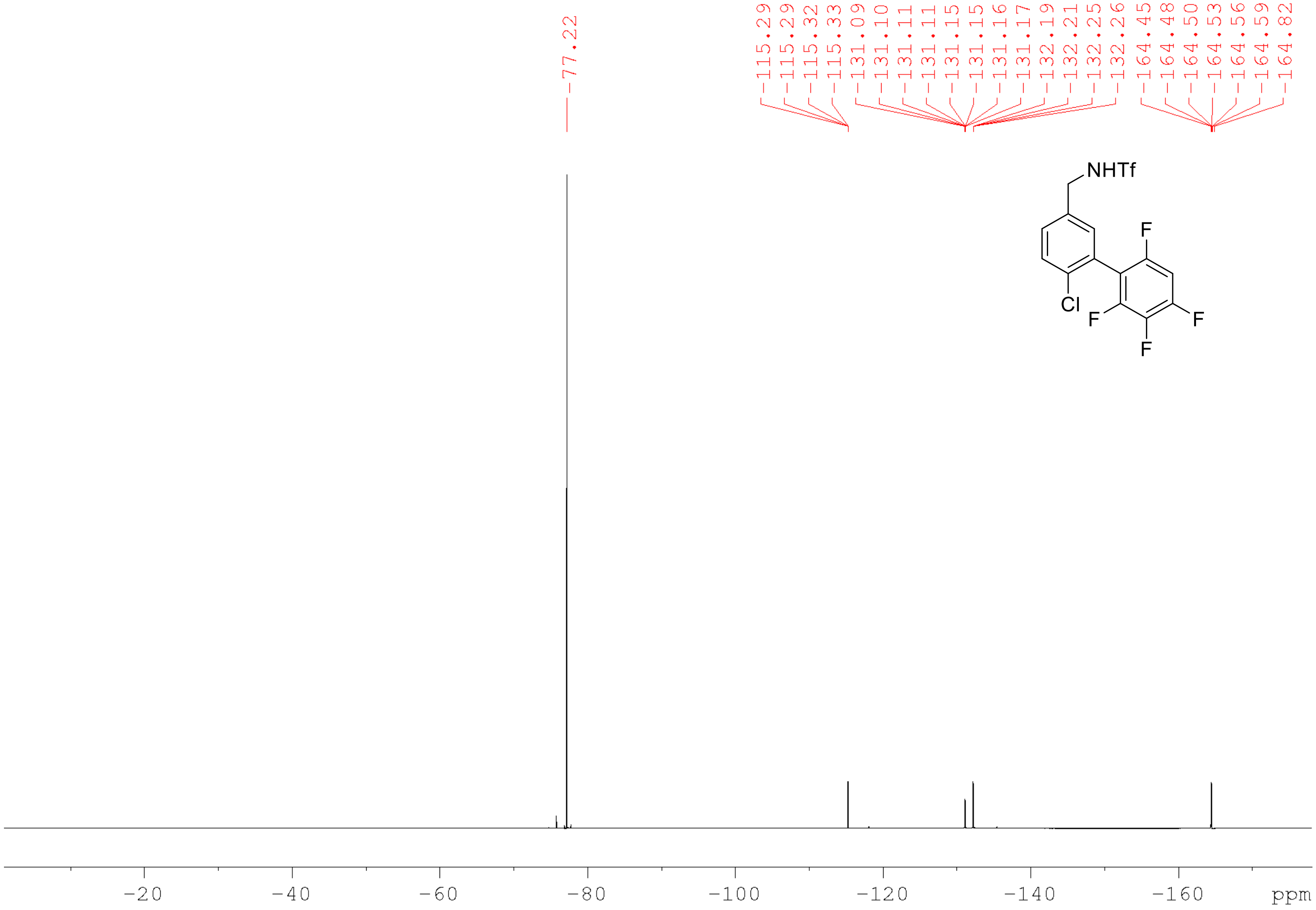

^{1}H NMR (400 MHZ, $CDCl_3$) **(3c)** N-((6-chloro-2',3',5',6'-tetrafluoro-[1,1'-biphenyl]-3-yl)methyl)-1,1,1-trifluoromethanesulfonamide

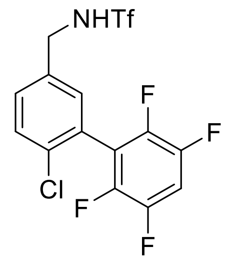

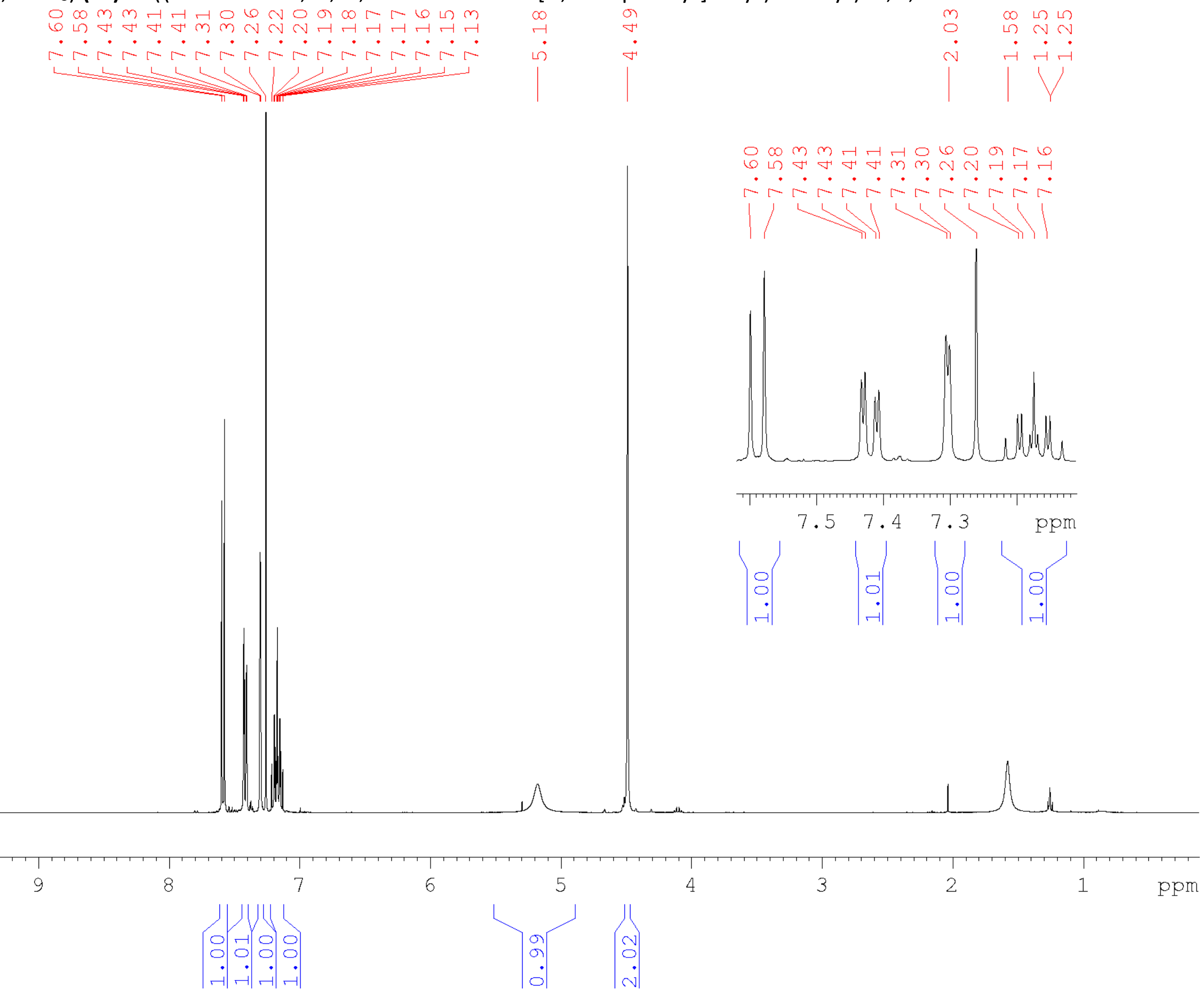

^{13}C NMR (101 MHZ, $CDCl_3$) **(3c)** N-((6-chloro-2',3',5',6'-tetrafluoro-[1,1'-biphenyl]-3-yl)methyl)-1,1,1-trifluoromethanesulfonamide

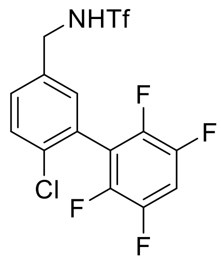

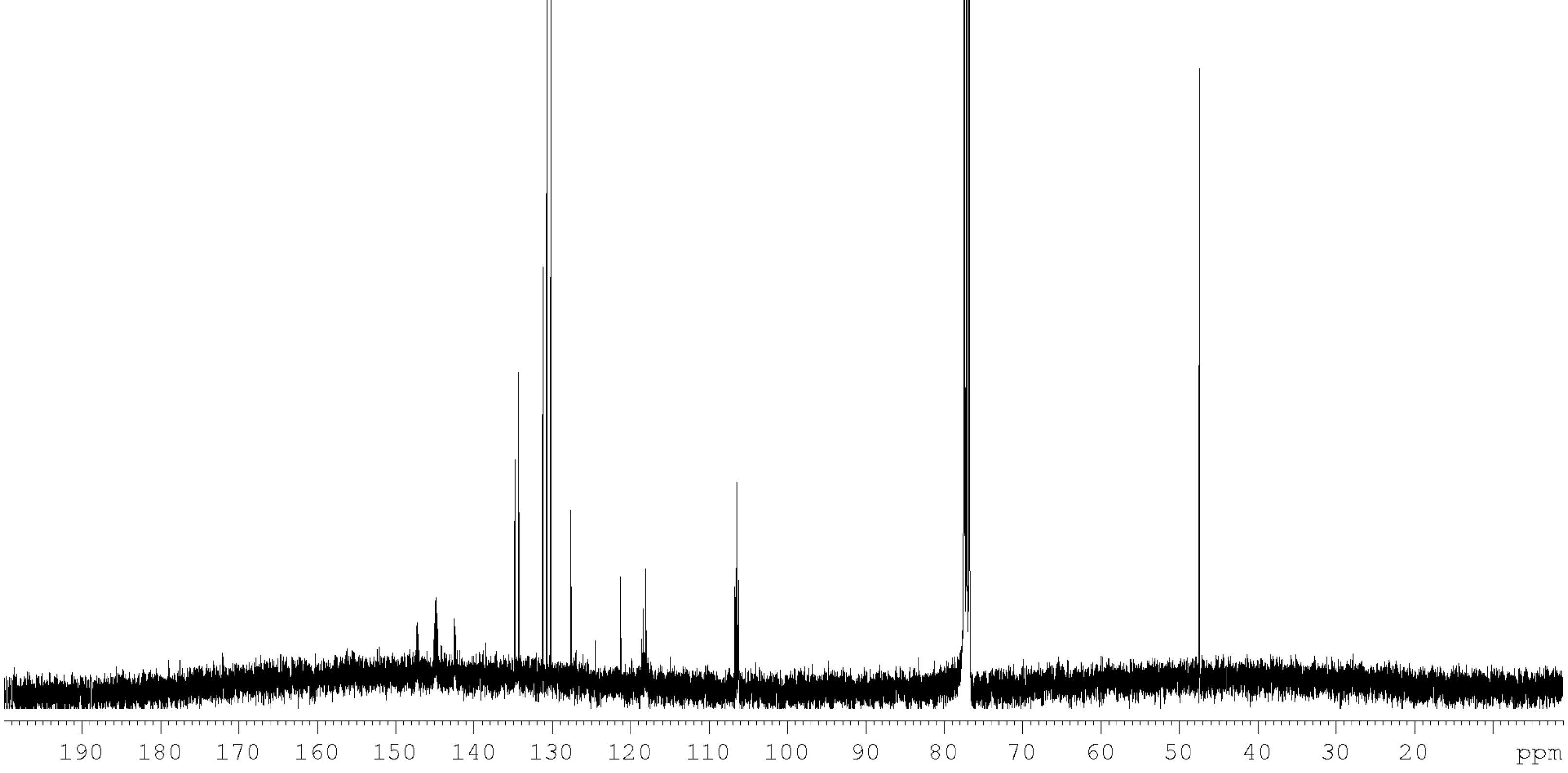

^{19}**F NMR** (376 MHZ, $CDCl_3$) **(3c)** N-((6-chloro-2',3',5',6'-tetrafluoro-[1,1'-biphenyl]-3-yl)methyl)-1,1,1-trifluoromethanesulfonamide

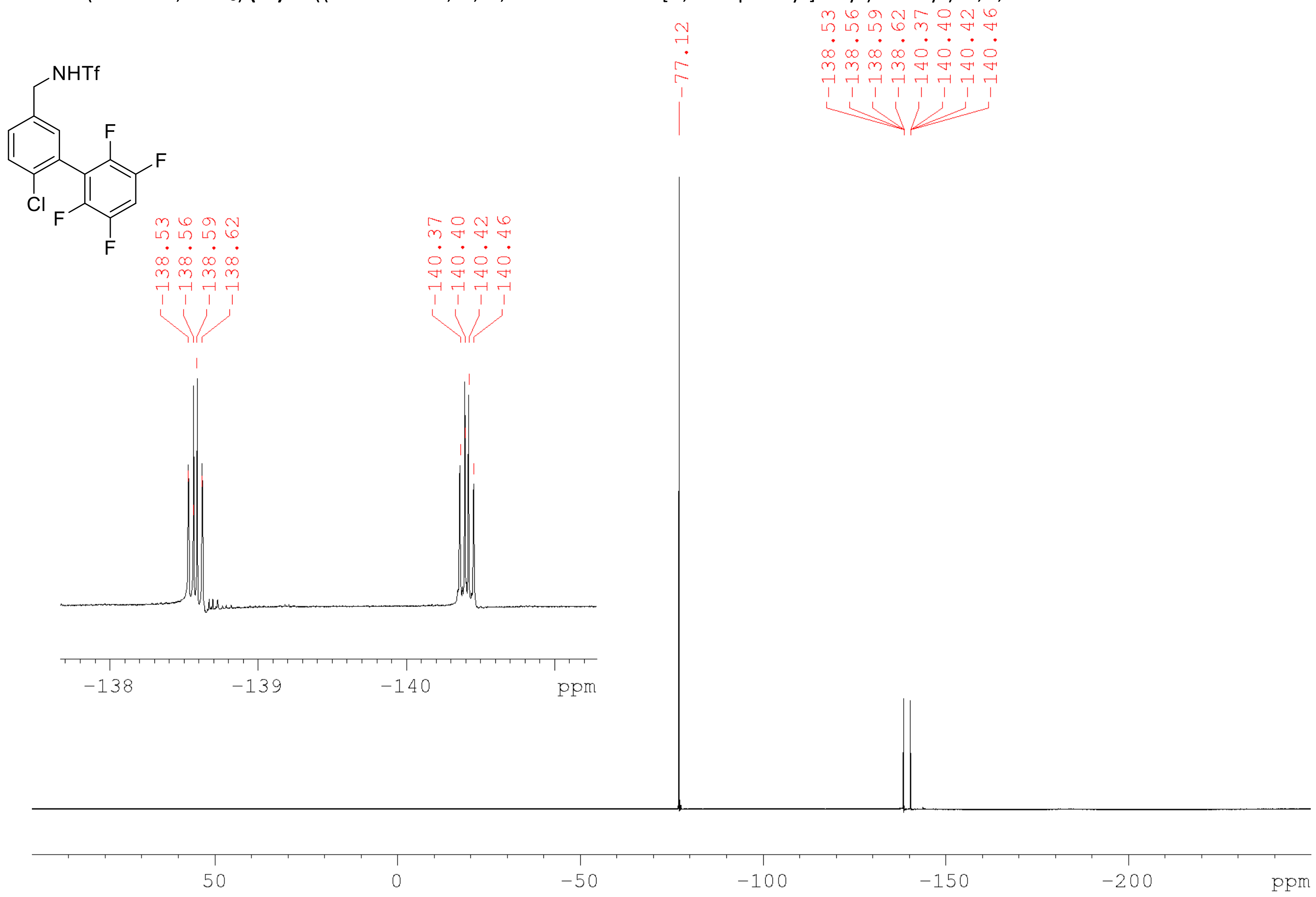

^{1}H NMR (400 MHZ, $CDCl_3$) **(3d)** N-((6-chloro-2',4',6'-trifluoro-[1,1'-biphenyl]-3-yl)methyl)-1,1,1-trifluoromethanesulfonamide

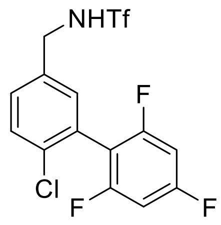

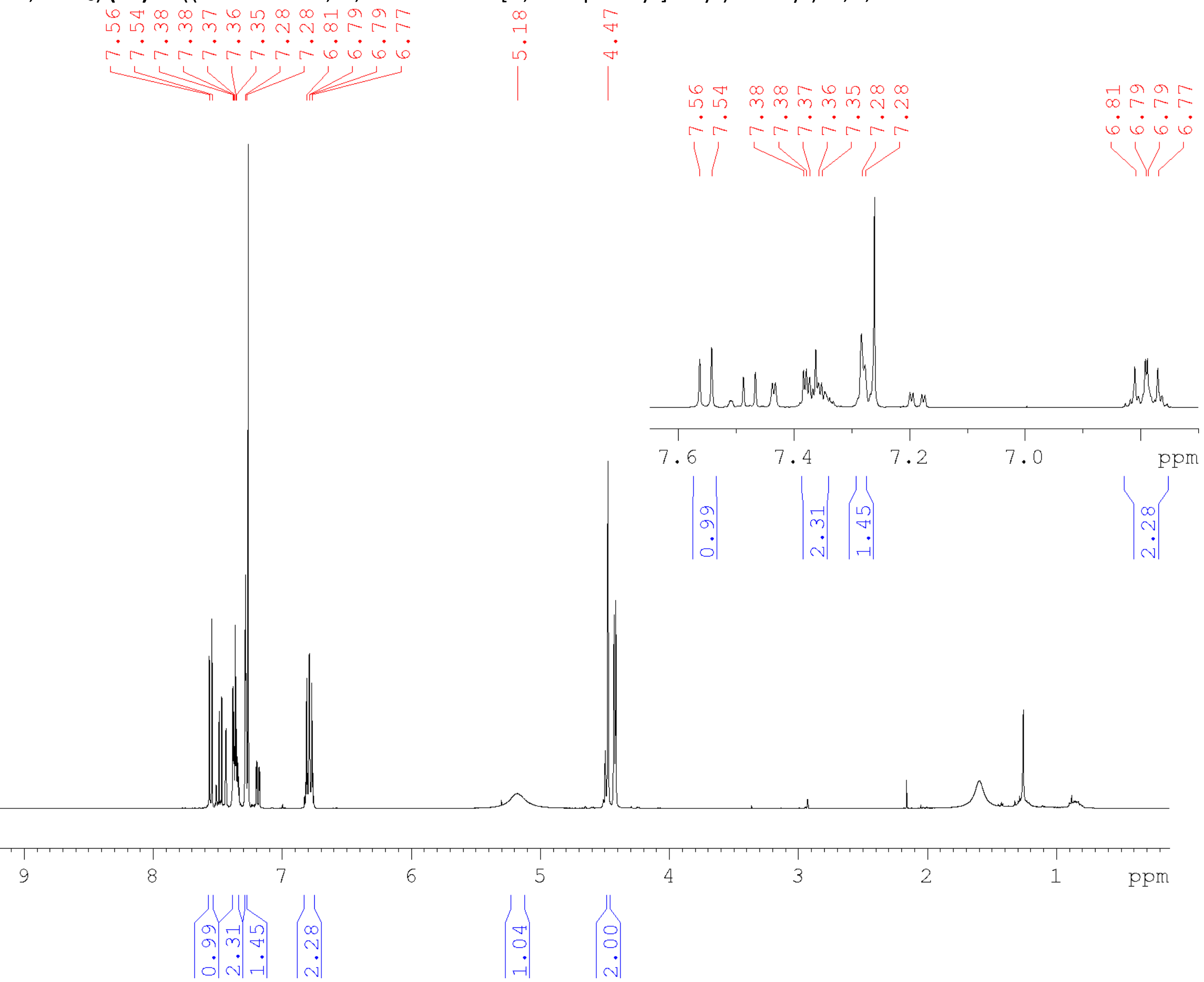

^{1}H NMR (400 MHZ, $CDCl_3$) **(3d)** N-((6-chloro-2',4',6'-trifluoro-[1,1'-biphenyl]-3-yl)methyl)-1,1,1-trifluoromethanesulfonamide(*further analysis – overlaid with meta product (**3a**) + starting material (**1a**))*

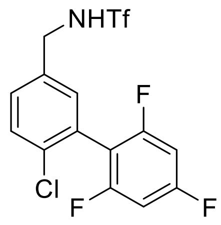

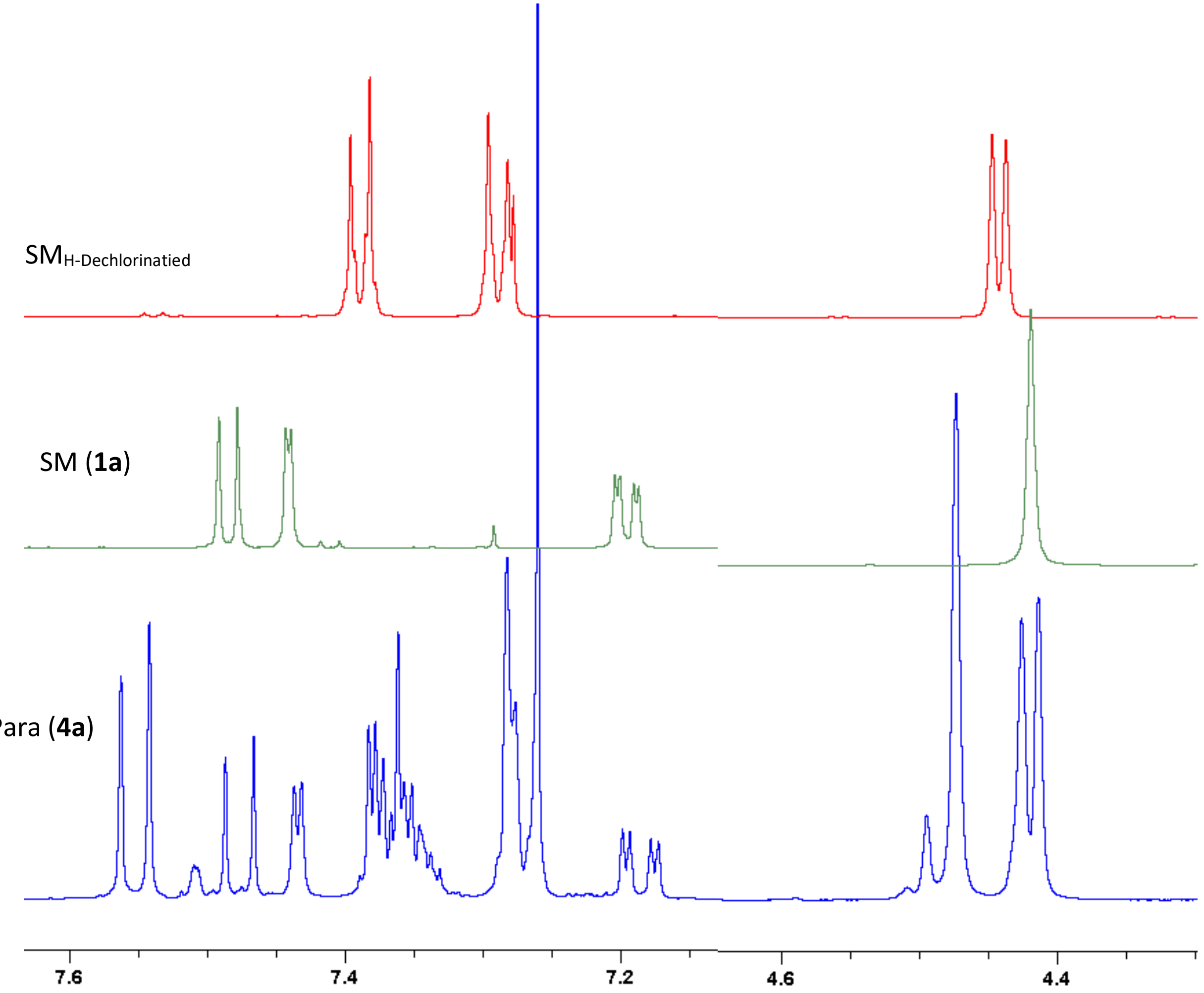

^{13}C NMR (126 MHZ, $CDCl_3$) **(3d)** N-((6-chloro-2',4',6'-trifluoro-[1,1'-biphenyl]-3-yl)methyl)-1,1,1-trifluoromethanesulfonamide

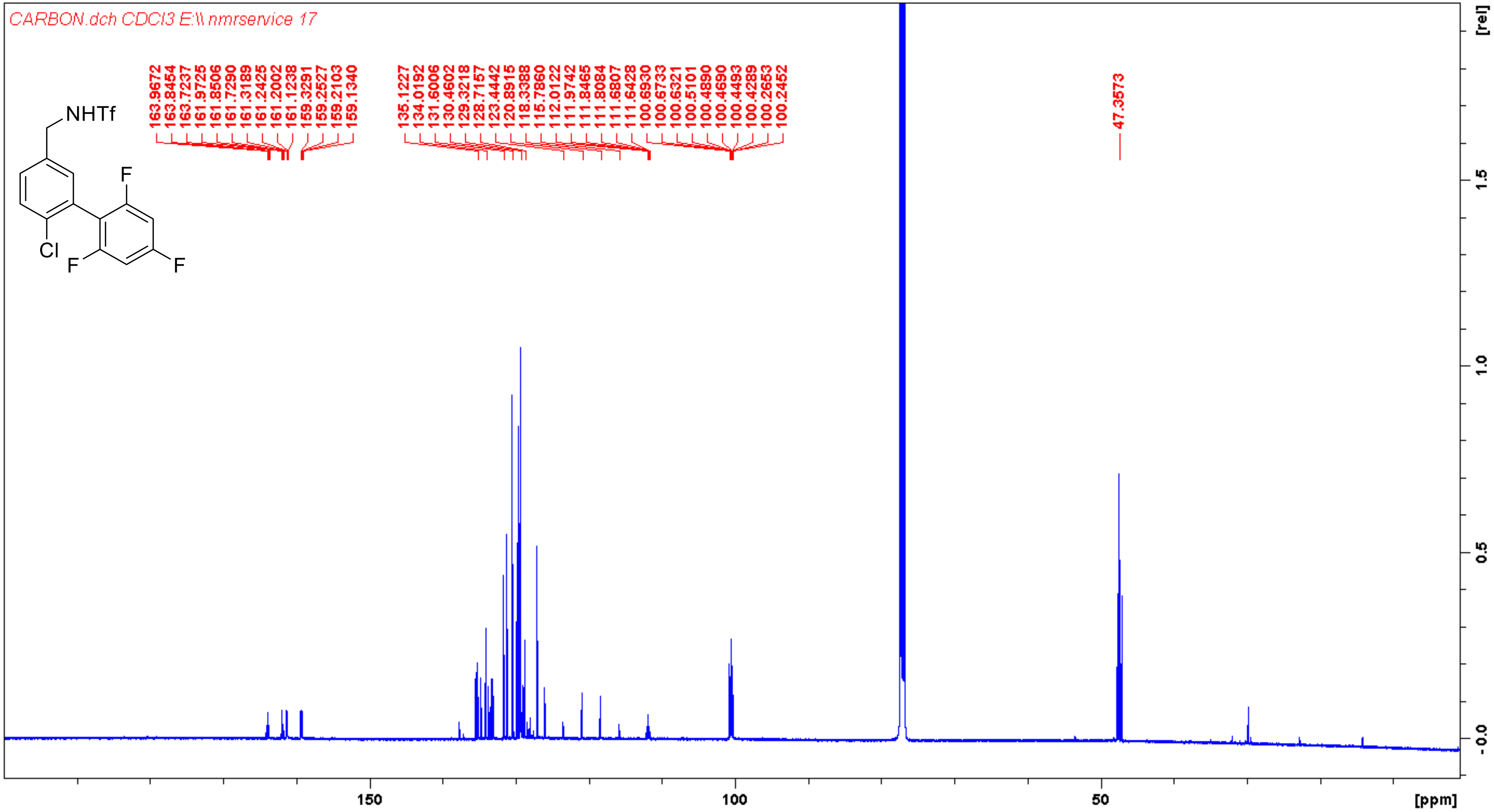

^{19}F NMR (376 MHZ, $CDCl_3$) **(3d)** N-((6-chloro-2',4',6'-trifluoro-[1,1'-biphenyl]-3-yl)methyl)-1,1,1-trifluoromethanesulfonamide

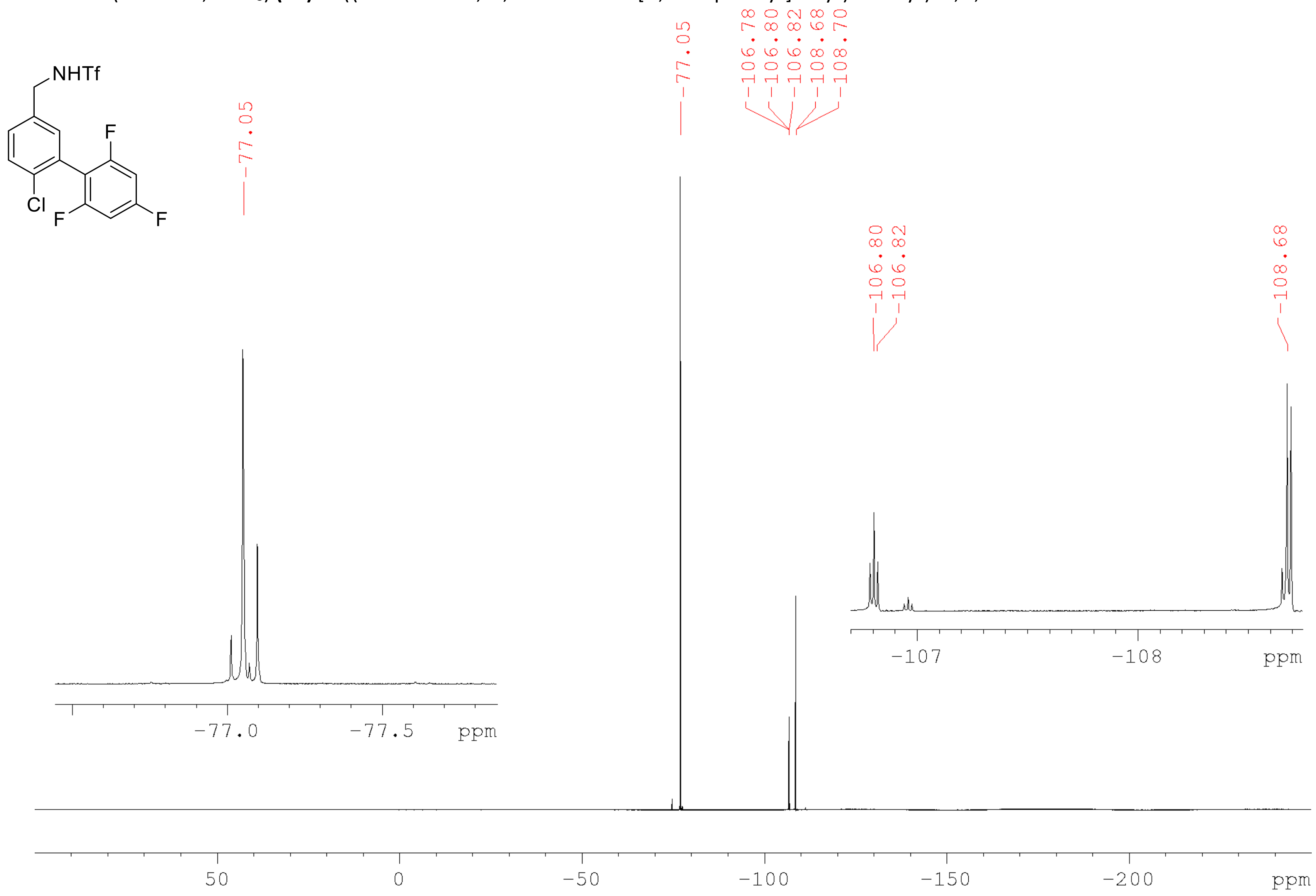

^{1}H NMR (400 MHZ, $CDCl_3$) **(3e)** N-((6-chloro-2',6'-difluoro-4'-methoxy-[1,1'-biphenyl]-3-yl)methyl)-1,1,1-trifluoromethanesulfonamide

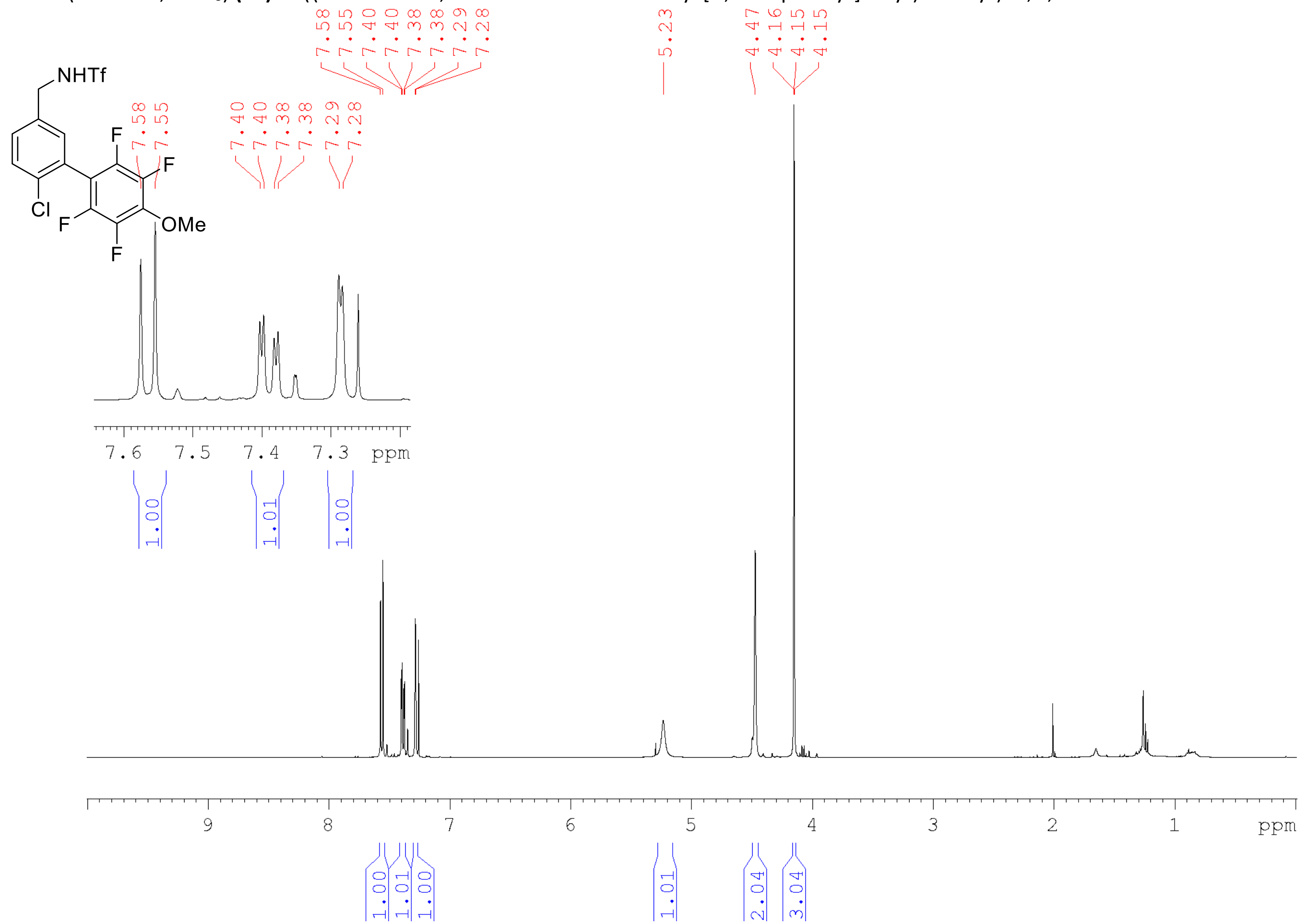

^{13}C NMR (101 MHZ, $CDCl_3$) **(3e)** N-((6-chloro-2',6'-difluoro-4'-methoxy-[1,1'-biphenyl]-3-yl)methyl)-1,1,1-trifluoromethanesulfonamide

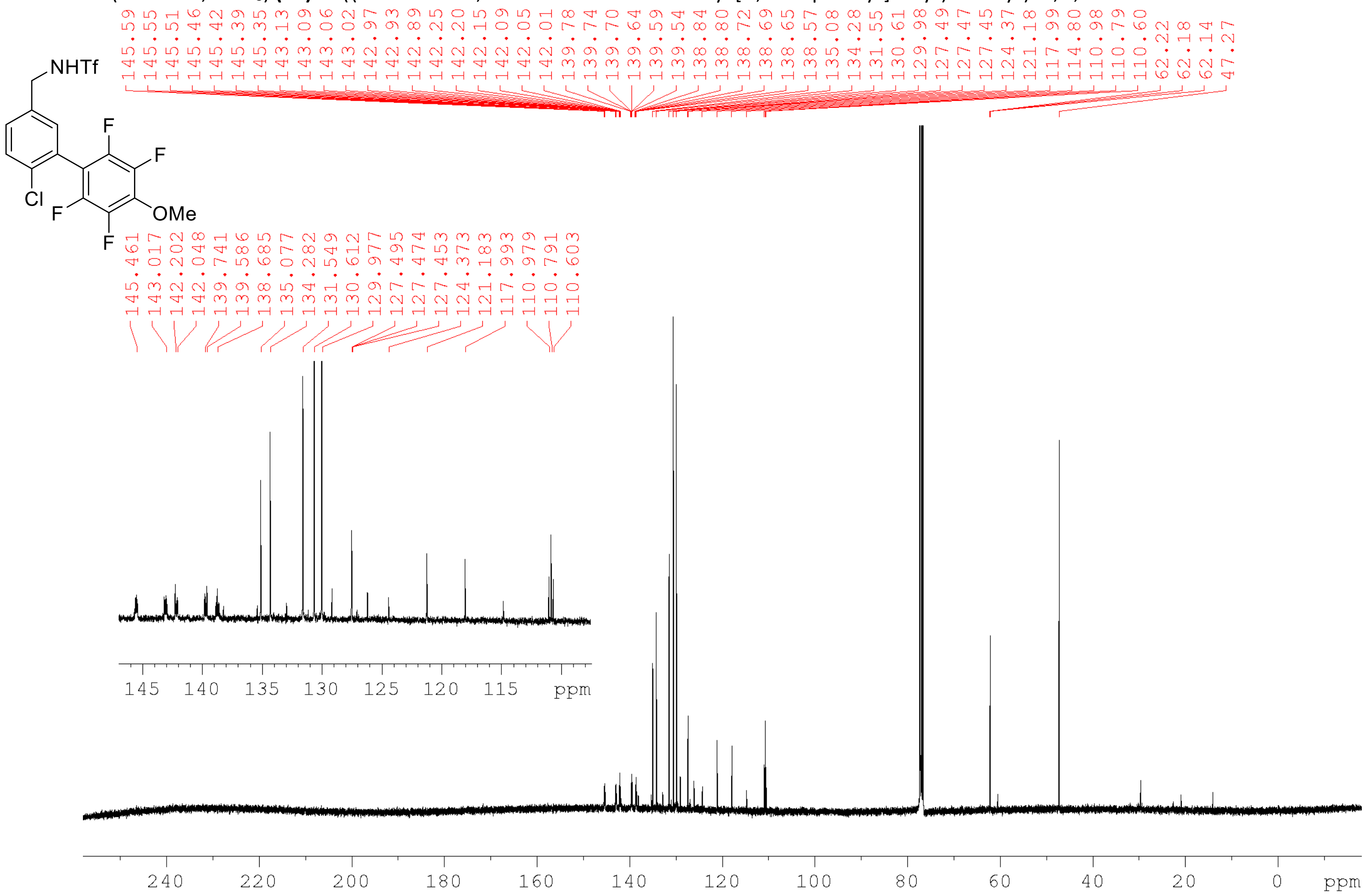

^{19}F NMR (101 MHZ, $CDCl_3$) **(3e)** N-((6-chloro-2',6'-difluoro-4'-methoxy-[1,1'-biphenyl]-3-yl)methyl)-1,1,1-trifluoromethanesulfonamide

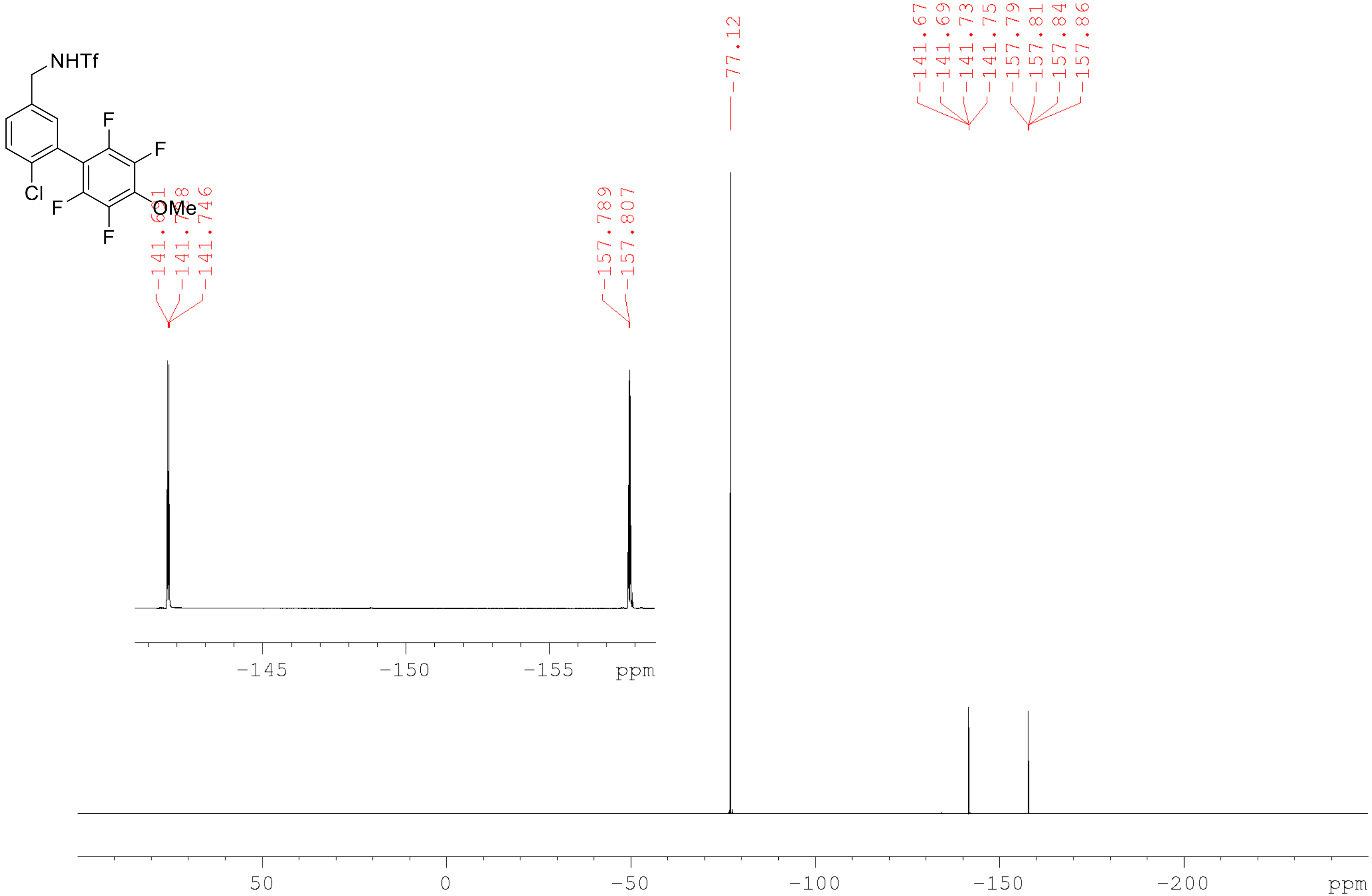

^{1}H NMR (400 MHZ, $CDCl_3$) **(3f)** N-((6-chloro-2',3',5',6'-tetrafluoro-4'-(trifluoromethyl)-[1,1'-biphenyl]-3-yl)methyl)-1,1,1-trifluoromethanesulfonamide

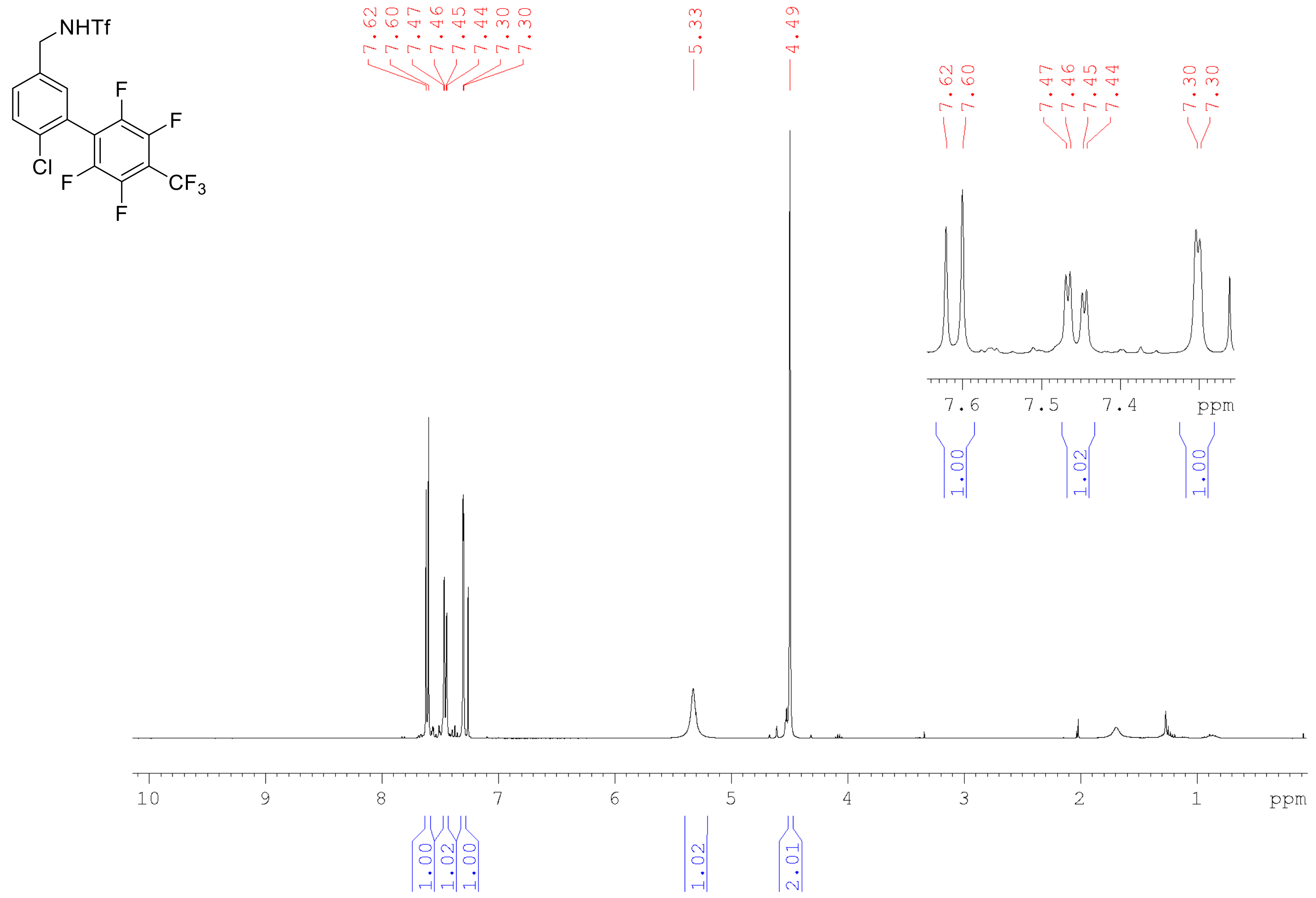

^{13}C NMR (101 MHZ, $CDCl_3$) **(3f)** N-((6-chloro-2',3',5',6'-tetrafluoro-4'-(trifluoromethyl)-[1,1'-biphenyl]-3-yl)methyl)-1,1,1-trifluoromethanesulfonamide

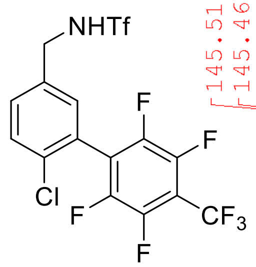

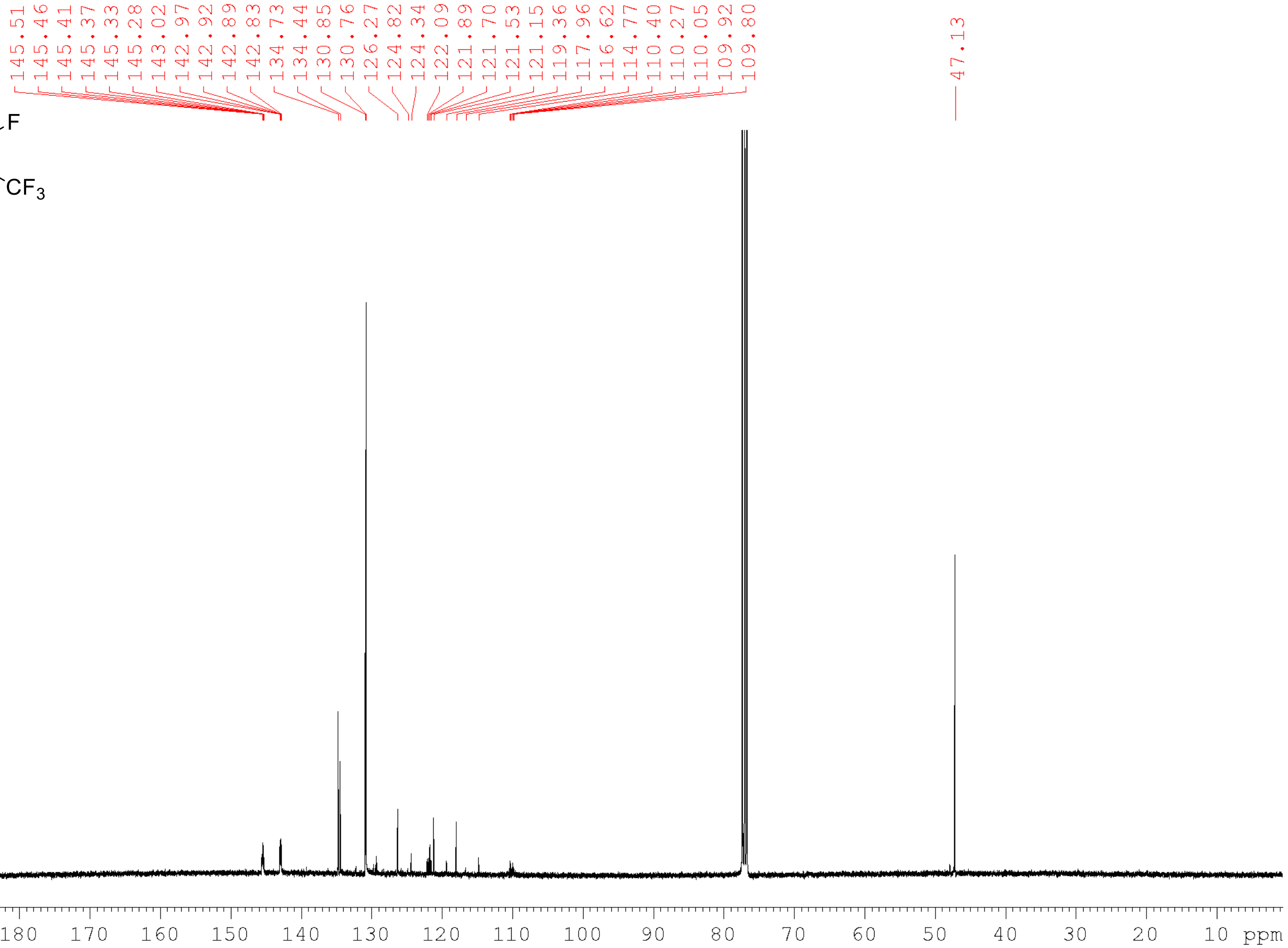

^{19}F NMR (376 MHZ, $CDCl_3$) **(3f)** N-((6-chloro-2',3',5',6'-tetrafluoro-4'-(trifluoromethyl)-[1,1'-biphenyl]-3-yl)methyl)-1,1,1-trifluoromethanesulfonamide

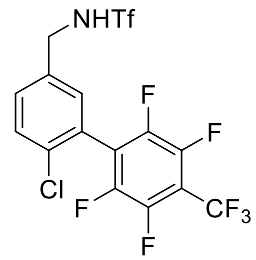

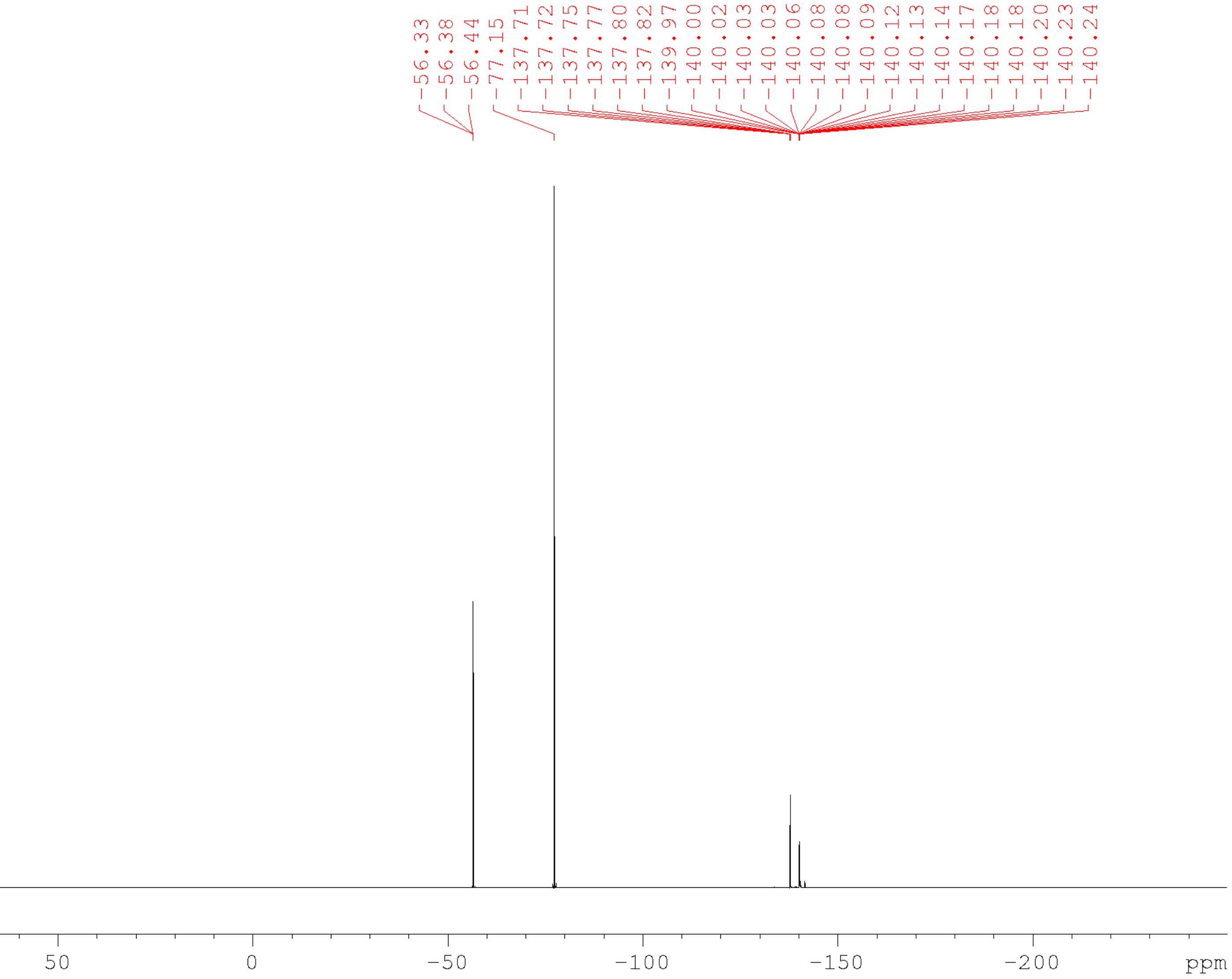

^{1}H NMR (400 MHZ, CDCl$_3$) **(3g)** N-((6-chloro-2',3',5',6'-tetrafluoro-4'-((triisopropylsilyl)ethynyl)-[1,1'-biphenyl]-3-yl)methyl)-1,1,1-trifluoromethanesulfonamide

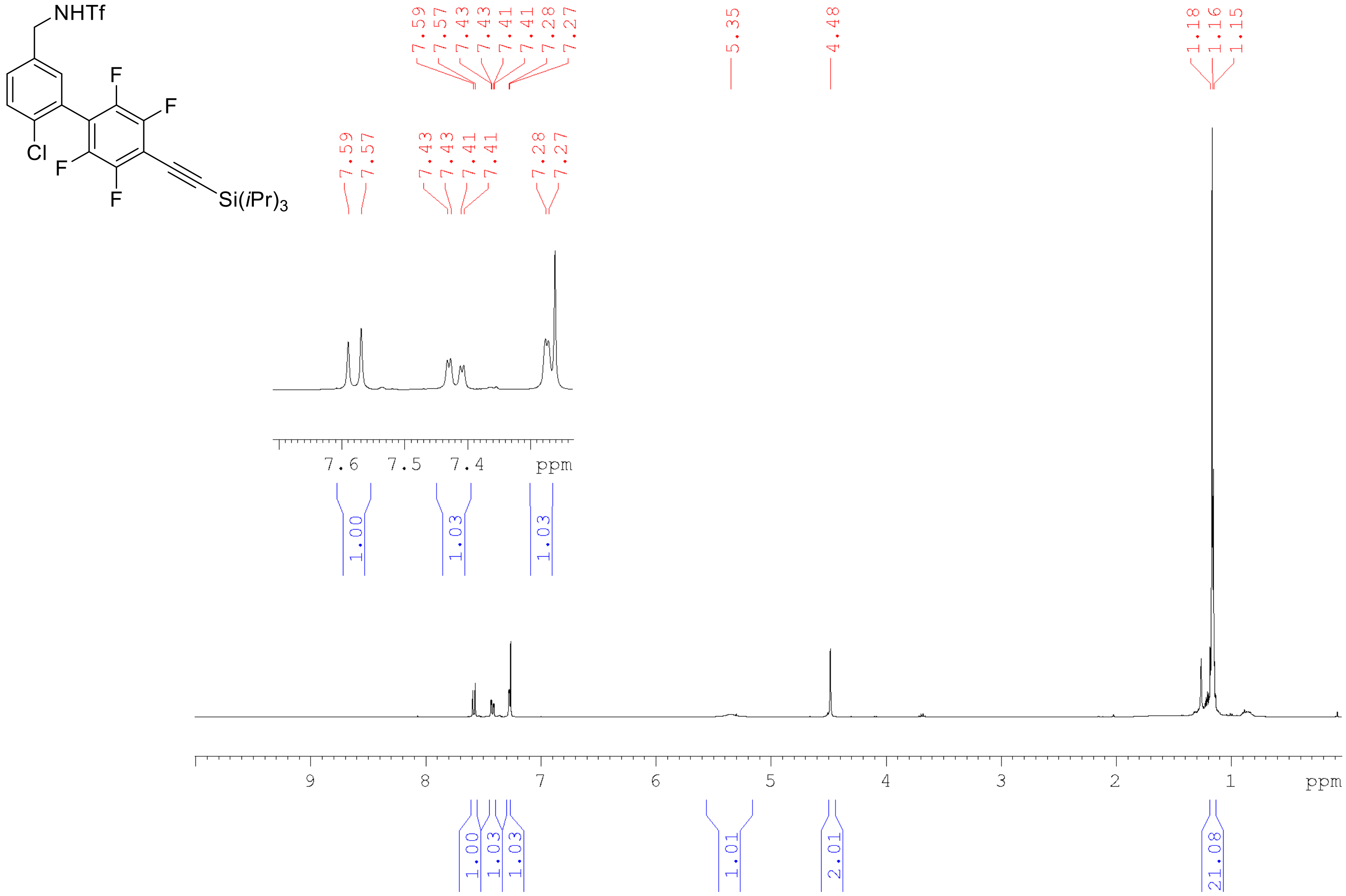

^{13}C NMR (101 MHZ, $CDCl_3$) **(3g)** N-((6-chloro-2',3',5',6'-tetrafluoro-4'-((triisopropylsilyl)ethynyl)-[1,1'-biphenyl]-3-yl)methyl)-1,1,1-trifluoromethanesulfonamide

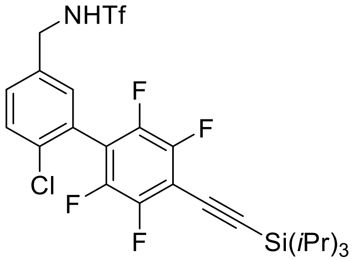

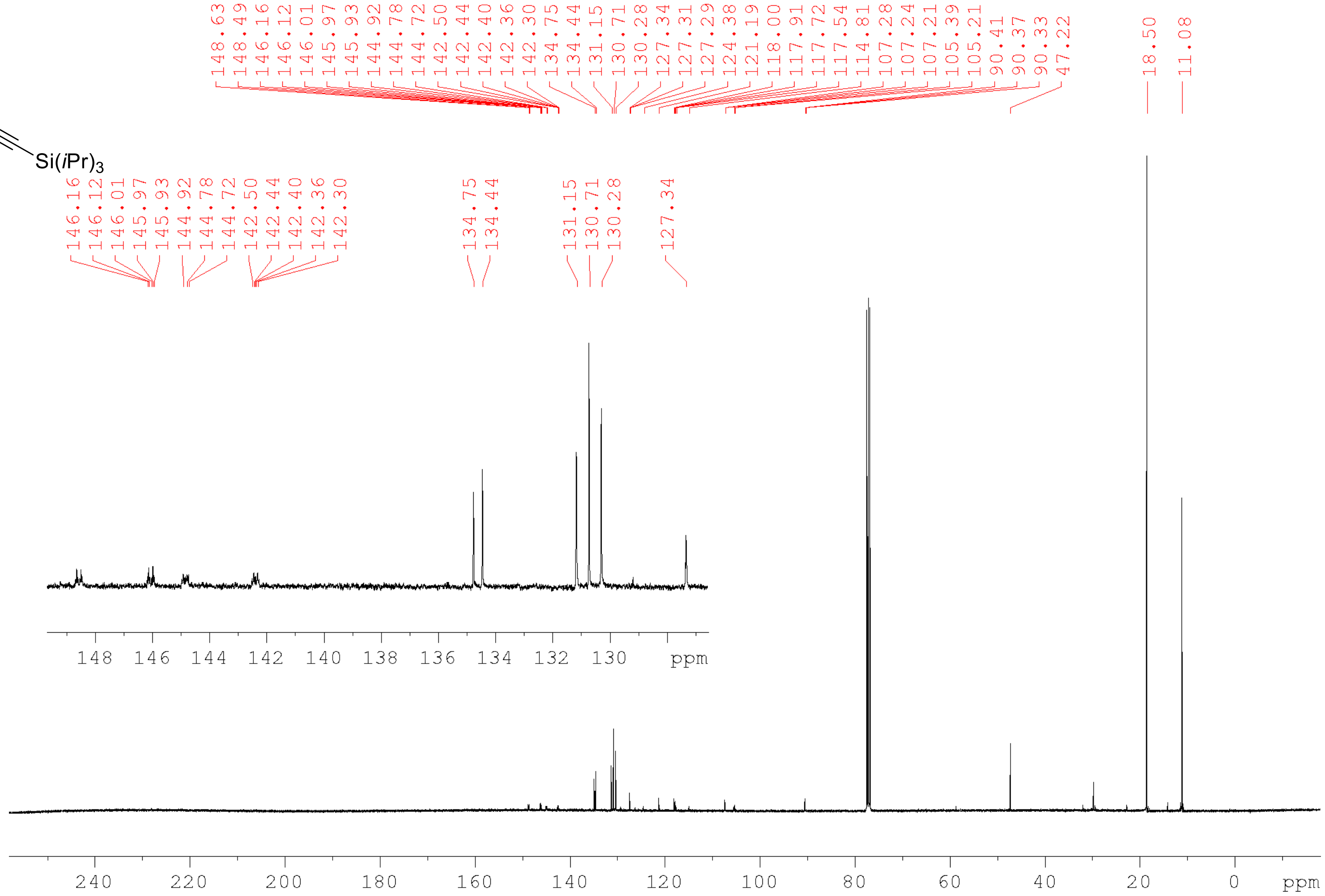

^{19}F NMR (376 MHZ, $CDCl_3$) **(3g)** N-((6-chloro-2',3',5',6'-tetrafluoro-4'-((triisopropylsilyl)ethynyl)-[1,1'-biphenyl]-3-yl)methyl)-1,1,1-trifluoromethanesulfonamide

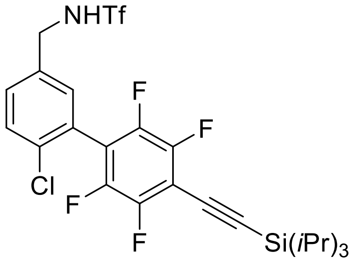

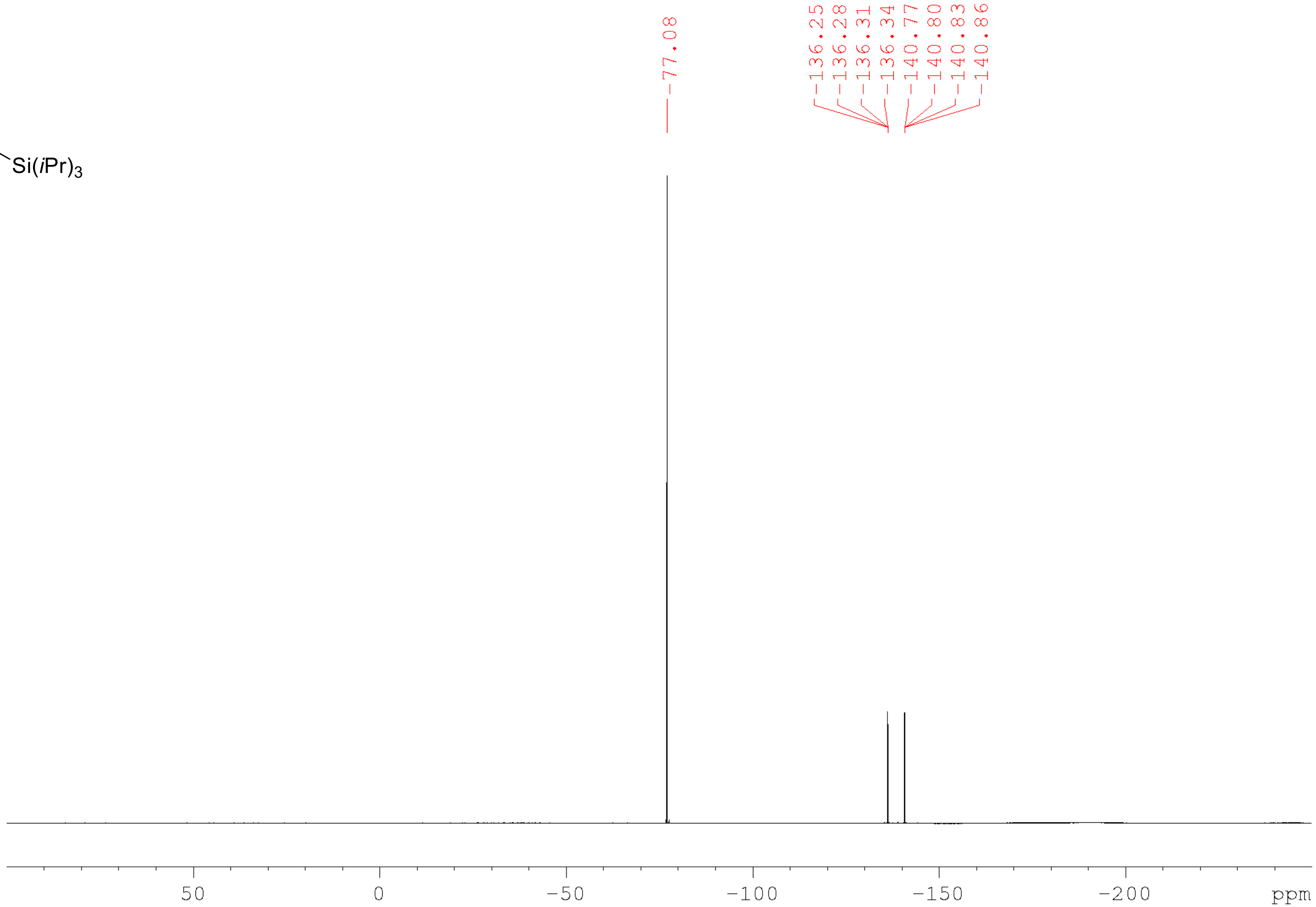

^{1}H NMR (400 MHZ, $CDCl_3$) **(3h)** methyl 2'-chloro-2,3,5,6-tetrafluoro-5'-(((trifluoromethyl)sulfonamido)methyl)-[1,1'-biphenyl]-4-carboxylate

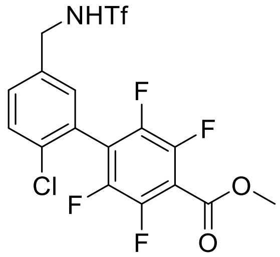

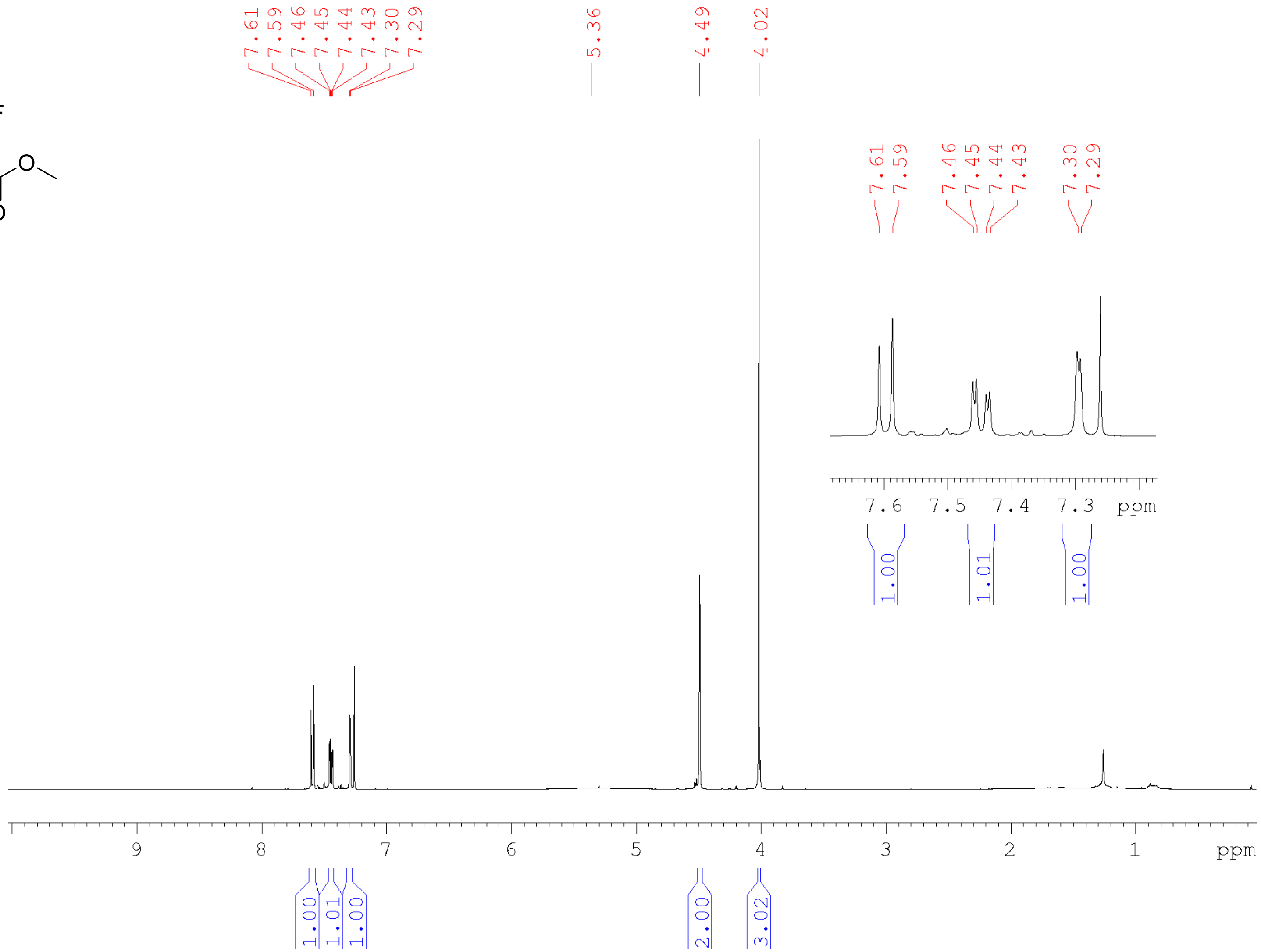

13C NMR (101 MHZ, $CDCl_3$) **(3h)** methyl 2'-chloro-2,3,5,6-tetrafluoro-5'-(((trifluoromethyl)sulfonamido)methyl)-[1,1'-biphenyl]-4-carboxylate

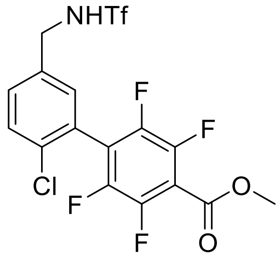

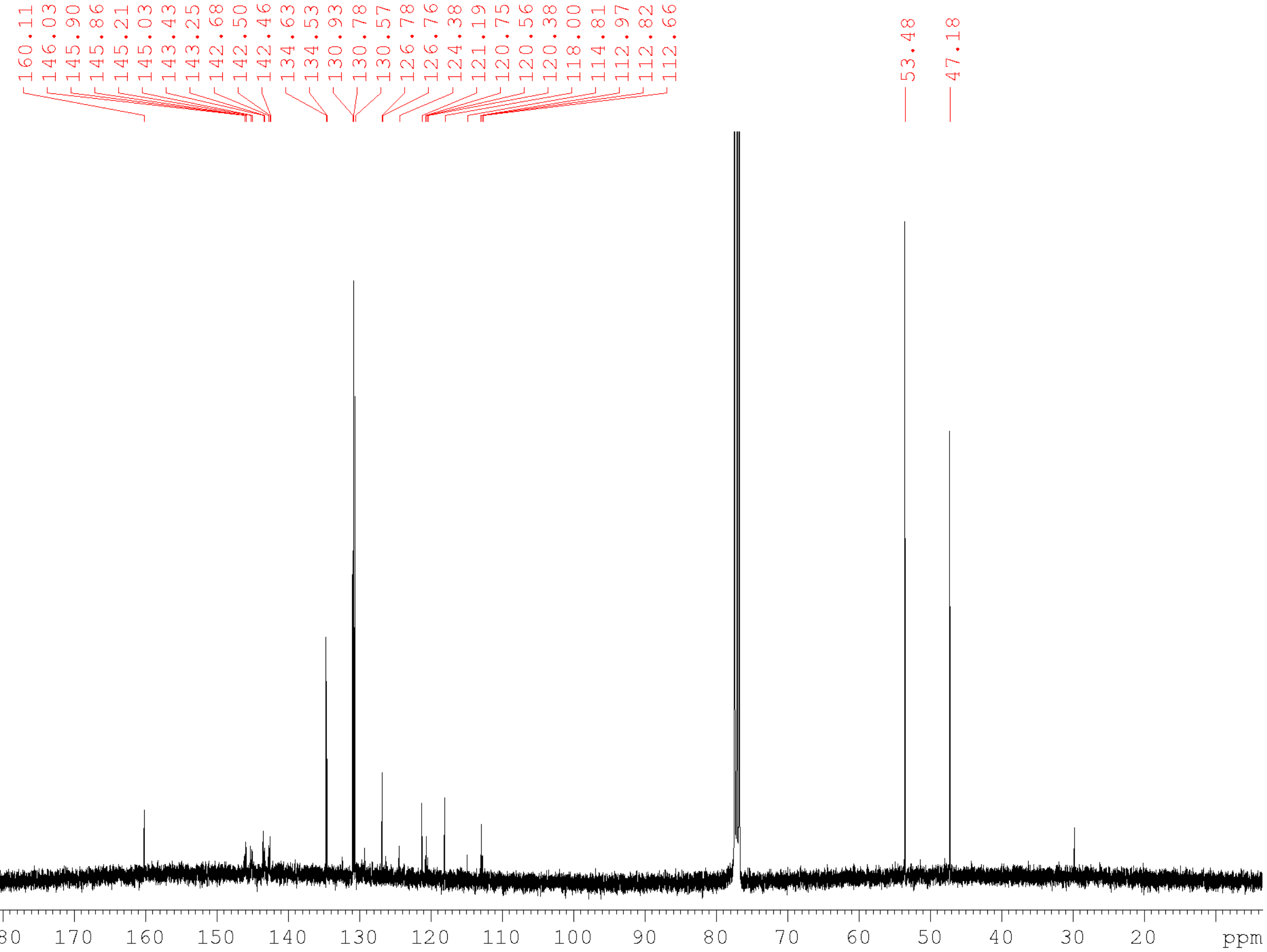

^{19}F NMR (376 MHZ, $CDCl_3$) **(3h)** methyl 2'-chloro-2,3,5,6-tetrafluoro-5'-(((trifluoromethyl)sulfonamido)methyl)-[1,1'-biphenyl]-4-carboxylate

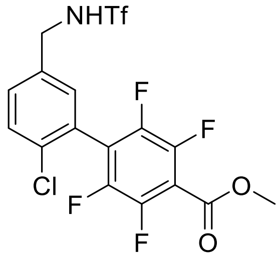

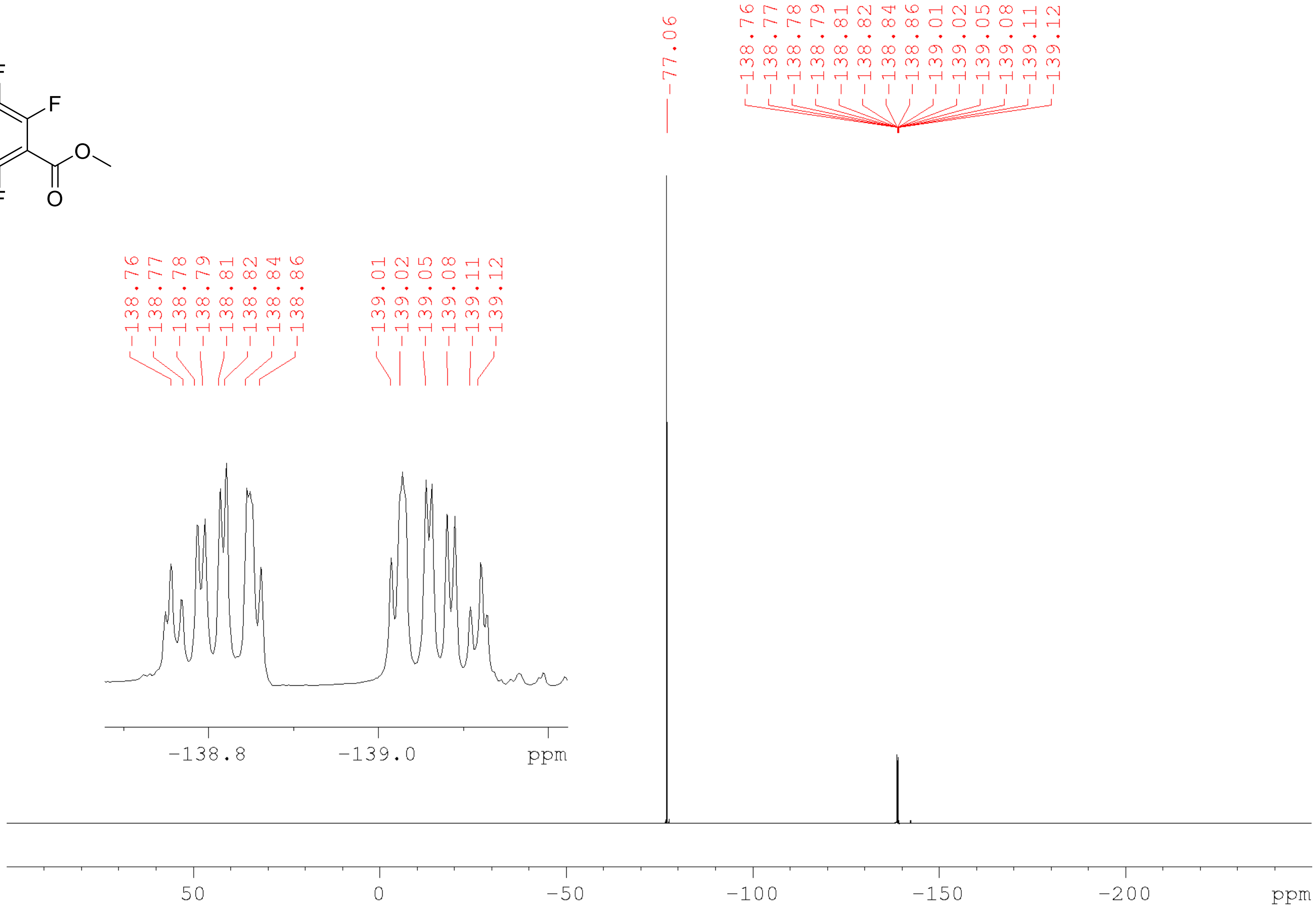

^{1}H NMR (400 MHZ, MeOD) **(3i)** N-(2'-chloro-2,3,5,6-tetrafluoro-5'-(((trifluoromethyl)sulfonamido)methyl)-[1,1'-biphenyl]-4-yl)acetamide

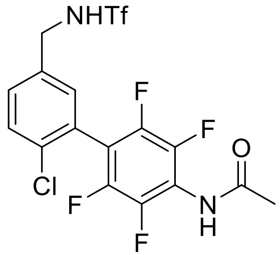

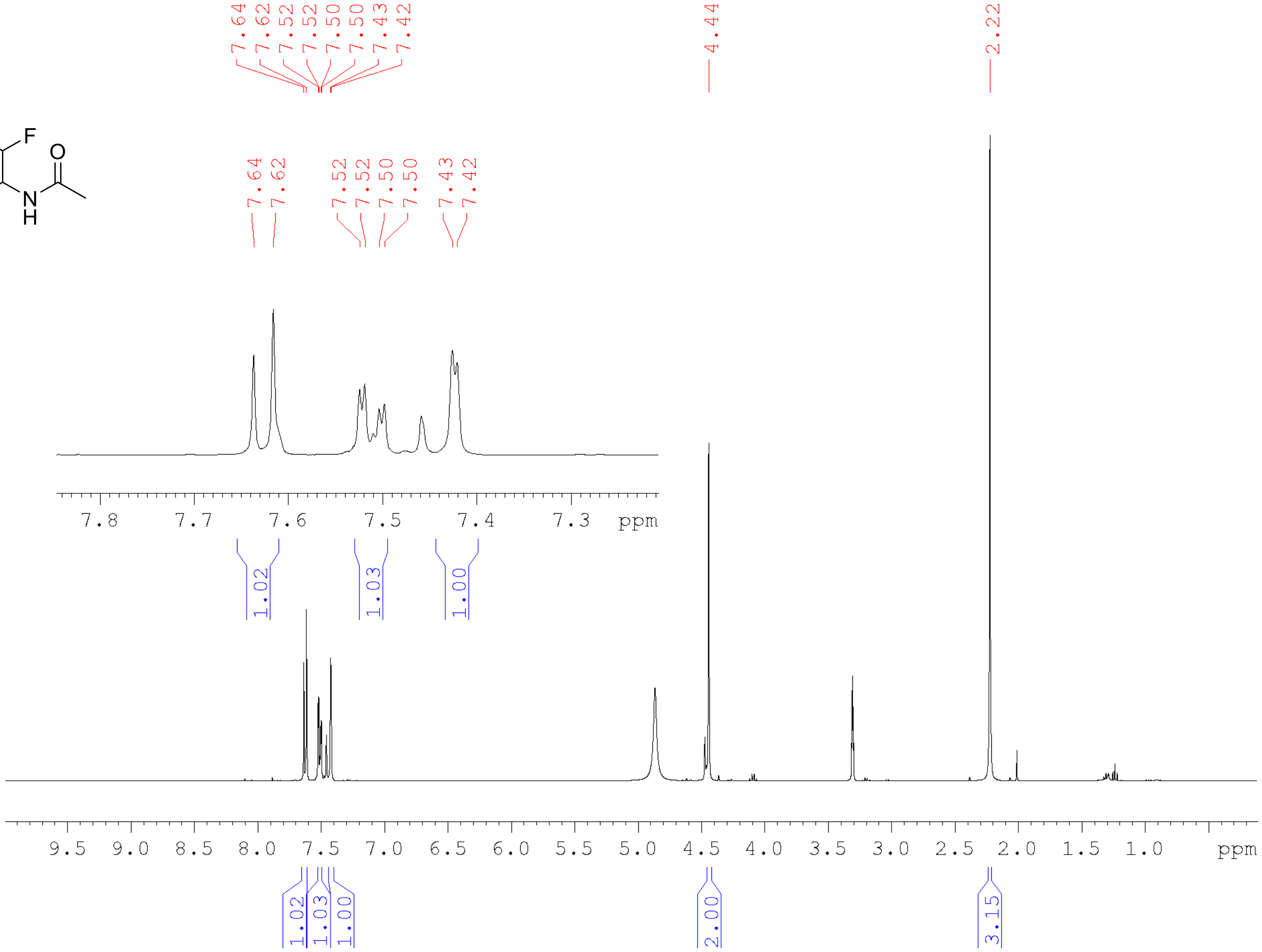

^{13}C NMR (101 MHZ, MeOD) **(3i)** N-(2'-chloro-2,3,5,6-tetrafluoro-5'-(((trifluoromethyl)sulfonamido)methyl)-[1,1'-biphenyl]-4-yl)acetamide

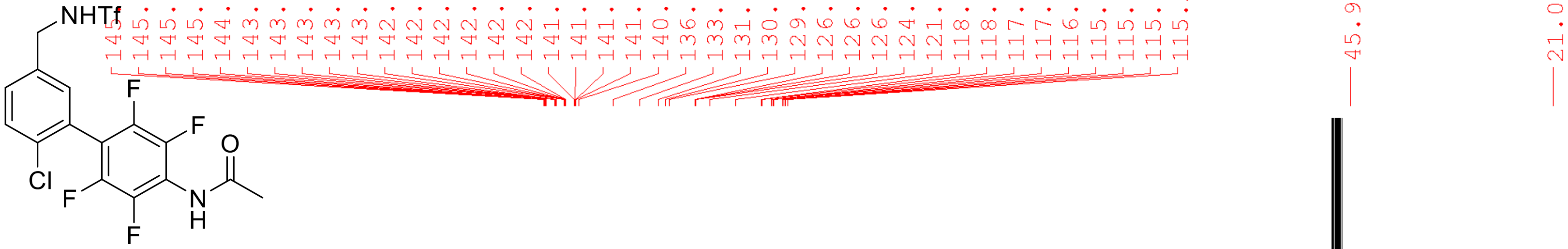

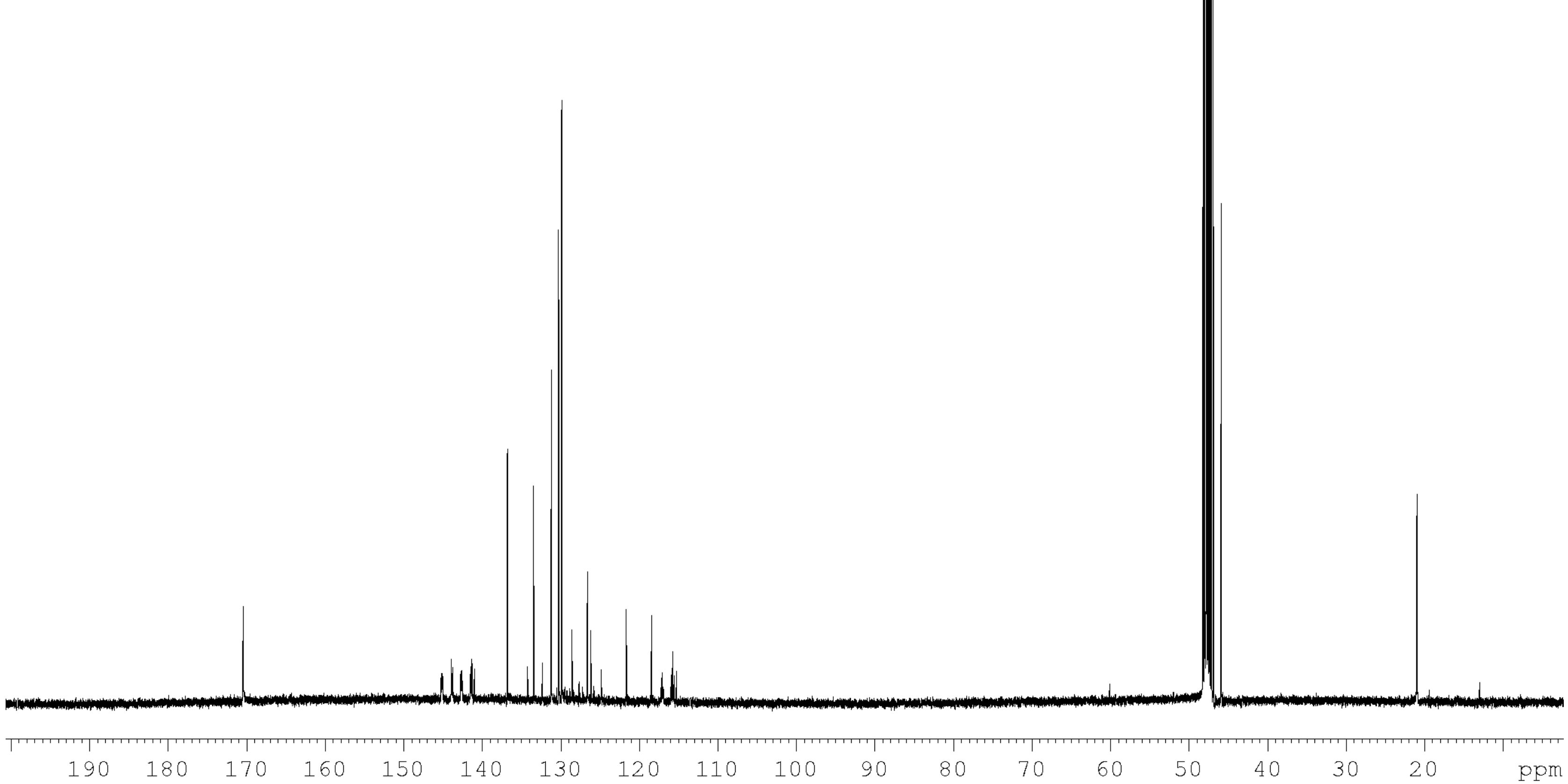

^{13}C NMR (101 MHZ, MeOD) **(3i)** N-(2'-chloro-2,3,5,6-tetrafluoro-5'-(((trifluoromethyl)sulfonamido)methyl)-[1,1'-biphenyl]-4-yl)acetamide

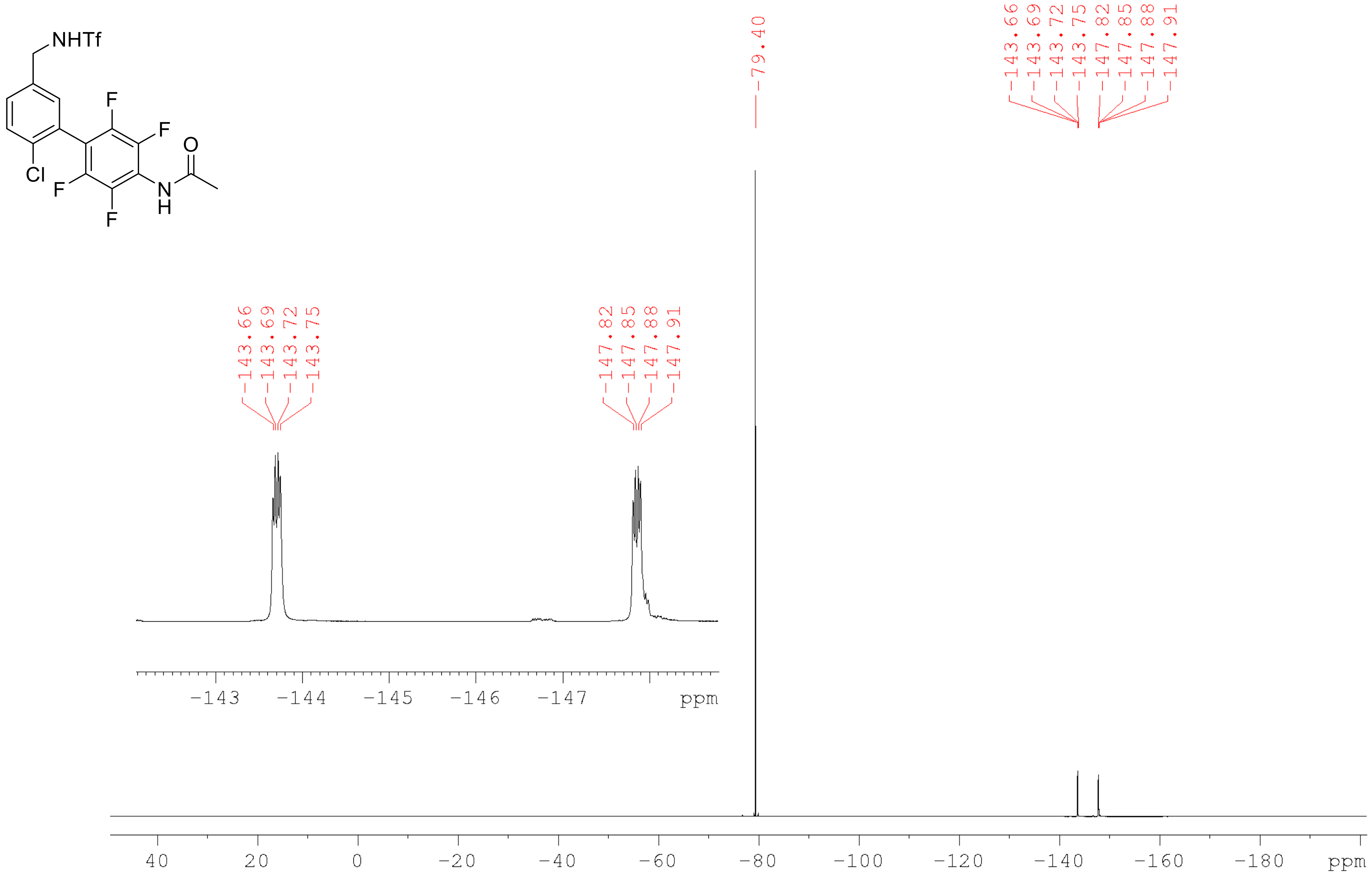

[1]**H NMR** (400 MHZ, $CDCl_3$) **(3j)** N-((6-chloro-2',2'',3',3'',4'',5',5'',6',6''-nonafluoro-[1,1':4',1''-terphenyl]-3-yl)methyl)-1,1,1-trifluoromethanesulfonamide

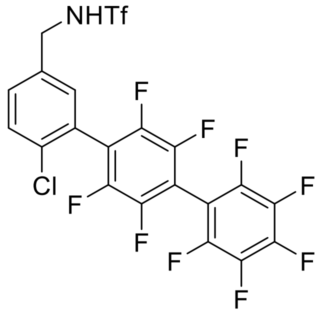

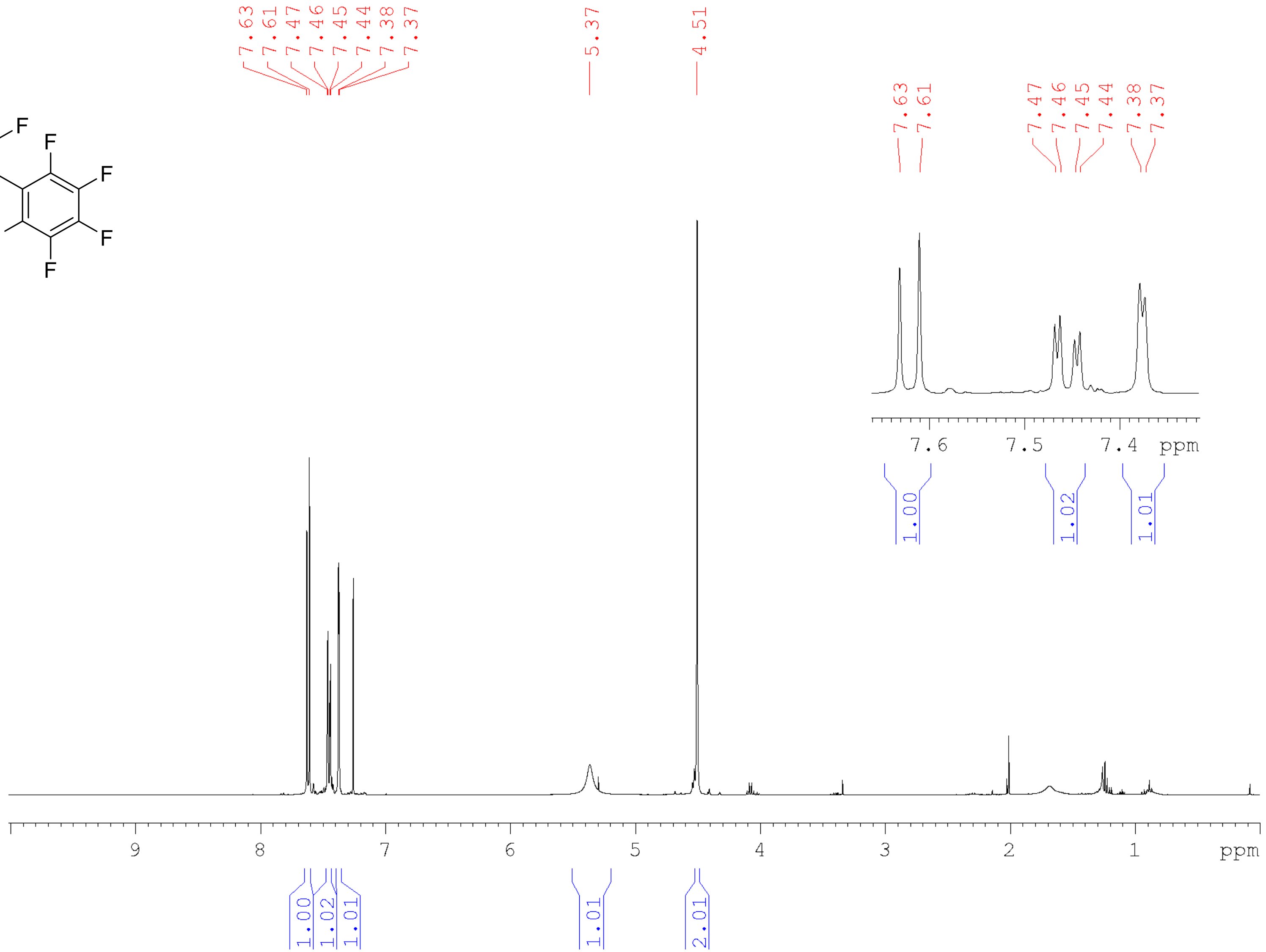

^{13}C NMR (101 MHZ, $CDCl_3$) **(3j)** N-((6-chloro-2',2'',3',3'',4'',5',5'',6',6''-nonafluoro-[1,1':4',1''-terphenyl]-3-yl)methyl)-1,1,1-trifluoromethanesulfonamide

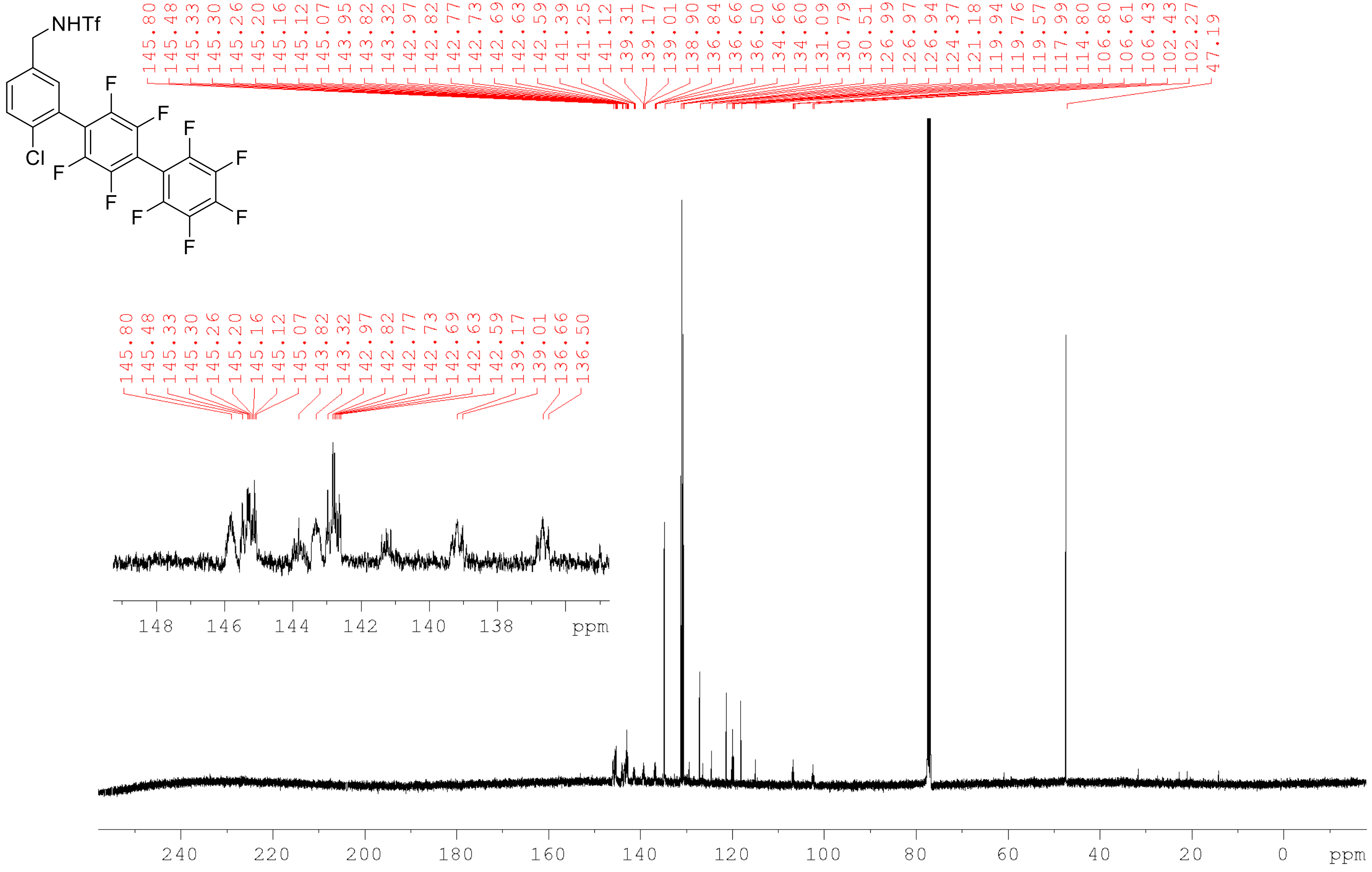

^{19}F NMR (376 MHZ, $CDCl_3$) **(3j)** N-((6-chloro-2',2'',3',3'',4'',5',5'',6',6''-nonafluoro-[1,1':4',1''-terphenyl]-3-yl)methyl)-1,1,1-trifluoromethanesulfonamide

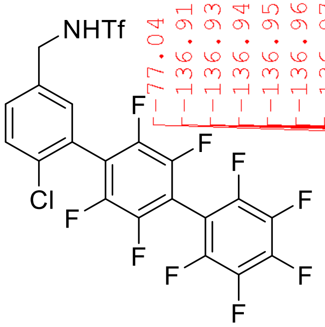

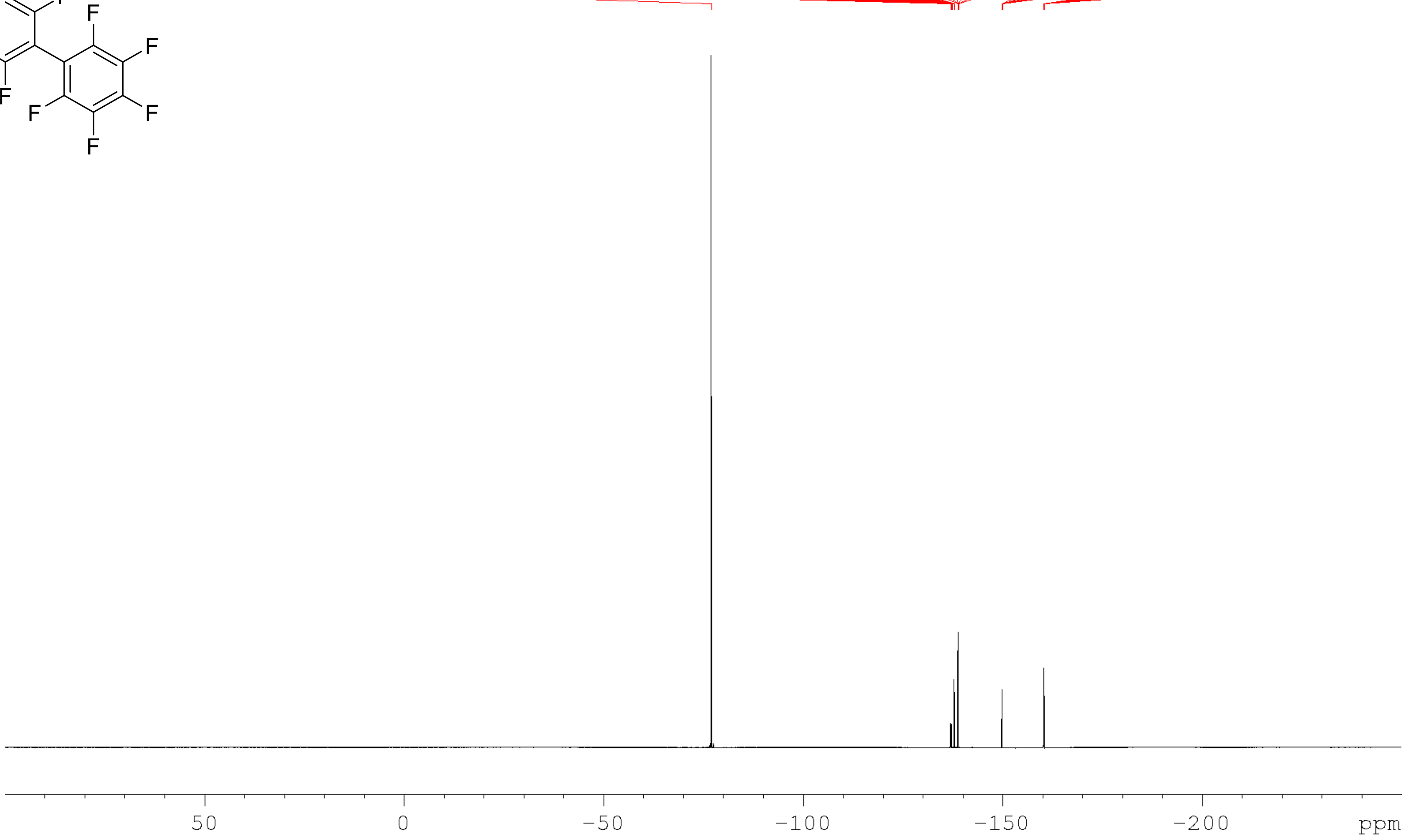

Fluorinated Pyridine scope

^{1}H NMR (400 MHZ, $CDCl_3$) **(5a)** N-(4-chloro-3-(perfluoropyridin-4-yl)benzyl)-1,1,1-trifluoromethanesulfonamide

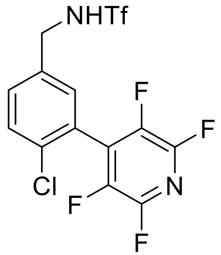

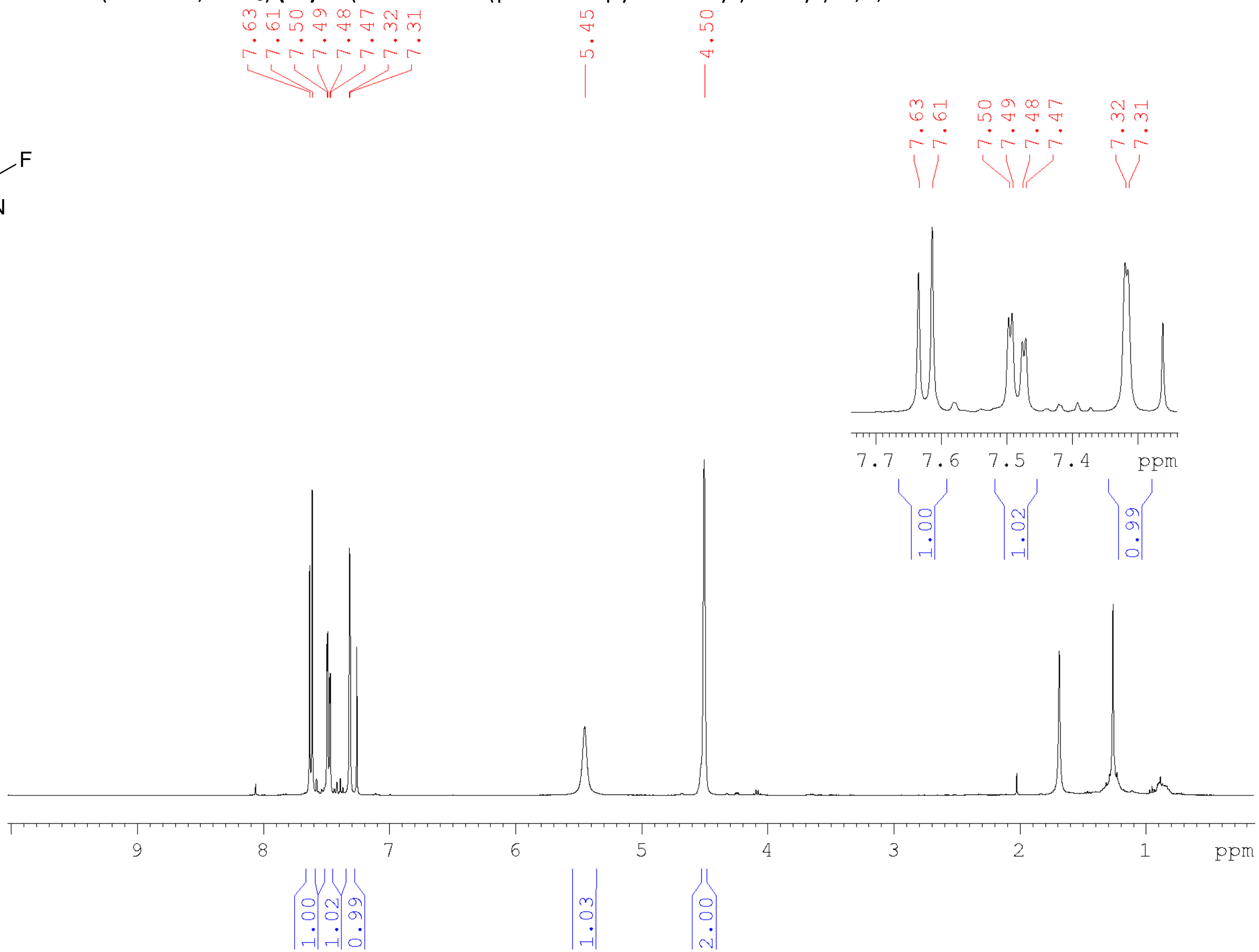

^{13}C NMR (101 MHZ, $CDCl_3$) **(5a)** N-(4-chloro-3-(perfluoropyridin-4-yl)benzyl)-1,1,1-trifluoromethanesulfonamide

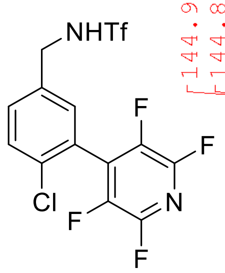

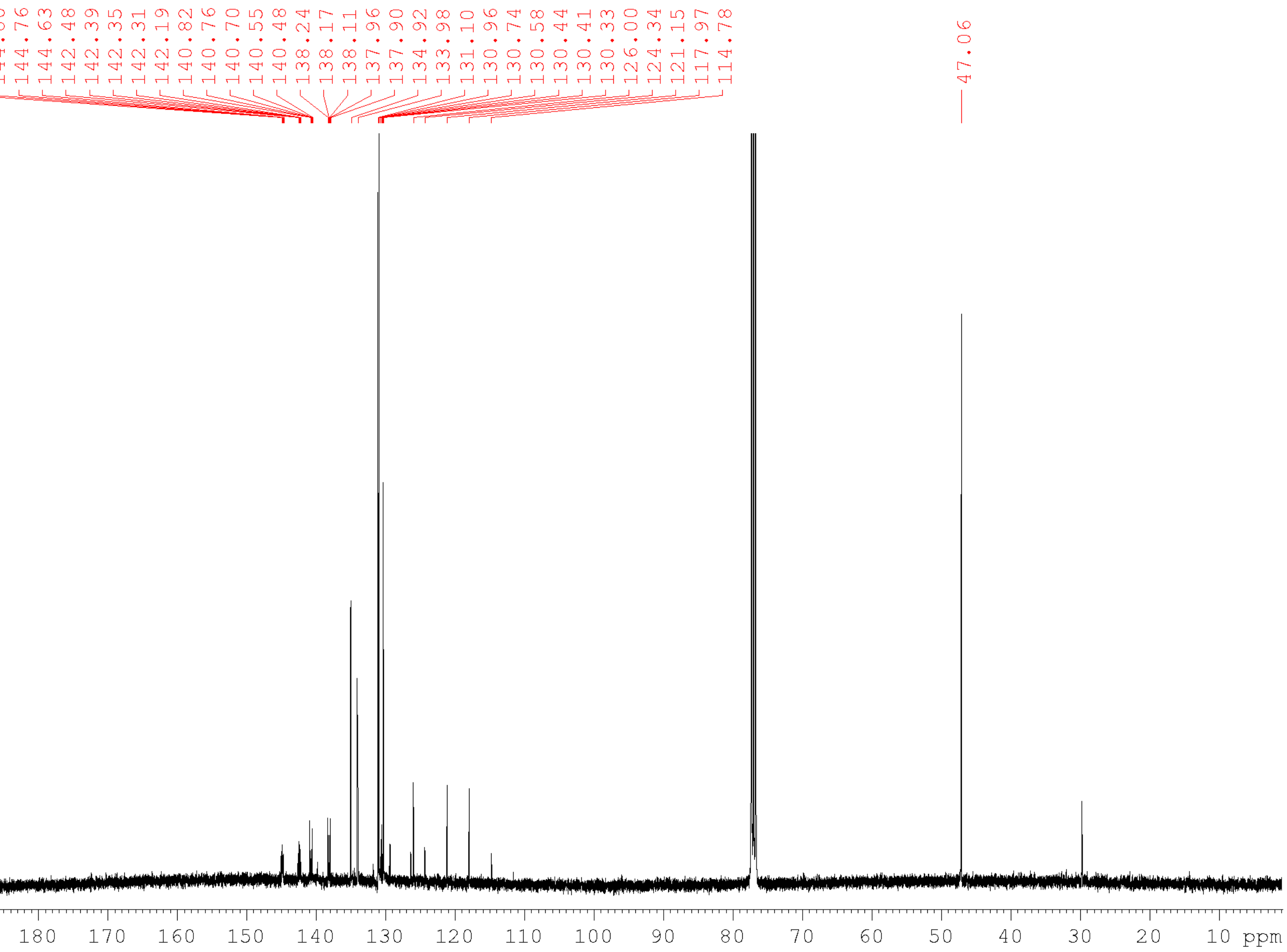

^{19}F NMR (376 MHZ, $CDCl_3$) **(5a)** N-(4-chloro-3-(perfluoropyridin-4-yl)benzyl)-1,1,1-trifluoromethanesulfonamide

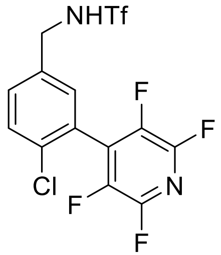

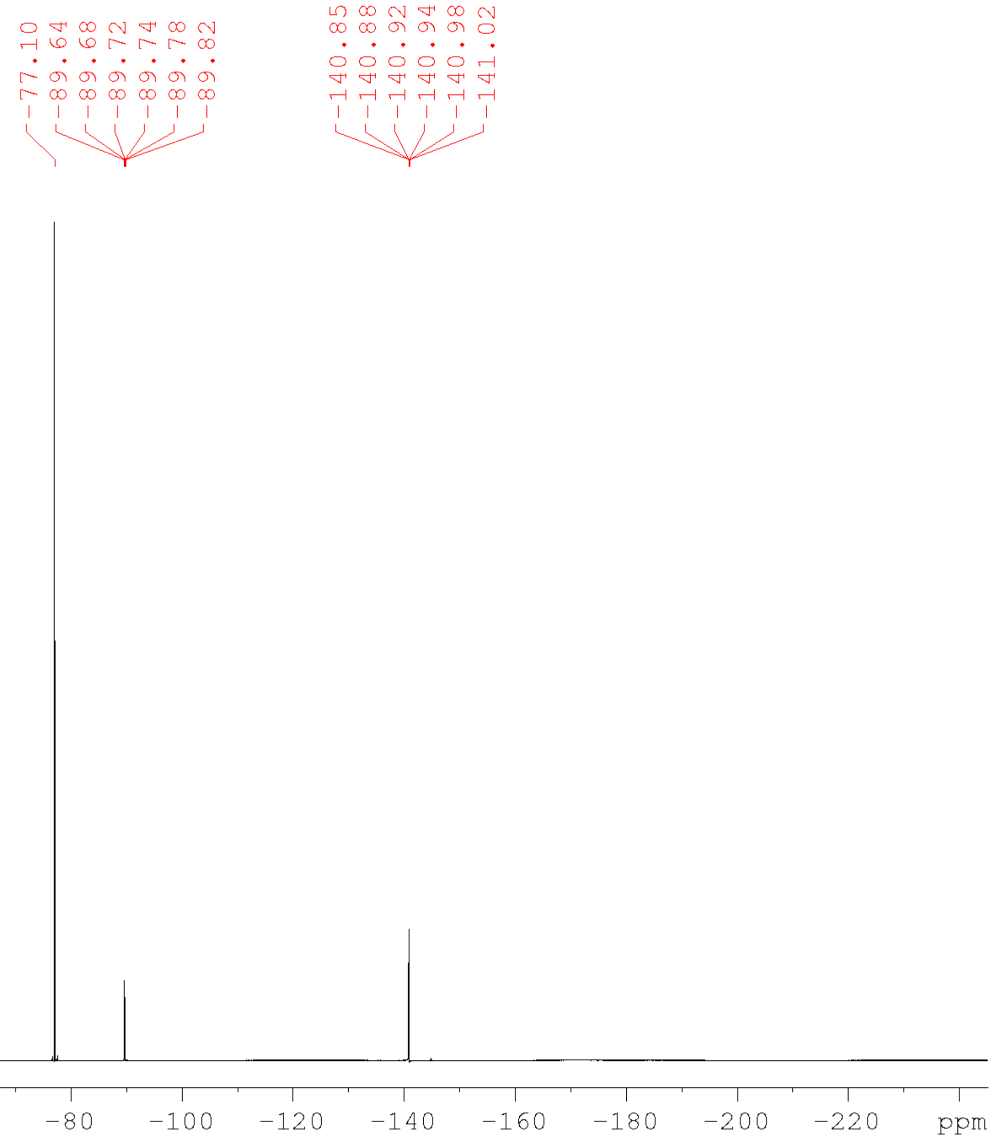

^{1}H NMR (400 MHZ, $CDCl_3$) **(5b)** N-(4-chloro-3-(2,3,5-trifluoropyridin-4-yl)benzyl)-1,1,1-trifluoromethanesulfonamide

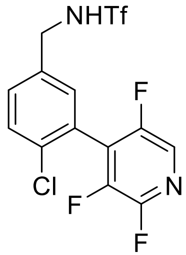

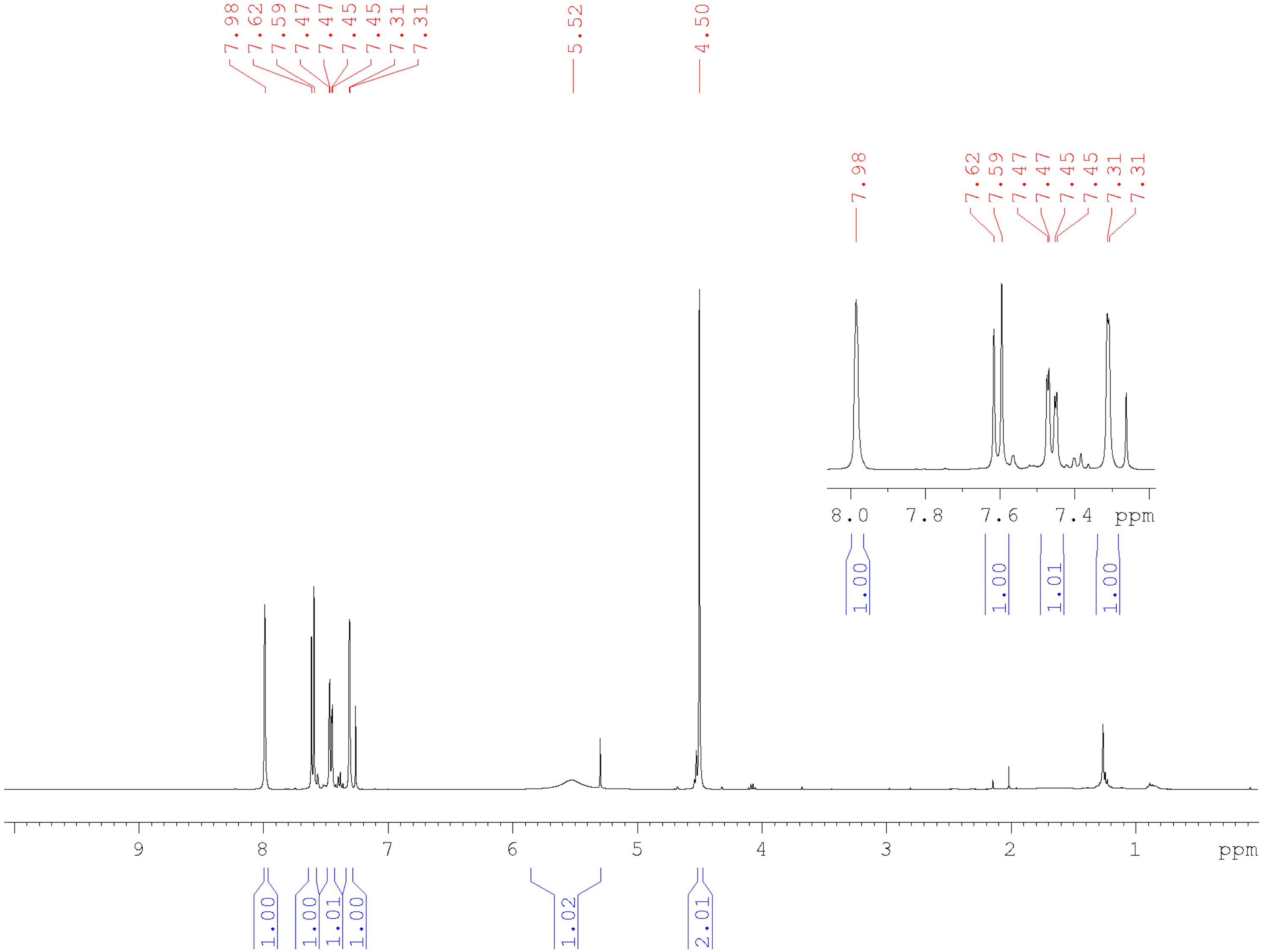

^{13}C NMR (101 MHZ, $CDCl_3$) **(5b)** N-(4-chloro-3-(2,3,5-trifluoropyridin-4-yl)benzyl)-1,1,1-trifluoromethanesulfonamide

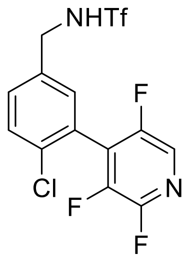

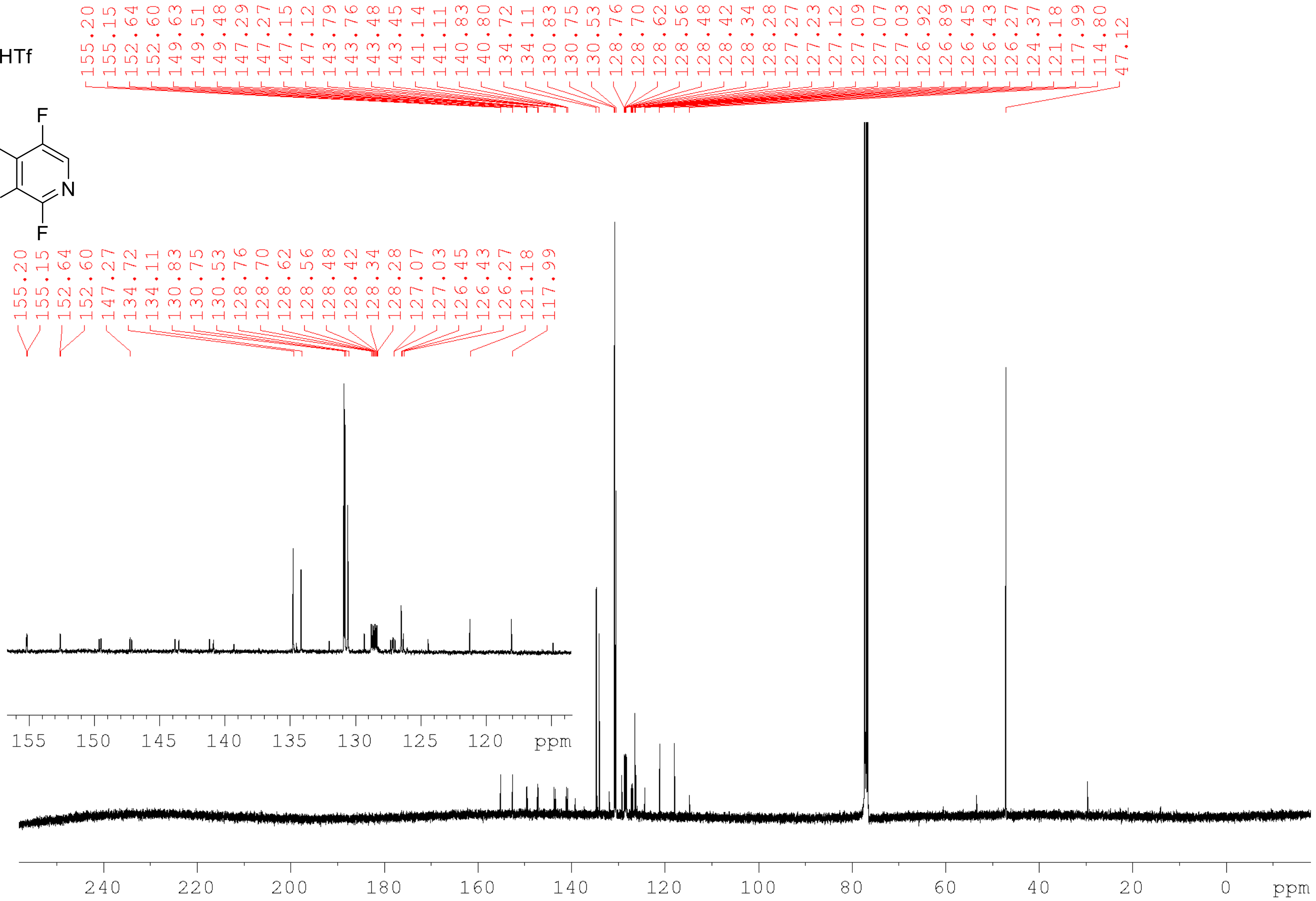

^{19}**F NMR** (376 MHZ, $CDCl_3$) **(5b)** N-(4-chloro-3-(2,3,5-trifluoropyridin-4-yl)benzyl)-1,1,1-trifluoromethanesulfonamide

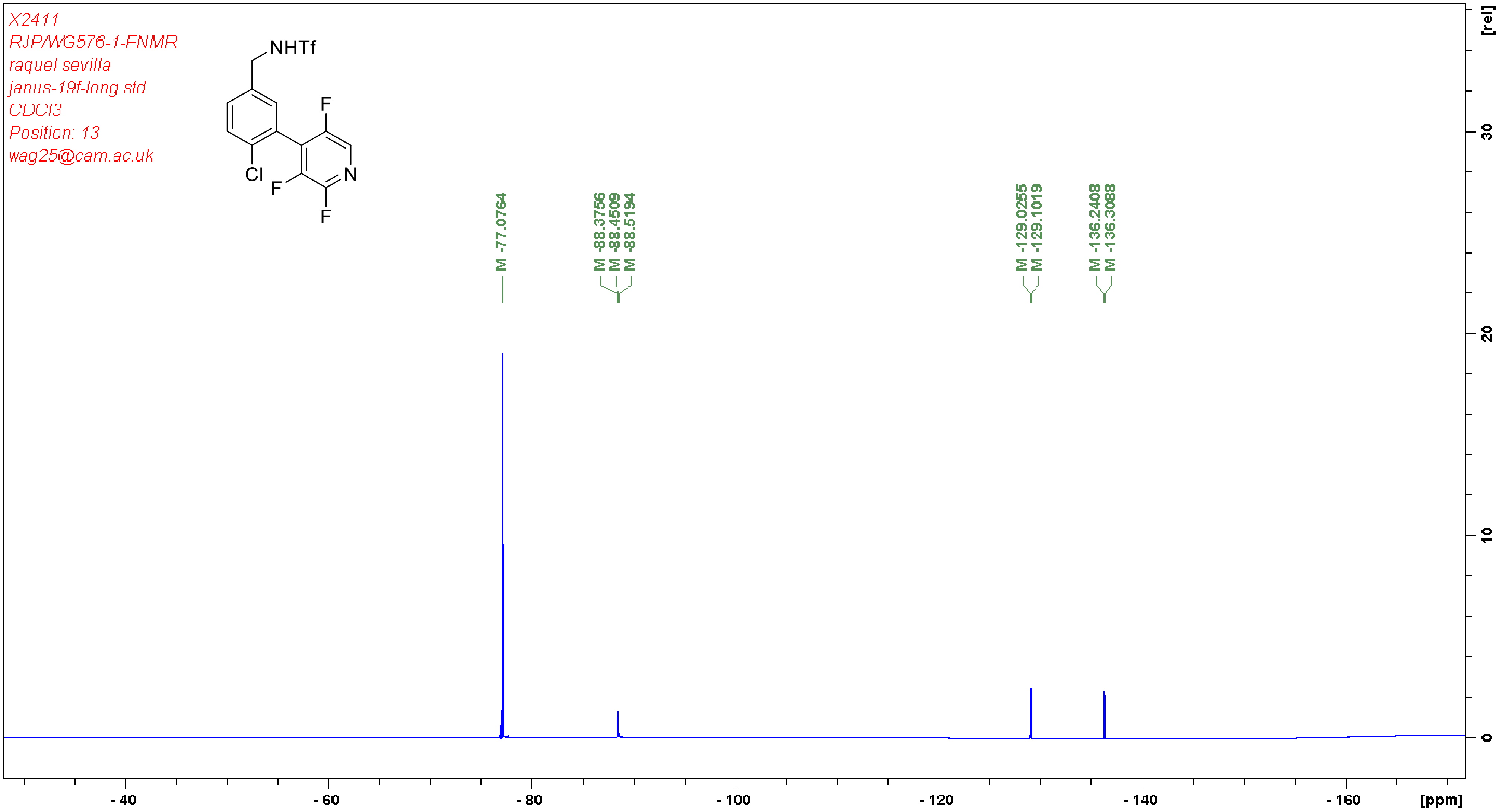

^{1}H NMR (400 MHZ, $CDCl_3$) **(5c)** N-(4-chloro-3-(2,4,6-trifluoropyridin-4-yl)benzyl)-1,1,1-trifluoromethanesulfonamide

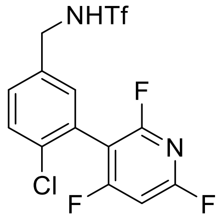

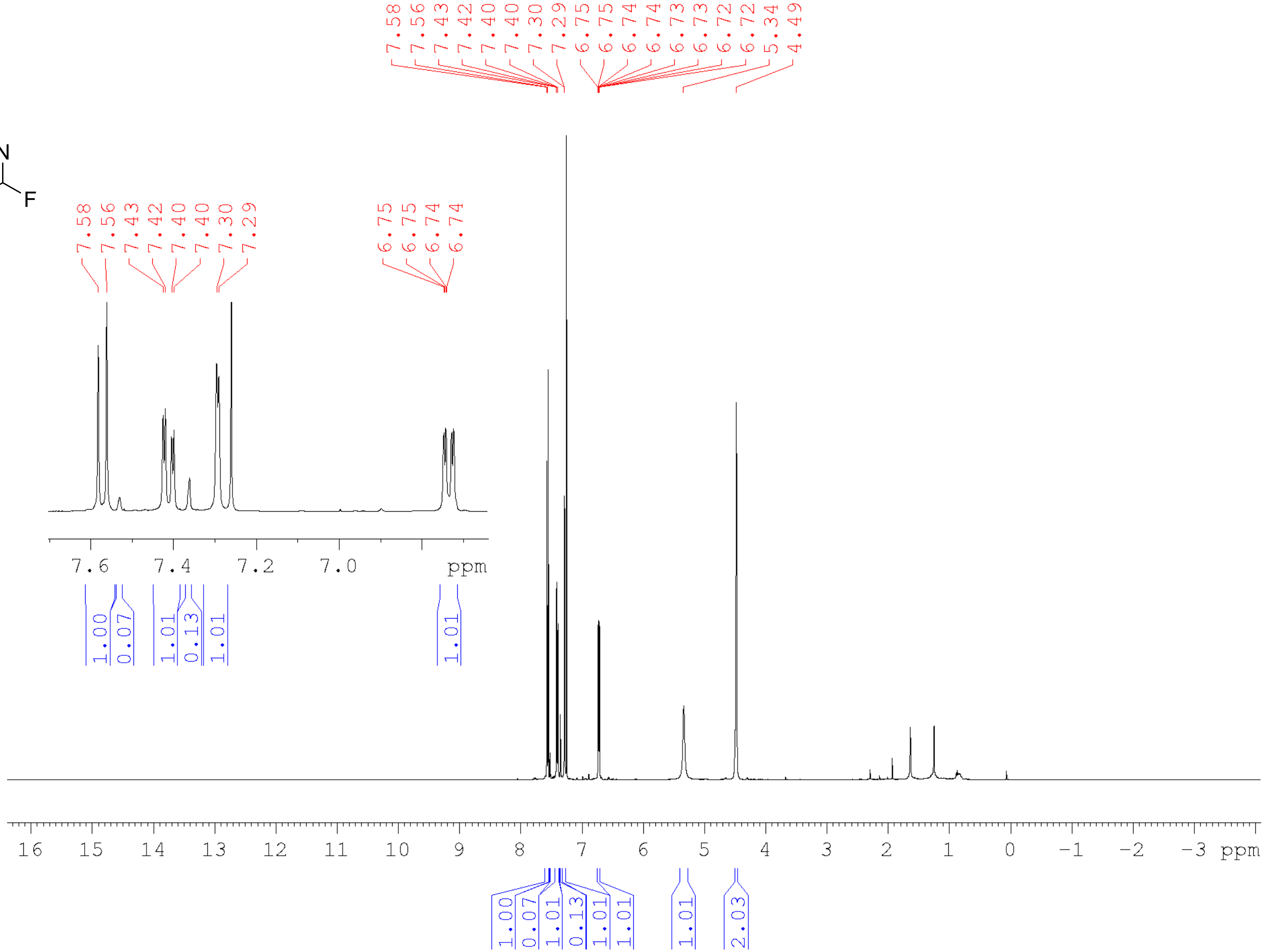

^{13}C NMR (101 MHZ, $CDCl_3$) **(5c)** N-(4-chloro-3-(2,4,6-trifluoropyridin-4-yl)benzyl)-1,1,1-trifluoromethanesulfonamide

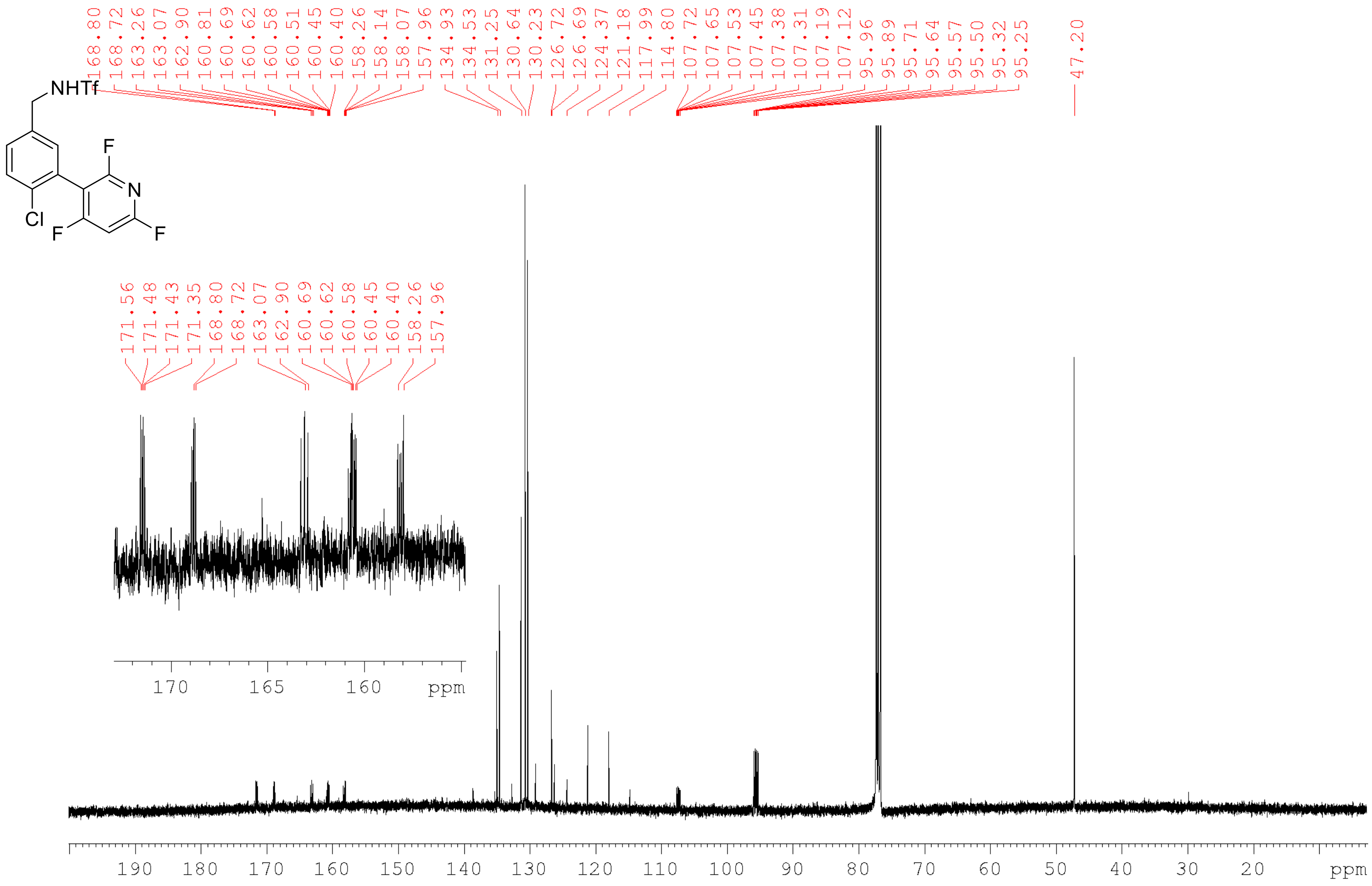

^{19}**F NMR** (376 MHZ, $CDCl_3$) **(5c)** N-(4-chloro-3-(2,4,6-trifluoropyridin-4-yl)benzyl)-1,1,1-trifluoromethanesulfonamide

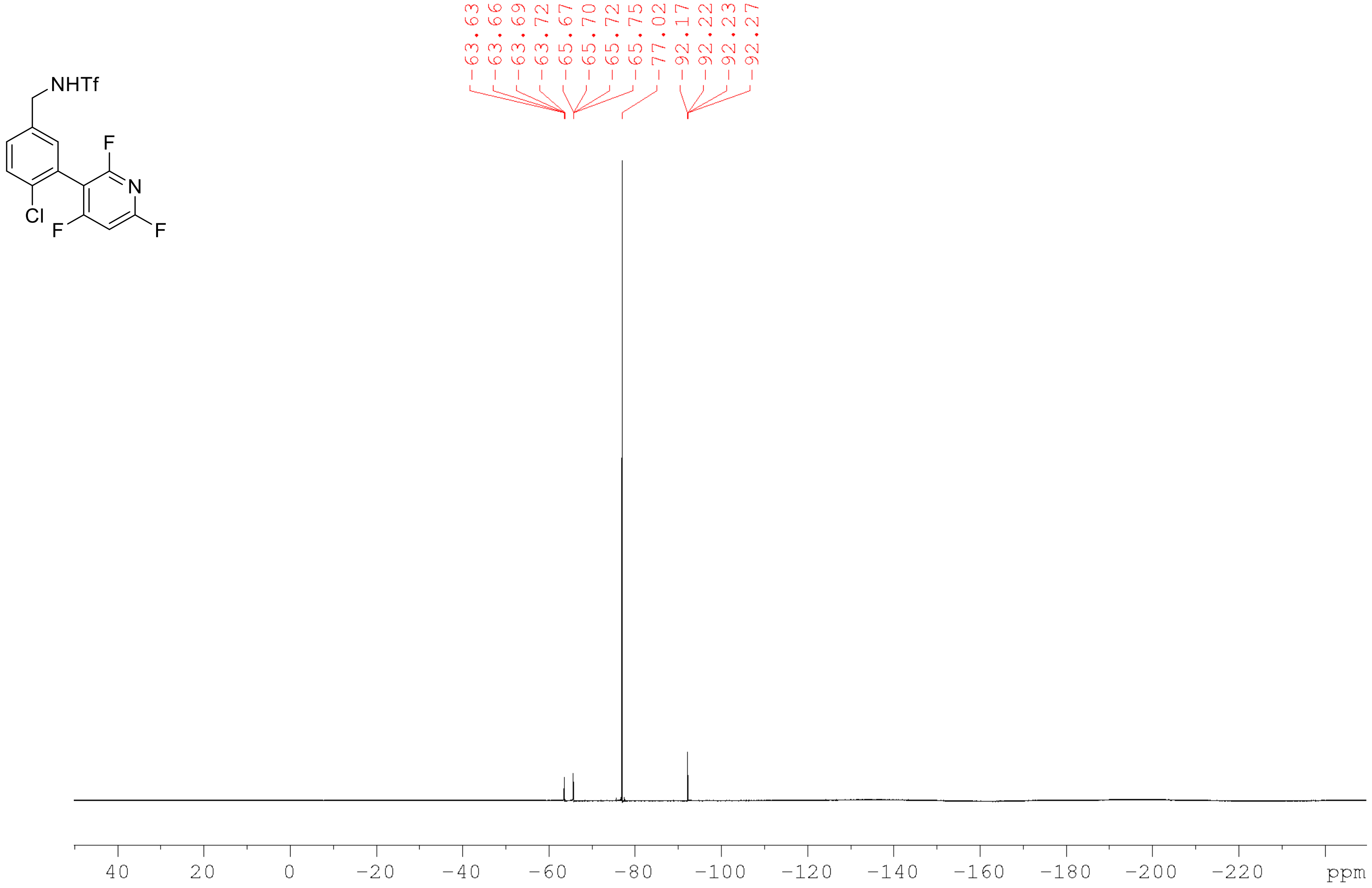

^{1}H NMR (400 MHZ, $CDCl_3$) **(5d)** N-(4-chloro-3-(3,5-diifluoropyridin-4-yl)benzyl)-1,1,1-trifluoromethanesulfonamide

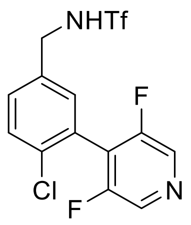

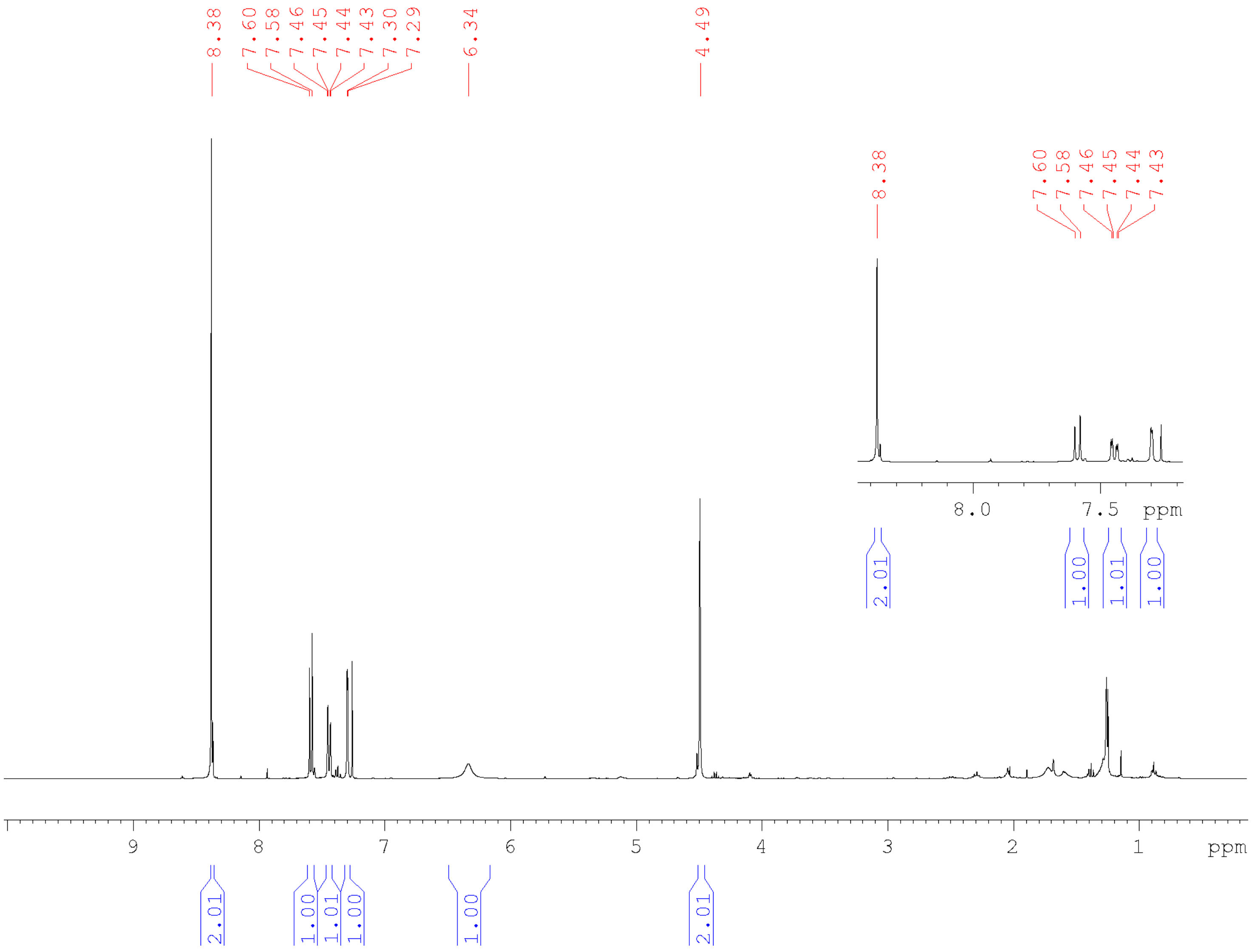

^{13}C NMR (101 MHZ, $CDCl_3$) **(5d)** N-(4-chloro-3-(3,5-diifluoropyridin-4-yl)benzyl)-1,1,1-trifluoromethanesulfonamide

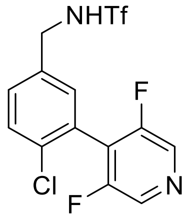

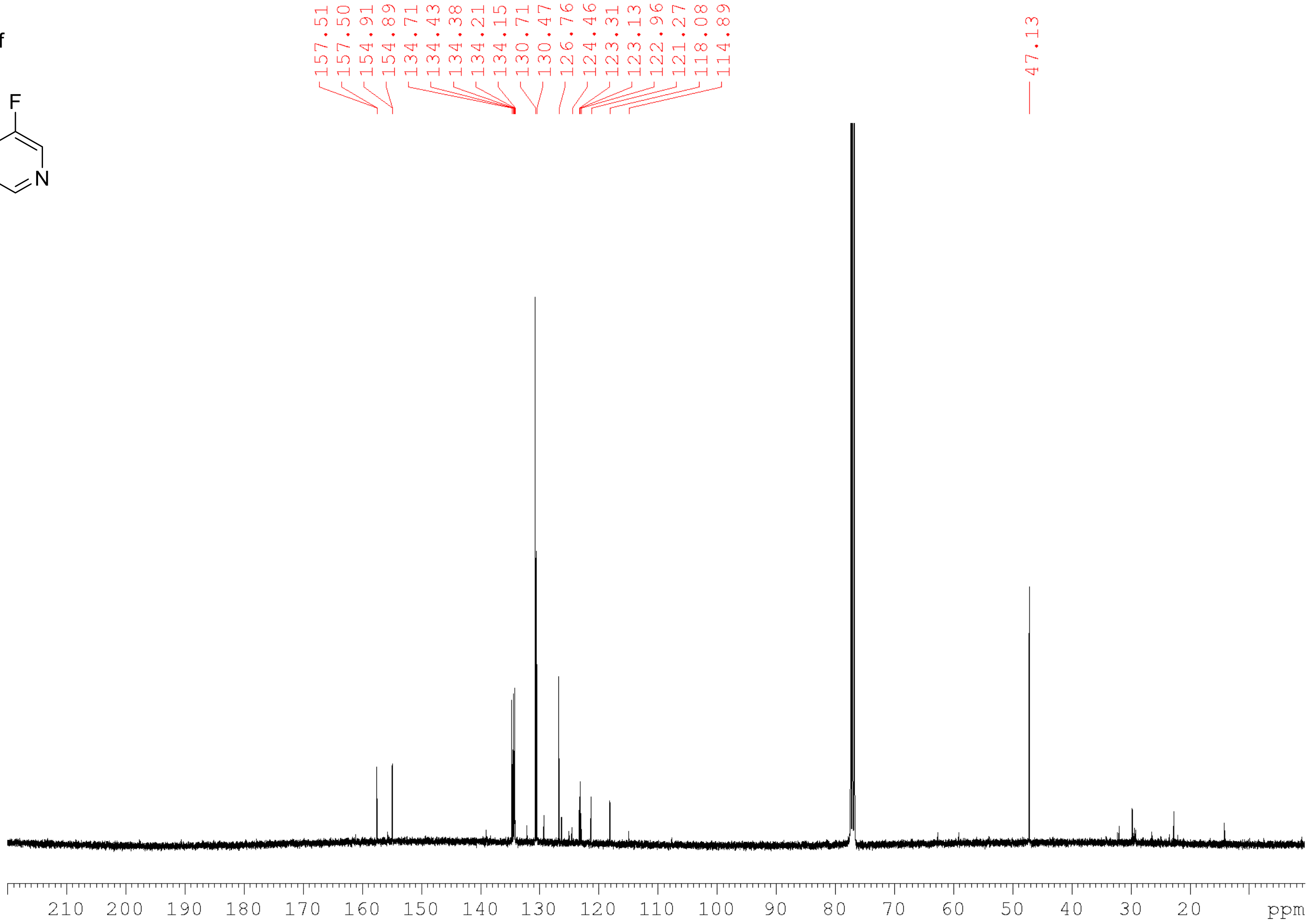

^{19}F NMR (376 MHZ, $CDCl_3$) **(5d)** N-(4-chloro-3-(3,5-diifluoropyridin-4-yl)benzyl)-1,1,1-trifluoromethanesulfonamide

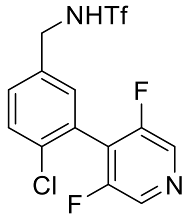

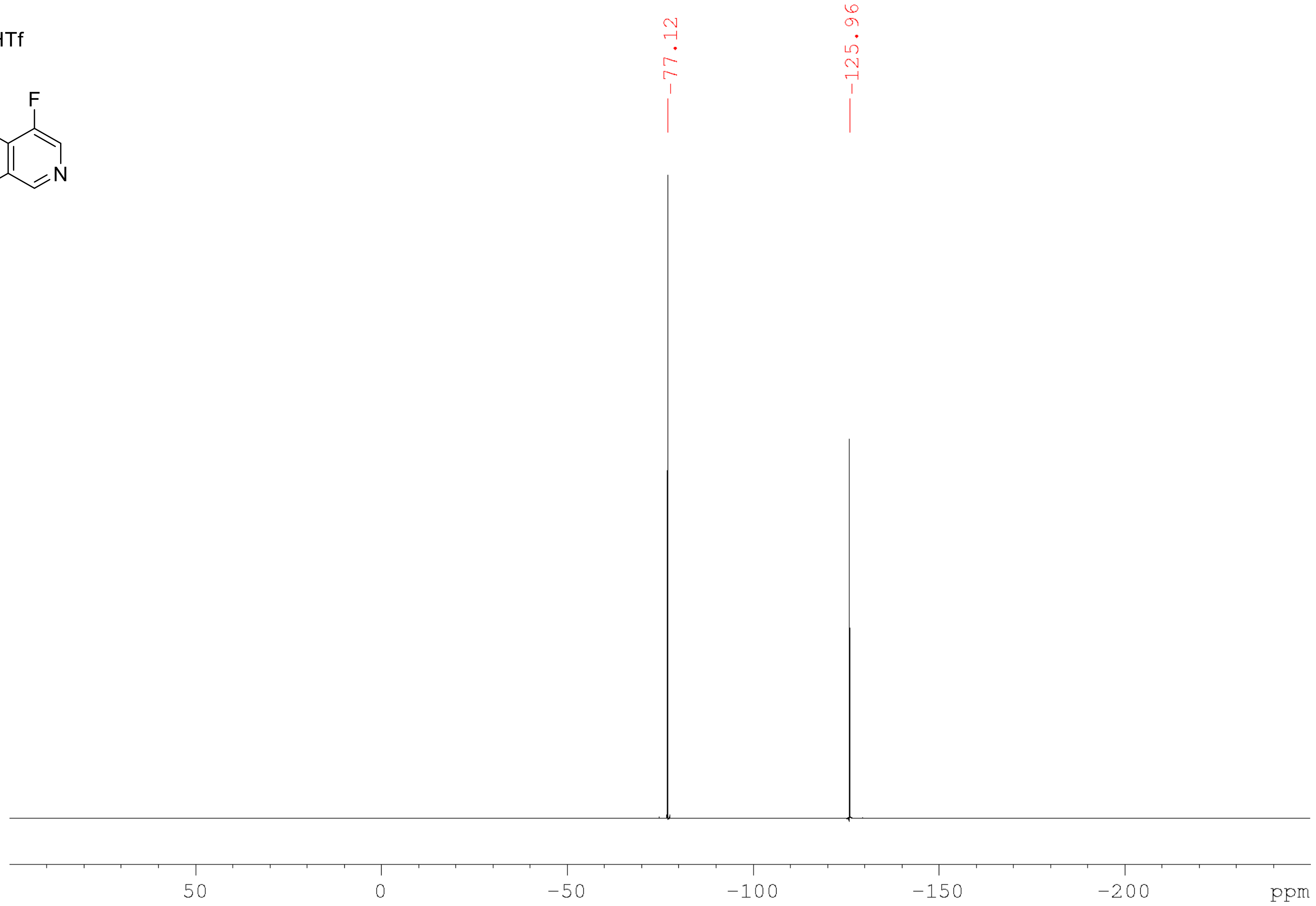

Dichloroarene scope

^{1}H NMR (400 MHZ, $CDCl_3$) **(5e)** N-(4-chloro-3-(perfluoropyridin-4-yl)phenethyl)-1,1,1-trifluoromethanesulfonamide

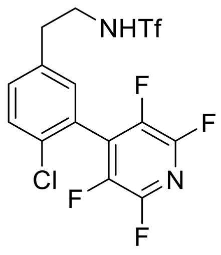

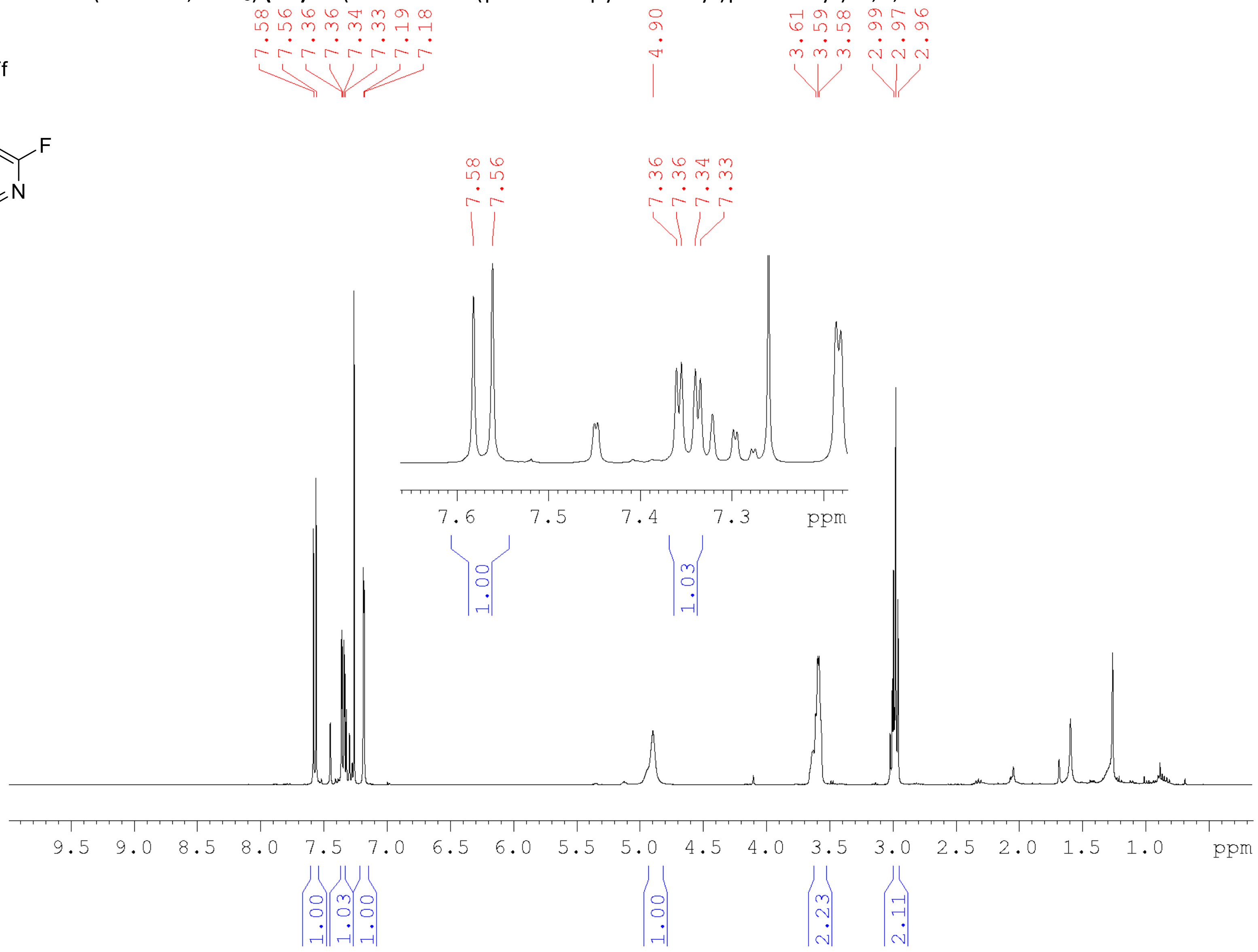

^{13}**C NMR** (101 MHZ, $CDCl_3$) **(5e)** N-(4-chloro-3-(perfluoropyridin-4-yl)phenethyl)-1,1,1-trifluoromethanesulfonamide

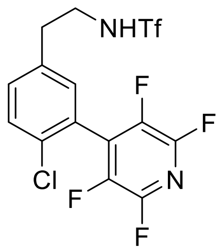

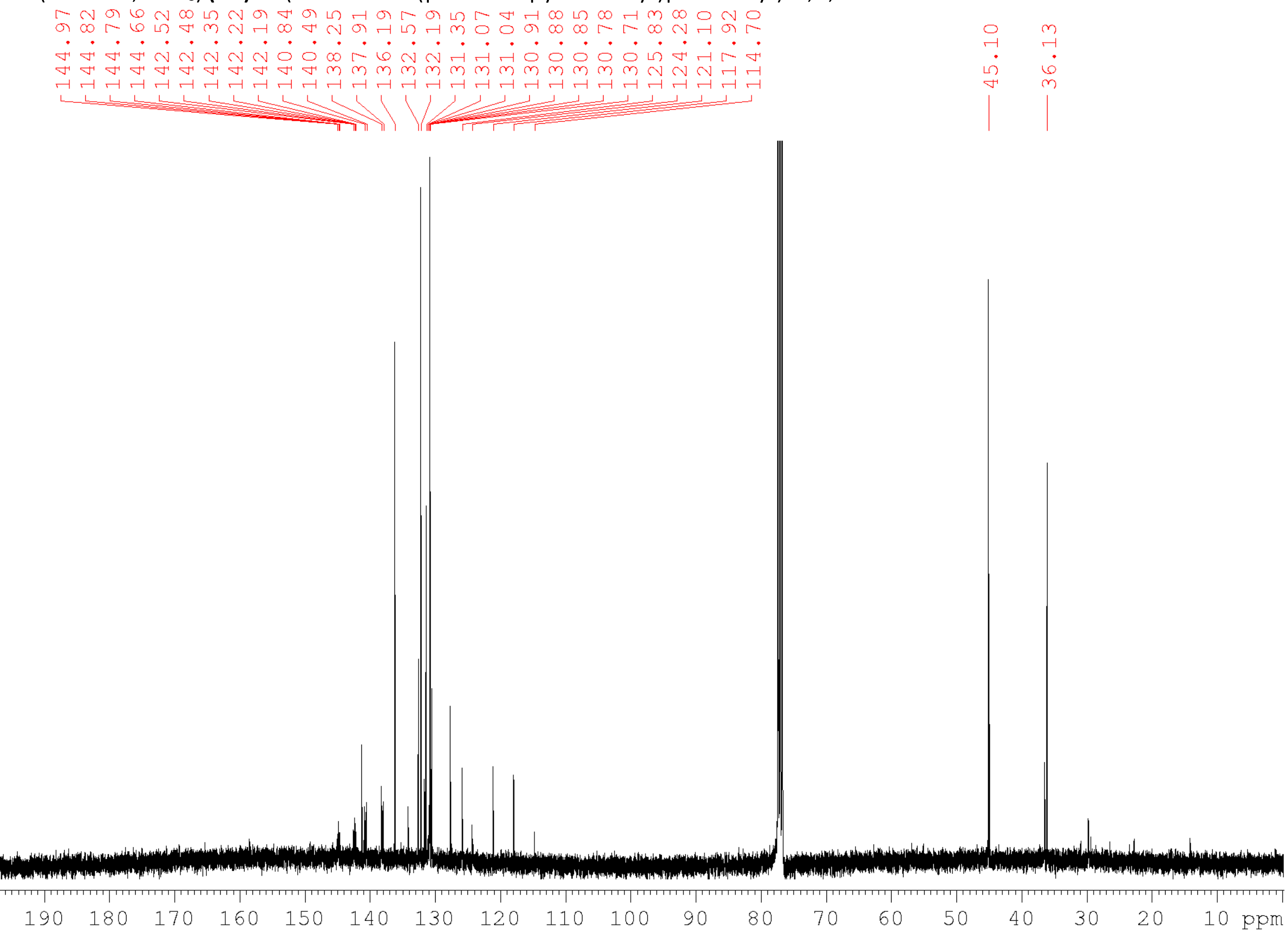

¹⁹F NMR (376 MHZ, $CDCl_3$) **(5e)** N-(4-chloro-3-(perfluoropyridin-4-yl)phenethyl)-1,1,1-trifluoromethanesulfonamide

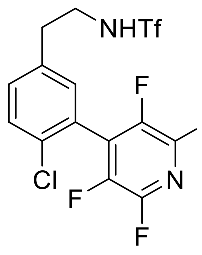

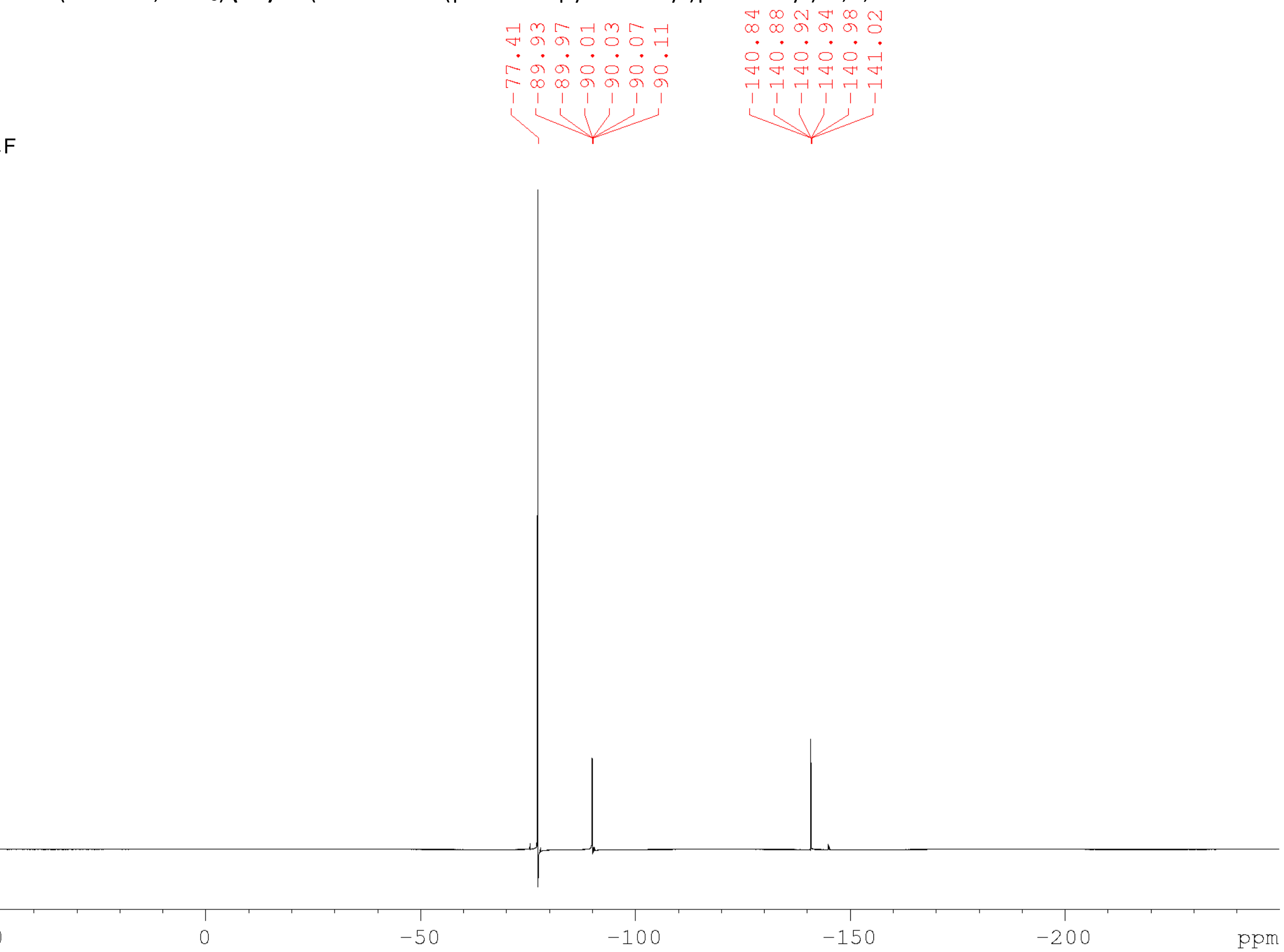

^{1}H NMR (400 MHZ, $CDCl_3$) **(5f)** N-(3-(4-chloro-3-(perfluoropyridin-4-yl)phenyl)propyl)-1,1,1-trifluoromethanesulfonamide

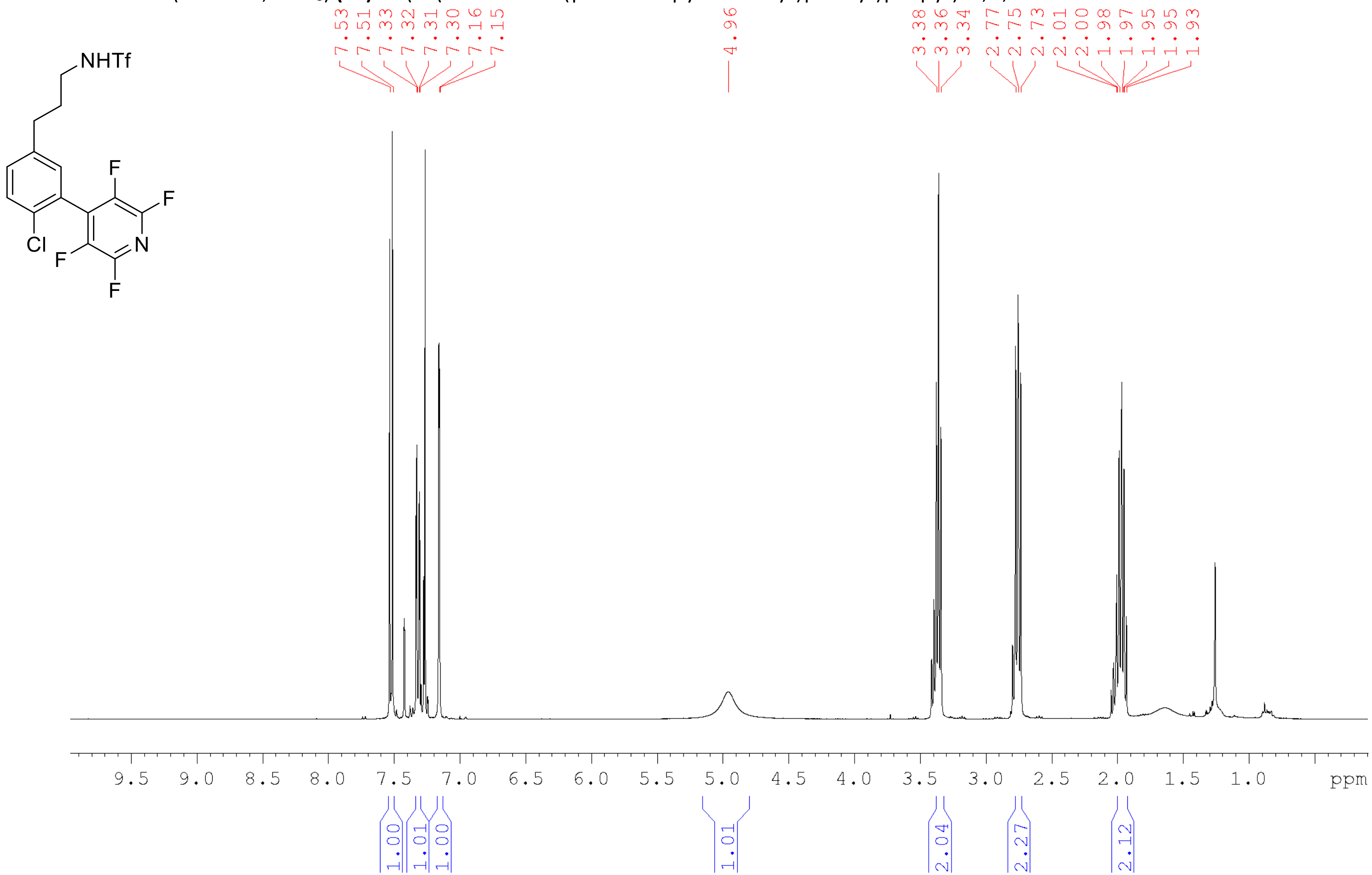

^{13}C NMR (101 MHZ, $CDCl_3$) **(5f)** N-(3-(4-chloro-3-(perfluoropyridin-4-yl)phenyl)propyl)-1,1,1-trifluoromethanesulfonamide

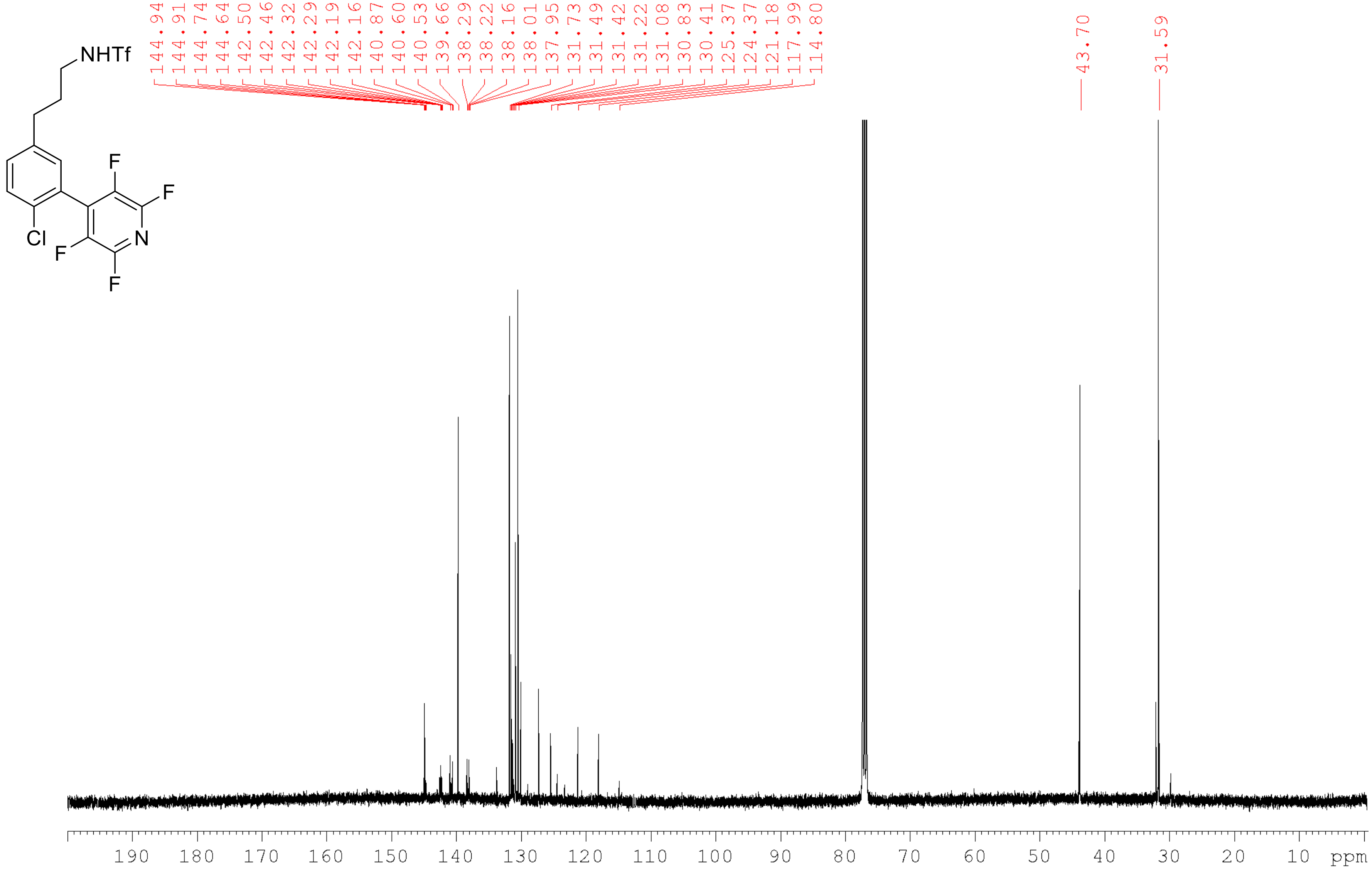

^{19}**F NMR** (376 MHZ, $CDCl_3$) **(5f)** N-(3-(4-chloro-3-(perfluoropyridin-4-yl)phenyl)propyl)-1,1,1-trifluoromethanesulfonamide

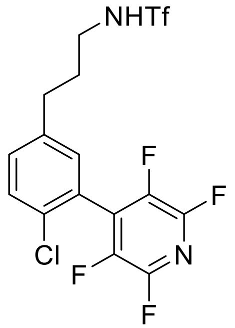

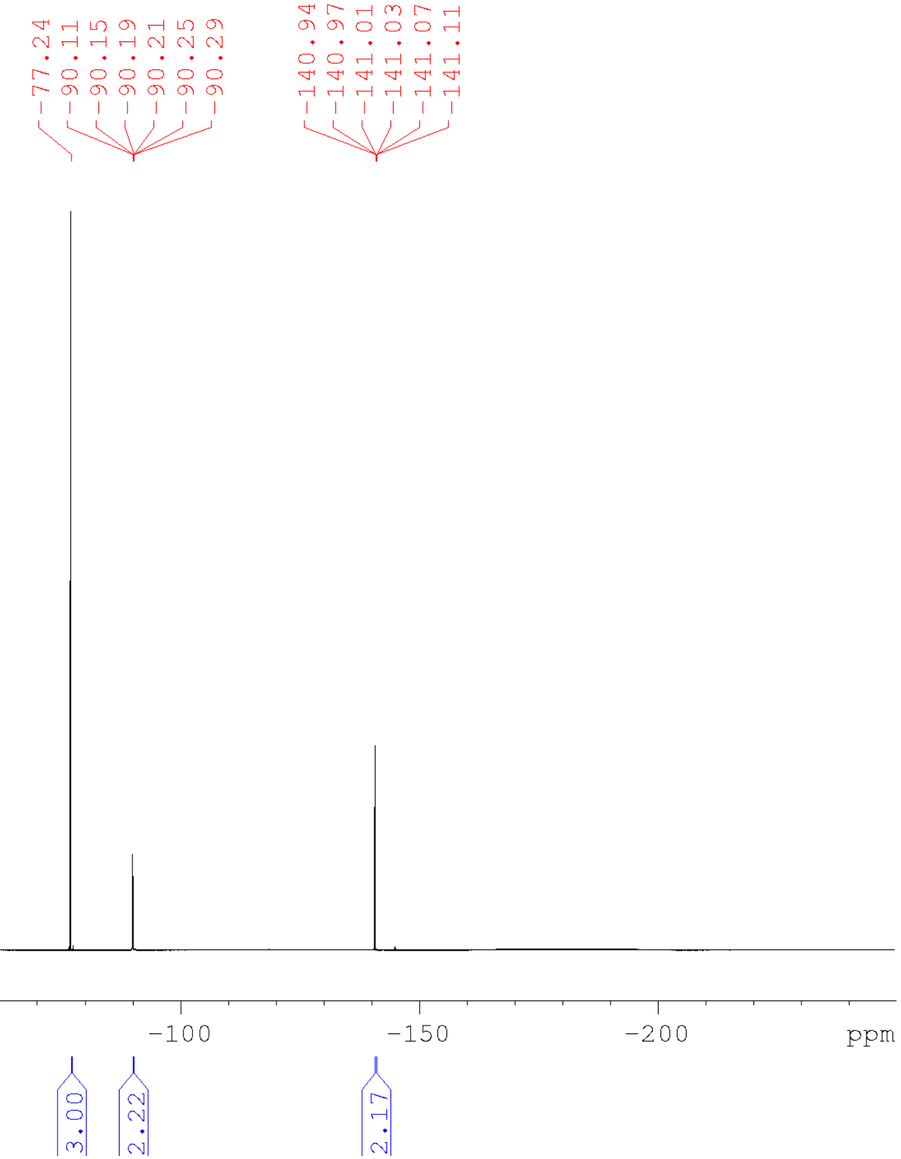

¹H NMR (400 MHZ, $CDCl_3$) **(5g)** N-(1-(4-chloro-3-(perfluoropyridin-4-yl)phenyl)ethyl)-1,1,1-trifluoromethanesulfonamide

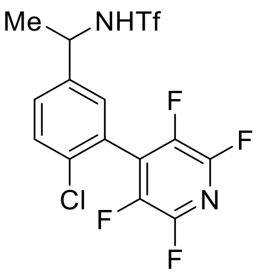

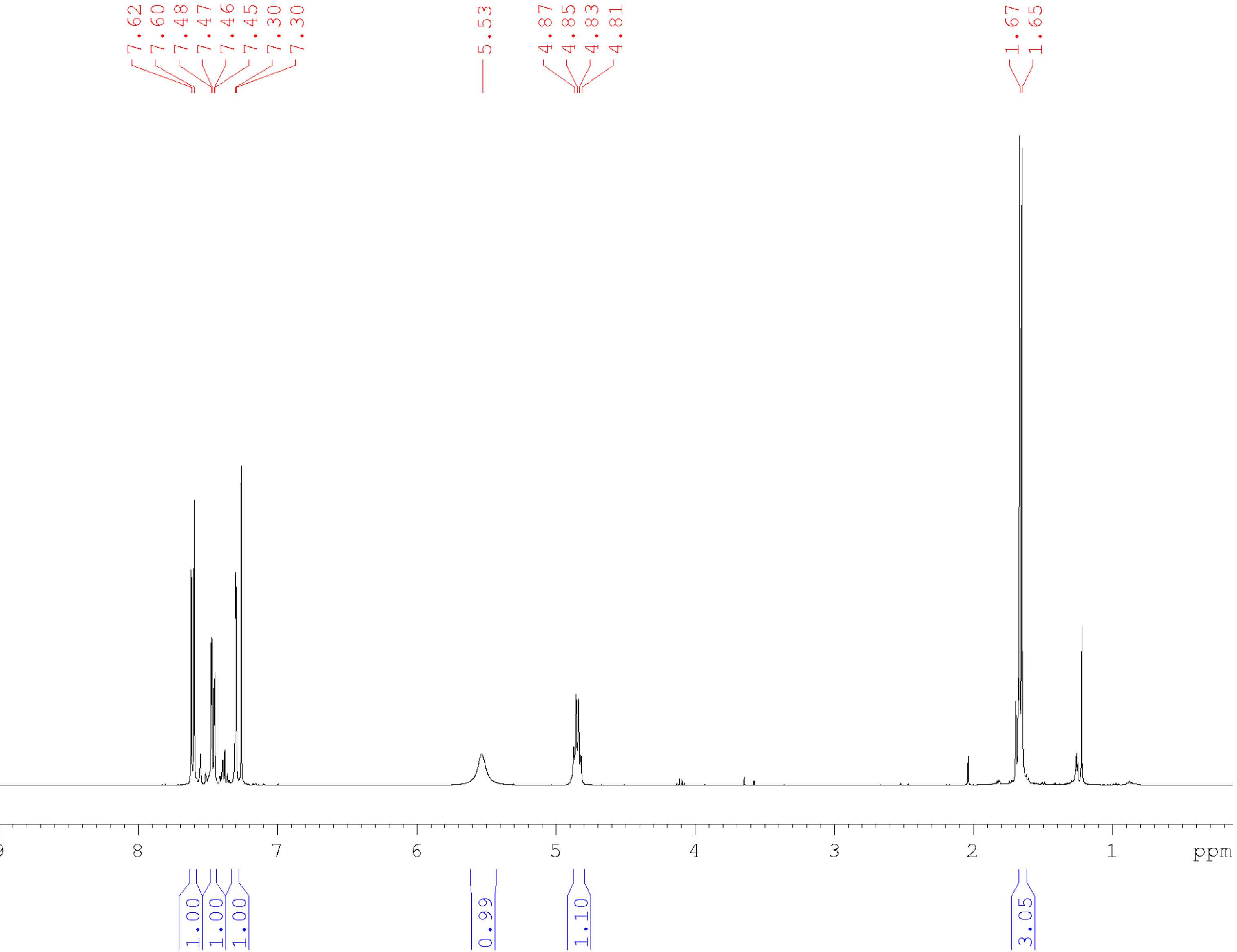

^{13}C NMR (101 MHZ, $CDCl_3$) **(5g)** N-(1-(4-chloro-3-(perfluoropyridin-4-yl)phenyl)ethyl)-1,1,1-trifluoromethanesulfonamide

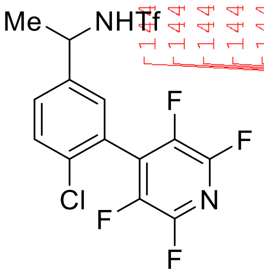

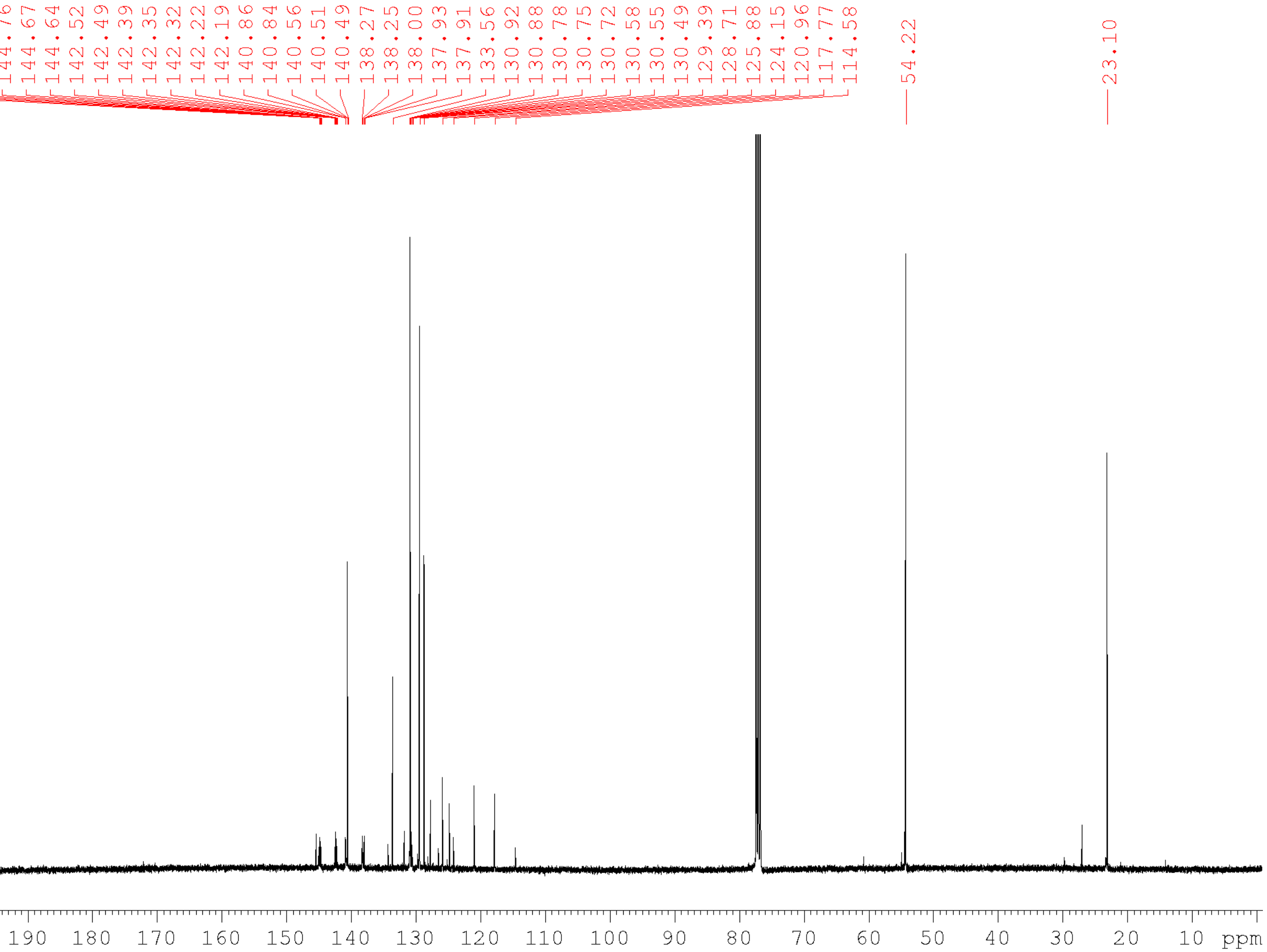

^{19}F NMR (376 MHZ, $CDCl_3$) **(5g)** N-(1-(4-chloro-3-(perfluoropyridin-4-yl)phenyl)ethyl)-1,1,1-trifluoromethanesulfonamide

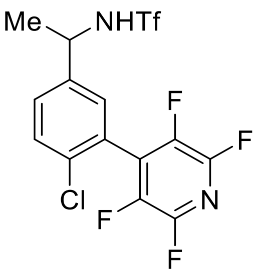

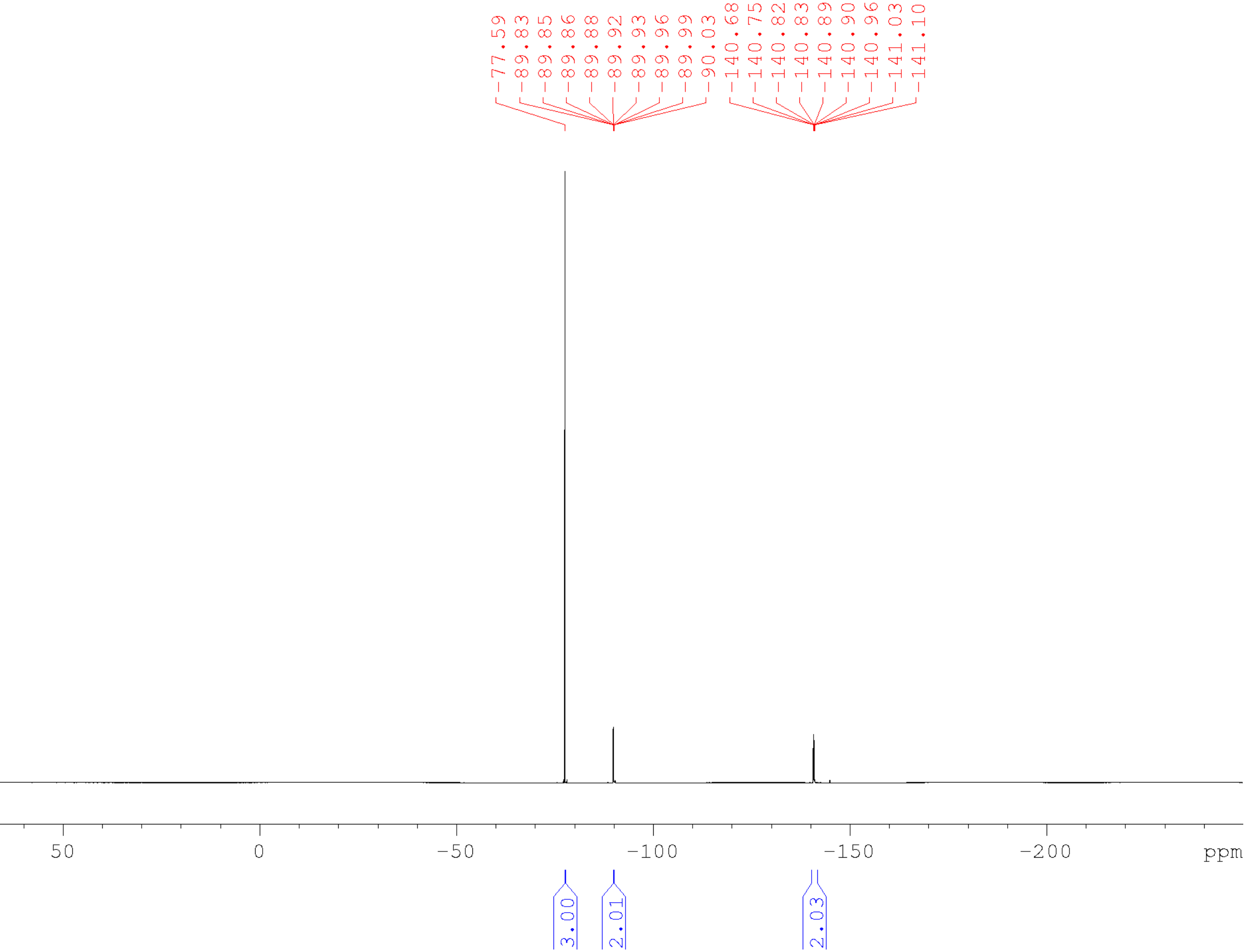

^{1}H NMR (400 MHZ, $CDCl_3$) **(5h)** N-((4-chloro-3-(perfluoropyridin-4-yl)phenyl)(phenyl)methyl)-1,1,1-trifluoromethanesulfonamide

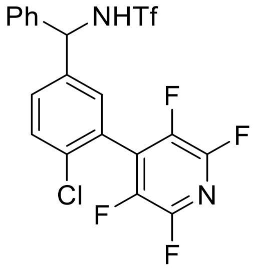

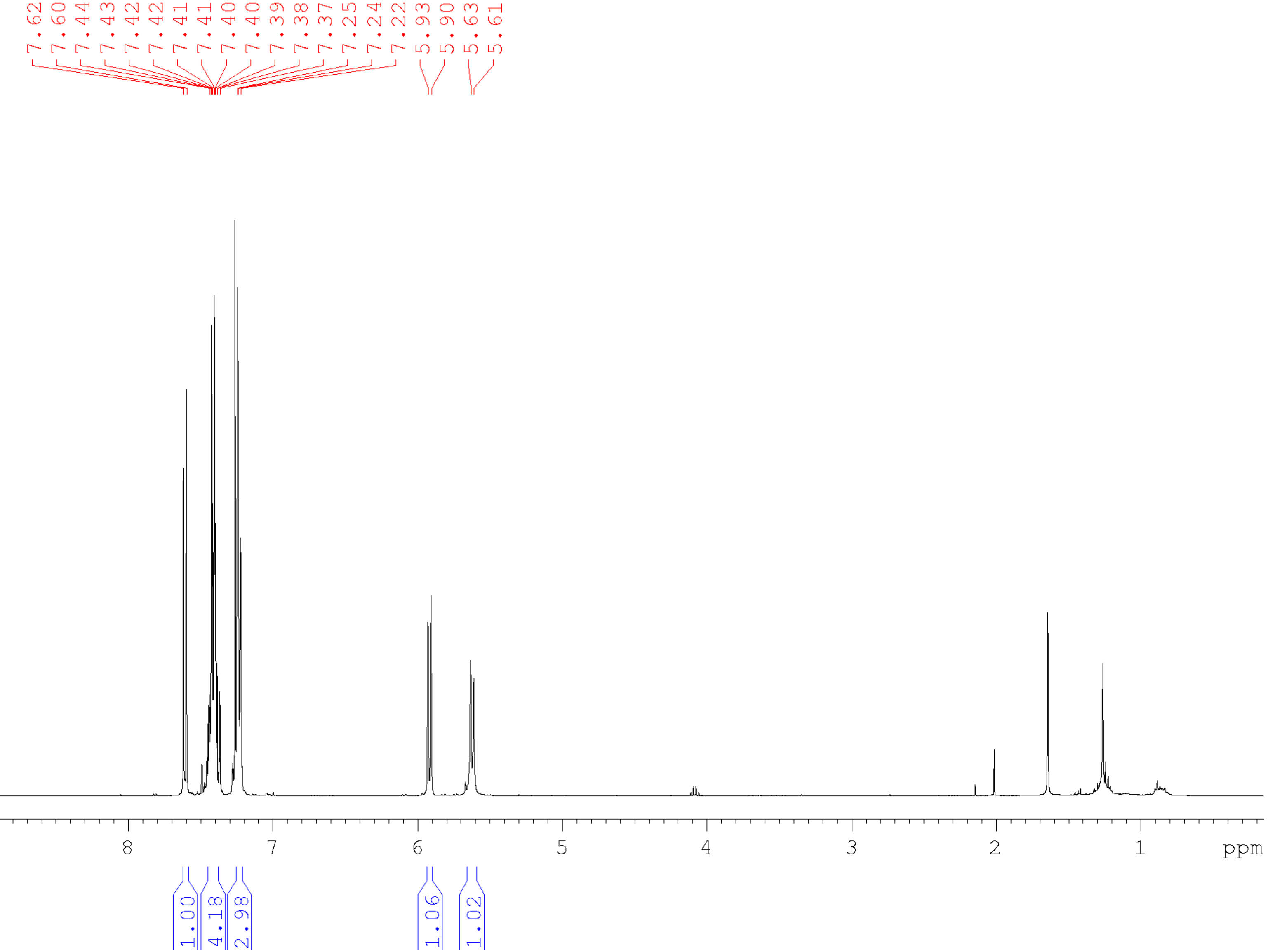

^{13}C NMR (101 MHZ, CDCl$_3$) **(5h)** N-((4-chloro-3-(perfluoropyridin-4-yl)phenyl)(phenyl)methyl)-1,1,1-trifluoromethanesulfonamide

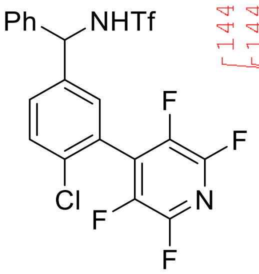

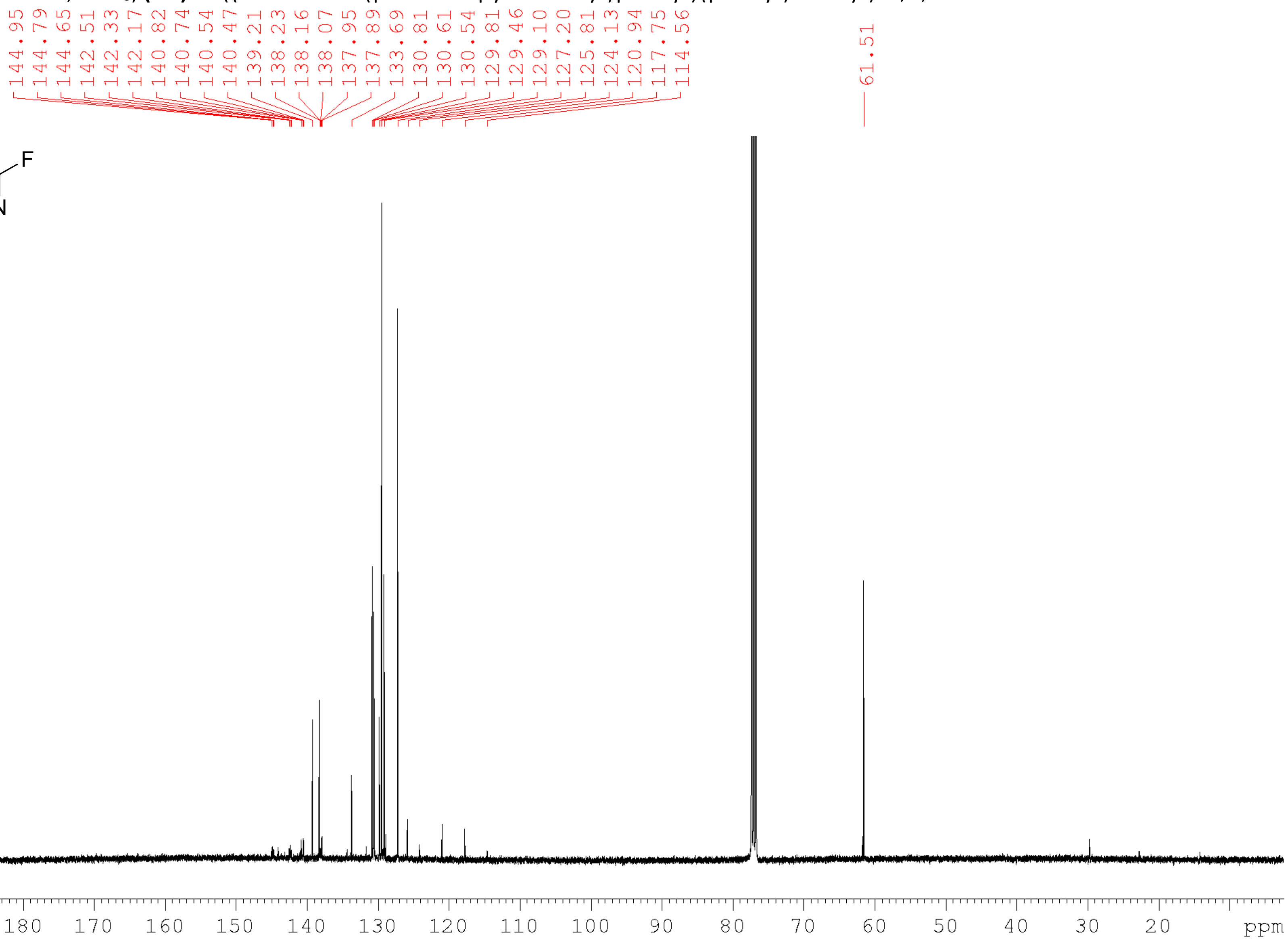

^{19}F NMR (376 MHZ, $CDCl_3$) **(5h)** N-((4-chloro-3-(perfluoropyridin-4-yl)phenyl)(phenyl)methyl)-1,1,1-trifluoromethanesulfonamide

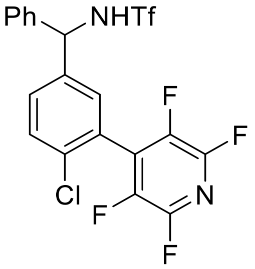

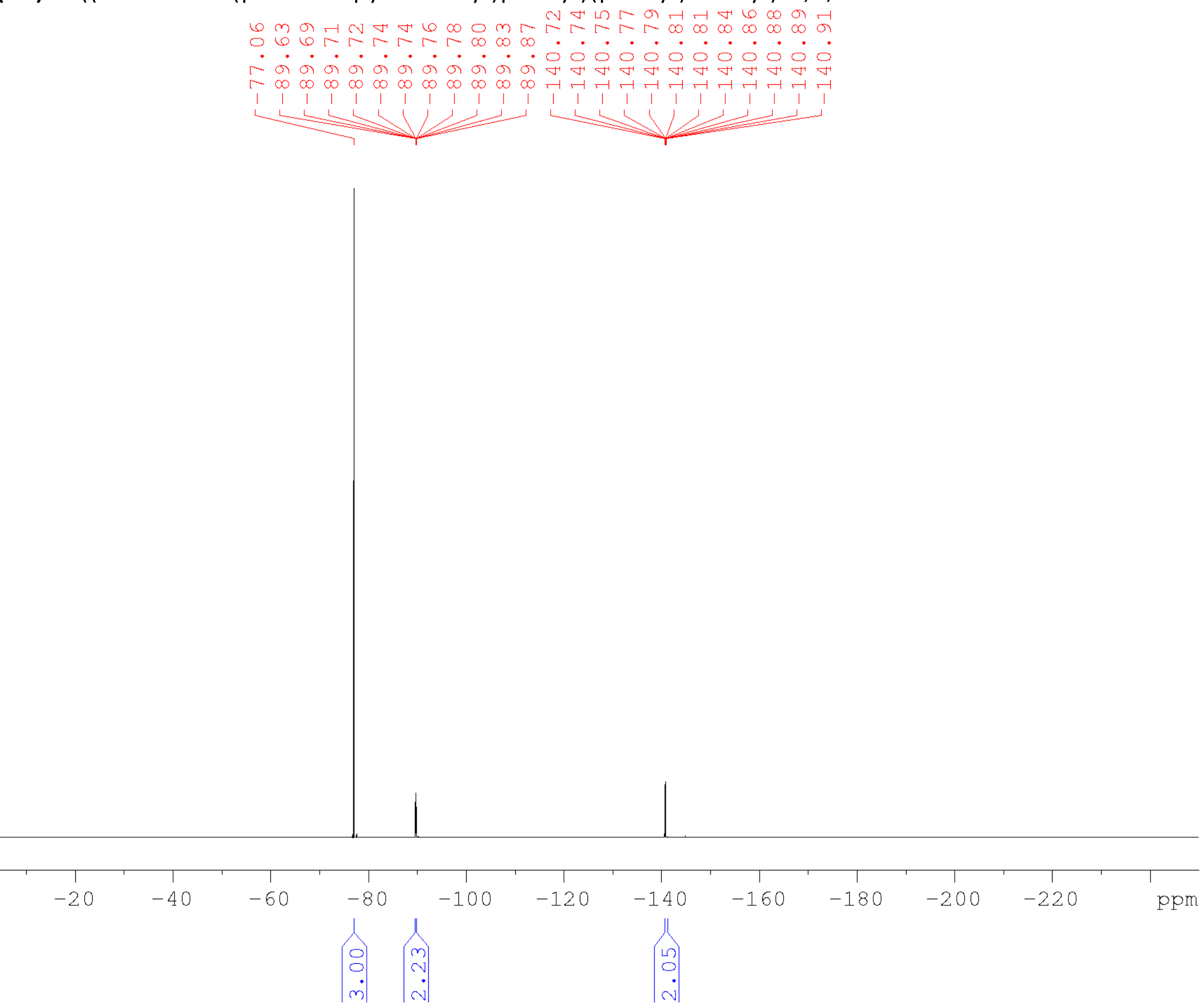

^{1}H NMR (400 MHZ, $CDCl_3$) **(5i)** N-butyl-1-(4-chloro-3-(perfluoropyridin-4-yl)phenyl)methanesulfonamide

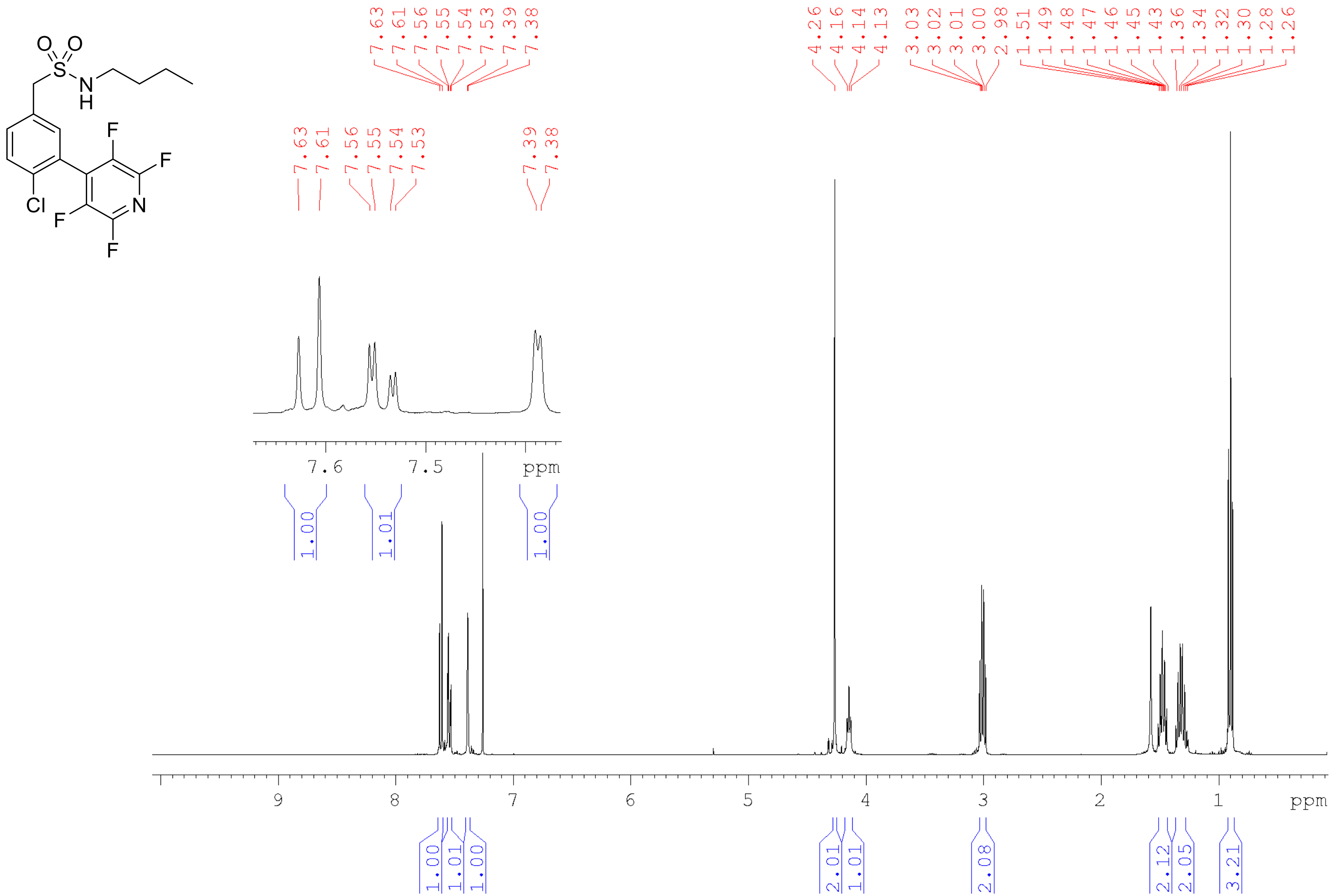

^{13}C NMR (101 MHZ, $CDCl_3$) **(5i)** N-butyl-1-(4-chloro-3-(perfluoropyridin-4-yl)phenyl)methanesulfonamide

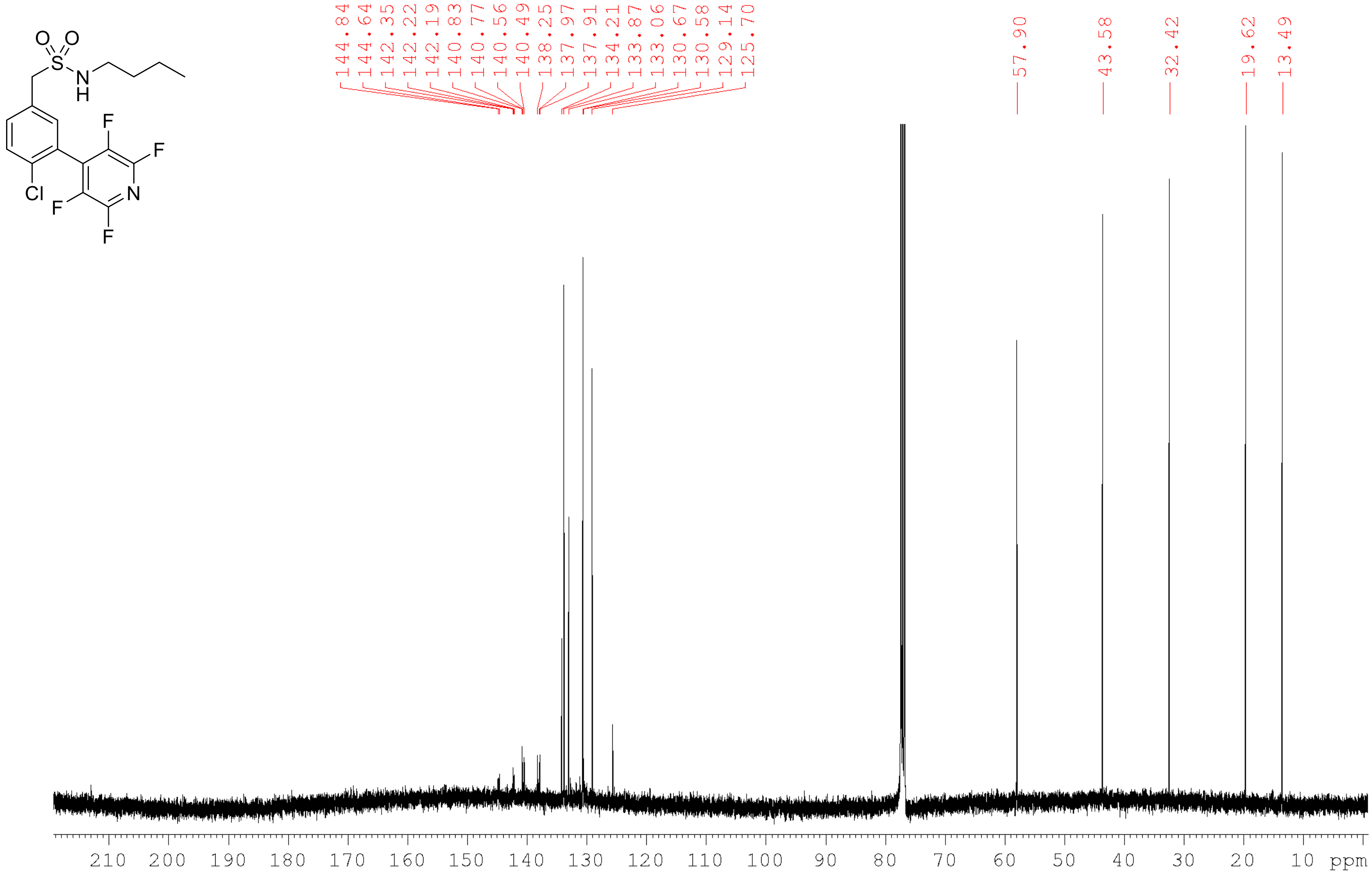

^{19}F NMR (376 MHZ, $CDCl_3$) **(5i)** N-butyl-1-(4-chloro-3-(perfluoropyridin-4-yl)phenyl)methanesulfonamide

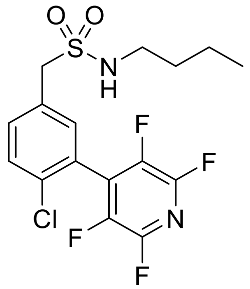

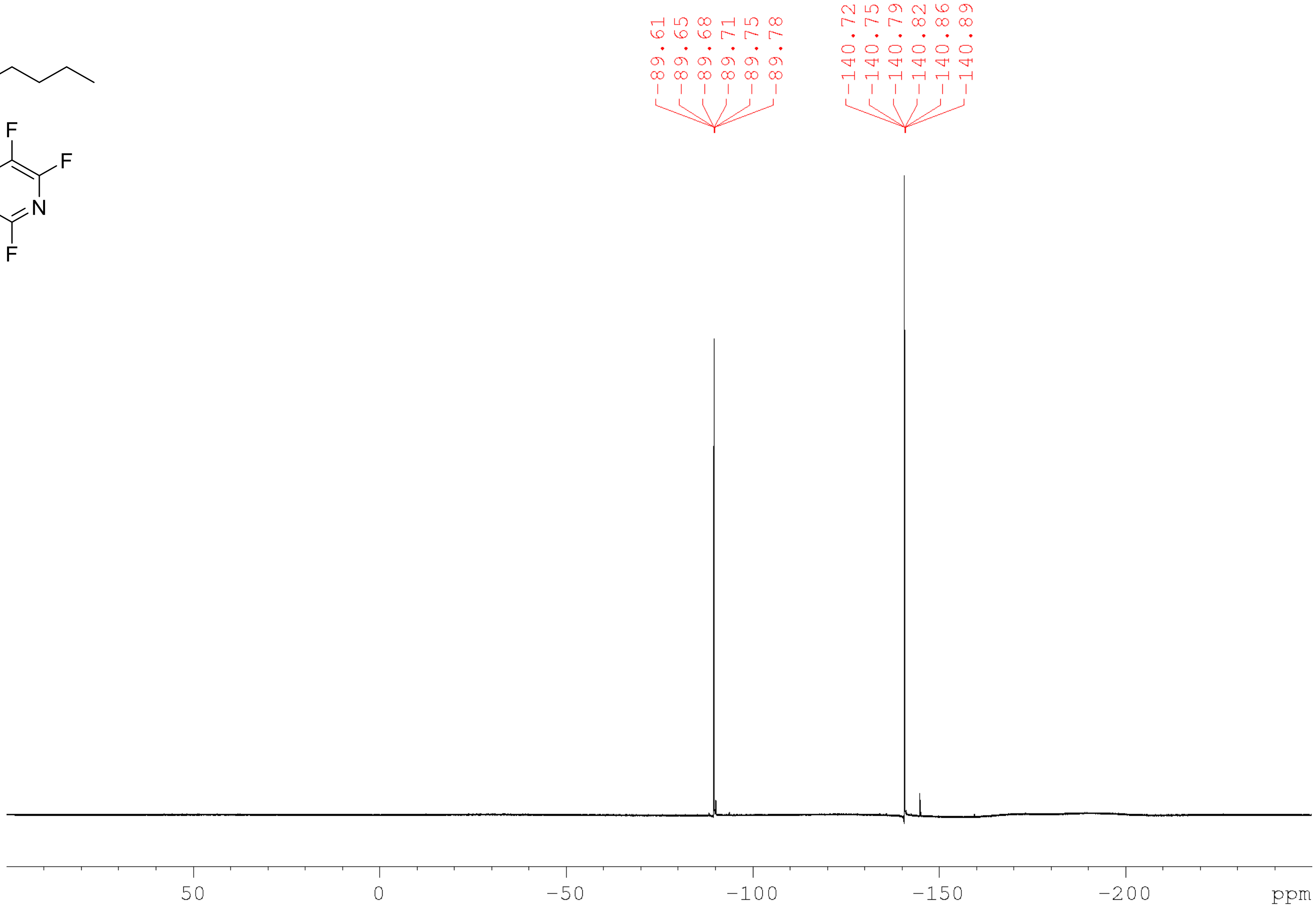

^{1}H NMR (400 MHZ, $CDCl_3$) **(5j)** N-(4-chloro-3-(perfluoropyridin-4-yl)benzyl)-2,2,2-trifluoroacetamide

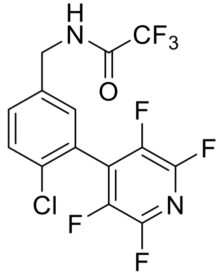

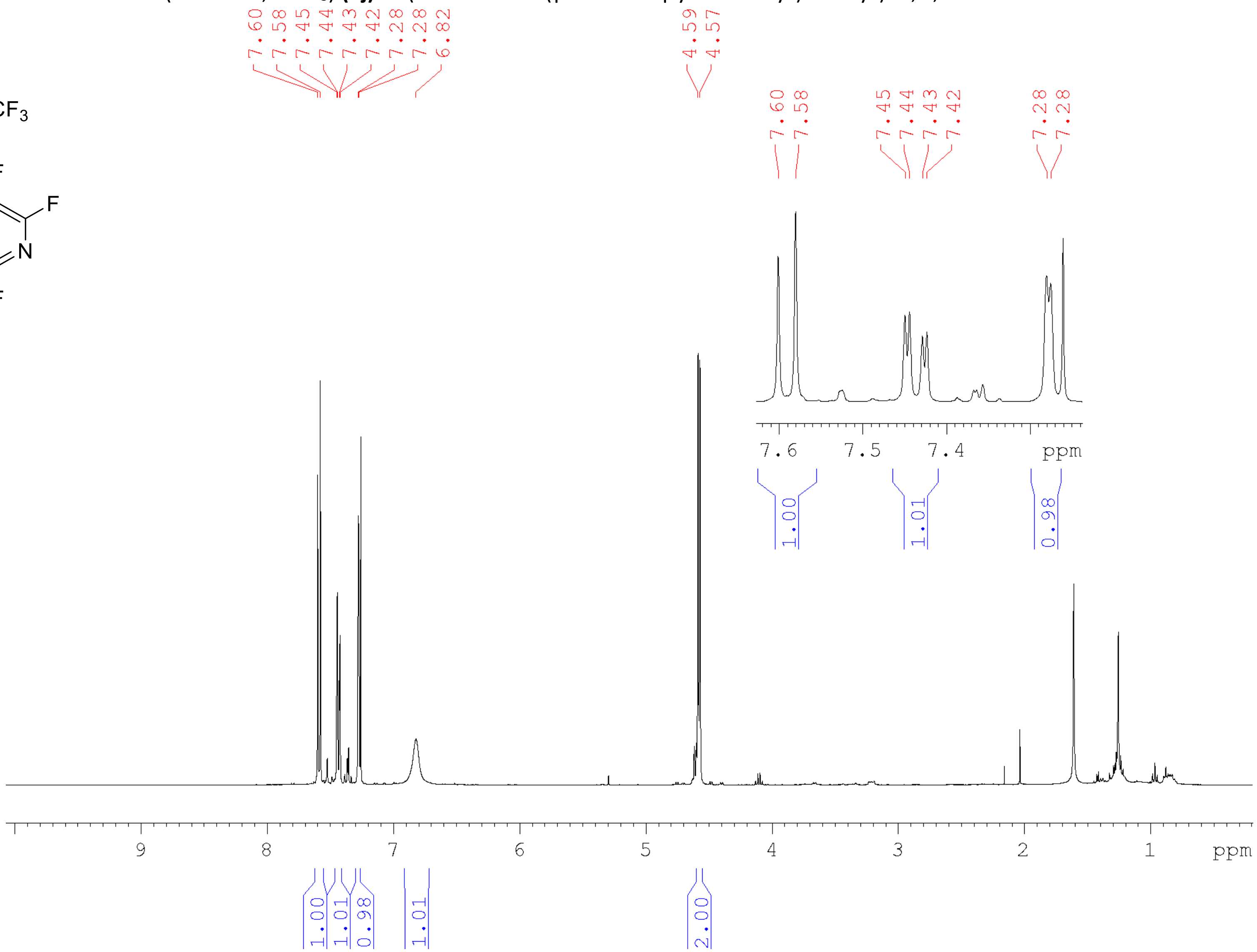

^{13}C NMR (101 MHZ, $CDCl_3$) **(5j)** N-(4-chloro-3-(perfluoropyridin-4-yl)benzyl)-2,2,2-trifluoroacetamide

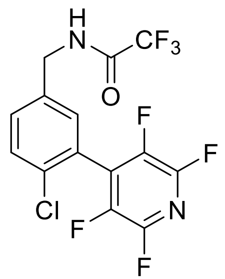

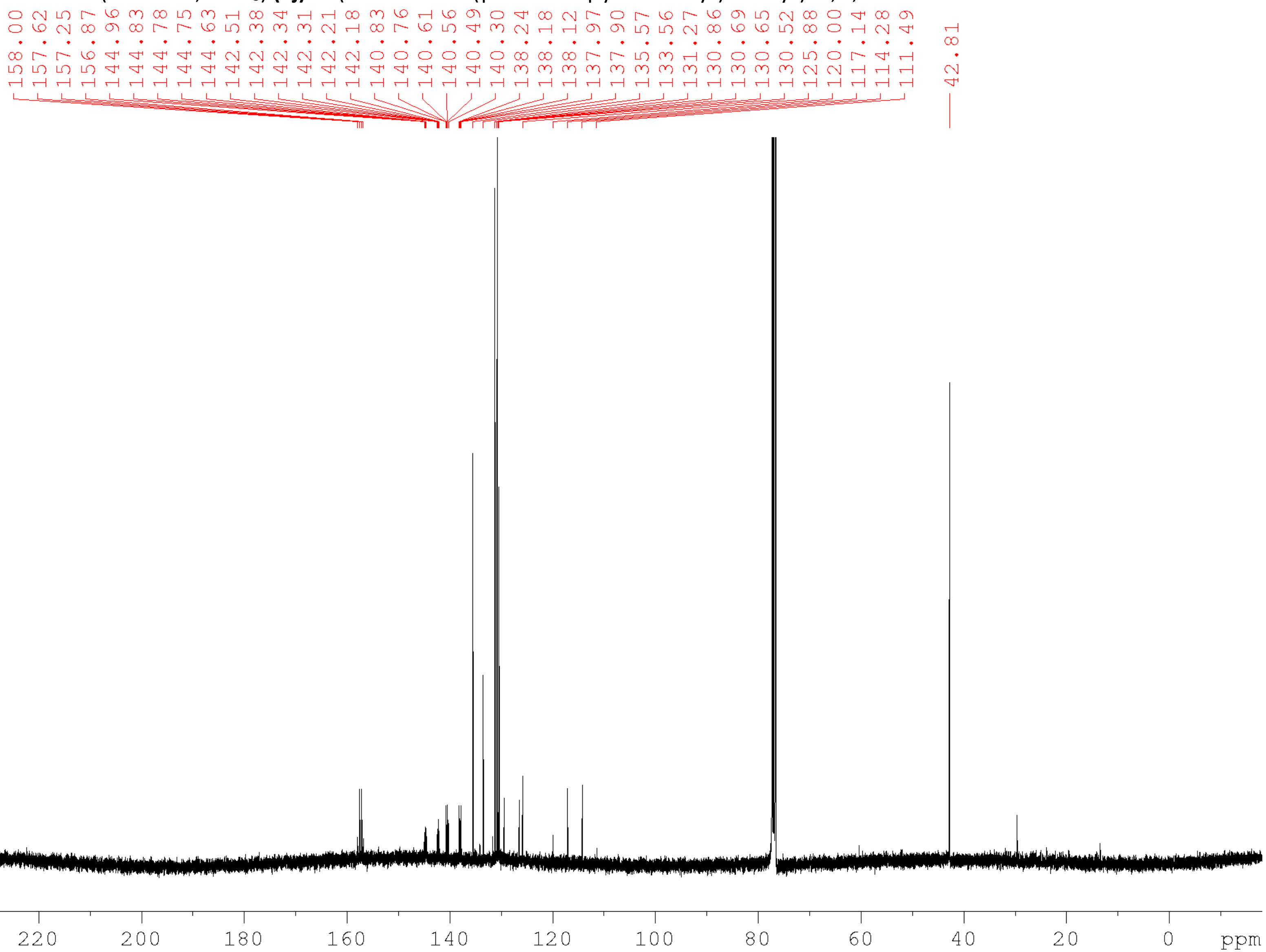

^{19}F NMR (376 MHZ, $CDCl_3$) **(5j)** N-(4-chloro-3-(perfluoropyridin-4-yl)benzyl)-2,2,2-trifluoroacetamide

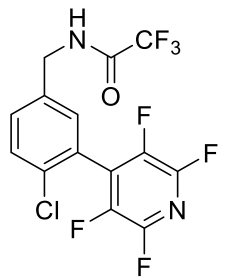

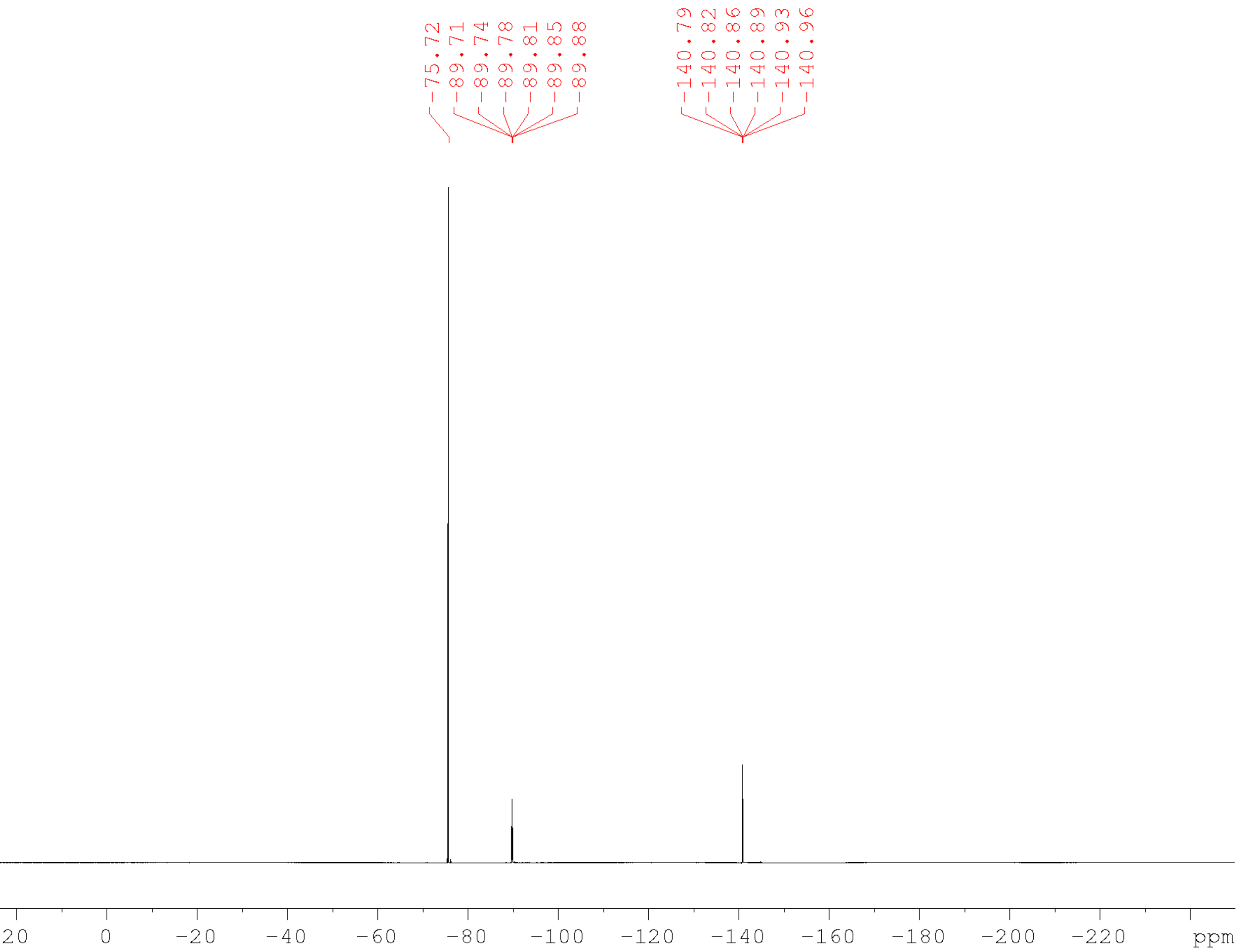

^{1}H NMR (400 MHZ, $CDCl_3$) **(5k)** ethyl methyl (4-chloro-3-(perfluoropyridin-4-yl)benzyl)phosphonate

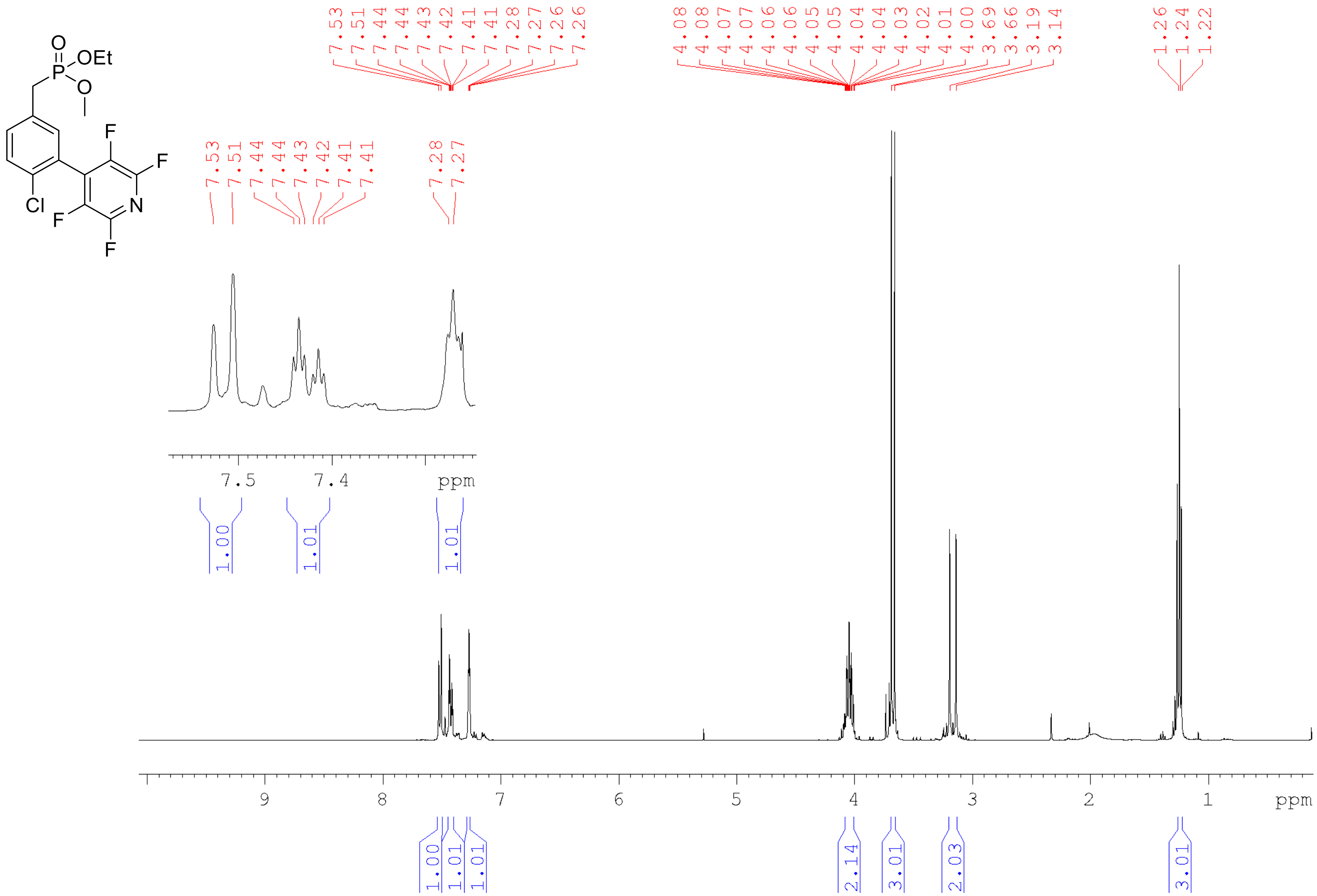

^{13}**C NMR** (101 MHZ, $CDCl_3$) **(5k)** ethyl methyl (4-chloro-3-(perfluoropyridin-4-yl)benzyl)phosphonate

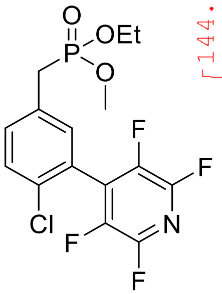

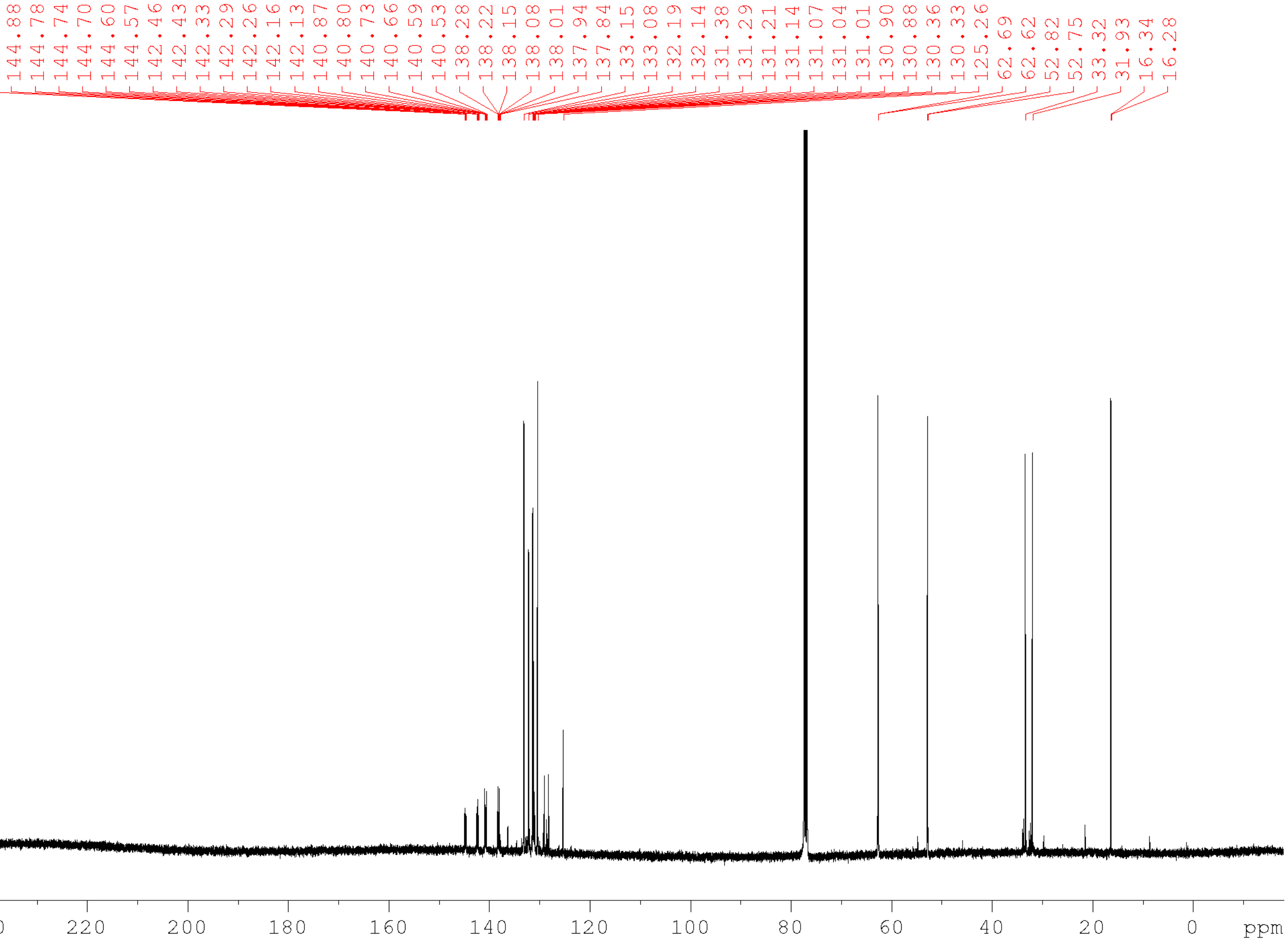

^{19}F NMR (376 MHZ, $CDCl_3$) **(5k)** ethyl methyl (4-chloro-3-(perfluoropyridin-4-yl)benzyl)phosphonate

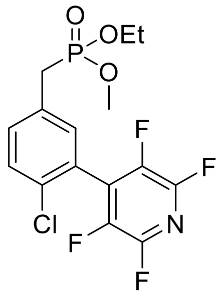

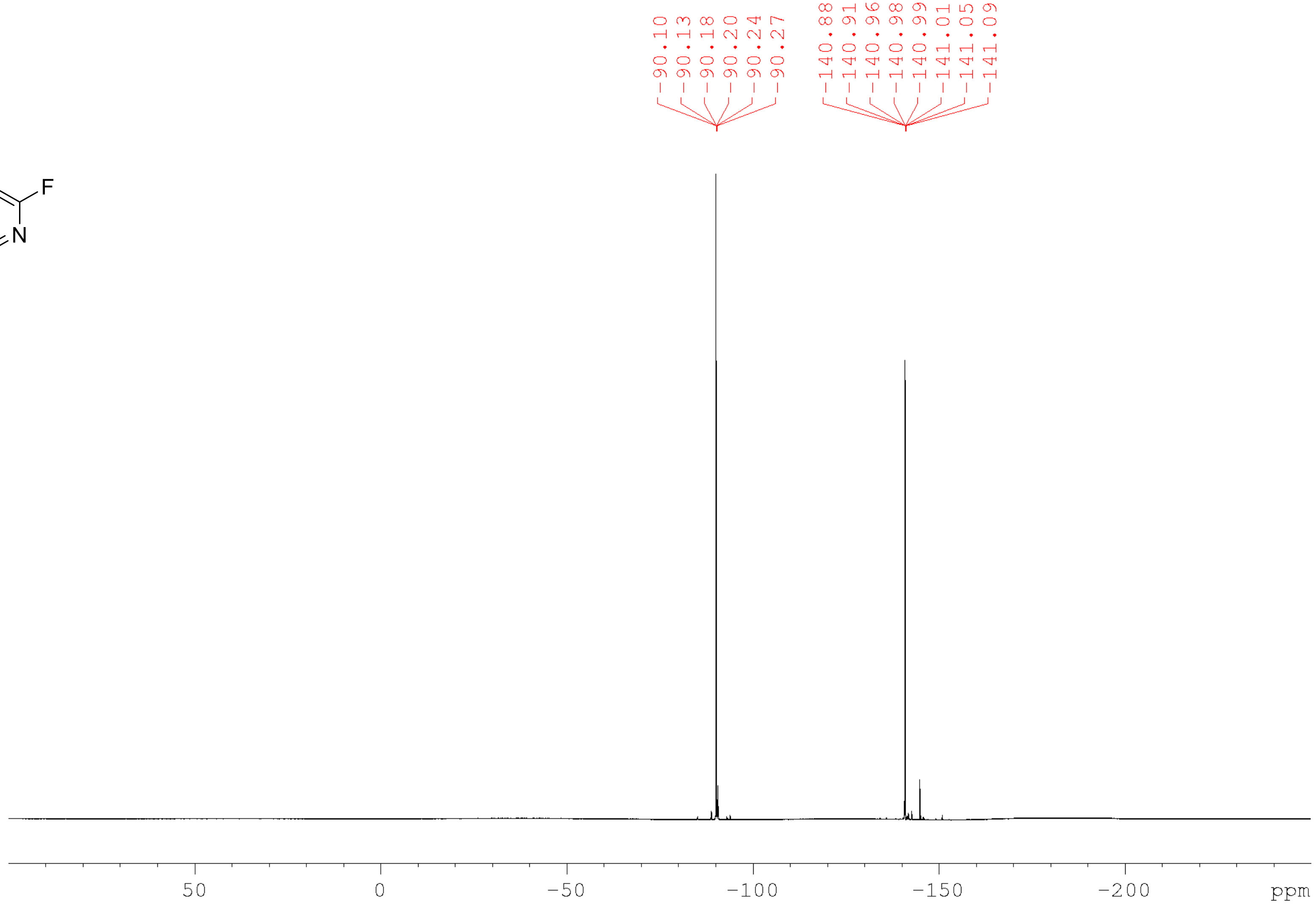

^{31}P NMR (376 MHZ, $CDCl_3$) **(5k)** ethyl methyl (4-chloro-3-(perfluoropyridin-4-yl)benzyl)phosphonate

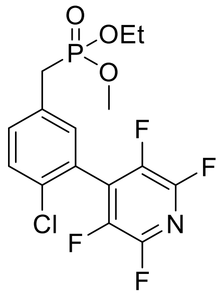

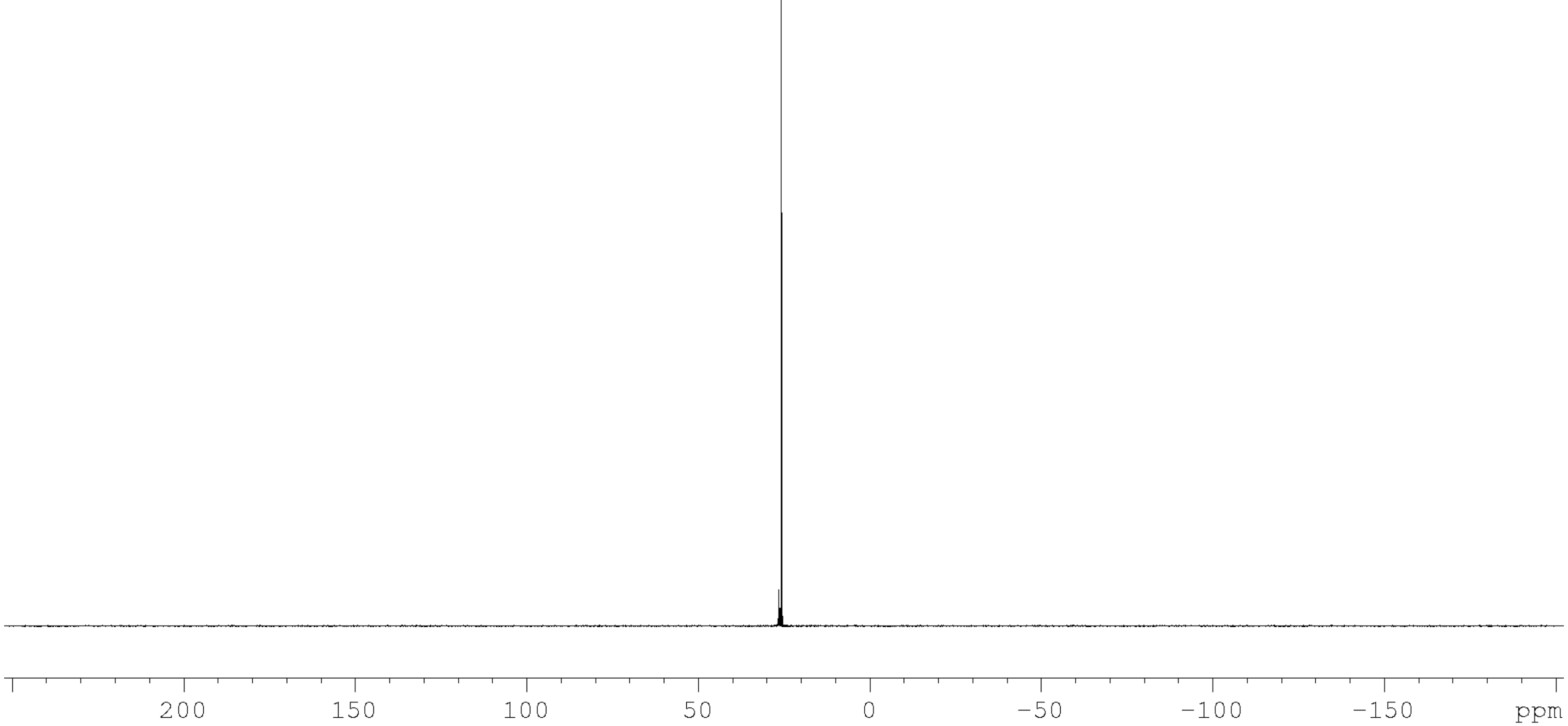

^{1}H NMR (400 MHZ, $CDCl_3$) **(5l)** methyl 4-chloro-3-(perfluoropyridin-4-yl)benzoate

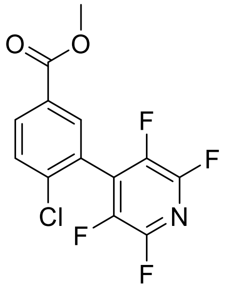

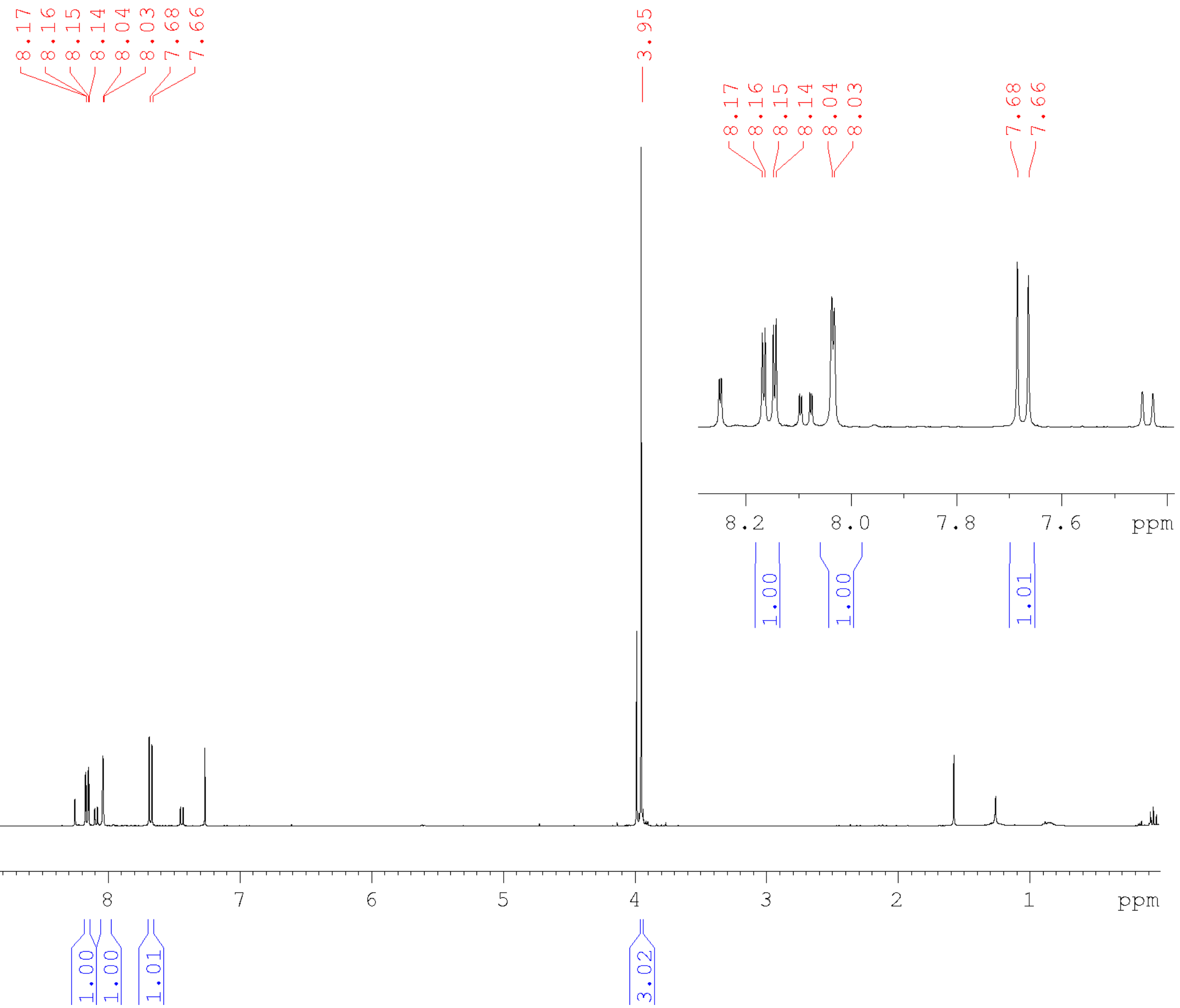

^{13}C NMR (101 MHZ, $CDCl_3$) **(5l)** methyl 4-chloro-3-(perfluoropyridin-4-yl)benzoate

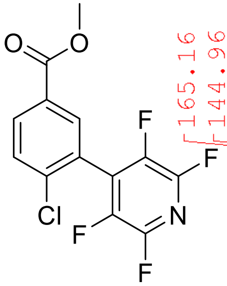

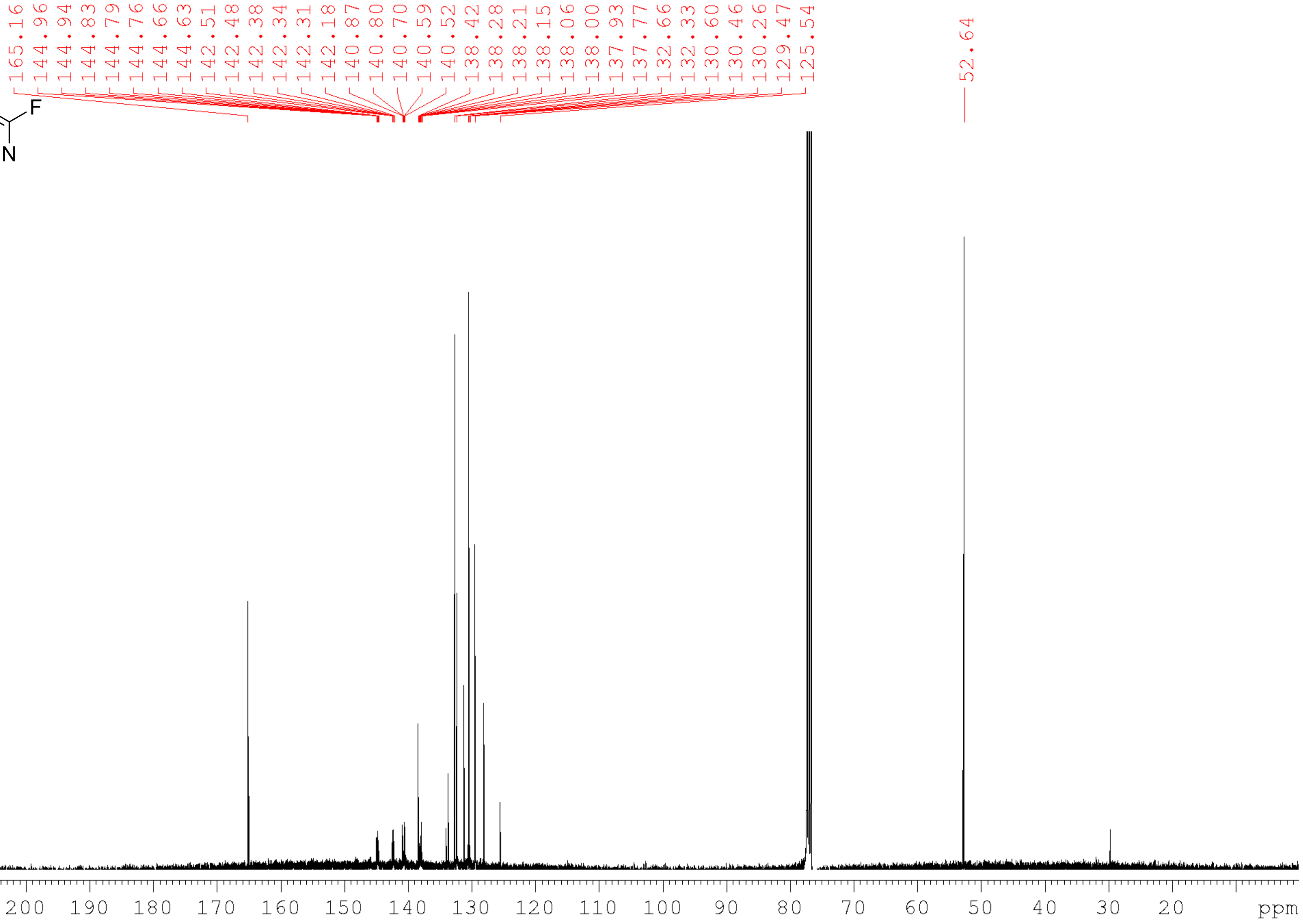

^{19}F NMR (376 MHZ, $CDCl_3$) **(5l)** methyl 4-chloro-3-(perfluoropyridin-4-yl)benzoate

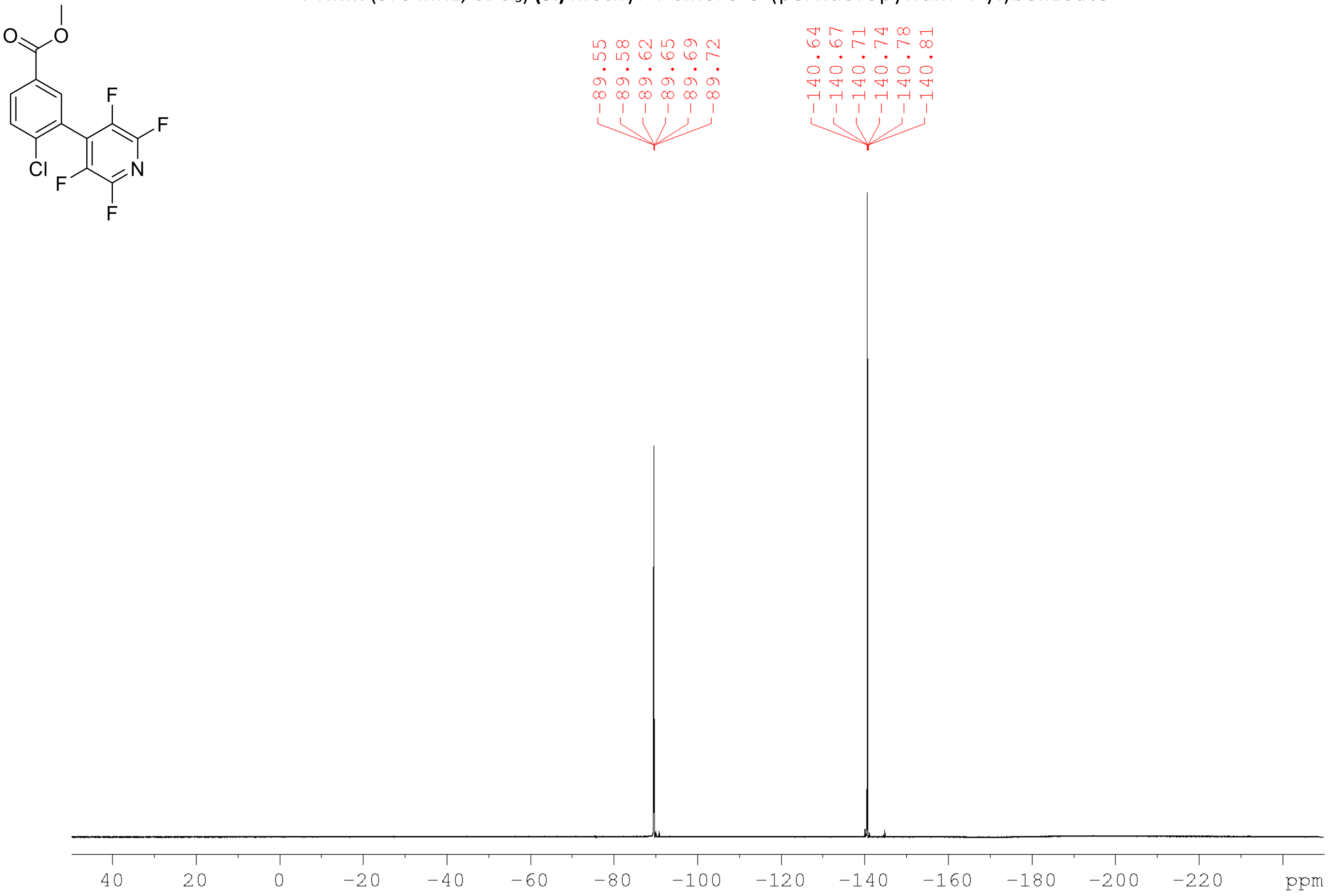

^{1}H NMR (400 MHZ, $CDCl_3$) **(6l)** methyl 2-(3-chloro-4-(perfluoropyridin-4-yl)phenyl)acetate

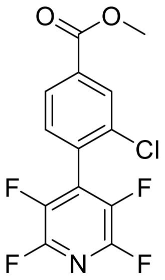

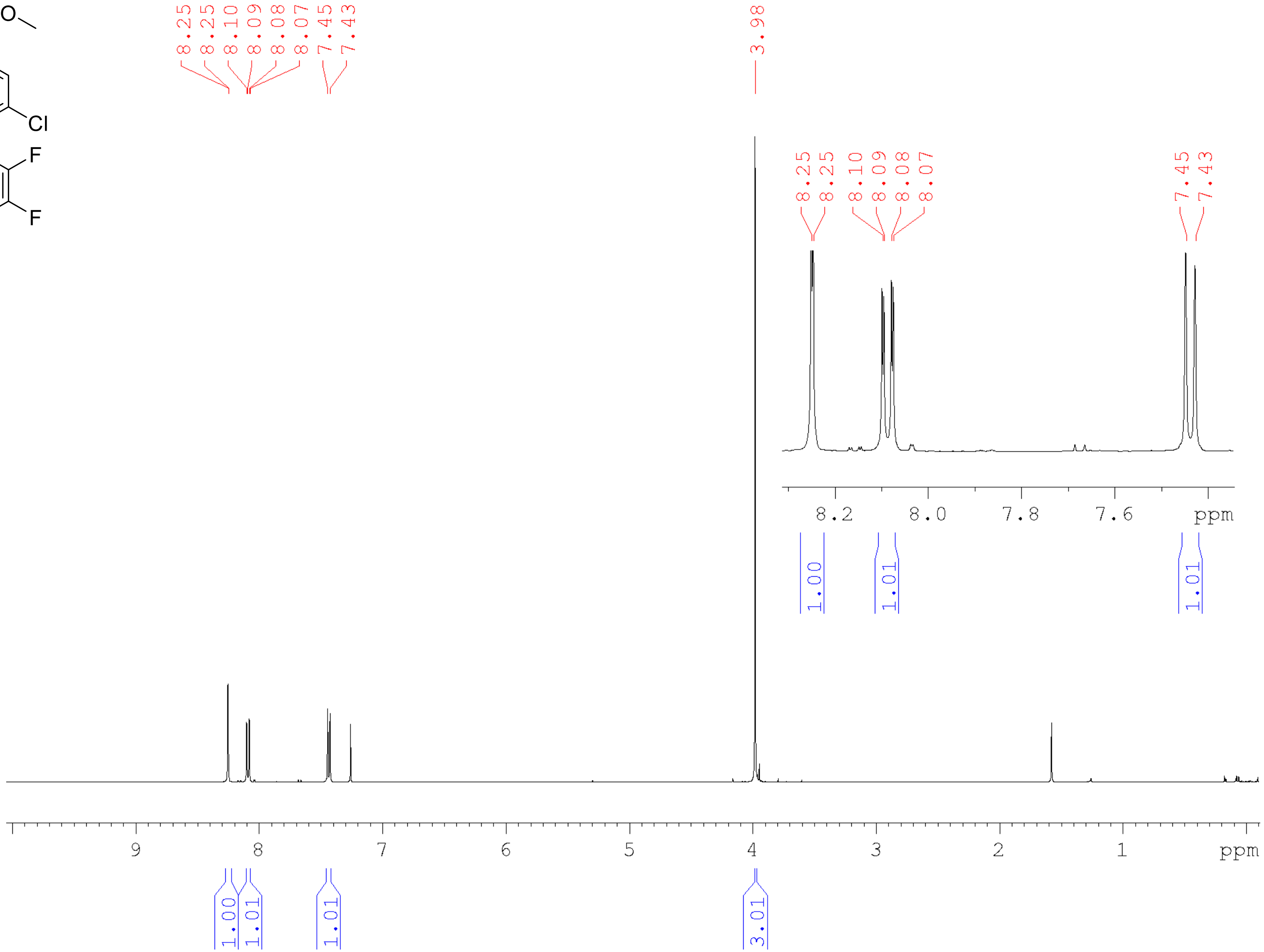

^{13}**C NMR** (101 MHZ, $CDCl_3$) **(6l)** methyl 2-(3-chloro-4-(perfluoropyridin-4-yl)phenyl)acetate

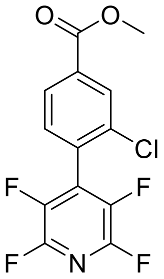

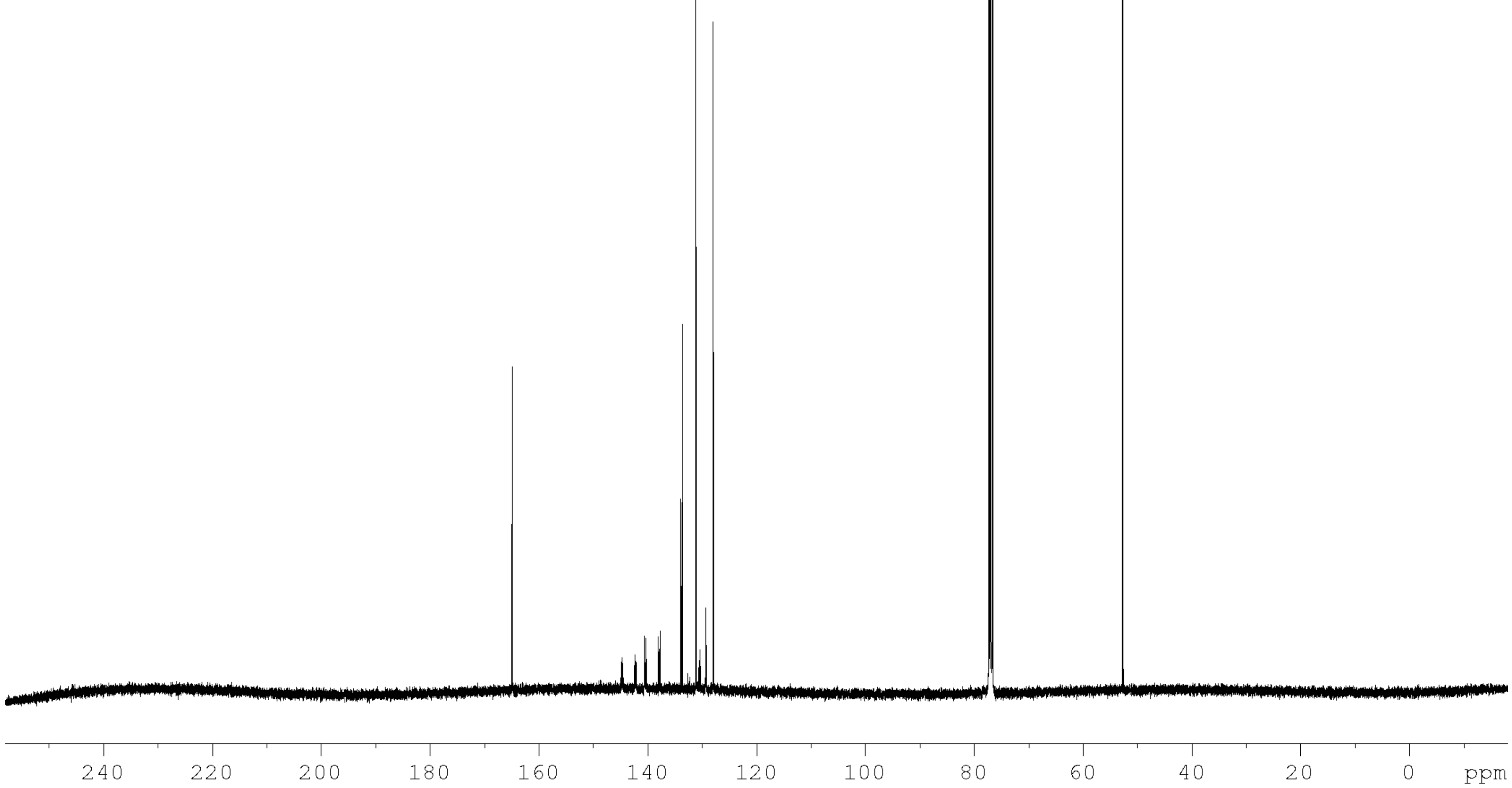

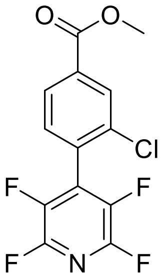

^{19}F NMR (376 MHZ, $CDCl_3$) **(6l)** methyl 2-(3-chloro-4-(perfluoropyridin-4-yl)phenyl)acetate

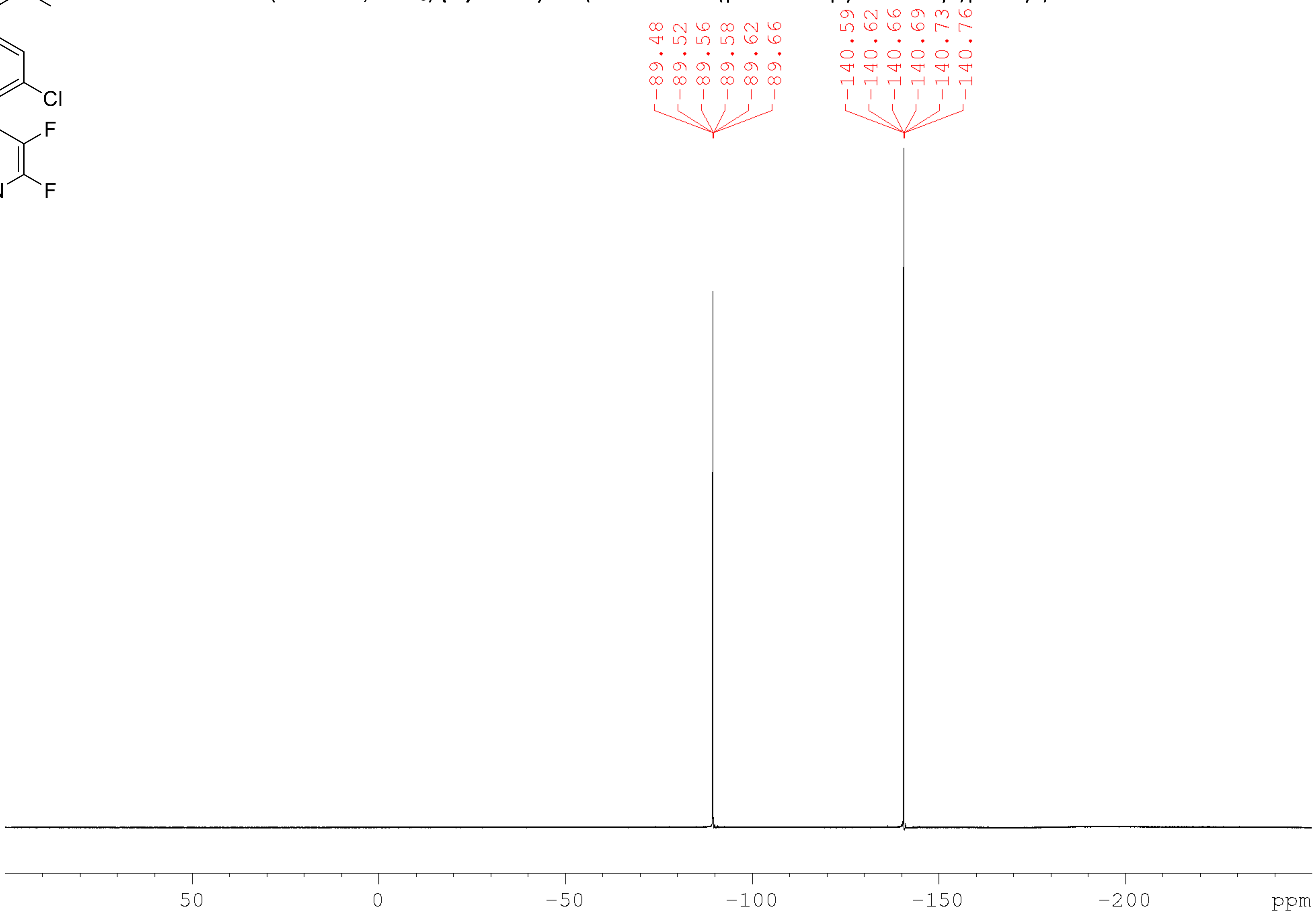

^{1}H NMR (400 MHZ, CDCl3) **(5m)** methyl 2-(4-chloro-3-(perfluoropyridin-4-yl)phenyl)acetate

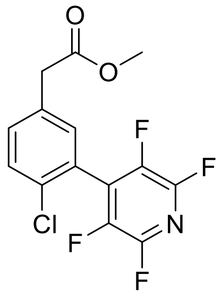

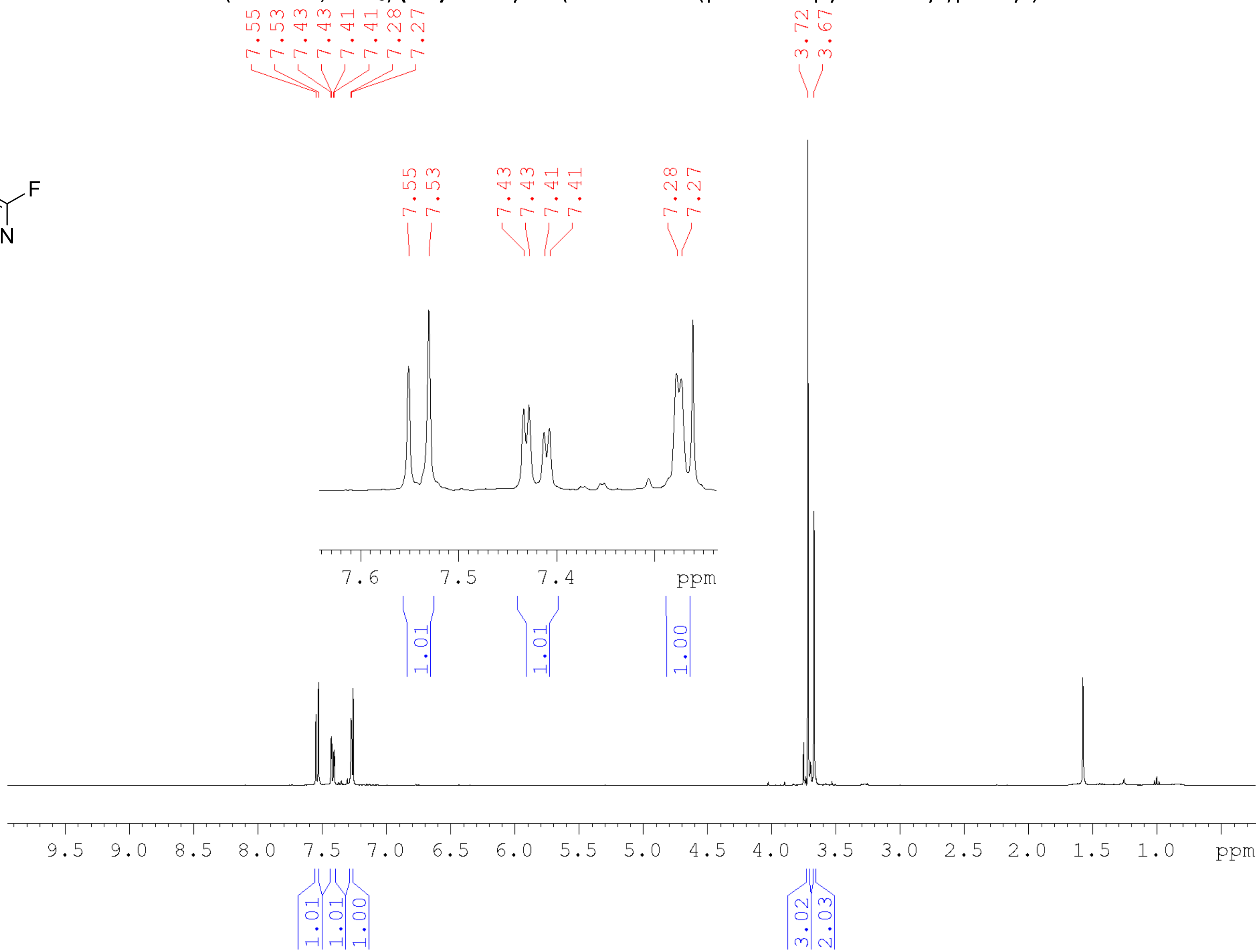

^{13}C NMR (101 MHZ, $CDCl_3$) **(5m)** methyl 2-(4-chloro-3-(perfluoropyridin-4-yl)phenyl)acetate

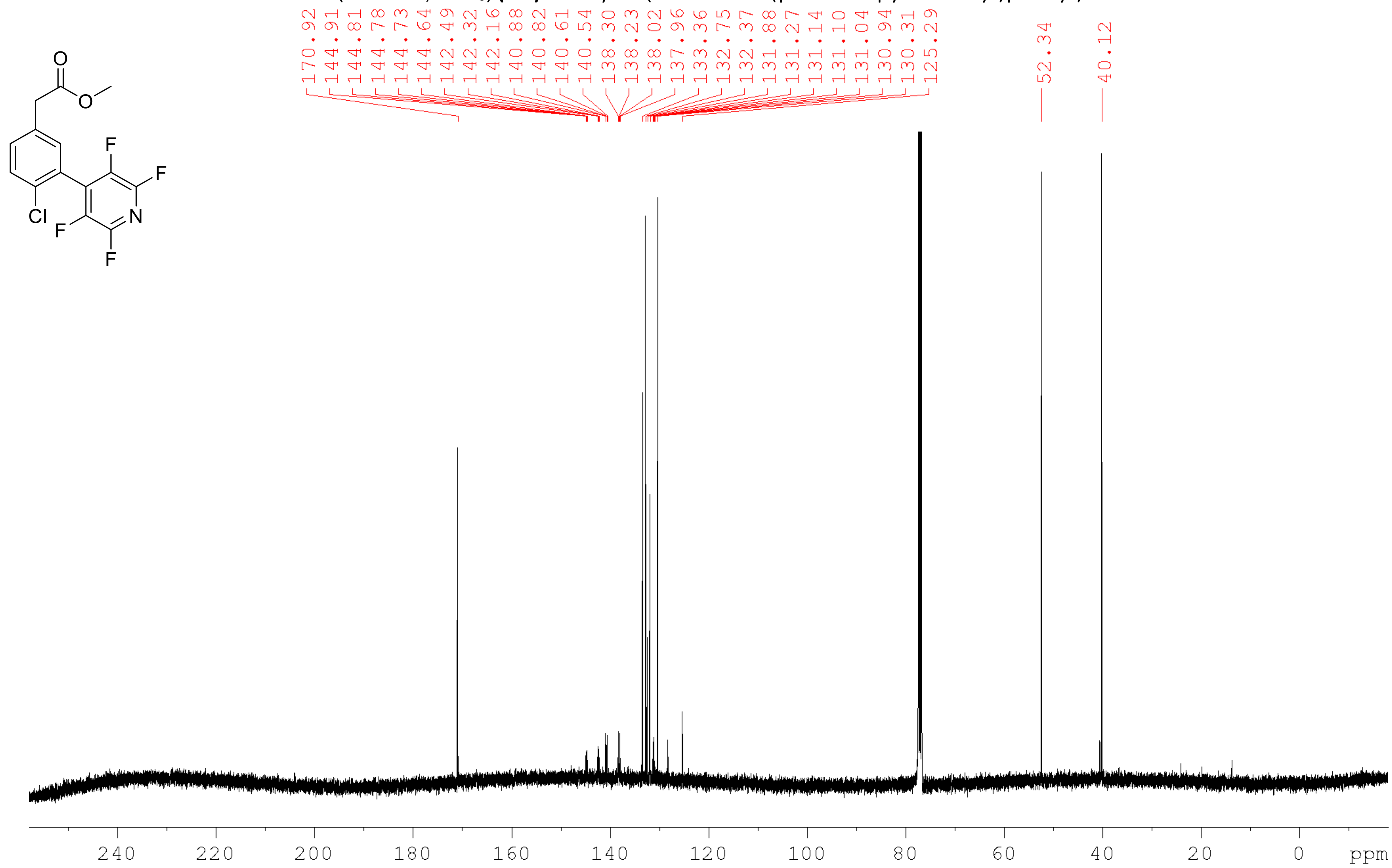

^{19}F NMR (376 MHZ, $CDCl_3$) **(5m)** methyl 2-(4-chloro-3-(perfluoropyridin-4-yl)phenyl)acetate

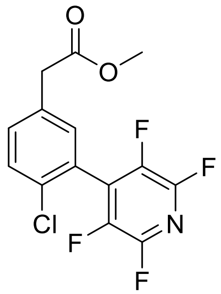

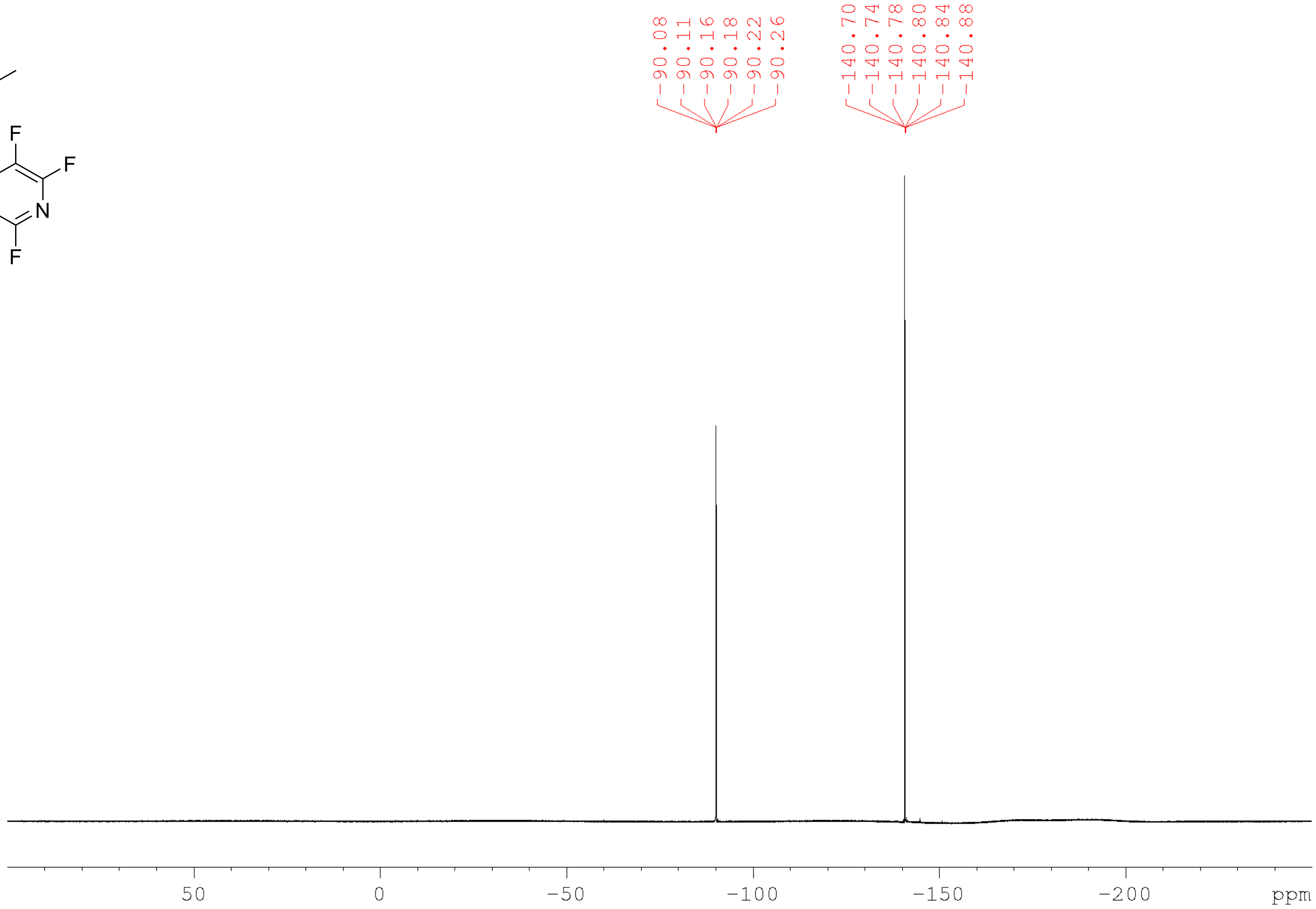

^{1}H NMR (400 MHZ, $CDCl_3$) **(6m)** methyl 2-(3-chloro-4-(perfluoropyridin-4-yl)phenyl)acetate

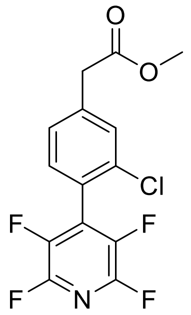

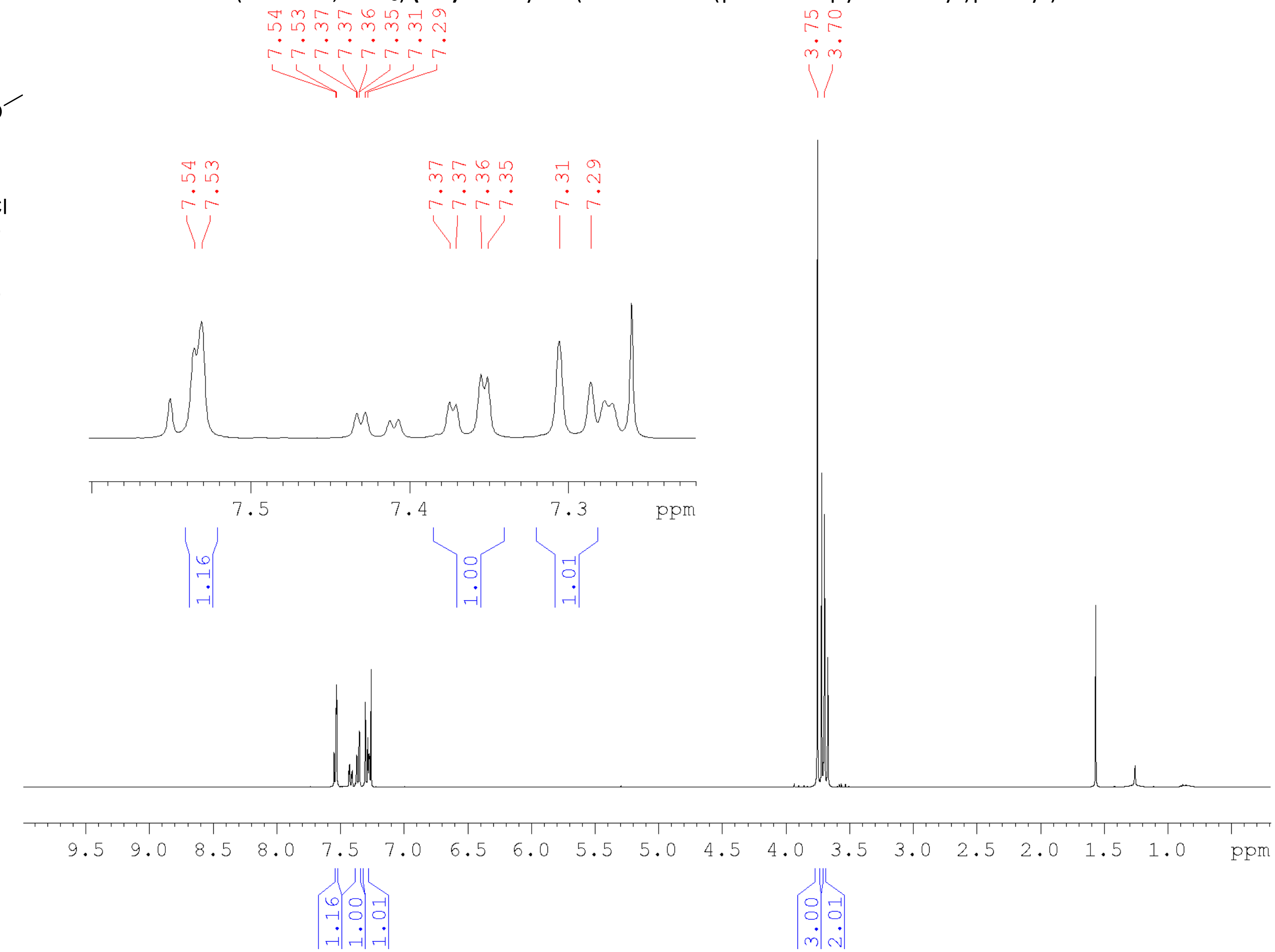

^{13}**C NMR** (101 MHZ, $CDCl_3$) **(6m)** methyl 2-(3-chloro-4-(perfluoropyridin-4-yl)phenyl)acetate

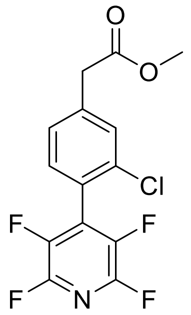

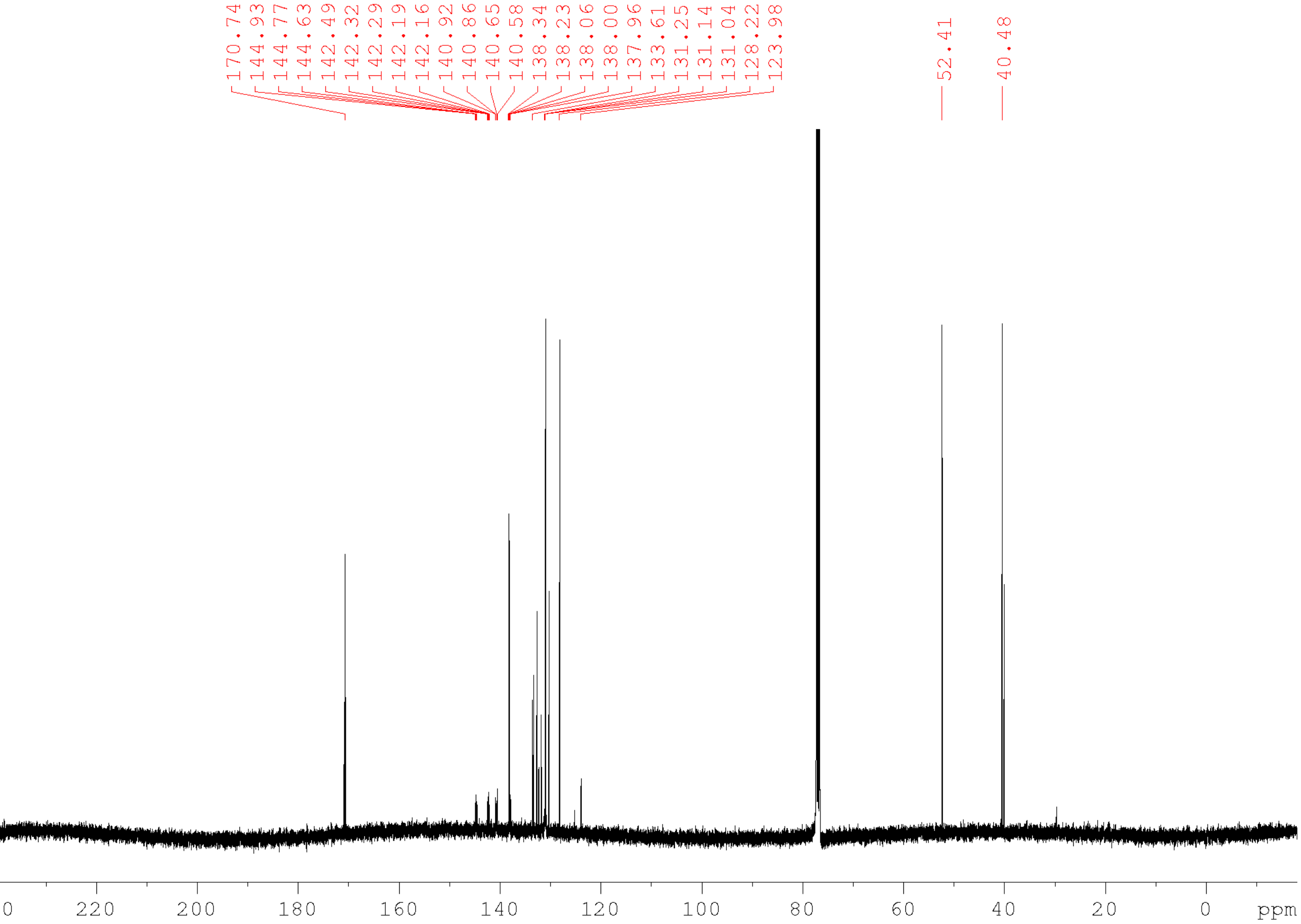

^{19}F NMR (376 MHZ, $CDCl_3$) **(6m)** methyl 2-(3-chloro-4-(perfluoropyridin-4-yl)phenyl)acetate

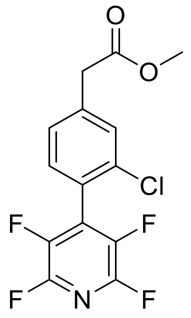

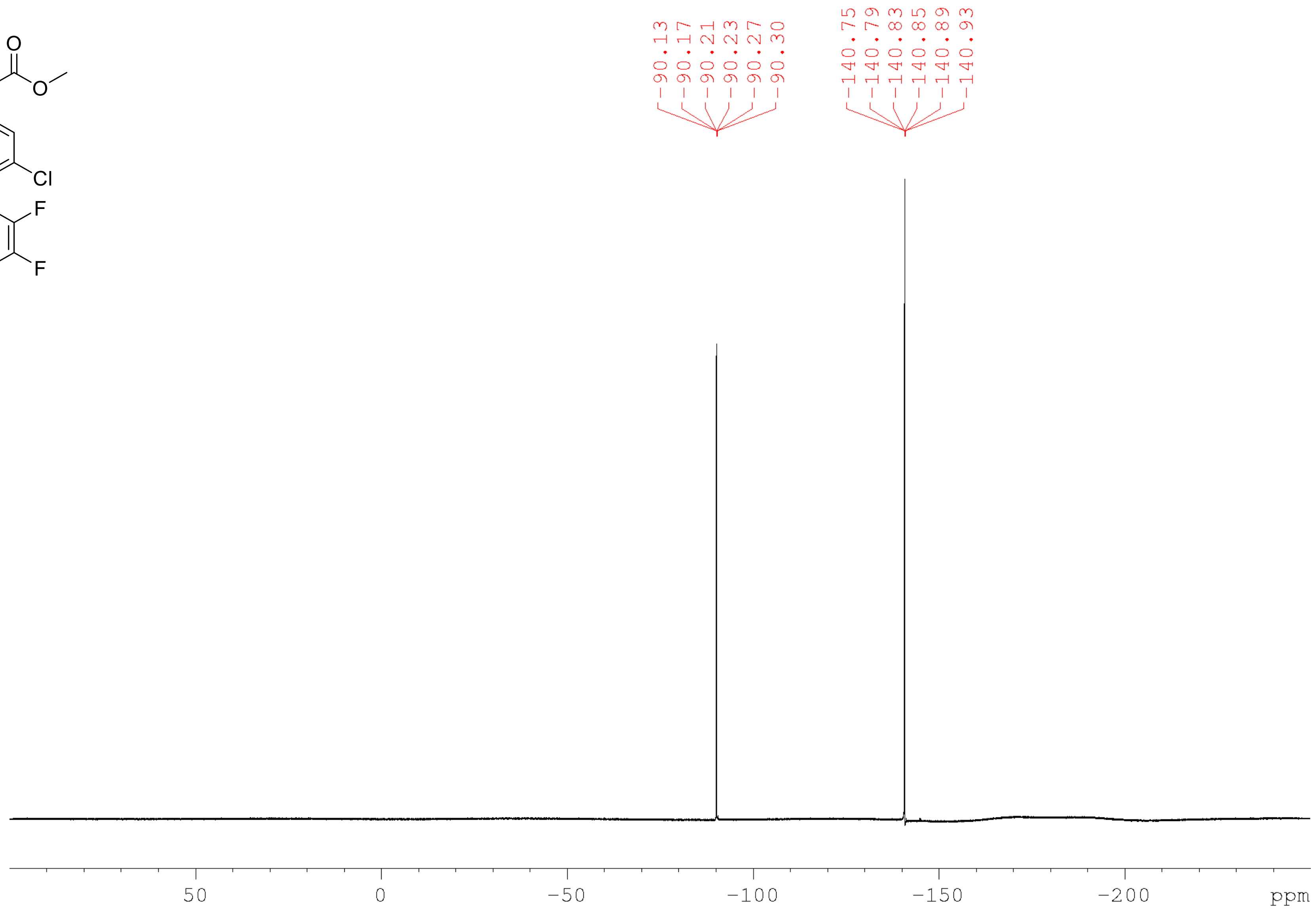

^{1}H NMR (400 MHZ, $CDCl_3$) **(5n)** methyl 3-(4-chloro-3-(perfluoropyridin-4-yl)phenyl)propanoate

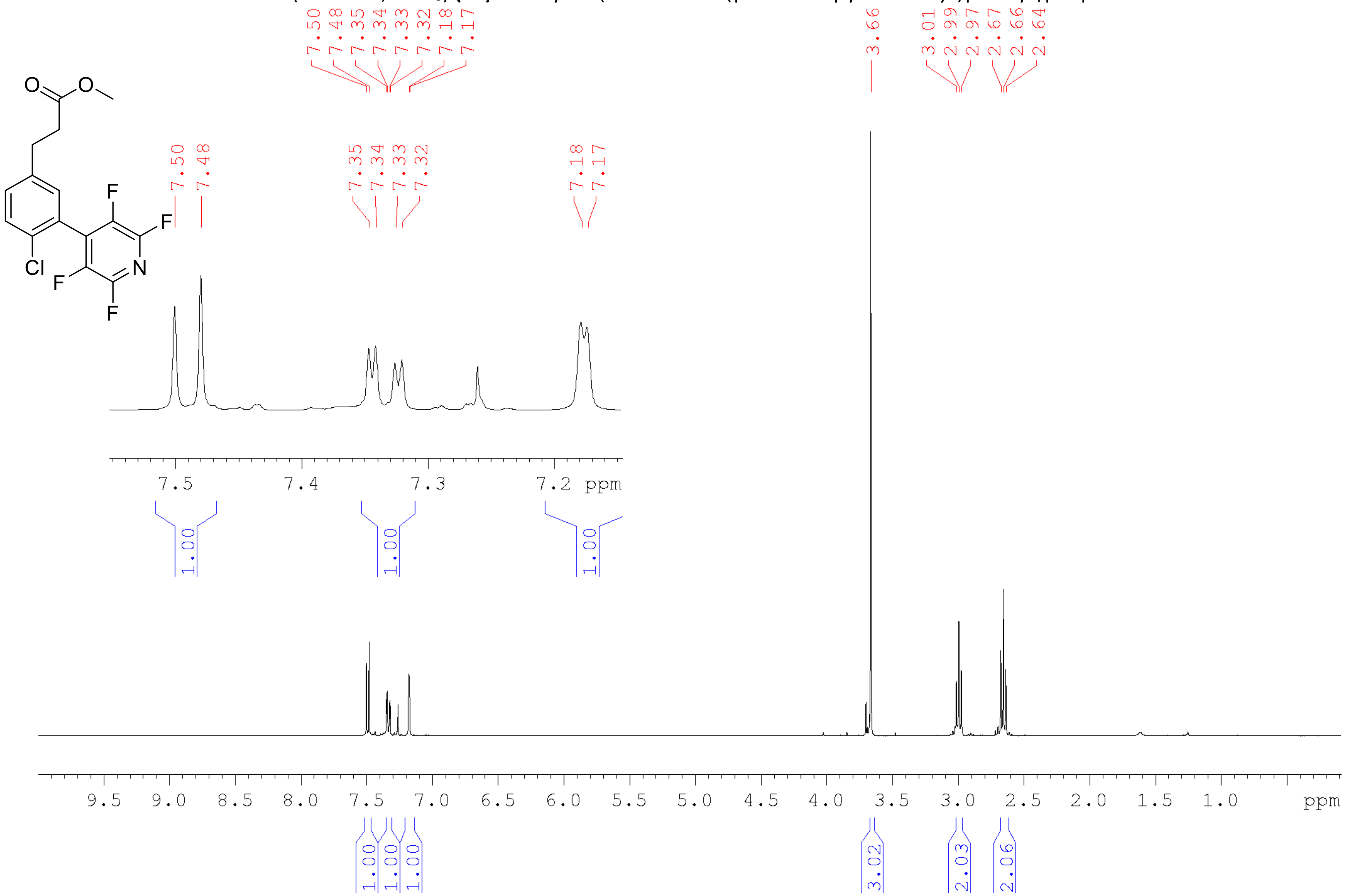

^{13}C NMR (101 MHZ, $CDCl_3$) **(5n)** methyl 3-(4-chloro-3-(perfluoropyridin-4-yl)phenyl)propanoate

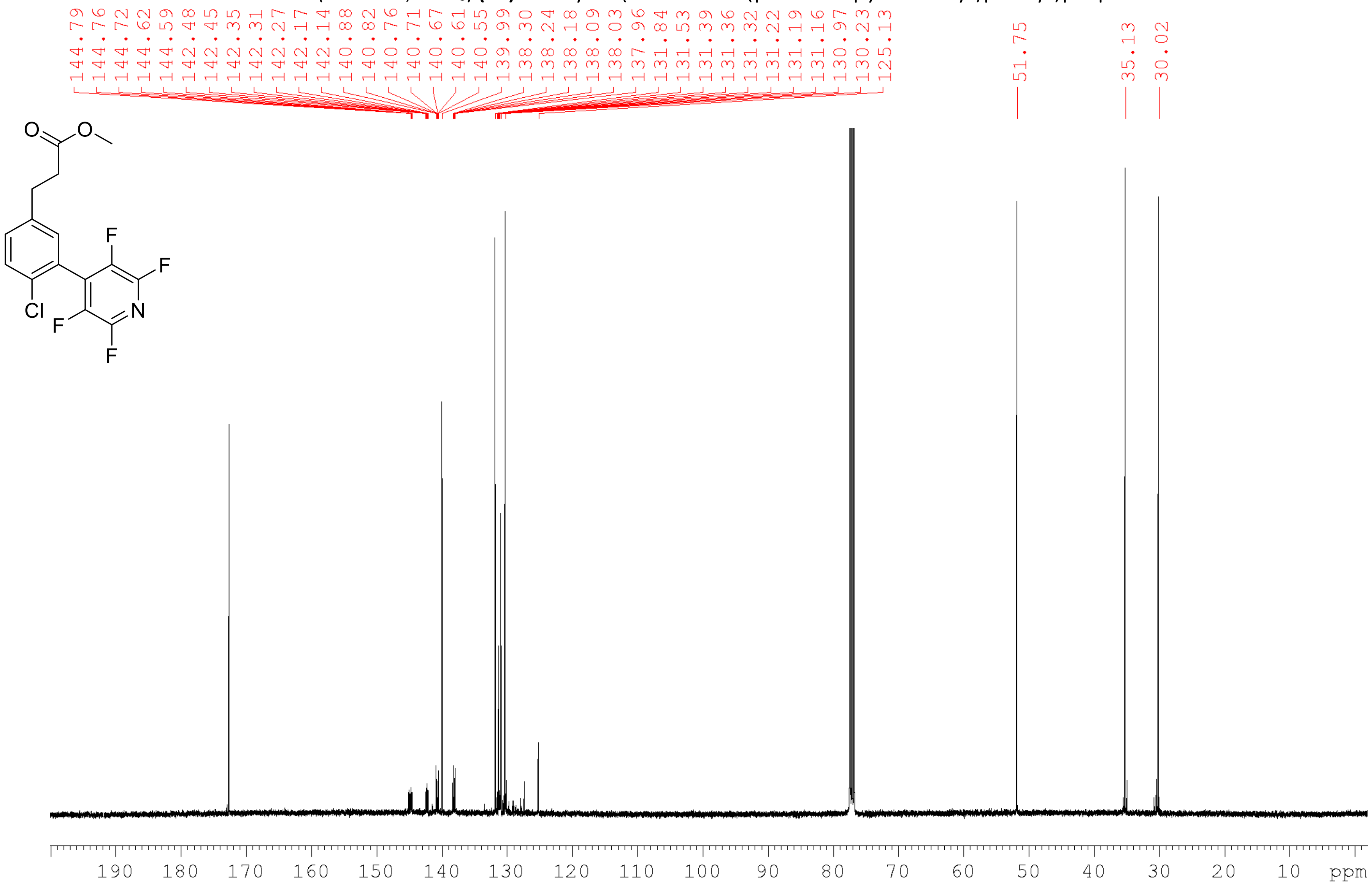

^{19}F NMR (376 MHZ, $CDCl_3$) **(5n)** methyl 3-(4-chloro-3-(perfluoropyridin-4-yl)phenyl)propanoate

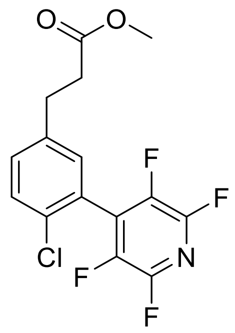

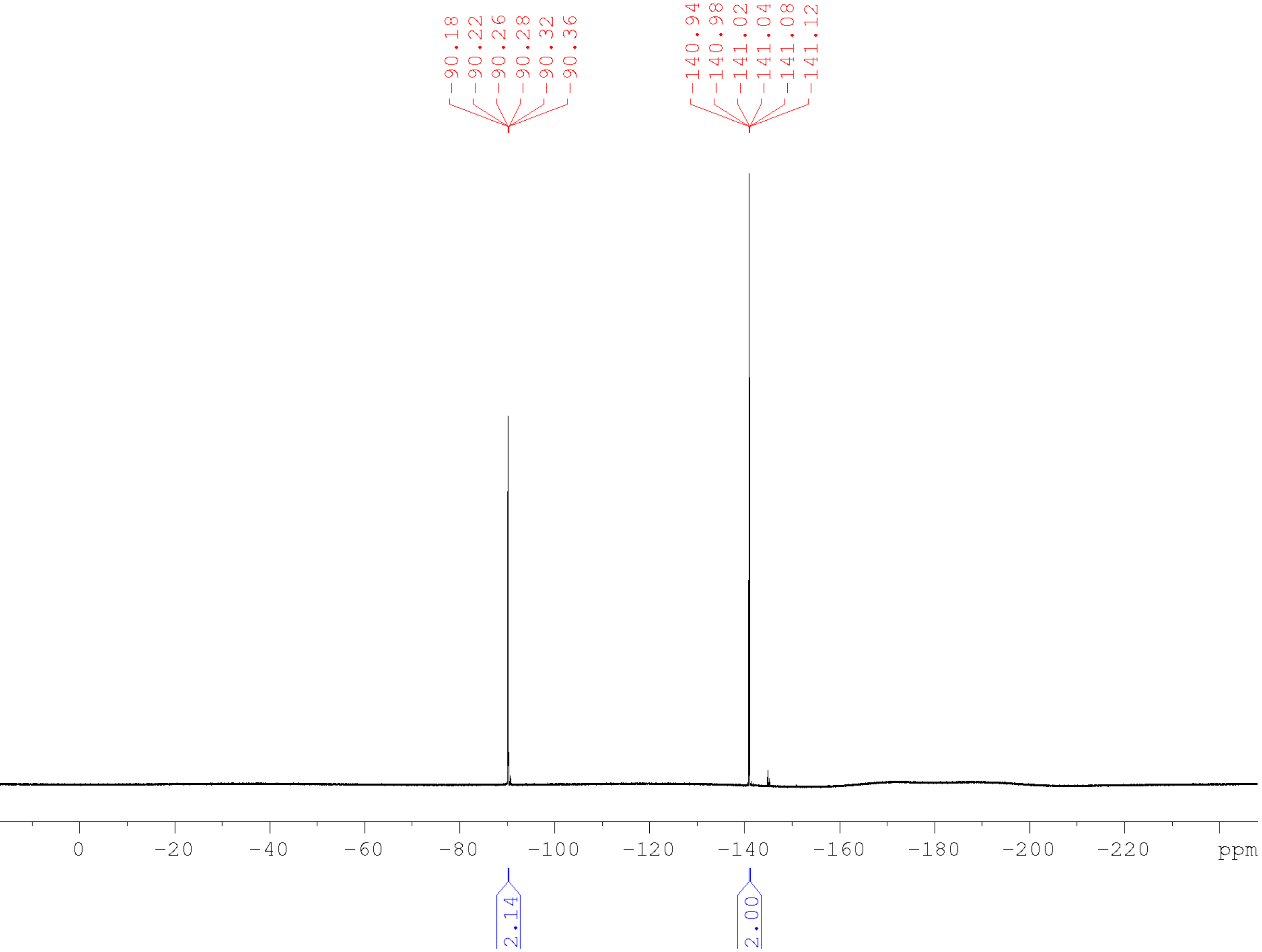

^{1}H NMR (400 MHZ, $CDCl_3$) **(5o)** methyl 4-(4-chloro-3-(perfluoropyridin-4-yl)phenyl)butanoate

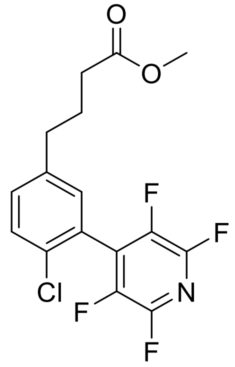

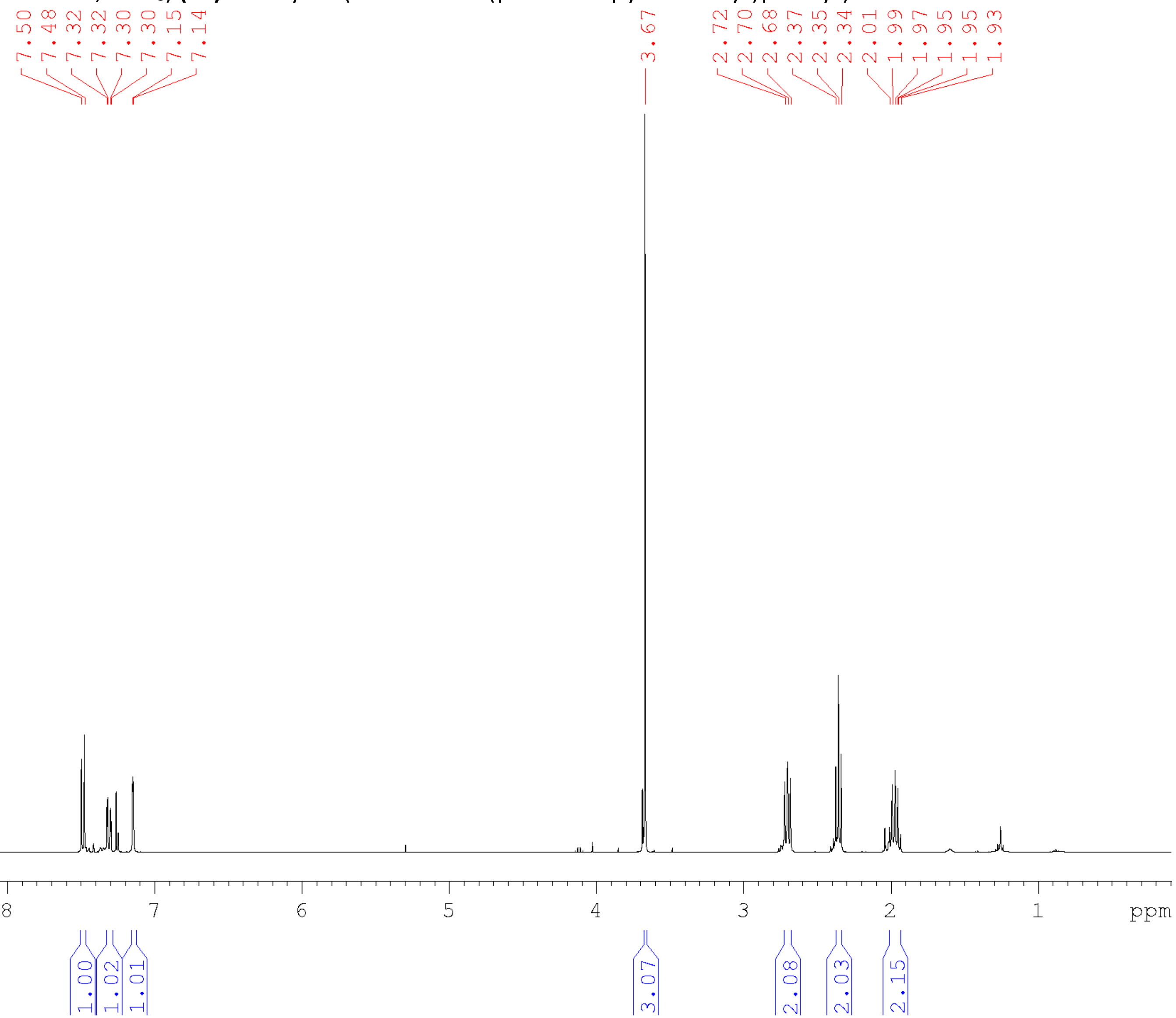

^{13}C NMR (101 MHZ, $CDCl_3$) **(5o)** methyl 4-(4-chloro-3-(perfluoropyridin-4-yl)phenyl)butanoate

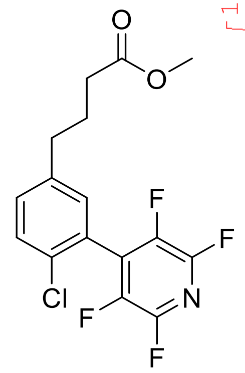

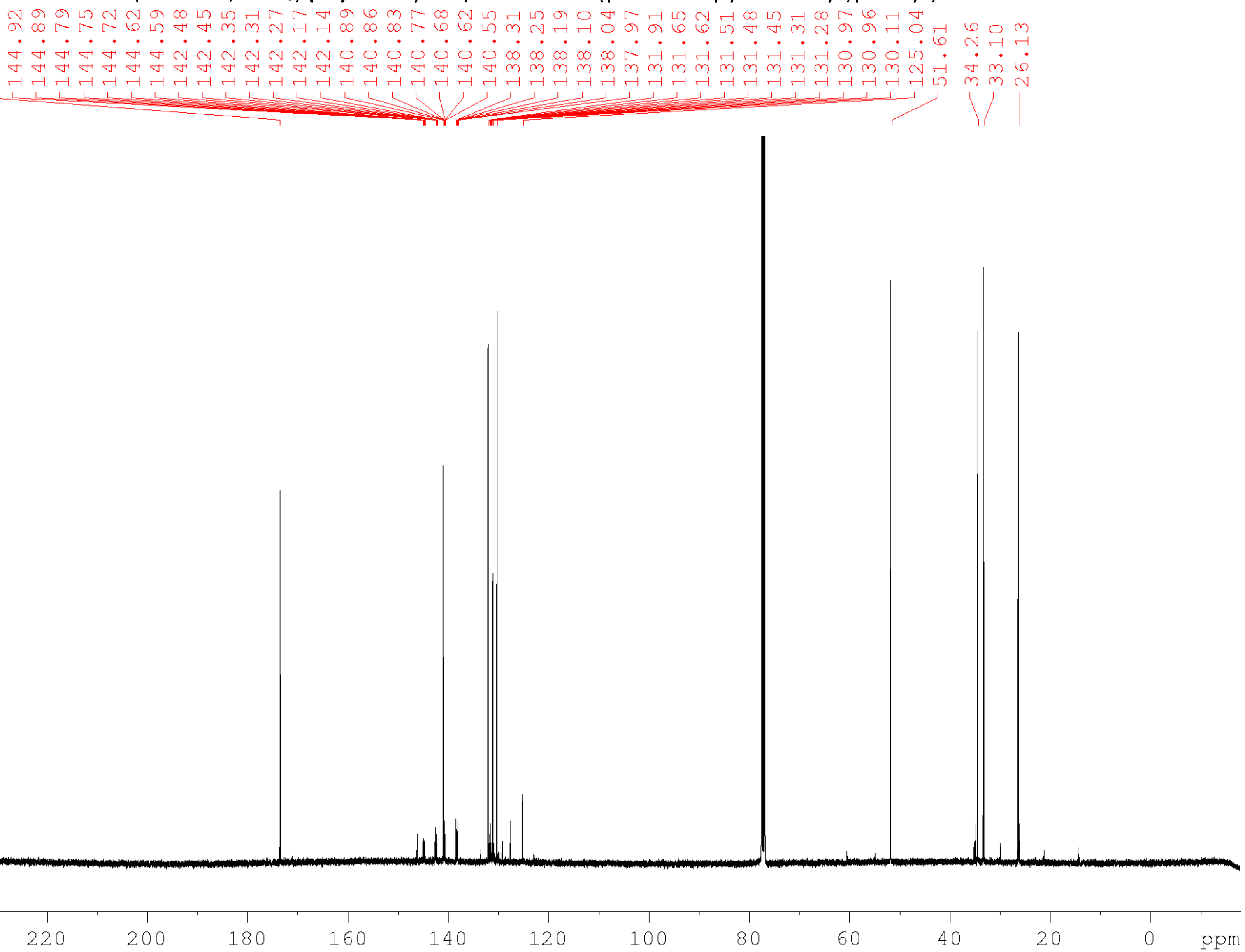

^{19}F NMR (376 MHZ, $CDCl_3$) **(5o)** methyl 4-(4-chloro-3-(perfluoropyridin-4-yl)phenyl)butanoate

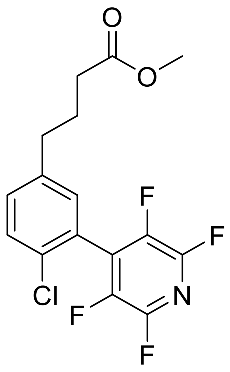

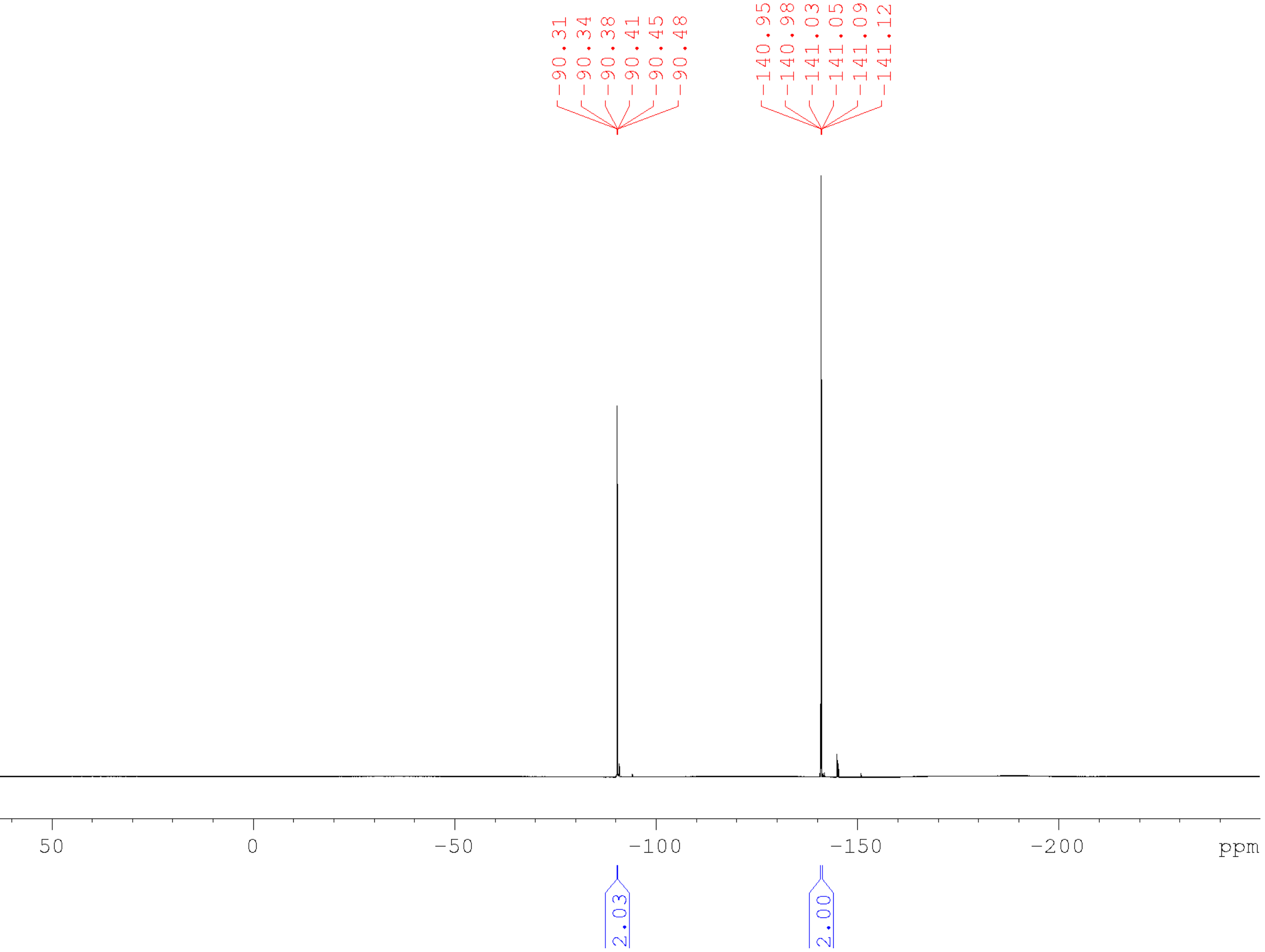

^{1}H NMR (400 MHZ, $CDCl_3$) **(5p)** methyl (E)-4-(4-chloro-3-(perfluoropyridin-4-yl)phenyl)but-3-enoate

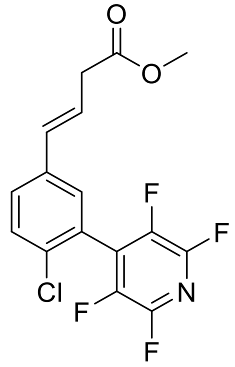

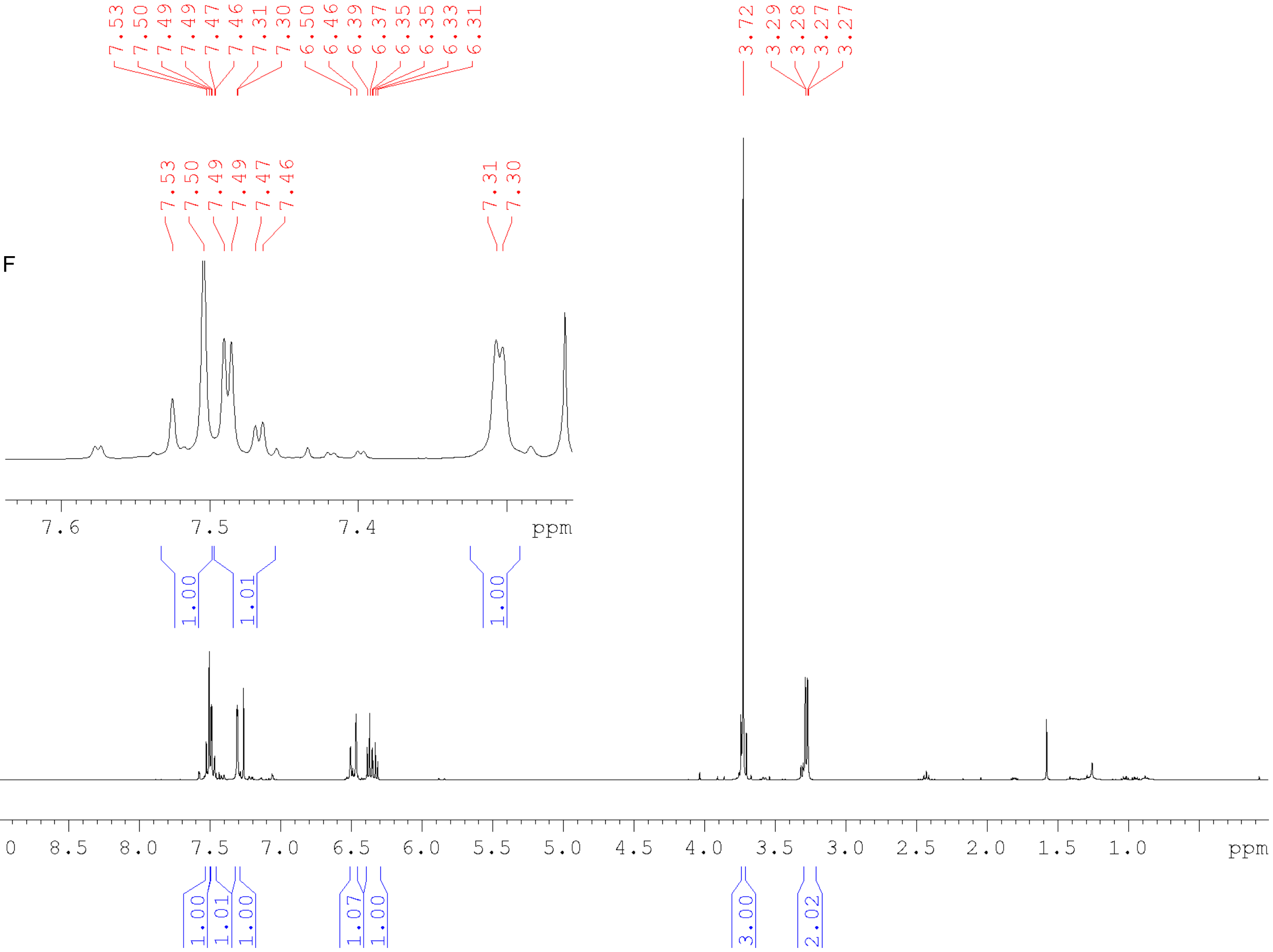

^{13}C NMR (101 MHZ, $CDCl_3$) **(5p)** methyl (E)-4-(4-chloro-3-(perfluoropyridin-4-yl)phenyl)but-3-enoate

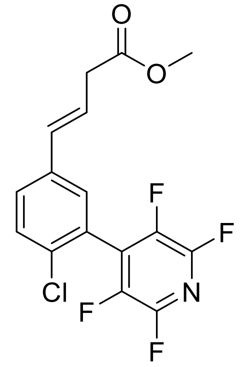

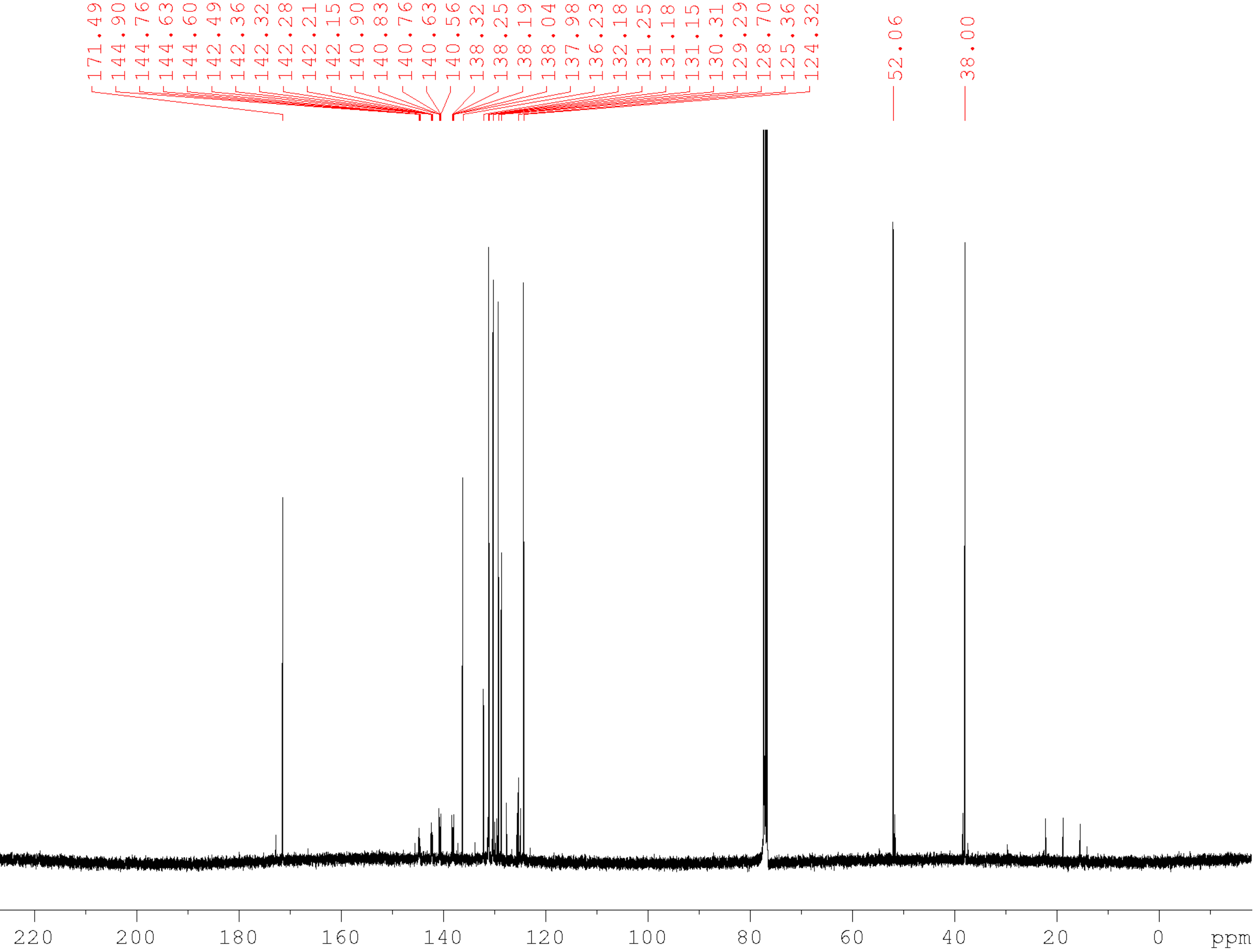

^{19}**F NMR** (376 MHZ, $CDCl_3$) **(5p)** methyl (E)-4-(4-chloro-3-(perfluoropyridin-4-yl)phenyl)but-3-enoate

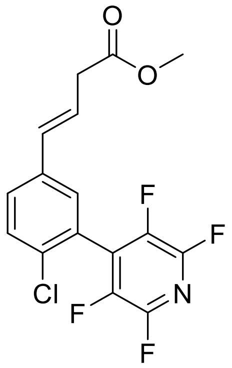

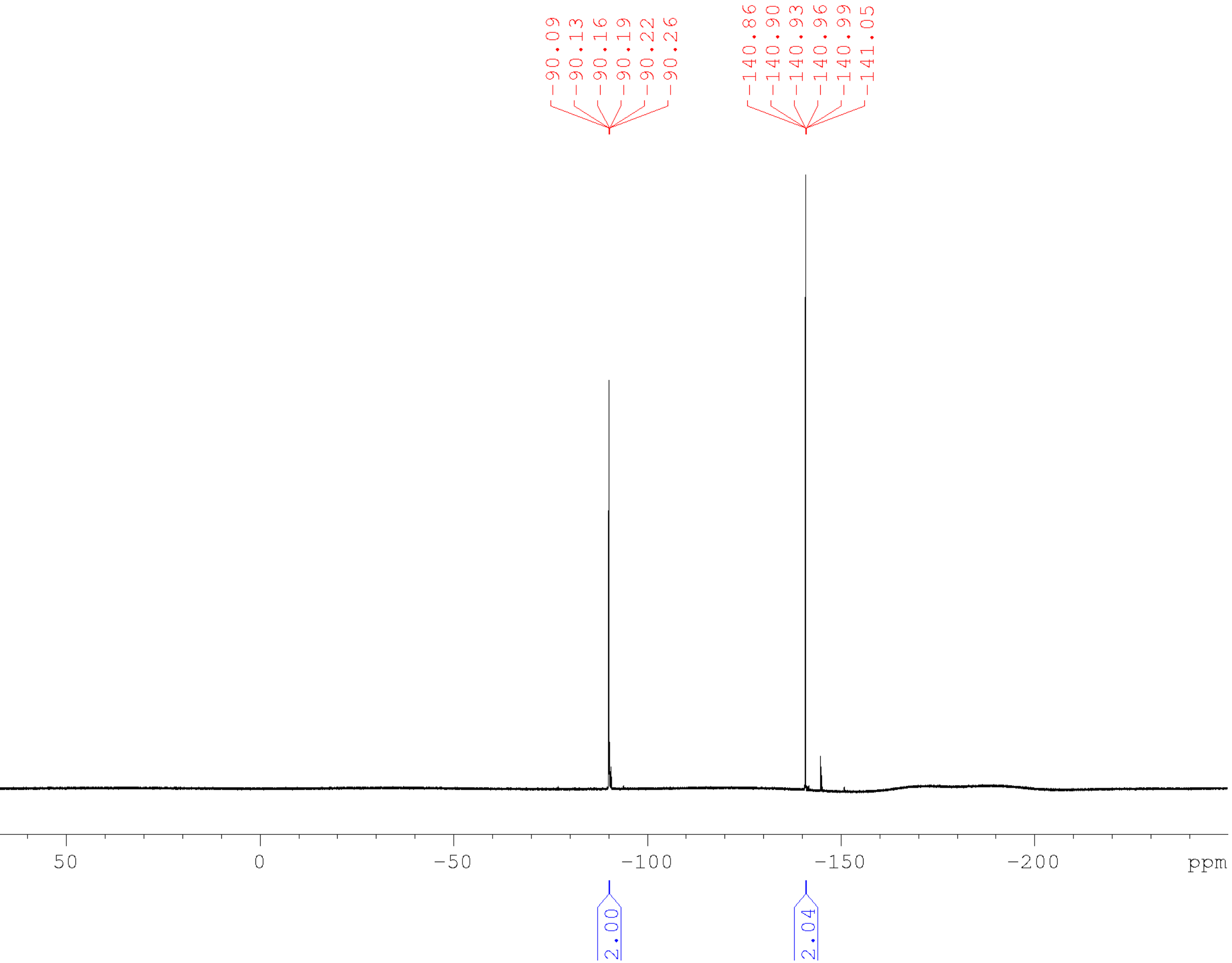

N-Oxide coupling

^{1}H NMR (400 MHZ, $CDCl_3$) **(7)** 2-(2-chloro-5-(((trifluoromethyl)sulfonamido)methyl)phenyl)-4,5-dimethylthiazole 3-oxide

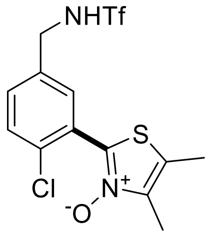

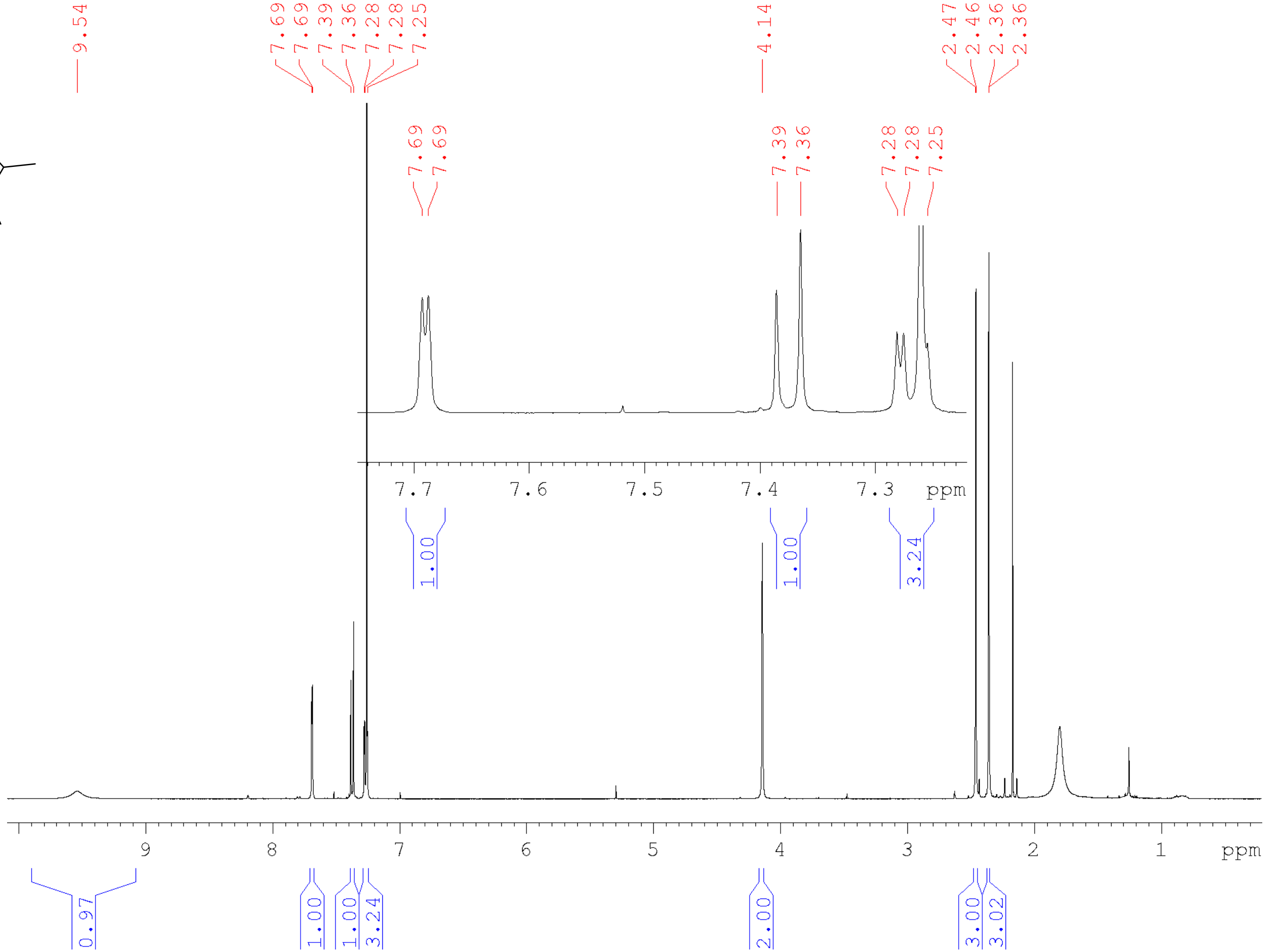

^{13}C NMR (101 MHZ, $CDCl_3$) **(7)** 2-(2-chloro-5-(((trifluoromethyl)sulfonamido)methyl)phenyl)-4,5-dimethylthiazole 3-oxide

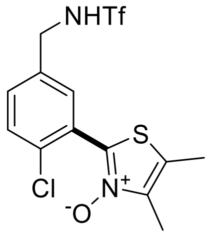

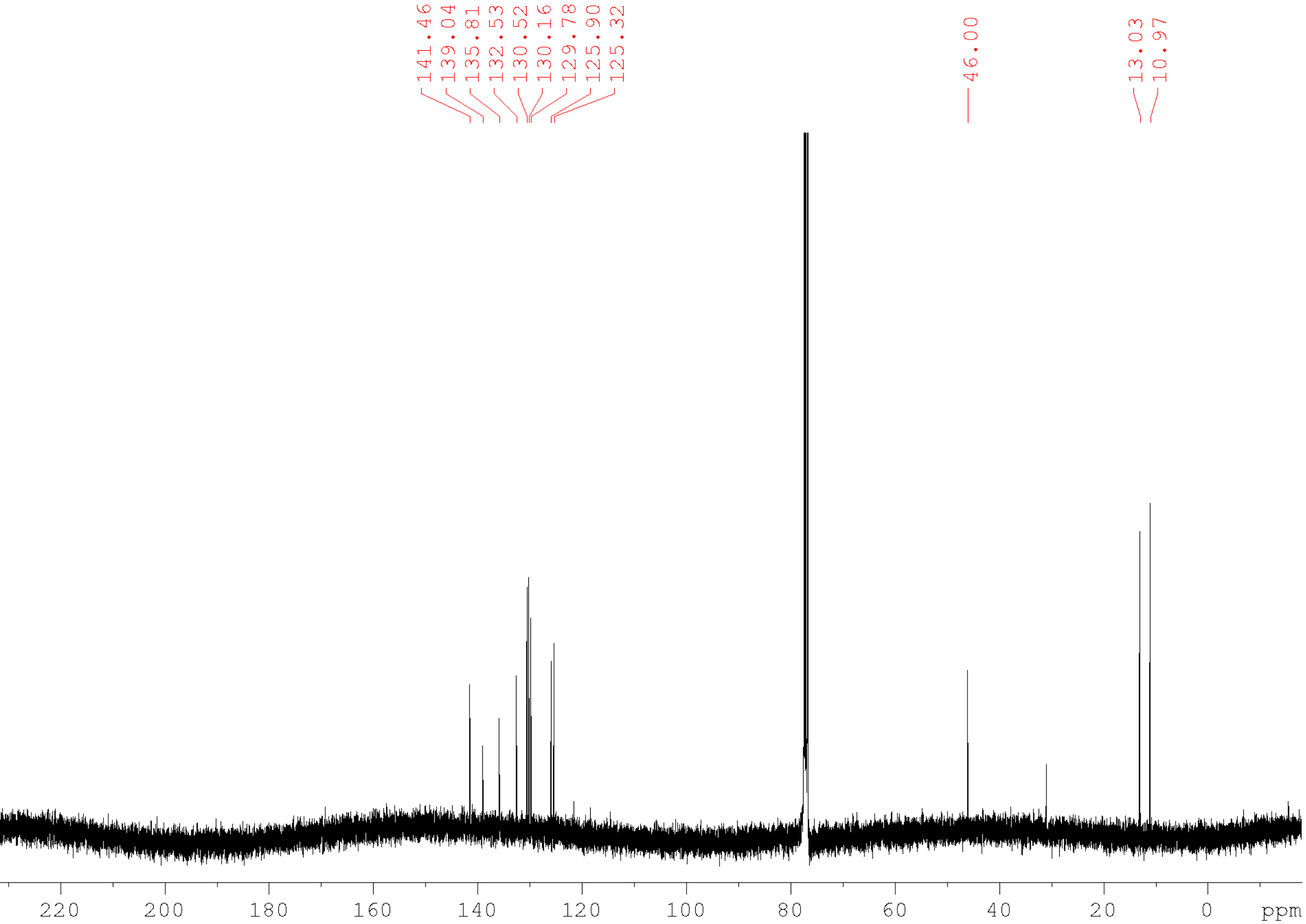

^{19}**F NMR** (376 MHZ, $CDCl_3$) **(7)** 2-(2-chloro-5-(((trifluoromethyl)sulfonamido)methyl)phenyl)-4,5-dimethylthiazole 3-oxide

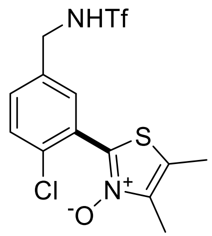

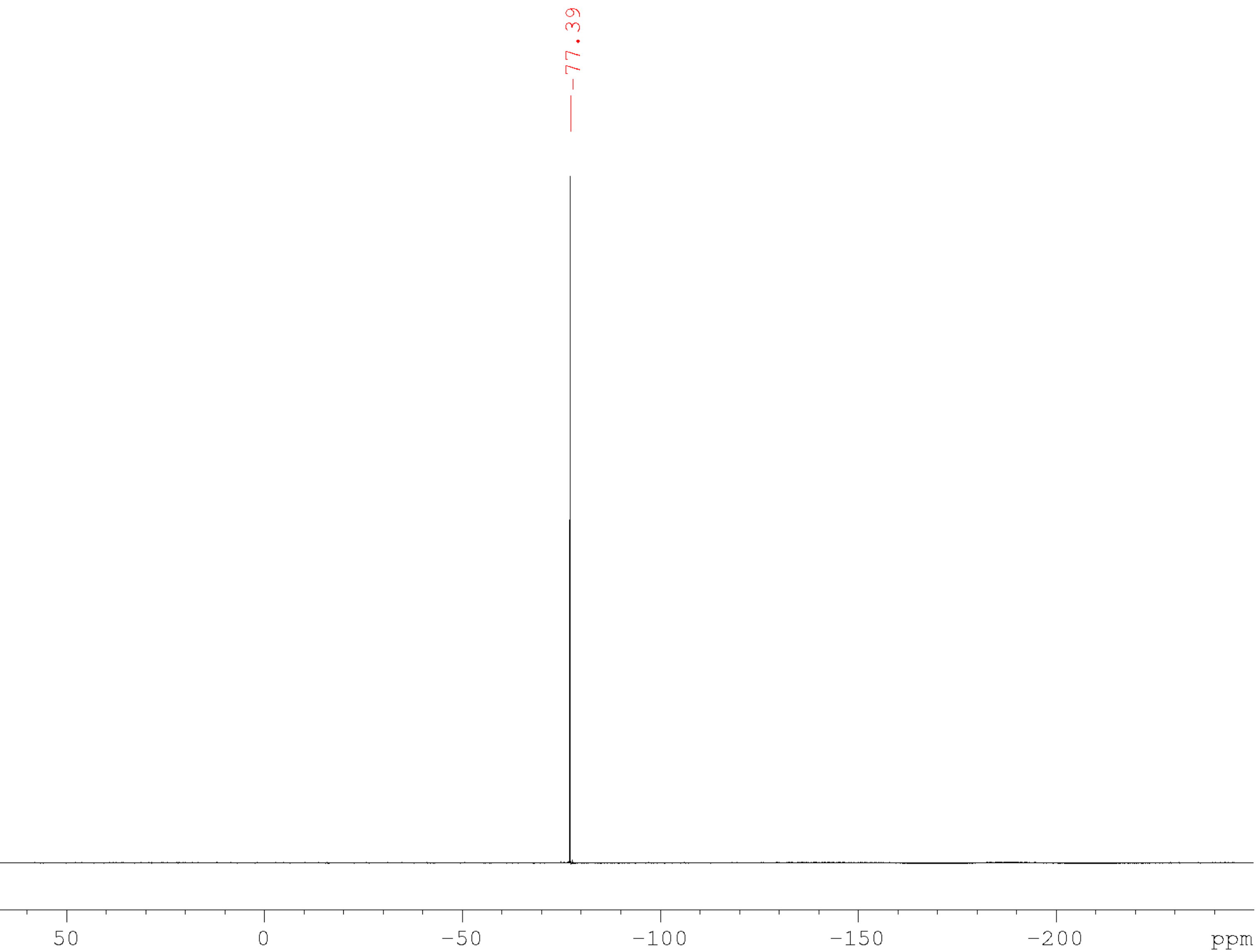

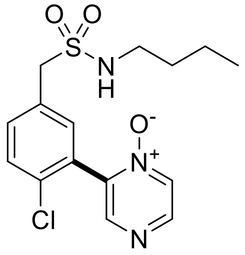

^{1}H NMR (400 MHZ, $CDCl_3$) **(8)** 2-(5-((N-butylsulfamoyl)methyl)-2-chlorophenyl)pyrazine 1-oxide

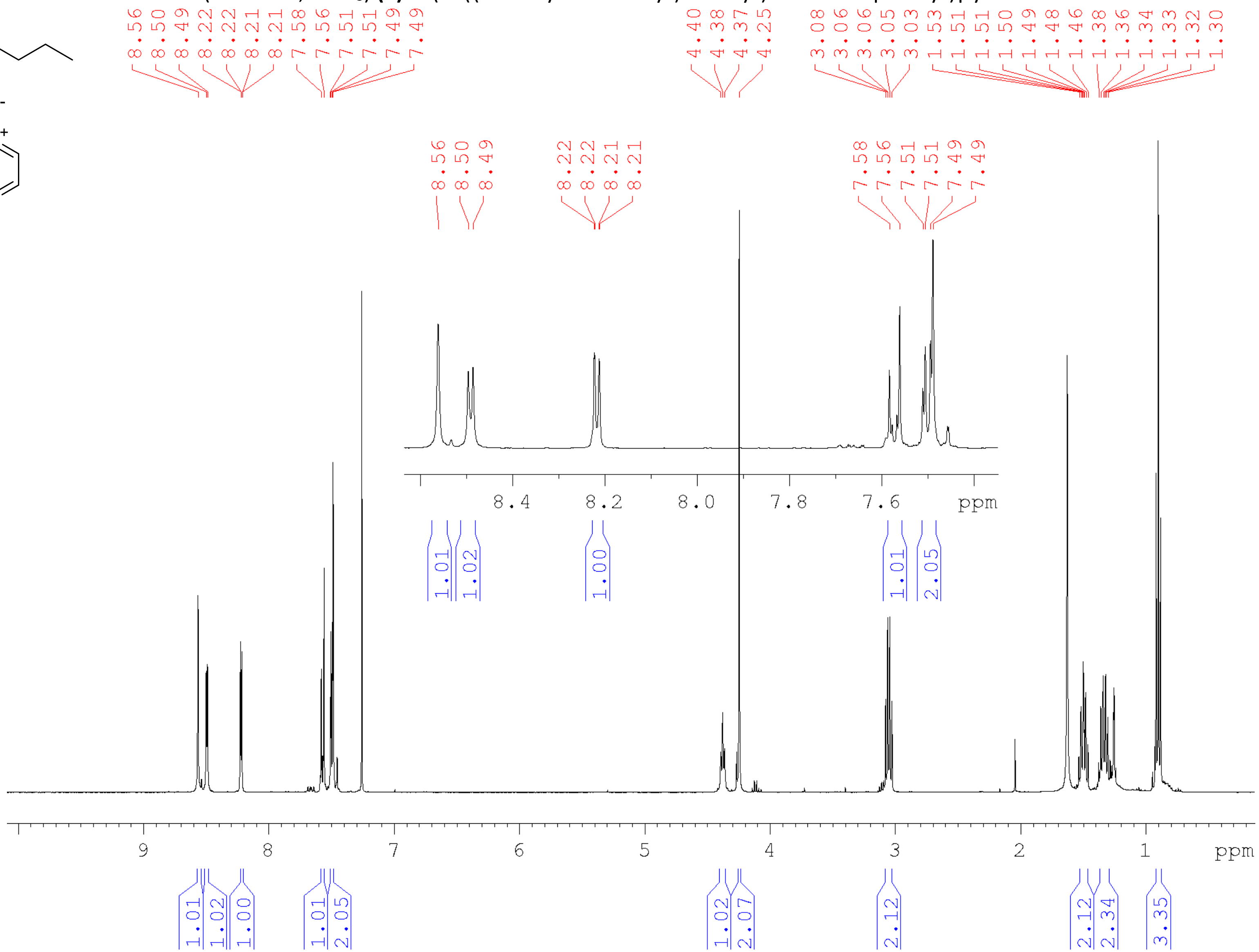

^{13}C NMR (101 MHZ, $CDCl_3$) **(8)** 2-(5-((N-butylsulfamoyl)methyl)-2-chlorophenyl)pyrazine 1-oxide

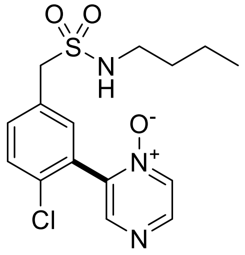

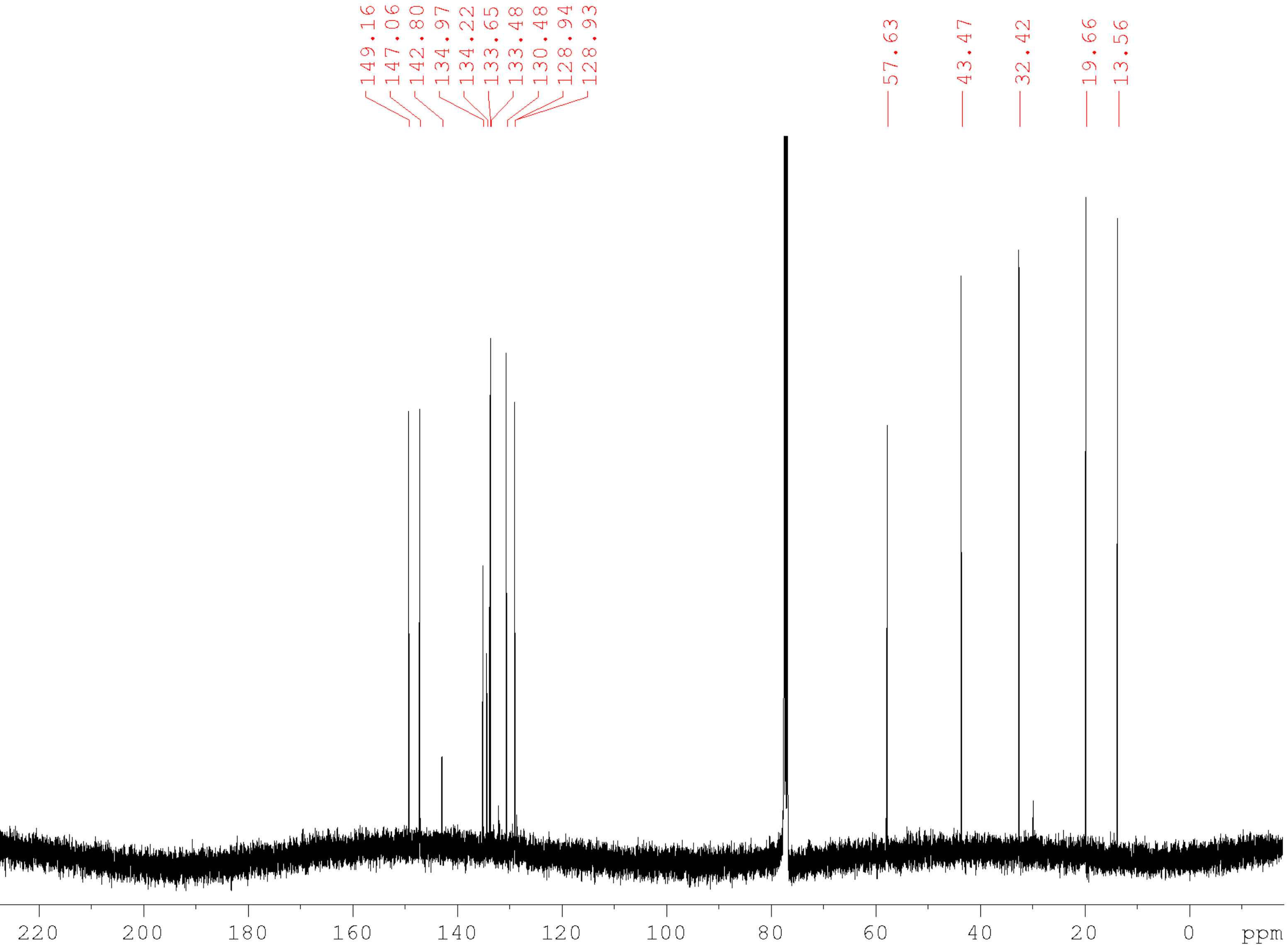

Supplement: SC-011-D0SC00105H-s001 [file SC-011-D0SC00105H-s001.pdf]
